# Association between Sleep Traits and Lung Cancer: A Mendelian Randomization Study

**DOI:** 10.1155/2021/1893882

**Published:** 2021-06-21

**Authors:** Jie Wang, Haibo Tang, Yumei Duan, Siyu Yang, Jian An

**Affiliations:** ^1^Health Management Center, The Third Xiangya Hospital, Central South University, Changsha 410013, China; ^2^Department of Cardiology, The Third Xiangya Hospital, Central South University, Changsha 410013, China; ^3^Department of Metabolic and Bariatric Surgery, The Third Xiangya Hospital, Central South University, Changsha 410013, China; ^4^Department of Pathology, Xiangya Hospital, Central South University, Changsha 410008, China; ^5^Suzhou Science and Technology Town Foreign Language School, China; ^6^Department of Respiratory Medicine, Xiangya Hospital, Central South University, Changsha 410008, China

## Abstract

Multidimensional sleep trait, which is related to circadian rhythms closely, affects some cancers predominantly, while the relationship between sleep and lung cancer is rarely illustrated. We aimed to investigate whether sleep is causally associated with risk of lung cancer, through a two-sample Mendelian randomization study. The main analysis used publicly available GWAS summary data from two large consortia (UK Biobank and International Lung Cancer Consortium). Two-sample Mendelian randomization (MR) analysis was used to examine whether chronotype, getting up in the morning, sleep duration, nap during the day, or sleeplessness was causally associated with the risk of lung cancer. Additionally, multivariate MR analysis was also conducted to estimate the direct effects between sleep traits and lung cancer risks independent of smoking status including pack years of smoking or current tobacco smoking. There was no evidence of causal association between chronotype, getting up in the morning, or nap during the day and lung cancer. Sleeplessness was associated with higher risk of lung adenocarcinoma (odds ratio 5.75, 95% confidence intervals 2.12-15.65), while sleep duration played a protective role in lung cancer (0.46, 0.26-0.83). In multivariate MR analysis, sleeplessness and sleep duration remained to have similar results. In conclusion, we found robust evidence for effect of sleeplessness on lung adenocarcinoma risk and inconsistent evidence for a protective effect of sleep duration on lung cancer risk.

## 1. Introduction

Lung cancer, which accounts for 11.6% of all newly diagnosed cancer cases and 18.4% of cancer-related deaths [1], brings a growing global burden of disease. Smoking has been identified as the most common risk factor for lung cancer, and a large number of epidemiological researches support this connection [2–4]. Smoking cessation before middle age can effectively decrease lung cancer risk. However, more and more nonsmokers were diagnosed with lung cancer over the past decades [5–7]. Based on this fact, attention has been focused on modified lifestyle risk factors other than smoking, such as sleep.

Many studies have shown that sleep plays an important role in cancer by affecting circadian rhythms, especially in breast cancer [8–11]. Nevertheless, only limited observational studies illustrated associations between sleep duration and lung cancer with inconsistent results [12–16]. These inconsistent results from epidemiological studies tend to be biased by small sample size, insufficient follow-up, and many unmeasured confounding, making inaccurate causation. Meanwhile, fewer studies have examined the relationship between sleep and lung cancer at the genetic level.

Mendelian randomization (MR) can use genetic variants that are associated robustly with exposure as instrumental variables to evaluate causal effects between the modifiable risk factors and the diseases [17, 18]. The selected instrumental variables used in MR must meet three important assumptions [19] including the following: (1) SNP should be associated with sleep traits, (2) SNP should not be associated with confounding, and (3) SNP must influence lung cancer through exposure without direct association. Thus, this approach may avoid measurement error, confounding, and reverse causation that always exist in conventional clinical studies.

Furthermore, sleep is a multidimensional concept, including chronotype, getting up in the morning, sleep duration, nap during the day, and sleeplessness. Therefore, the exploration of association between sleep and lung cancer should not be finite to sleep duration. Based on the limited evidence for effects of sleep traits on lung cancer and the significant association between unfavorable sleep duration and lung function [20], we aimed to conduct a two-sample MR study to estimate the causal inferences between sleep traits and lung cancer risks.

## 2. Materials and Methods

### 2.1. GWAS Data on Exposure

Our exposure data were extracted from the UK Biobank, a large cohort study with deep genetic and phenotypic data collected on more than 500,000 individuals from across the United Kingdom [21]. Genome-wide association study (GWAS) of chronotype, getting up in the morning, sleep duration, nap during the day, sleeplessness/insomnia, pack years of smoking, and current tobacco smoking was performed among individuals of European ancestry (*n* = 413, 343-462,434). With statistically significant threshold [*P* < 5 × 10^–8^; linkage disequilibrium (LD) *r*^2^ < 0.001, LD distance > 10,000 kb], we identified single nucleotide polymorphisms (SNPs) robustly associated with sleep traits to generate genetic instruments. *F* statistic represents the strength of relationship between SNPs and sleep traits. It is related to the explained variance for exposure (*R*^2^), sample size (*n*), and number of SNPs (*k*) by the formula *F* = [(*n* − *k* − 1)/*k*]/[*R*^2^/(1 − *R*^2^)]. Generally, *F* > 10 indicating that selected SNPs may strongly predict sleep traits [22].

### 2.2. GWAS Data on Outcome

GWAS summary data of lung cancer were extracted from the International Lung Cancer Consortium (ILCCO) [23] with 27,209 participants (11,348 cases and 15,861 controls). ILCCO also provided information of histological subtypes including squamous cell cancer and adenocarcinoma. For each of the SNP associated with sleep traits, we retrieved its effect on lung cancer from ILCCO and proxy SNP (LD *r*^2^ > 0.8) from the 1000 Genomes Project, which were absent in outcome dataset.

### 2.3. Statistical Analysis

#### 2.3.1. Univariate Two-Sample MR Analysis

The associations between exposure (sleeping traits) and outcome (lung cancer) were calculated with two-sample MR analysis [24]. We used inverse variance weighted (IVW) to clarify the causal associations. We also performed the same procedure for its subtypes (squamous cell cancer and adenocarcinoma). The results were shown as odds ratios (OR) and 95% confidence intervals (CI). To account for sensitivity of results, we used MR Egger regression, weighted median [25], and weighted mode to evaluate causal association. Moreover, we performed heterogeneity test which can suggest reliability of MR estimates. We also used Egger regression intercept to estimate the magnitude of horizontal pleiotropy, which can further illustrate whether SNPs influence the lung cancer risks through the sleep traits.

To further detect causal estimates for potential violation of the MR assumptions, we also performed RadialMR [26] to ascertain outliers in MR analysis and conducted reanalysis after excluding these outliers. RadialMR analysis was conducted using modified second-order weights and an *α* level of 0.05.

#### 2.3.2. Multivariate Two-Sample MR Analysis

Considering that smoking is recognized as the common risk factor for lung cancer, we conducted IVW multivariable MR to estimate the effect of each sleeping traits after adjusting for pack years of smoking or current tobacco smoking status. To further eliminate the interaction effect between different exposures and avoid the multicollinearity, we also performed IVW multivariable MR after applying LASSO feature selection to identify effects of sleep duration, nap during the day, and sleeplessness for lung cancer. All analyses were replicated on squamous cell cancer and adenocarcinoma.

MR analyses were performed using the R package “TwoSampleMR” (version 0.5.5) in R (version 4.0.3).

## 3. Results

### 3.1. Character of SNP for Analysis

Table 1 shows the source of GWAS data. Each SNP extracted from different sleep traits and its *F* statistic and *R*^2^ are shown in Supplementary Table 3. There were 156 SNPs for chronotype, 75 for getting up in the morning, 91 for nap during the day, 70 for sleep duration, and 42 for sleeplessness. After harmonization of the SNP effects, the SNPs available in univariate two-sample MR analysis are presented in Supplementary Table 4. Finally, 147 SNPs were used to instrument chronotype, 72 for getting up in the morning, 87 for nap during the day, 65 for sleep duration, and 42 for sleeplessness. *F* statistics range from 45 to 59, representing strong instruments in the MR analysis.

### 3.2. Causal Effect from Sleeping Traits to Lung Cancer

#### 3.2.1. Lung Cancer

We found adverse effects of sleeplessness (OR 2.53, 95% CI 1.25-5.12) and protective effects of sleep duration (0.46, 0.26-0.83) on lung cancer risk. However, the effects of chronotype, getting up in the morning, and nap during the day were not statistically significant (0.98, 0.70-1.16 for chronotype; 0.99, 0.62-1.60 for getting up in the morning; 1.37, 0.77-2.24 for sleep duration).

#### 3.2.2. Squamous Cell Lung Cancer

All MR results were not statistically significant (0.87, 0.99-3.79 for chronotype; 1.08, 0.55-2.12 for getting up in the morning; 0.46, 0.18-1.18 for sleep duration; 1.07, 0.48-2.35 for nap during the day; and 2.46, 0.83-7.34 for sleeplessness).

#### 3.2.3. Lung Adenocarcinoma

We observed a strongly hazardous effect of sleeplessness (5.75, 2.12-15.65) on the risk of lung adenocarcinoma, while little evidence of causal effects of other sleeping traits was obtained (0.85, 0.61-1.2 for chronotype; 2.21, 0.81-5.99 for getting up in the morning; 0.62, 0.29-1.31 for sleep duration; and 2.04, 0.66-6.35 for nap during the day).

**Figure 1:**
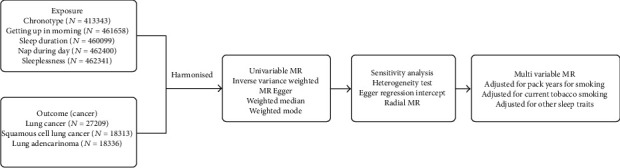
Flow diagram of Mendelian randomization.

**Figure 2:**
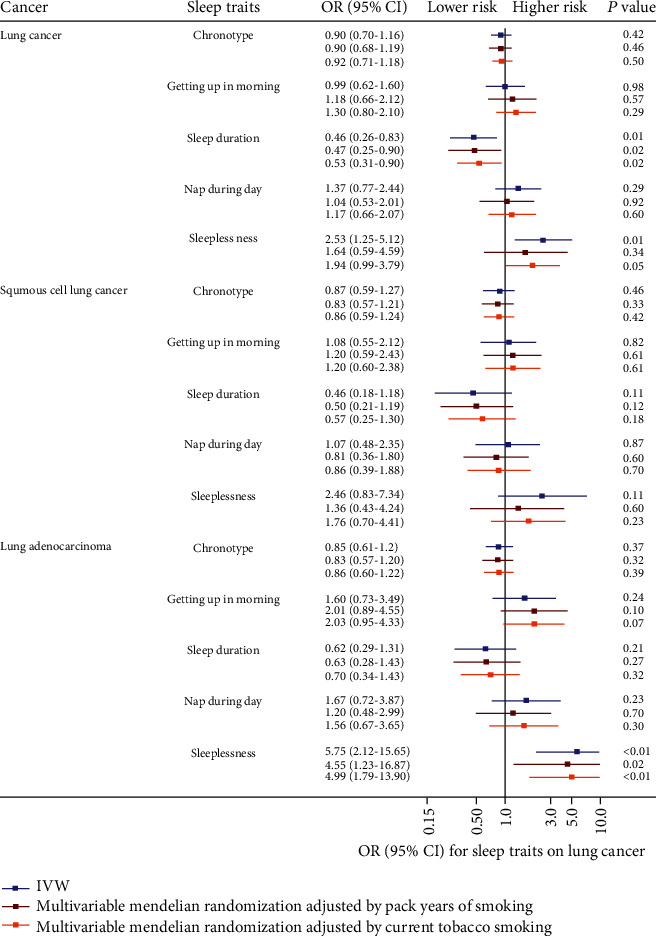
Forest plot of Mendelian randomization (MR) estimates for association between sleep traits and cancer risk. OR: odds ratios; 95% CI: 95% confidence interval; IVW: inverse variants weighted; MVMR: multivariable variant Mendelian randomization.

In multivariate MR analysis, sleeplessness still showed an adverse effect on lung adenocarcinoma adjusted for pack years of smoking (4.55, 1.23-16.87) or current tobacco smoking (4.99, 1.79-13.90), while sleep duration showed a protective influence on lung cancer adjusted for these two smoking statuses (0.47, 0.25-0.90, and 0.53, 0.31-0.90, respectively).  Figure 1 showed the study design. All MR results are shown in Table 2 and Figure 2.

Through the LASSO feature selection function, only relevant features and instruments were retained. The results of MVMR performed on remaining SNP data were also similar with univariate analysis (in Supplementary Table 6).

### 3.3. Sensitivity Analyses

Other results estimated by MR Egger, weighted median, and weighted mode are available in Supplementary Table 1. There was no evidence supporting the presence of horizontal pleiotropy in the MR Egger regression analysis (Supplementary Table 2). Heterogeneity was observed in the chronotype and nap during the day analysis. Sleep duration showed heterogeneity only in lung cancer analysis. We did not observe heterogeneity in other MR results. A detailed heterogeneity test and pleiotropy are available in Supplementary Table 2. After excluding outliers of these results with heterogeneity, MR results were consistent with the results before excluding (in Supplementary Table 5).

## 4. Discussion

In this study, we explored the causal effects of five sleep traits including chronotype, getting up in the morning, sleep duration, nap during the day, and sleeplessness on lung cancer, squamous cell lung cancer, and lung adenocarcinoma. Insomnia was causally associated with a higher risk of lung adenocarcinoma, while sleep duration showed a protective effect on lung cancer risk.

Previous epidemiological studies have just focused on the relationship between sleep duration and lung cancer. Some studies have reported the U-shaped association [13, 14], indicating that longer sleep duration and short sleep duration are both associated with unhealthy outcomes. Furthermore, a meta-analysis including 32 studies also suggested that long sleep duration increases cancer-specific mortality, especially for lung cancer [12]. However, a US male physician cohort study with a mean follow-up of 7.5 years had a different conclusion that altered sleep duration (≤6 h/day or ≥8 h/day) failed to increase lung cancer incidence. Another prospective cohort study including 21,804 participants in Canada also identified no significant effects of unfavorable sleep duration (<7 h/day or >9 h/day) while night shift work may contribute to lung cancer incidence. Unlike observational studies, our study showed that sleep duration was a protective factor for lung cancer, suggesting that longer sleep duration could decrease the risk for lung cancer.

In addition to sleep duration, other sleep traits also reflect sleep conditions; a comprehensive evaluation should contain the impacts of chronotype, getting up in the morning, and sleeplessness on lung cancer. Only Xie and his colleagues [13] explored associations of other sleep traits and lung cancer, indicating that evening chronotype also increases lung cancer risk except for unfavorable sleep duration while sleeplessness has no effects. Chronotype and getting up in the morning, related to circadian rhythms closely, were reported as risk factors for cancer such as breast cancer [8] and epithelial ovarian cancer [27]. However, compared with Xie et al.'s study, our study showed opposed findings that chronotype did not contribute to lung cancer incidence and sleep duration showed a protective effect. Given the heterogeneity of different subtypes, we replicated all analyses on other subtypes such as squamous cell lung cancer and lung adenocarcinoma. Although sleeplessness may not be harmful to lung cancer, there surprisingly appeared a strong association with lung adenocarcinoma. For the cancer patient, sleeplessness is often a common and enduring symptom [28, 29], especially for patients in the terminal stage of lung cancer [30].

The mechanisms underlying these associations are poorly understood. One possible pathway is that sleep disturbances may lead to chronic lung disease through circadian rhythm disruption [31]. Sleep deprivation leads to a more severe lung inflammation [32], which is essential for the risk of lung cancer [33]. These findings may support the adverse effect of short sleep duration sleeplessness and are consistent with our results partially. However, there is lack of evidence on the histology-specific impact of sleeplessness.

To our knowledge, this study is the first to explore connections between sleep traits and lung cancer risks at the level of genes. Although random control trial (RCT) can provide the most compelling evidence, it involves many ethical issues and costs much money. For observation studies, despite these results from observed studies that were adjusted by other relative variables, undetected biases could not be ignored. Therefore, the results provided by MR are the most convincing. Bias due to confounding and reverse sources could be decreased by MR. To minimize the potential violation of the MR assumption, we also conducted serials of sensitivity analysis and detected any outliers by RadialMR analysis. We also conducted multivariable MR to adjust for smoking, the most common and important risk factor of lung cancer.

Several limitations should be considered in our study. First, our study was based on the European population. Thus, whether our study could be generalizable to other populations requires further investigations. Second, the summary data used in our MR analyses were not stratified by gender or smoking. Finally, all sleep traits were self-reported. Thus, it is possible to lead to misclassification of exposure.

In conclusion, MR analysis provides stronger evidence for the causal effect of sleeplessness on lung adenocarcinoma and highlights the importance of sleep duration in lung cancer incidence. Although other sleep traits did not show protective or adverse effects on lung cancer, these findings imply that we still need to pay attention to sleep health to mitigate the risk of incident lung cancer. Our results may further emphasize the importance of enough sleep for health. Further studies are needed to illustrate the association between sleep traits and lung cancer in females and nonsmokers.

## Figures and Tables

**(a):** 

Exposure	Consortium	Sample size	Population
Chronotype	MRC-IEU	413343	European
Getting up in the morning	MRC-IEU	461658	European
Sleep duration	MRC-IEU	460099	European
Nap during the day	MRC-IEU	462400	European
Sleeplessness/insomnia	MRC-IEU	462341	European
Current tobacco smoking	MRC-IEU	462434	European
Pack years of smoking	MRC-IEU	142387	European

**(b):** 

Outcomes	Consortium	Cases/control	Sample size	Population
Lung cancer	ILCCO	11348/15861	27209	European
Squamous cell cancer	ILCCO	3275/15038	18313	European
Adenocarcinoma	ILCCO	3442/14894	18336	European

**Table 2:** Two-sample Mendelian randomization estimations showing the effect of sleep traits on cancer using the IVW method.

Exposure	Method	OR (95% CI)	*P* value
Chronotype	Inverse variance weighted	0.90 (0.70-1.16)	0.42
	MVMR adjusted for pack years for smoking	0.90 (0.68-1.19)	0.46
	MVMR adjusted for current tobacco smoking	0.92 (0.71-1.18)	0.50
Getting up in the morning	Inverse variance weighted	0.99 (0.62-1.60)	0.98
	MVMR adjusted for pack years for smoking	1.18 (0.66-2.12)	0.57
	MVMR adjusted for current tobacco smoking	1.30 (0.80-2.10)	0.29
Sleep duration	Inverse variance weighted	0.46 (0.26-0.83)	0.01
	MVMR adjusted for pack years for smoking	0.47 (0.25-0.90)	0.02
	MVMR adjusted for current tobacco smoking	0.53 (0.31-0.90)	0.02
Nap during the day	Inverse variance weighted	1.37 (0.77-2.44)	0.29
	MVMR adjusted for pack years for smoking	1.04 (0.53-2.01)	0.92
	MVMR adjusted for current tobacco smoking	1.17 (0.66-2.07)	0.60
Sleeplessness	Inverse variance weighted	2.53 (1.25-5.12)	0.01
	MVMR adjusted for pack years for smoking	1.64 (0.59-4.59)	0.34
	MVMR adjusted for current tobacco smoking	1.94 (0.99-3.79)	0.05
Chronotype	Inverse variance weighted	0.87 (0.59-1.27)	0.46
	MVMR adjusted for pack years for smoking	0.83 (0.57-1.21)	0.33
	MVMR adjusted for current tobacco smoking	0.86 (0.59-1.24)	0.42
Getting up in the morning	Inverse variance weighted	1.08 (0.55-2.12)	0.82
	MVMR adjusted for pack years for smoking	1.20 (0.59-2.43)	0.61
	MVMR adjusted for current tobacco smoking	1.20 (0.60-2.38)	0.61
Sleep duration	Inverse variance weighted	0.46 (0.18-1.18)	0.11
	MVMR adjusted for pack years for smoking	0.50 (0.21-1.19)	0.12
	MVMR adjusted for current tobacco smoking	0.57 (0.25-1.30)	0.18
Nap during the day	Inverse variance weighted	1.07 (0.48-2.35)	0.87
	MVMR adjusted for pack years for smoking	0.81 (0.36-1.80)	0.60
	MVMR adjusted for current tobacco smoking	0.86 (0.39-1.88)	0.70
Sleeplessness	Inverse variance weighted	2.46 (0.83-7.34)	0.11
	MVMR adjusted for pack years for smoking	1.36 (0.43-4.24)	0.60
	MVMR adjusted for current tobacco smoking	1.76 (0.70-4.41)	0.23
Chronotype	Inverse variance weighted	0.85 (0.61-1.20)	0.37
	MVMR adjusted for pack years for smoking	0.83 (0.57-1.20)	0.32
	MVMR adjusted for current tobacco smoking	0.86 (0.60-1.22)	0.39
Getting up in the morning	Inverse variance weighted	1.60 (0.73-3.49)	0.24
	MVMR adjusted for pack years for smoking	2.01 (0.89-4.55)	0.10
	MVMR adjusted for current tobacco smoking	2.03 (0.95-4.33)	0.07
Sleep duration	Inverse variance weighted	0.62 (0.29-1.31)	0.21
	MVMR adjusted for pack years for smoking	0.63 (0.28-1.43)	0.27
	MVMR adjusted for current tobacco smoking	0.70 (0.34-1.43)	0.32
Nap during the day	Inverse variance weighted	1.67 (0.72-3.87)	0.23
	MVMR adjusted for pack years for smoking	1.20 (0.48-2.99)	0.70
	MVMR adjusted for current tobacco smoking	1.56 (0.67-3.65)	0.30
Sleeplessness	Inverse variance weighted	5.75 (2.12-15.65)	<0.01
	MVMR adjusted for pack years for smoking	4.55 (1.23-16.87)	0.02
	MVMR adjusted for current tobacco smoking	4.99 (1.79-13.90)	<0.01

OR: odds ratios; 95% CI: 95% confidence interval; IVW: inverse variants weighted; MVMR: multivariable variant Mendelian randomization.

## Data Availability

Our data was from the UK Biobank and the International Lung Cancer Consortium, the two open-access datasets (https://www.mrbase.org/;https://www.ukbiobank.ac.uk/;https://ilcco.iarc.fr/).

## Supplementary Materials

Supplementary Materials Supplementary Table 1: two-sample Mendelian randomization estimations showing the effect of sleep traits on cancer using the MR Egger, weighted median, and weighted mode method. Supplementary Table 2: sensitivity analysis performed by Egger regression intercept and heterogeneity test. Supplementary Table 3: SNPs of sleep traits extracted from UK Biobank with statistically significant threshold [*P* < 5 × 10^−8^; linkage disequilibrium (LD) *r*^2^ < 0.001, LD distance > 10000 kb]. Supplementary Table 4: SNPs used in two-sample Mendelian randomization analysis. Supplementary Table 5: outliers selected by RadialMR and the reanalysis results after excluding outliers. Supplementary Table 6: multivariable two-sample Mendelian randomization estimation showing the effects of different sleep traits on lung cancer.
