# Supplementary material for: Association between Sleep Traits and Lung Cancer: A Mendelian Randomization Study
Source: J Immunol Res. 2021 Jun 21;2021:1893882. doi: 10.1155/2021/1893882 (PMC8238591; doi:10.1155/2021/1893882)
Supplement: Supplementary Materials — Supplementary Table 1: two-sample Mendelian randomization estimations showing the effect of sleep traits on cancer using the MR Egger, weighted median, and weighted mode method. Supplementary Table 2: sensitivity analysis performed by Egger regression intercept and heterogeneity test. Supplementary Table 3: SNPs of sleep traits extracted from UK Biobank with statistically significant threshold [P < 5 × 10^−8^; linkage disequilibrium (LD) r2 < 0.001, LD distance > 10000 kb]. Supplementary Table 4: SNPs used in two-sample Mendelian randomization analysis. Supplementary Table 5: outliers selected by RadialMR and the reanalysis results after excluding outliers. Supplementary Table 6: multivariable two-sample Mendelian randomization estimation showing the effects of different sleep traits on lung cancer. [file 1893882.f1.zip › Supplementary Table 1 (1).docx]

Supplementary Table 1:Two-sample Mendelian randomization estimations showing the effect of sleep traits on cancer using MR egger, Weighted median, and Weighted mode method.

| Outcomes | **Exposure** | **Method** | **OR (95% CI)** | **P-value** |
| --- | --- | --- | --- | --- |
| **Lung cancer** | Chronotype | MR Egger | 0.54 (0.24-1.21) | 0.14 |
|  |  | Weighted median | 0.98 (0.68-1.39) | 0.90 |
|  |  | Weighted mode | 1.42 (0.43-4.73) | 0.56 |
|  | Getting up in morning | MR Egger | 3.80 (0.67-21.58) | 0.14 |
|  |  | Weighted median | 1.17 (0.62-2.23) | 0.63 |
|  |  | Weighted mode | 1.17 (0.26-5.33) | 0.84 |
|  | Sleep duration | MR Egger | 1.40 (0.16-11.92) | 0.76 |
|  |  | Weighted median | 0.65 (0.32-1.32) | 0.24 |
|  |  | Weighted mode | 0.84 (0.25-2.83) | 0.78 |
|  | Nap during day | MR Egger | 3.41 (0.42-27.91) | 0.26 |
|  |  | Weighted median | 2.09 (1.02-4.29) | 0.04 |
|  |  | Weighted mode | 3.12 (0.78-12.46) | 0.11 |
|  | Sleeplessness | MR Egger | 1.35 (0.16-11.09) | 0.78 |
|  |  | Weighted median | 1.77 (0.66-4.73) | 0.25 |
|  |  | Weighted mode | 2.00 (0.49-8.11) | 0.34 |
| **Squamous cell lung cancer** | Chronotype | MR Egger | 0.96 (0.29-3.20) | 0.95 |
|  |  | Weighted median | 0.66 (0.39-1.12) | 0.13 |
|  |  | Weighted mode | 0.27 (0.07-1.07) | 0.06 |
|  | Getting up in morning | MR Egger | 3.99 (0.34-46.52) | 0.27 |
|  |  | Weighted median | 0.96 (0.37-2.52) | 0.94 |
|  |  | Weighted mode | 1.45 (0.18-12.00) | 0.73 |
|  | Sleep duration | MR Egger | 4.70 (0.16-137.43) | 0.69 |
|  |  | Weighted median | 0.72 (0.24-2.16) | 0.84 |
|  |  | Weighted mode | 1.24 (0.18-8.54) | 0.94 |
|  | Nap during day | MR Egger | 1.95 (0.11-34.99) | 0.65 |
|  |  | Weighted median | 1.71 (0.56-5.17) | 0.34 |
|  |  | Weighted mode | 5.33 (0.51-55.43) | 0.17 |
|  | Sleeplessness | MR Egger | 0.52 (0.02-12.08) | 0.69 |
|  |  | Weighted median | 1.15 (0.30-4.47) | 0.84 |
|  |  | Weighted mode | 1.07 (0.16-7.07) | 0.94 |
| Lung Adenocarcinoma | Chronotype | MR Egger | 0.37 (0.13-1.09) | 0.07 |
|  |  | Weighted median | 0.95 (0.58-1.57) | 0.85 |
|  |  | Weighted mode | 1.52 (0.5-4.59) | 0.46 |
|  | Getting up in morning | MR Egger | 11.78 (0.69-200.76) | 0.09 |
|  |  | Weighted median | 2.21 (0.81-5.99) | 0.12 |
|  |  | Weighted mode | 5.48 (0.50-60.05) | 0.17 |
|  | Sleep duration | MR Egger | 2.28 (0.15-34.94) | 0.56 |
|  |  | Weighted median | 0.78 (0.26-2.33) | 0.66 |
|  |  | Weighted mode | 0.76 (0.14-4.08) | 0.75 |
|  | Nap during day | MR Egger | 4.85 (0.23-104.31) | 0.32 |
|  |  | Weighted median | 2.04 (0.66-6.35) | 0.22 |
|  |  | Weighted mode | 2.66 (0.36-19.87) | 0.34 |
|  | Sleeplessness | MR Egger | 1.39 (0.07-27.60) | 0.83 |
|  |  | Weighted median | 3.76 (0.95-14.96) | 0.06 |
|  |  | Weighted mode | 5.33 (0.56-50.46) | 0.15 |
