# Supplementary material for: Association between Sleep Traits and Lung Cancer: A Mendelian Randomization Study
Source: J Immunol Res. 2021 Jun 21;2021:1893882. doi: 10.1155/2021/1893882 (PMC8238591; doi:10.1155/2021/1893882)
Supplement: Supplementary Materials — Supplementary Table 1: two-sample Mendelian randomization estimations showing the effect of sleep traits on cancer using the MR Egger, weighted median, and weighted mode method. Supplementary Table 2: sensitivity analysis performed by Egger regression intercept and heterogeneity test. Supplementary Table 3: SNPs of sleep traits extracted from UK Biobank with statistically significant threshold [P < 5 × 10^−8^; linkage disequilibrium (LD) r2 < 0.001, LD distance > 10000 kb]. Supplementary Table 4: SNPs used in two-sample Mendelian randomization analysis. Supplementary Table 5: outliers selected by RadialMR and the reanalysis results after excluding outliers. Supplementary Table 6: multivariable two-sample Mendelian randomization estimation showing the effects of different sleep traits on lung cancer. [file 1893882.f1.zip › Supplementary Table 2 (2).docx]

Supplementary Table 2: sensitivity analysis performed by egger regression intercept and heterogeneity test.

| **Outcomes** | **Exposure** | **Method** | **Q** | **Q-value** | **Egger intercept** | **Egger intercept-p value** |  |  |
| --- | --- | --- | --- | --- | --- | --- | --- | --- |
| **Lung cancer** | Chronotype | MR Egger | 162.72 | 0.02 | 0.01 | 0.19 |  |  |
|  |  | Inverse variance weighted | 164.90 | 0.02 |  |  |  |  |
|  | Getting up in morning | MR Egger | 71.54 | 0.17 | -0.02 | 0.12 |  |  |
|  |  | Inverse variance weighted | 74.44 | 0.13 |  |  |  |  |
|  | Sleep duration | MR Egger | 88.70 | <0.01 | -0.01 | 0.30 |  |  |
|  |  | Inverse variance weighted | 90.54 | <0.01 |  |  |  |  |
|  | Nap during day | MR Egger | 113.53 | <0.01 | -0.01 | 0.38 |  |  |
|  |  | Inverse variance weighted | 114.68 | <0.01 |  |  |  |  |
|  | Sleeplessness | MR Egger | 39.48 | 0.12 | 0.01 | 0.54 |  |  |
|  |  | Inverse variance weighted | 39.99 | 0.13 |  |  |  |  |
| **Squamous cell lung cancer** | Chronotype | MR Egger | 160.88 | 0.02 | <0.01 | 0.85 |  |  |
|  |  | Inverse variance weighted | 160.92 | 0.03 |  |  |  |  |
|  | Getting up in morning | MR Egger | 56.51 | 0.64 | -0.02 | 0.28 |  |  |
|  |  | Inverse variance weighted | 57.68 | 0.63 |  |  |  |  |
|  | Sleep duration | MR Egger | 92.81 | <0.01 | -0.03 | 0.17 |  |  |
|  |  | Inverse variance weighted | 96.23 | <0.01 |  |  |  |  |
|  | Nap during day | MR Egger | 90.05 | 0.15 | -0.01 | 0.67 |  |  |
|  |  | Inverse variance weighted | 90.26 | 0.16 |  |  |  |  |
|  | Sleeplessness | MR Egger | 40.12 | 0.10 | 0.02 | 0.31 |  |  |
|  |  | Inverse variance weighted | 41.55 | 0.10 |  |  |  |  |
| Lung **Adenocarcinoma** | Chronotype | MR Egger | 121.27 | 0.63 | 0.01 | 0.85 |  |  |
|  |  | Inverse variance weighted | 123.80 | 0.59 |  |  |  |  |
|  | Getting up in morning | MR Egger | 81.86 | 0.04 | -0.03 | 0.28 |  |  |
|  |  | Inverse variance weighted | 84.62 | 0.03 |  |  |  |  |
|  | Sleep duration | MR Egger | 62.31 | 0.18 | -0.02 | 0.17 |  |  |
|  |  | Inverse variance weighted | 63.43 | 0.18 |  |  |  |  |
|  | Nap during day | MR Egger | 104.03 | 0.02 | -0.01 | 0.67 |  |  |
|  |  | Inverse variance weighted | 104.71 | 0.02 |  |  |  |  |
|  | Sleeplessness | MR Egger | 32.99 | 0.32 | 0.02 | 0.31 |  |  |
|  |  | Inverse variance weighted | 34.07 | 0.32 |  |  |  |  |
