# Supplementary material for: Association between Sleep Traits and Lung Cancer: A Mendelian Randomization Study
Source: J Immunol Res. 2021 Jun 21;2021:1893882. doi: 10.1155/2021/1893882 (PMC8238591; doi:10.1155/2021/1893882)
Supplement: Supplementary Materials — Supplementary Table 1: two-sample Mendelian randomization estimations showing the effect of sleep traits on cancer using the MR Egger, weighted median, and weighted mode method. Supplementary Table 2: sensitivity analysis performed by Egger regression intercept and heterogeneity test. Supplementary Table 3: SNPs of sleep traits extracted from UK Biobank with statistically significant threshold [P < 5 × 10^−8^; linkage disequilibrium (LD) r2 < 0.001, LD distance > 10000 kb]. Supplementary Table 4: SNPs used in two-sample Mendelian randomization analysis. Supplementary Table 5: outliers selected by RadialMR and the reanalysis results after excluding outliers. Supplementary Table 6: multivariable two-sample Mendelian randomization estimation showing the effects of different sleep traits on lung cancer. [file 1893882.f1.zip › Supplementary Table 3 (2).docx]

Supplementary Table 3: SNPs of sleep traits extracted from UK Biobank with statistically significant threshold [P < 5×10–8; linkage disequilibrium (LD) r2 <0.001, LD distance > 10000 kb].

| **exposure** | **chr.exposure** | **SNP** | **Effect Allele** | **Alternate Allele** | **EAF** | **Sample size per SNP** | **Beta** | **SE** | **p-value** | **r2** | **F statistic** | **Overall F statistic** |
| --- | --- | --- | --- | --- | --- | --- | --- | --- | --- | --- | --- | --- |
| Getting up in morning | 1 | rs12044778 | A | G | 0.180076 | 461658 | -0.011657 | 0.00208425 | 2.20E-08 | 0.008235011 | 8.824226391 | 48.87476024 |
| Getting up in morning | 1 | rs10518446 | C | G | 0.162759 | 461658 | 0.0196877 | 0.00216558 | 9.80E-20 | 0.013378914 | 14.4109196 |  |
| Getting up in morning | 1 | rs3766163 | C | T | 0.272949 | 461658 | 0.0103943 | 0.00179234 | 6.70E-09 | 0.008533437 | 9.146756903 |  |
| Getting up in morning | 1 | rs10779704 | C | A | 0.624521 | 461658 | 0.0095243 | 0.00166155 | 9.90E-09 | 0.008436536 | 9.042007778 |  |
| Getting up in morning | 1 | rs12752290 | C | T | 0.442454 | 461658 | 0.0116401 | 0.00161486 | 5.70E-13 | 0.010607153 | 11.39334626 |  |
| Getting up in morning | 1 | rs141391319 | A | G | 0.031987 | 461658 | 0.0347714 | 0.00452675 | 1.60E-14 | 0.011301424 | 12.1476002 |  |
| Getting up in morning | 1 | rs10462020 | G | T | 0.195744 | 461658 | 0.0165379 | 0.00200975 | 1.90E-16 | 0.012109178 | 13.02647613 |  |
| Getting up in morning | 1 | rs12736689 | C | T | 0.030093 | 461658 | 0.0525952 | 0.00469226 | 3.70E-29 | 0.016494268 | 17.82285924 |  |
| Getting up in morning | 2 | rs17777135 | A | G | 0.199867 | 461658 | 0.0115293 | 0.00201572 | 1.10E-08 | 0.008410212 | 9.013554969 |  |
| Getting up in morning | 2 | rs147325583 | T | C | 0.516407 | 461658 | -0.0107449 | 0.00159864 | 1.80E-11 | 0.00989186 | 10.61736086 |  |
| Getting up in morning | 2 | rs406952 | C | T | 0.376409 | 461658 | 0.0111325 | 0.00164876 | 1.50E-11 | 0.009930882 | 10.65966414 |  |
| Getting up in morning | 2 | rs1470503 | T | C | 0.4276 | 461658 | -0.0128159 | 0.0016125 | 1.90E-15 | 0.011696395 | 12.57716812 |  |
| Getting up in morning | 2 | rs4483990 | C | A | 0.157001 | 461658 | -0.0183869 | 0.0022034 | 7.10E-17 | 0.012281524 | 13.21418255 |  |
| Getting up in morning | 2 | rs1223149 | C | T | 0.803843 | 461658 | 0.0142946 | 0.0020143 | 1.30E-12 | 0.010440661 | 11.21262707 |  |
| Getting up in morning | 2 | rs6745423 | A | T | 0.721988 | 461658 | -0.012839 | 0.00178363 | 6.10E-13 | 0.010593575 | 11.37860613 |  |
| Getting up in morning | 2 | rs1606803 | T | C | 0.287134 | 461658 | 0.0114168 | 0.00176337 | 9.50E-11 | 0.009528868 | 10.22399675 |  |
| Getting up in morning | 2 | rs114443104 | A | G | 0.058491 | 461658 | 0.01923 | 0.00345721 | 2.70E-08 | 0.00818257 | 8.767569525 |  |
| Getting up in morning | 2 | rs10175975 | T | C | 0.18154 | 461658 | 0.0122931 | 0.00208479 | 3.70E-09 | 0.008678829 | 9.30396327 |  |
| Getting up in morning | 3 | rs145831787 | T | C | 0.11301 | 461658 | -0.0169965 | 0.00252122 | 1.60E-11 | 0.009917103 | 10.6447256 |  |
| Getting up in morning | 4 | rs13116306 | T | C | 0.421435 | 461658 | -0.00957982 | 0.00162761 | 4.00E-09 | 0.008659871 | 9.283461655 |  |
| Getting up in morning | 4 | rs45510091 | G | A | 0.054615 | 461658 | 0.0204309 | 0.0035079 | 5.70E-09 | 0.008573249 | 9.189799216 |  |
| Getting up in morning | 5 | rs4958317 | A | G | 0.289184 | 461658 | 0.0145939 | 0.00176188 | 1.20E-16 | 0.012189942 | 13.11442982 |  |
| Getting up in morning | 5 | rs13155750 | G | A | 0.233844 | 461658 | -0.0121208 | 0.00188507 | 1.30E-10 | 0.009458915 | 10.14822387 |  |
| Getting up in morning | 6 | rs9399613 | T | C | 0.289087 | 461658 | -0.0110033 | 0.0017711 | 5.20E-10 | 0.009143666 | 9.806880212 |  |
| Getting up in morning | 6 | rs10455248 | T | C | 0.282109 | 461658 | -0.0099208 | 0.00178212 | 2.60E-08 | 0.008192258 | 8.778035992 |  |
| Getting up in morning | 6 | rs2653355 | A | C | 0.823231 | 461658 | -0.0236437 | 0.00209564 | 1.60E-29 | 0.016603098 | 17.94244039 |  |
| Getting up in morning | 6 | rs12523700 | A | T | 0.117842 | 461658 | 0.0144542 | 0.00250964 | 8.40E-09 | 0.008477443 | 9.086225223 |  |
| Getting up in morning | 6 | rs72895663 | G | A | 0.23113 | 461658 | 0.0110153 | 0.00189208 | 5.80E-09 | 0.00856897 | 9.185172863 |  |
| Getting up in morning | 6 | rs620598 | G | A | 0.225728 | 461658 | 0.0110675 | 0.00191416 | 7.40E-09 | 0.008508872 | 9.120200985 |  |
| Getting up in morning | 7 | rs1914397 | A | T | 0.448253 | 461658 | 0.0103906 | 0.0016104 | 1.10E-10 | 0.009496228 | 10.18863999 |  |
| Getting up in morning | 7 | rs112613078 | G | A | 0.191237 | 461658 | 0.0124877 | 0.00202865 | 7.50E-10 | 0.009058631 | 9.714844223 |  |
| Getting up in morning | 7 | rs2971970 | G | T | 0.782088 | 461658 | -0.0107092 | 0.00193586 | 3.20E-08 | 0.00813882 | 8.720306918 |  |
| Getting up in morning | 8 | rs7833021 | T | C | 0.772533 | 461658 | 0.0113061 | 0.00191103 | 3.30E-09 | 0.008706581 | 9.333975137 |  |
| Getting up in morning | 8 | rs17716502 | T | C | 0.204344 | 461658 | 0.0109469 | 0.00199543 | 4.10E-08 | 0.008074605 | 8.650943958 |  |
| Getting up in morning | 8 | rs2360802 | T | A | 0.225184 | 461658 | 0.012836 | 0.00191626 | 2.10E-11 | 0.009858783 | 10.58150347 |  |
| Getting up in morning | 8 | rs9644465 | A | G | 0.212173 | 461658 | 0.0115942 | 0.00195424 | 3.00E-09 | 0.008729633 | 9.358905865 |  |
| Getting up in morning | 8 | rs2044742 | A | G | 0.131812 | 461658 | -0.0134326 | 0.00236126 | 1.30E-08 | 0.008368304 | 8.968262085 |  |
| Getting up in morning | 8 | rs73179222 | A | G | 0.37177 | 461658 | 0.0100968 | 0.00165786 | 1.10E-09 | 0.008968889 | 9.617730247 |  |
| Getting up in morning | 9 | rs77641763 | T | C | 0.122477 | 461658 | -0.0185204 | 0.00243328 | 2.70E-14 | 0.011202335 | 12.03988543 |  |
| Getting up in morning | 9 | rs1854558 | A | G | 0.262243 | 461658 | -0.0104995 | 0.00181939 | 7.90E-09 | 0.008492672 | 9.102687706 |  |
| Getting up in morning | 10 | rs7899208 | C | T | 0.873766 | 461658 | 0.0136082 | 0.00240256 | 1.50E-08 | 0.008332246 | 8.929293488 |  |
| Getting up in morning | 11 | rs73608603 | G | A | 0.131243 | 461658 | -0.0134609 | 0.00236786 | 1.30E-08 | 0.008368304 | 8.968262085 |  |
| Getting up in morning | 11 | rs7105482 | G | A | 0.394887 | 461658 | 0.0089503 | 0.00163468 | 4.40E-08 | 0.008056218 | 8.631084548 |  |
| Getting up in morning | 11 | rs17152364 | G | A | 0.300997 | 461658 | -0.0118163 | 0.00174884 | 1.40E-11 | 0.009945624 | 10.67564743 |  |
| Getting up in morning | 12 | rs17464772 | A | G | 0.352041 | 461658 | 0.0125433 | 0.00167464 | 6.90E-14 | 0.011022576 | 11.84453315 |  |
| Getting up in morning | 12 | rs12227309 | T | C | 0.246638 | 461658 | 0.0118489 | 0.00185018 | 1.50E-10 | 0.009426838 | 10.11348201 |  |
| Getting up in morning | 12 | rs61926781 | T | C | 0.059112 | 461658 | -0.0194724 | 0.00338623 | 8.90E-09 | 0.00846307 | 9.070688678 |  |
| Getting up in morning | 12 | rs1017168 | C | A | 0.64442 | 461658 | 0.0104984 | 0.00167444 | 3.60E-10 | 0.00922829 | 9.898487677 |  |
| Getting up in morning | 13 | rs1333536 | T | C | 0.384778 | 461658 | -0.00921206 | 0.00164775 | 2.30E-08 | 0.008223653 | 8.811954565 |  |
| Getting up in morning | 13 | rs3125735 | T | C | 0.199178 | 461658 | -0.0120184 | 0.00199962 | 1.90E-09 | 0.00883932 | 9.477549098 |  |
| Getting up in morning | 13 | rs9573982 | C | T | 0.05141 | 461658 | -0.0206352 | 0.00362074 | 1.20E-08 | 0.008388407 | 8.98998781 |  |
| Getting up in morning | 14 | rs7144028 | C | A | 0.499138 | 461658 | -0.00903684 | 0.00159722 | 1.50E-08 | 0.008332246 | 8.929293488 |  |
| Getting up in morning | 14 | rs77556698 | T | G | 0.21591 | 461658 | -0.0117773 | 0.00194498 | 1.40E-09 | 0.008911934 | 9.556105748 |  |
| Getting up in morning | 14 | rs17112198 | G | A | 0.298533 | 461658 | 0.0101831 | 0.00174461 | 5.30E-09 | 0.008591107 | 9.209107849 |  |
| Getting up in morning | 14 | rs34757401 | G | A | 0.226798 | 461658 | 0.0108337 | 0.00192296 | 1.80E-08 | 0.008286093 | 8.879420535 |  |
| Getting up in morning | 14 | rs17766755 | A | G | 0.360087 | 461658 | -0.00946774 | 0.00166437 | 1.30E-08 | 0.008368304 | 8.968262085 |  |
| Getting up in morning | 15 | rs11629621 | G | C | 0.577452 | 461658 | -0.011935 | 0.00162498 | 2.10E-13 | 0.010805532 | 11.60875607 |  |
| Getting up in morning | 16 | rs1420607 | A | G | 0.272429 | 461658 | 0.0121786 | 0.0017983 | 1.30E-11 | 0.009961425 | 10.69277827 |  |
| Getting up in morning | 16 | rs1421085 | C | T | 0.40345 | 461658 | 0.0109591 | 0.0016269 | 1.60E-11 | 0.009917103 | 10.6447256 |  |
| Getting up in morning | 16 | rs7206027 | T | A | 0.385657 | 461658 | -0.00974046 | 0.00163897 | 2.80E-09 | 0.008746283 | 9.3769137 |  |
| Getting up in morning | 16 | rs11075924 | A | C | 0.495783 | 461658 | -0.00994218 | 0.00159828 | 5.00E-10 | 0.009152725 | 9.816686757 |  |
| Getting up in morning | 17 | rs7210219 | C | T | 0.224138 | 461658 | 0.0129321 | 0.0019463 | 3.00E-11 | 0.009781785 | 10.49804469 |  |
| Getting up in morning | 17 | rs4790352 | A | G | 0.917939 | 461658 | 0.0178551 | 0.00291327 | 8.80E-10 | 0.009021279 | 9.674421852 |  |
| Getting up in morning | 17 | rs77556405 | A | G | 0.172495 | 461658 | 0.0162404 | 0.00211178 | 1.50E-14 | 0.011313573 | 12.1608078 |  |
| Getting up in morning | 17 | rs3760185 | T | C | 0.247523 | 461658 | -0.012079 | 0.00187134 | 1.10E-10 | 0.009496228 | 10.18863999 |  |
| Getting up in morning | 17 | rs12601968 | T | G | 0.32673 | 461658 | -0.0110555 | 0.00170497 | 8.90E-11 | 0.009543337 | 10.23967107 |  |
| Getting up in morning | 18 | rs627685 | C | T | 0.303582 | 461658 | 0.0104007 | 0.00174935 | 2.80E-09 | 0.008746283 | 9.3769137 |  |
| Getting up in morning | 18 | rs12326675 | G | C | 0.193346 | 461658 | 0.0119267 | 0.00217634 | 4.20E-08 | 0.008068332 | 8.644168712 |  |
| Getting up in morning | 18 | rs12969848 | T | C | 0.529476 | 461658 | 0.00952836 | 0.00160486 | 2.90E-09 | 0.008737817 | 9.367757627 |  |
| Getting up in morning | 19 | rs113232113 | T | A | 0.116606 | 461658 | -0.0139036 | 0.002488 | 2.30E-08 | 0.008223653 | 8.811954565 |  |
| Getting up in morning | 19 | rs11672103 | T | C | 0.553457 | 461658 | -0.00937007 | 0.0016161 | 6.70E-09 | 0.008533437 | 9.146756903 |  |
| Getting up in morning | 19 | rs11669535 | C | T | 0.21707 | 461658 | 0.0106355 | 0.00194612 | 4.60E-08 | 0.008044621 | 8.618559423 |  |
| Getting up in morning | 20 | rs74555583 | A | G | 0.085288 | 461658 | -0.0171376 | 0.00285841 | 2.00E-09 | 0.008827065 | 9.464291708 |  |
| Getting up in morning | 20 | rs6141724 | G | T | 0.370474 | 461658 | -0.00997727 | 0.00166947 | 2.30E-09 | 0.008793595 | 9.428087322 |  |
| Getting up in morning | 22 | rs133067 | T | C | 0.790791 | 461658 | 0.0111745 | 0.00197572 | 1.50E-08 | 0.008332246 | 8.929293488 |  |
| Chronotype | 1 | rs61773390 | T | G | 0.195349 | 413343 | -0.0256729 | 0.00256295 | 1.30E-23 | 0.015576721 | 15.0542023 | 54.33837194 |
| Chronotype | 1 | rs72720396 | G | A | 0.229643 | 413343 | -0.0206181 | 0.00241443 | 1.30E-17 | 0.013287816 | 12.81228738 |  |
| Chronotype | 1 | rs10737452 | T | C | 0.624486 | 413343 | 0.014352 | 0.00209801 | 7.90E-12 | 0.010638982 | 10.23078198 |  |
| Chronotype | 1 | rs56372114 | T | C | 0.384232 | 413343 | 0.0121296 | 0.0020884 | 6.30E-09 | 0.009034394 | 8.673691915 |  |
| Chronotype | 1 | rs7547493 | G | A | 0.177307 | 413343 | -0.0281137 | 0.00265737 | 3.70E-26 | 0.016453633 | 15.91587521 |  |
| Chronotype | 1 | rs509476 | C | T | 0.970151 | 413343 | 0.0878165 | 0.00598009 | 8.10E-49 | 0.022834543 | 22.23247407 |  |
| Chronotype | 1 | rs17448682 | T | C | 0.232109 | 413343 | -0.0164854 | 0.00241021 | 7.90E-12 | 0.010638982 | 10.23078198 |  |
| Chronotype | 1 | rs6658041 | A | G | 0.599474 | 413343 | -0.0120027 | 0.00207692 | 7.50E-09 | 0.008988879 | 8.629597842 |  |
| Chronotype | 1 | rs12117333 | A | G | 0.077242 | 413343 | 0.0240933 | 0.00380244 | 2.40E-10 | 0.009850393 | 9.464906306 |  |
| Chronotype | 1 | rs12140153 | T | G | 0.093978 | 413343 | 0.0269965 | 0.00357405 | 4.20E-14 | 0.011749808 | 11.31168808 |  |
| Chronotype | 1 | rs4949980 | G | A | 0.066883 | 413343 | -0.0229312 | 0.00410535 | 2.30E-08 | 0.008690966 | 8.341085165 |  |
| Chronotype | 1 | rs17575798 | A | G | 0.193371 | 413343 | 0.0160596 | 0.00256837 | 4.00E-10 | 0.009727137 | 9.345309754 |  |
| Chronotype | 1 | rs11587758 | A | G | 0.396163 | 413343 | -0.0187958 | 0.00207298 | 1.20E-19 | 0.014104793 | 13.6112972 |  |
| Chronotype | 1 | rs111761918 | A | G | 0.068055 | 413343 | 0.0220452 | 0.00403864 | 4.80E-08 | 0.008490017 | 8.146574661 |  |
| Chronotype | 2 | rs848552 | G | C | 0.527723 | 413343 | -0.0123111 | 0.0020344 | 1.40E-09 | 0.009418353 | 9.045826226 |  |
| Chronotype | 2 | rs7586062 | C | G | 0.471841 | 413343 | 0.0180322 | 0.00204753 | 1.30E-18 | 0.013695286 | 13.21063086 |  |
| Chronotype | 2 | rs4549082 | C | T | 0.484139 | 413343 | -0.0153086 | 0.00203567 | 5.50E-14 | 0.01169506 | 11.25835837 |  |
| Chronotype | 2 | rs13011556 | G | C | 0.239067 | 413343 | -0.0168735 | 0.00239296 | 1.80E-12 | 0.010963685 | 10.5464884 |  |
| Chronotype | 2 | rs116131939 | T | C | 0.083611 | 413343 | -0.0228637 | 0.00372053 | 8.00E-10 | 0.009557464 | 9.180724926 |  |
| Chronotype | 2 | rs4671379 | C | T | 0.58559 | 413343 | -0.0123818 | 0.00206477 | 2.00E-09 | 0.009328662 | 8.958871689 |  |
| Chronotype | 2 | rs6718119 | G | A | 0.376746 | 413343 | -0.0119862 | 0.00209794 | 1.10E-08 | 0.008888125 | 8.532003449 |  |
| Chronotype | 2 | rs138964083 | T | C | 0.058599 | 413343 | -0.0256922 | 0.0043341 | 3.10E-09 | 0.009217319 | 8.850947849 |  |
| Chronotype | 2 | rs57435966 | T | C | 0.08629 | 413343 | 0.0410747 | 0.00362263 | 8.50E-30 | 0.017632644 | 17.07682362 |  |
| Chronotype | 2 | rs12713014 | G | A | 0.058725 | 413343 | 0.0262121 | 0.00436185 | 1.90E-09 | 0.009341614 | 8.971427344 |  |
| Chronotype | 2 | rs10175975 | T | C | 0.181726 | 413343 | -0.0183756 | 0.00265358 | 4.40E-12 | 0.010768564 | 10.35674898 |  |
| Chronotype | 2 | rs28380327 | T | A | 0.369986 | 413343 | 0.0154008 | 0.00210437 | 2.50E-13 | 0.011383205 | 10.95469079 |  |
| Chronotype | 2 | rs197273 | G | A | 0.529834 | 413343 | 0.0120956 | 0.00204052 | 3.10E-09 | 0.009217319 | 8.850947849 |  |
| Chronotype | 2 | rs114870822 | A | G | 0.012965 | 413343 | -0.0493504 | 0.00902627 | 4.60E-08 | 0.008501762 | 8.157941149 |  |
| Chronotype | 2 | rs62182115 | T | C | 0.341565 | 413343 | 0.0132852 | 0.00214181 | 5.50E-10 | 0.009649539 | 9.270031448 |  |
| Chronotype | 2 | rs2712056 | T | C | 0.185573 | 413343 | -0.0157302 | 0.00261566 | 1.80E-09 | 0.009355243 | 8.984640533 |  |
| Chronotype | 2 | rs2850298 | G | A | 0.698902 | 413343 | -0.0186925 | 0.00221403 | 3.10E-17 | 0.013130791 | 12.65886784 |  |
| Chronotype | 2 | rs812925 | G | C | 0.351718 | 413343 | -0.0151176 | 0.00212493 | 1.10E-12 | 0.01106978 | 10.64968854 |  |
| Chronotype | 2 | rs62198772 | A | T | 0.410702 | 413343 | 0.0118177 | 0.00207674 | 1.30E-08 | 0.008843836 | 8.489110131 |  |
| Chronotype | 2 | rs2706762 | T | C | 0.149575 | 413343 | 0.0180336 | 0.00284507 | 2.30E-10 | 0.009860594 | 9.474805333 |  |
| Chronotype | 3 | rs17786957 | C | G | 0.16512 | 413343 | 0.0163112 | 0.00274705 | 2.90E-09 | 0.009234343 | 8.867447826 |  |
| Chronotype | 3 | rs114848860 | T | A | 0.024346 | 413343 | -0.041456 | 0.00662279 | 3.90E-10 | 0.009733283 | 9.351272987 |  |
| Chronotype | 3 | rs112555644 | T | C | 0.067556 | 413343 | -0.0285588 | 0.00421866 | 1.30E-11 | 0.010527468 | 10.1224061 |  |
| Chronotype | 3 | rs7652260 | G | C | 0.161797 | 413343 | 0.016002 | 0.00277482 | 8.10E-09 | 0.008968715 | 8.610064555 |  |
| Chronotype | 3 | rs13316611 | T | G | 0.255868 | 413343 | -0.0135195 | 0.00233302 | 6.80E-09 | 0.009014482 | 8.654401361 |  |
| Chronotype | 3 | rs11714441 | T | C | 0.400637 | 413343 | 0.0117967 | 0.00207554 | 1.30E-08 | 0.008843836 | 8.489110131 |  |
| Chronotype | 3 | rs7626349 | C | T | 0.705763 | 413343 | -0.0125384 | 0.00223179 | 1.90E-08 | 0.008742441 | 8.390923683 |  |
| Chronotype | 3 | rs34244172 | T | C | 0.292264 | 413343 | 0.0124769 | 0.00224924 | 2.90E-08 | 0.008628129 | 8.280252647 |  |
| Chronotype | 3 | rs2239626 | C | T | 0.305676 | 413343 | -0.0139103 | 0.0022141 | 3.30E-10 | 0.00977373 | 9.390516015 |  |
| Chronotype | 3 | rs6441169 | A | G | 0.856023 | 413343 | -0.016178 | 0.00289542 | 2.30E-08 | 0.008690966 | 8.341085165 |  |
| Chronotype | 3 | rs9831488 | G | A | 0.353395 | 413343 | -0.0138691 | 0.0021483 | 1.10E-10 | 0.010035842 | 9.644904484 |  |
| Chronotype | 3 | rs6442446 | G | A | 0.708837 | 413343 | 0.0130204 | 0.00225053 | 7.20E-09 | 0.008999556 | 8.639941135 |  |
| Chronotype | 3 | rs66710942 | T | C | 0.592333 | 413343 | -0.0133068 | 0.0020796 | 1.60E-10 | 0.009947186 | 9.558845891 |  |
| Chronotype | 3 | rs4484214 | G | A | 0.314742 | 413343 | 0.0136358 | 0.00219015 | 4.80E-10 | 0.009682782 | 9.302280085 |  |
| Chronotype | 3 | rs1800828 | G | C | 0.253098 | 413343 | 0.0131149 | 0.00233756 | 2.00E-08 | 0.008728648 | 8.377568419 |  |
| Chronotype | 3 | rs149611468 | C | T | 0.011957 | 413343 | 0.0585049 | 0.00954645 | 8.90E-10 | 0.009531112 | 9.155168109 |  |
| Chronotype | 3 | rs13059636 | G | A | 0.469784 | 413343 | -0.0133959 | 0.00204675 | 6.00E-11 | 0.010177662 | 9.782601314 |  |
| Chronotype | 4 | rs1135946 | C | T | 0.232423 | 413343 | 0.0172702 | 0.00240488 | 6.90E-13 | 0.011169375 | 10.74658588 |  |
| Chronotype | 4 | rs4241964 | G | T | 0.475791 | 413343 | -0.015226 | 0.00204725 | 1.00E-13 | 0.011572902 | 11.13938438 |  |
| Chronotype | 5 | rs10058356 | T | C | 0.697815 | 413343 | 0.013099 | 0.00222077 | 3.70E-09 | 0.009172004 | 8.807031052 |  |
| Chronotype | 5 | rs147762489 | T | C | 0.249106 | 413343 | 0.0162874 | 0.00236044 | 5.20E-12 | 0.010731745 | 10.32095388 |  |
| Chronotype | 5 | rs10461917 | C | T | 0.689585 | 413343 | 0.0123205 | 0.00220996 | 2.50E-08 | 0.008668413 | 8.319251158 |  |
| Chronotype | 5 | rs4518438 | C | T | 0.510399 | 413343 | -0.015264 | 0.0020348 | 6.30E-14 | 0.011667453 | 11.2314678 |  |
| Chronotype | 5 | rs286808 | C | T | 0.524555 | 413343 | 0.0117126 | 0.00204007 | 9.40E-09 | 0.008929603 | 8.572178401 |  |
| Chronotype | 5 | rs9291813 | C | T | 0.760663 | 413343 | -0.0133858 | 0.00238253 | 1.90E-08 | 0.008742441 | 8.390923683 |  |
| Chronotype | 5 | rs67988891 | G | C | 0.318639 | 413343 | -0.0187173 | 0.00218678 | 1.10E-17 | 0.013317766 | 12.84155549 |  |
| Chronotype | 5 | rs7735794 | A | G | 0.223944 | 413343 | -0.0151744 | 0.0025788 | 4.00E-09 | 0.009151968 | 8.787614977 |  |
| Chronotype | 6 | rs610590 | G | C | 0.211955 | 413343 | -0.0146358 | 0.00249762 | 4.60E-09 | 0.009115946 | 8.752708403 |  |
| Chronotype | 6 | rs76223855 | C | T | 0.010981 | 413343 | -0.0798575 | 0.00975491 | 2.70E-16 | 0.0127316 | 12.26906048 |  |
| Chronotype | 6 | rs12525312 | C | T | 0.550602 | 413343 | 0.0122911 | 0.00204528 | 1.90E-09 | 0.009341614 | 8.971427344 |  |
| Chronotype | 6 | rs9395520 | T | C | 0.304164 | 413343 | -0.0173714 | 0.00221234 | 4.10E-15 | 0.012211926 | 11.76207555 |  |
| Chronotype | 6 | rs35101255 | G | A | 0.079468 | 413343 | 0.0244145 | 0.00376113 | 8.50E-11 | 0.010096421 | 9.703717027 |  |
| Chronotype | 6 | rs9348050 | C | T | 0.510601 | 413343 | 0.0124568 | 0.0020362 | 9.50E-10 | 0.009514954 | 9.139497876 |  |
| Chronotype | 6 | rs486416 | A | G | 0.636771 | 413343 | 0.0115784 | 0.00210256 | 3.70E-08 | 0.008561619 | 8.215873824 |  |
| Chronotype | 6 | rs1983891 | T | C | 0.276348 | 413343 | 0.0129088 | 0.00224143 | 8.50E-09 | 0.008956068 | 8.597814106 |  |
| Chronotype | 6 | rs2653349 | G | A | 0.787049 | 413343 | 0.0283958 | 0.0024837 | 2.90E-30 | 0.017778377 | 17.22051662 |  |
| Chronotype | 6 | rs9476310 | T | C | 0.51056 | 413343 | -0.0126008 | 0.00203934 | 6.50E-10 | 0.009608593 | 9.230314737 |  |
| Chronotype | 6 | rs2881955 | T | C | 0.278459 | 413343 | -0.0136327 | 0.00227345 | 2.00E-09 | 0.009328662 | 8.958871689 |  |
| Chronotype | 7 | rs1996399 | A | G | 0.300648 | 413343 | -0.0121719 | 0.00222228 | 4.30E-08 | 0.008520345 | 8.17592578 |  |
| Chronotype | 7 | rs202157 | T | C | 0.701066 | 413343 | 0.0189794 | 0.00222883 | 1.70E-17 | 0.01323955 | 12.76512449 |  |
| Chronotype | 7 | rs2971970 | G | T | 0.782046 | 413343 | 0.0151566 | 0.00246689 | 8.00E-10 | 0.009557464 | 9.180724926 |  |
| Chronotype | 7 | rs17161045 | C | T | 0.369932 | 413343 | 0.0153426 | 0.0021211 | 4.70E-13 | 0.011250683 | 10.82570686 |  |
| Chronotype | 7 | rs4729854 | A | T | 0.482 | 413343 | 0.0216699 | 0.00208203 | 2.30E-25 | 0.01618531 | 15.65205192 |  |
| Chronotype | 7 | rs7783012 | A | G | 0.590654 | 413343 | -0.0121216 | 0.00207487 | 5.20E-09 | 0.009084239 | 8.721985496 |  |
| Chronotype | 7 | rs10280205 | C | T | 0.309279 | 413343 | 0.0133149 | 0.00220293 | 1.50E-09 | 0.009401068 | 9.029068081 |  |
| Chronotype | 7 | rs6967481 | T | C | 0.497073 | 413343 | -0.0155247 | 0.0020431 | 3.00E-14 | 0.011817711 | 11.37784136 |  |
| Chronotype | 8 | rs10954933 | G | A | 0.42758 | 413343 | -0.0154825 | 0.00206223 | 6.00E-14 | 0.01167735 | 11.24110787 |  |
| Chronotype | 8 | rs769066 | C | T | 0.18416 | 413343 | -0.0165403 | 0.00263064 | 3.20E-10 | 0.009781161 | 9.397726014 |  |
| Chronotype | 8 | rs3100052 | G | A | 0.613151 | 413343 | 0.0122033 | 0.00208964 | 5.20E-09 | 0.009084239 | 8.721985496 |  |
| Chronotype | 8 | rs13258797 | A | G | 0.168546 | 413343 | -0.0158848 | 0.00271665 | 5.00E-09 | 0.009094392 | 8.731823692 |  |
| Chronotype | 8 | rs17716502 | T | C | 0.204456 | 413343 | -0.018837 | 0.00254156 | 1.20E-13 | 0.011535384 | 11.10285085 |  |
| Chronotype | 8 | rs4321976 | C | T | 0.221209 | 413343 | 0.0172055 | 0.00245224 | 2.30E-12 | 0.010910539 | 10.49480126 |  |
| Chronotype | 8 | rs35524253 | A | G | 0.356248 | 413343 | -0.012109 | 0.00212995 | 1.30E-08 | 0.008843836 | 8.489110131 |  |
| Chronotype | 8 | rs6601686 | T | A | 0.410116 | 413343 | 0.0137112 | 0.00208275 | 4.60E-11 | 0.010239252 | 9.842412498 |  |
| Chronotype | 9 | rs12377175 | C | A | 0.22858 | 413343 | 0.0155356 | 0.00236169 | 4.80E-11 | 0.010229456 | 9.832898832 |  |
| Chronotype | 9 | rs10988239 | T | C | 0.512284 | 413343 | 0.012885 | 0.00206881 | 4.70E-10 | 0.009687913 | 9.30725686 |  |
| Chronotype | 9 | rs62553781 | T | C | 0.034554 | 413343 | 0.0379349 | 0.00558132 | 1.10E-11 | 0.010564971 | 10.1588513 |  |
| Chronotype | 9 | rs113171806 | C | T | 0.11444 | 413343 | 0.0181433 | 0.00319962 | 1.40E-08 | 0.008824118 | 8.470014281 |  |
| Chronotype | 9 | rs28458909 | T | C | 0.122383 | 413343 | 0.0298802 | 0.00309799 | 5.20E-22 | 0.014999076 | 14.48743236 |  |
| Chronotype | 9 | rs2291589 | G | T | 0.377049 | 413343 | 0.0151909 | 0.00209801 | 4.50E-13 | 0.011259866 | 10.83464325 |  |
| Chronotype | 9 | rs10118767 | T | C | 0.197744 | 413343 | 0.0139673 | 0.0025533 | 4.50E-08 | 0.008507825 | 8.163809243 |  |
| Chronotype | 9 | rs57994353 | C | T | 0.299321 | 413343 | -0.0128023 | 0.00221782 | 7.80E-09 | 0.008978612 | 8.619652136 |  |
| Chronotype | 10 | rs2893787 | A | G | 0.743579 | 413343 | 0.0148125 | 0.00232708 | 1.90E-10 | 0.009906275 | 9.519137973 |  |
| Chronotype | 10 | rs12249410 | T | G | 0.109634 | 413343 | 0.018994 | 0.00331917 | 1.00E-08 | 0.008913297 | 8.556384349 |  |
| Chronotype | 10 | rs3808964 | T | G | 0.633453 | 413343 | -0.0121189 | 0.00211446 | 1.00E-08 | 0.008913297 | 8.556384349 |  |
| Chronotype | 11 | rs1914772 | A | T | 0.894653 | 413343 | 0.0211605 | 0.00336816 | 3.30E-10 | 0.00977373 | 9.390516015 |  |
| Chronotype | 11 | rs4936291 | G | A | 0.389142 | 413343 | -0.0136292 | 0.00214398 | 2.10E-10 | 0.009882369 | 9.495937801 |  |
| Chronotype | 11 | rs74357745 | G | A | 0.12053 | 413343 | 0.0202381 | 0.00312645 | 9.60E-11 | 0.010067849 | 9.675977048 |  |
| Chronotype | 11 | rs9795439 | G | A | 0.803776 | 413343 | 0.0147756 | 0.0025608 | 7.90E-09 | 0.00897527 | 8.616414624 |  |
| Chronotype | 11 | rs4141920 | A | G | 0.455163 | 413343 | 0.0116524 | 0.00204526 | 1.20E-08 | 0.008865081 | 8.509684826 |  |
| Chronotype | 11 | rs2077432 | T | C | 0.269742 | 413343 | -0.013398 | 0.00229973 | 5.70E-09 | 0.009060425 | 8.698912399 |  |
| Chronotype | 11 | rs3729986 | T | C | 0.10224 | 413343 | -0.018897 | 0.00334958 | 1.70E-08 | 0.008772271 | 8.419807987 |  |
| Chronotype | 11 | rs4237555 | T | C | 0.527732 | 413343 | -0.0117019 | 0.00203815 | 9.40E-09 | 0.008929603 | 8.572178401 |  |
| Chronotype | 11 | rs72632979 | G | A | 0.172073 | 413343 | 0.0165475 | 0.0027028 | 9.20E-10 | 0.009522902 | 9.147205992 |  |
| Chronotype | 11 | rs10742179 | G | A | 0.739373 | 413343 | 0.0129008 | 0.00231806 | 2.60E-08 | 0.008657787 | 8.308964217 |  |
| Chronotype | 11 | rs11032362 | A | G | 0.090981 | 413343 | -0.0262159 | 0.00353388 | 1.20E-13 | 0.011535384 | 11.10285085 |  |
| Chronotype | 11 | rs3168135 | A | G | 0.24042 | 413343 | 0.0149704 | 0.00237964 | 3.20E-10 | 0.009781161 | 9.397726014 |  |
| Chronotype | 12 | rs12811046 | G | A | 0.445344 | 413343 | 0.0129567 | 0.00204647 | 2.40E-10 | 0.009850393 | 9.464906306 |  |
| Chronotype | 12 | rs4595586 | T | A | 0.506544 | 413343 | 0.0227532 | 0.00203888 | 6.40E-29 | 0.017355771 | 16.80394202 |  |
| Chronotype | 12 | rs7304278 | G | A | 0.723741 | 413343 | -0.0147233 | 0.00228303 | 1.10E-10 | 0.010035842 | 9.644904484 |  |
| Chronotype | 12 | rs80097534 | T | G | 0.098204 | 413343 | 0.0223132 | 0.00345166 | 1.00E-10 | 0.010058272 | 9.666679067 |  |
| Chronotype | 12 | rs11183201 | C | T | 0.508456 | 413343 | -0.0129151 | 0.00204003 | 2.40E-10 | 0.009850393 | 9.464906306 |  |
| Chronotype | 12 | rs74097630 | T | G | 0.140832 | 413343 | 0.0182454 | 0.0029315 | 4.80E-10 | 0.009682782 | 9.302280085 |  |
| Chronotype | 12 | rs7959983 | C | T | 0.405084 | 413343 | -0.0140795 | 0.00206412 | 9.00E-12 | 0.01060991 | 10.20252633 |  |
| Chronotype | 13 | rs9573971 | G | A | 0.03387 | 413343 | 0.0525677 | 0.0056289 | 9.70E-21 | 0.014524881 | 14.02266261 |  |
| Chronotype | 13 | rs2762088 | G | T | 0.761336 | 413343 | -0.0144671 | 0.0023956 | 1.60E-09 | 0.009384871 | 9.013364193 |  |
| Chronotype | 13 | rs17517 | A | G | 0.511747 | 413343 | 0.0116417 | 0.00204935 | 1.30E-08 | 0.008843836 | 8.489110131 |  |
| Chronotype | 13 | rs9597250 | A | C | 0.189491 | 413343 | 0.0160838 | 0.00260302 | 6.50E-10 | 0.009608593 | 9.230314737 |  |
| Chronotype | 13 | rs1927719 | A | T | 0.765682 | 413343 | -0.0139841 | 0.002412 | 6.70E-09 | 0.009018351 | 8.658149666 |  |
| Chronotype | 14 | rs12432176 | A | C | 0.379609 | 413343 | -0.0120801 | 0.00210587 | 9.70E-09 | 0.008921329 | 8.564164309 |  |
| Chronotype | 14 | rs10149448 | G | A | 0.396431 | 413343 | 0.0115459 | 0.00208314 | 3.00E-08 | 0.008618904 | 8.271323054 |  |
| Chronotype | 14 | rs698015 | T | C | 0.646935 | 413343 | -0.0131346 | 0.00216074 | 1.20E-09 | 0.009456868 | 9.083171234 |  |
| Chronotype | 14 | rs7148842 | T | C | 0.386473 | 413343 | 0.0121827 | 0.00210712 | 7.40E-09 | 0.008992391 | 8.633000277 |  |
| Chronotype | 15 | rs1439319 | C | G | 0.645445 | 413343 | 0.0121652 | 0.00214366 | 1.40E-08 | 0.008824118 | 8.470014281 |  |
| Chronotype | 15 | rs2701524 | C | T | 0.413565 | 413343 | 0.0114927 | 0.0020789 | 3.20E-08 | 0.008601313 | 8.254294944 |  |
| Chronotype | 15 | rs4886947 | A | G | 0.660074 | 413343 | 0.0119315 | 0.00216342 | 3.50E-08 | 0.008576831 | 8.230597384 |  |
| Chronotype | 16 | rs4784655 | C | G | 0.321967 | 413343 | 0.0163395 | 0.00218439 | 7.40E-14 | 0.011634607 | 11.19947702 |  |
| Chronotype | 16 | rs12927162 | G | A | 0.276664 | 413343 | 0.0209482 | 0.00227398 | 3.20E-20 | 0.014327069 | 13.82891405 |  |
| Chronotype | 16 | rs1421085 | C | T | 0.403565 | 413343 | -0.0208469 | 0.00207358 | 8.90E-24 | 0.015634847 | 15.11127006 |  |
| Chronotype | 16 | rs9932577 | A | C | 0.505674 | 413343 | 0.0126513 | 0.00205454 | 7.40E-10 | 0.009576692 | 9.199373494 |  |
| Chronotype | 16 | rs17604349 | A | G | 0.179748 | 413343 | 0.0219477 | 0.00265632 | 1.40E-16 | 0.012854034 | 12.38858333 |  |
| Chronotype | 17 | rs3760185 | T | C | 0.247661 | 413343 | 0.0189059 | 0.00238368 | 2.20E-15 | 0.012332746 | 11.87989795 |  |
| Chronotype | 17 | rs225298 | G | T | 0.830934 | 413343 | 0.0151765 | 0.00271222 | 2.20E-08 | 0.00870297 | 8.352706634 |  |
| Chronotype | 17 | rs6504758 | G | A | 0.535685 | 413343 | -0.0124697 | 0.00205007 | 1.20E-09 | 0.009456868 | 9.083171234 |  |
| Chronotype | 17 | rs2364972 | G | A | 0.462953 | 413343 | -0.0128022 | 0.00204355 | 3.70E-10 | 0.009746045 | 9.363654385 |  |
| Chronotype | 17 | rs72829706 | G | A | 0.039463 | 413343 | 0.0295339 | 0.00524217 | 1.80E-08 | 0.008756954 | 8.404975896 |  |
| Chronotype | 17 | rs2518022 | C | T | 0.914438 | 413343 | 0.0307024 | 0.0036278 | 2.60E-17 | 0.013162718 | 12.69005734 |  |
| Chronotype | 18 | rs12965577 | G | A | 0.334996 | 413343 | 0.015874 | 0.00215922 | 2.00E-13 | 0.011429682 | 10.99993552 |  |
| Chronotype | 18 | rs12969848 | T | C | 0.529314 | 413343 | -0.0164219 | 0.00204453 | 9.60E-16 | 0.012491885 | 12.03513201 |  |
| Chronotype | 18 | rs62082401 | G | C | 0.191147 | 413343 | -0.0200454 | 0.00258841 | 9.60E-15 | 0.012044925 | 11.59926485 |  |
| Chronotype | 18 | rs9962650 | G | C | 0.422583 | 413343 | -0.0139169 | 0.00206328 | 1.50E-11 | 0.01049519 | 10.09104082 |  |
| Chronotype | 18 | rs9964420 | A | C | 0.302896 | 413343 | 0.0214879 | 0.0022239 | 4.40E-22 | 0.015025686 | 14.51352749 |  |
| Chronotype | 18 | rs56076457 | T | C | 0.529246 | 413343 | -0.0117206 | 0.00203837 | 8.90E-09 | 0.008943986 | 8.586110566 |  |
| Chronotype | 19 | rs10402849 | T | C | 0.201704 | 413343 | -0.0151712 | 0.00254031 | 2.30E-09 | 0.00929329 | 8.924583744 |  |
| Chronotype | 19 | rs12971913 | A | G | 0.448148 | 413343 | 0.012664 | 0.0020508 | 6.60E-10 | 0.009604843 | 9.226677326 |  |
| Chronotype | 19 | rs12462111 | T | C | 0.464858 | 413343 | 0.0131658 | 0.0020669 | 1.90E-10 | 0.009906275 | 9.519137973 |  |
| Chronotype | 19 | rs1874493 | G | A | 0.680061 | 413343 | 0.0123004 | 0.00218337 | 1.80E-08 | 0.008756954 | 8.404975896 |  |
| Chronotype | 20 | rs78095690 | C | T | 0.437331 | 413343 | -0.0114876 | 0.00205775 | 2.40E-08 | 0.008679463 | 8.329948878 |  |
| Chronotype | 20 | rs2072727 | C | T | 0.564423 | 413343 | 0.0116552 | 0.00205334 | 1.40E-08 | 0.008824118 | 8.470014281 |  |
| Chronotype | 20 | rs6131942 | G | A | 0.579505 | 413343 | -0.0143086 | 0.00206747 | 4.50E-12 | 0.010763645 | 10.35196656 |  |
| Chronotype | 22 | rs1056322 | G | C | 0.321001 | 413343 | 0.0126409 | 0.00217877 | 6.60E-09 | 0.009022273 | 8.661949033 |  |
| Chronotype | 22 | rs139911 | T | C | 0.575785 | 413343 | 0.0175504 | 0.00206661 | 2.00E-17 | 0.013210217 | 12.73646431 |  |
| Nap during day | 1 | rs6696864 | A | T | 0.401498 | 462400 | -0.00704241 | 0.00125496 | 2.00E-08 | 0.00825268 | 8.857543697 | 50.70852245 |
| Nap during day | 1 | rs2786547 | T | C | 0.177277 | 462400 | -0.0109884 | 0.00160158 | 6.80E-12 | 0.010090389 | 10.85004963 |  |
| Nap during day | 1 | rs12042846 | C | T | 0.178571 | 462400 | 0.00905398 | 0.00160707 | 1.80E-08 | 0.008279442 | 8.886507711 |  |
| Nap during day | 1 | rs7555990 | T | C | 0.13587 | 462400 | -0.010372 | 0.00178981 | 6.80E-09 | 0.00852293 | 9.150094576 |  |
| Nap during day | 1 | rs11121194 | T | C | 0.633703 | 462400 | 0.00706318 | 0.00126583 | 2.40E-08 | 0.008206177 | 8.807219517 |  |
| Nap during day | 1 | rs903678 | A | G | 0.336999 | 462400 | 0.013653 | 0.00129298 | 4.60E-26 | 0.015526555 | 16.78767026 |  |
| Nap during day | 1 | rs12140153 | T | G | 0.09424 | 462400 | -0.0242712 | 0.0021468 | 1.20E-29 | 0.016626916 | 17.99752306 |  |
| Nap during day | 1 | rs1931175 | G | C | 0.38274 | 462400 | 0.00746118 | 0.00126008 | 3.20E-09 | 0.008707034 | 9.349482252 |  |
| Nap during day | 2 | rs75022160 | T | C | 0.1366 | 462400 | -0.00976296 | 0.00179002 | 4.90E-08 | 0.008021671 | 8.607598669 |  |
| Nap during day | 2 | rs11688767 | T | A | 0.486928 | 462400 | 0.00731846 | 0.00122558 | 2.40E-09 | 0.008776332 | 9.424552048 |  |
| Nap during day | 2 | rs2390669 | C | A | 0.128947 | 462400 | -0.0112781 | 0.00183747 | 8.40E-10 | 0.009024914 | 9.693925699 |  |
| Nap during day | 2 | rs13023587 | G | C | 0.508078 | 462400 | -0.00792715 | 0.0012238 | 9.30E-11 | 0.009525922 | 10.23724869 |  |
| Nap during day | 2 | rs12615434 | T | C | 0.11309 | 462400 | 0.0116344 | 0.00193313 | 1.80E-09 | 0.008845113 | 9.499072146 |  |
| Nap during day | 2 | rs13033444 | G | A | 0.281934 | 462400 | 0.0094407 | 0.00136451 | 4.60E-12 | 0.010172126 | 10.93884308 |  |
| Nap during day | 2 | rs7422655 | T | C | 0.736305 | 462400 | -0.00805295 | 0.00139094 | 7.10E-09 | 0.008512271 | 9.138552826 |  |
| Nap during day | 2 | rs908442 | T | A | 0.408474 | 462400 | -0.00977066 | 0.0012473 | 4.70E-15 | 0.011520863 | 12.4061446 |  |
| Nap during day | 2 | rs11125776 | G | T | 0.144182 | 462400 | -0.0120412 | 0.00174757 | 5.60E-12 | 0.010131072 | 10.89424315 |  |
| Nap during day | 2 | rs12992648 | G | A | 0.281533 | 462400 | -0.00762551 | 0.00136135 | 2.10E-08 | 0.008240259 | 8.84410222 |  |
| Nap during day | 3 | rs75411336 | T | C | 0.054402 | 462400 | -0.015756 | 0.0027004 | 5.40E-09 | 0.008579635 | 9.211498706 |  |
| Nap during day | 3 | rs40005 | A | G | 0.76889 | 462400 | 0.00807247 | 0.00145429 | 2.80E-08 | 0.008166661 | 8.764460516 |  |
| Nap during day | 3 | rs1001817 | T | C | 0.494347 | 462400 | -0.00776666 | 0.00122507 | 2.30E-10 | 0.009322912 | 10.01702615 |  |
| Nap during day | 3 | rs77154532 | G | A | 0.359504 | 462400 | -0.00724236 | 0.00128118 | 1.60E-08 | 0.008309261 | 8.918780929 |  |
| Nap during day | 3 | rs4856536 | A | G | 0.728768 | 462400 | -0.00859681 | 0.00138106 | 4.80E-10 | 0.009154795 | 9.834723048 |  |
| Nap during day | 4 | rs9998136 | G | C | 0.748397 | 462400 | 0.00948143 | 0.00141666 | 2.20E-11 | 0.00984087 | 10.57907821 |  |
| Nap during day | 4 | rs113886333 | T | C | 0.036115 | 462400 | 0.0182674 | 0.00329762 | 3.00E-08 | 0.00814892 | 8.745263564 |  |
| Nap during day | 4 | rs7698842 | G | A | 0.741815 | 462400 | 0.00777807 | 0.00141692 | 4.00E-08 | 0.008074536 | 8.664787001 |  |
| Nap during day | 4 | rs4692709 | T | C | 0.546706 | 462400 | -0.00700067 | 0.00123617 | 1.50E-08 | 0.008325558 | 8.936420139 |  |
| Nap during day | 5 | rs12657723 | T | C | 0.320683 | 462400 | 0.0081807 | 0.00131152 | 4.40E-10 | 0.009174832 | 9.856448284 |  |
| Nap during day | 5 | rs467897 | A | G | 0.68009 | 462400 | -0.00930896 | 0.00131516 | 1.50E-12 | 0.010403116 | 11.18985557 |  |
| Nap during day | 5 | rs2431108 | C | T | 0.328424 | 462400 | 0.012511 | 0.00130232 | 7.50E-22 | 0.014125928 | 15.25158218 |  |
| Nap during day | 5 | rs10875622 | A | G | 0.575874 | 462400 | 0.0102674 | 0.00124068 | 1.30E-16 | 0.012166167 | 13.10959304 |  |
| Nap during day | 5 | rs6452787 | G | A | 0.466869 | 462400 | -0.0072452 | 0.00122693 | 3.50E-09 | 0.00868534 | 9.325982992 |  |
| Nap during day | 5 | rs2099810 | G | A | 0.496352 | 462400 | -0.00780818 | 0.0012254 | 1.90E-10 | 0.009366102 | 10.06387077 |  |
| Nap during day | 5 | rs72781017 | G | A | 0.402575 | 462400 | -0.00756808 | 0.00124775 | 1.30E-09 | 0.008922308 | 9.582721744 |  |
| Nap during day | 6 | rs2653349 | G | A | 0.787137 | 462400 | -0.0163076 | 0.0014949 | 1.00E-27 | 0.016046369 | 17.35887195 |  |
| Nap during day | 6 | rs7752899 | T | C | 0.442806 | 462400 | 0.00847983 | 0.00123126 | 5.70E-12 | 0.010127363 | 10.89021327 |  |
| Nap during day | 6 | rs62425620 | T | C | 0.369978 | 462400 | 0.00775191 | 0.00127613 | 1.20E-09 | 0.008941197 | 9.603191177 |  |
| Nap during day | 6 | rs34262487 | A | C | 0.072621 | 462400 | -0.0146376 | 0.00236551 | 6.10E-10 | 0.009099386 | 9.774652204 |  |
| Nap during day | 6 | rs6919087 | G | T | 0.311989 | 462400 | -0.0103817 | 0.00132307 | 4.30E-15 | 0.011537272 | 12.42402058 |  |
| Nap during day | 6 | rs2143792 | A | G | 0.431158 | 462400 | -0.00721564 | 0.00124498 | 6.80E-09 | 0.00852293 | 9.150094576 |  |
| Nap during day | 6 | rs1856502 | A | T | 0.456906 | 462400 | 0.00710396 | 0.00122766 | 7.20E-09 | 0.008508817 | 9.134813554 |  |
| Nap during day | 6 | rs785145 | G | T | 0.431525 | 462400 | 0.00706492 | 0.00123603 | 1.10E-08 | 0.008403462 | 9.020748307 |  |
| Nap during day | 6 | rs614987 | C | A | 0.613566 | 462400 | 0.0107556 | 0.00125938 | 1.30E-17 | 0.012563306 | 13.54297303 |  |
| Nap during day | 6 | rs9467772 | T | A | 0.199731 | 462400 | -0.00911826 | 0.00152838 | 2.40E-09 | 0.008776332 | 9.424552048 |  |
| Nap during day | 7 | rs35851551 | G | A | 0.101129 | 462400 | -0.0119501 | 0.00204914 | 5.50E-09 | 0.008575133 | 9.206624182 |  |
| Nap during day | 7 | rs1011024 | G | A | 0.154712 | 462400 | -0.0102274 | 0.00169762 | 1.70E-09 | 0.008858717 | 9.513812421 |  |
| Nap during day | 7 | rs6942927 | A | G | 0.12313 | 462400 | 0.0146516 | 0.00217116 | 1.50E-11 | 0.009922911 | 10.66815814 |  |
| Nap during day | 8 | rs7814873 | T | C | 0.616133 | 462400 | -0.00704488 | 0.00127461 | 3.30E-08 | 0.008124349 | 8.718678949 |  |
| Nap during day | 8 | rs351776 | C | A | 0.548043 | 462400 | 0.00762604 | 0.00123034 | 5.70E-10 | 0.0091151 | 9.791688199 |  |
| Nap during day | 8 | rs13266972 | G | A | 0.698405 | 462400 | -0.00729523 | 0.00133527 | 4.70E-08 | 0.008032554 | 8.619371024 |  |
| Nap during day | 8 | rs285815 | A | T | 0.546163 | 462400 | -0.0073837 | 0.00123101 | 2.00E-09 | 0.00881998 | 9.471841591 |  |
| Nap during day | 9 | rs10757347 | G | A | 0.222815 | 462400 | 0.00810359 | 0.00147647 | 4.10E-08 | 0.008068124 | 8.657850243 |  |
| Nap during day | 9 | rs971415 | G | A | 0.122716 | 462400 | -0.0108343 | 0.00186482 | 6.30E-09 | 0.008541756 | 9.170480066 |  |
| Nap during day | 9 | rs12346996 | C | T | 0.728608 | 462400 | -0.00832202 | 0.00137794 | 1.50E-09 | 0.008888439 | 9.546019616 |  |
| Nap during day | 9 | rs10868046 | A | G | 0.603704 | 462400 | 0.00727374 | 0.00126066 | 7.90E-09 | 0.008485856 | 9.109951574 |  |
| Nap during day | 9 | rs7038206 | G | A | 0.608433 | 462400 | 0.00738228 | 0.00125472 | 4.00E-09 | 0.00865292 | 9.290868532 |  |
| Nap during day | 9 | rs62560863 | T | C | 0.100702 | 462400 | 0.0114884 | 0.00203914 | 1.80E-08 | 0.008279442 | 8.886507711 |  |
| Nap during day | 9 | rs13284688 | C | T | 0.207055 | 462400 | 0.0144228 | 0.00151104 | 1.40E-21 | 0.014031125 | 15.14776733 |  |
| Nap during day | 10 | rs11252681 | A | G | 0.045481 | 462400 | 0.016111 | 0.00293663 | 4.10E-08 | 0.008068124 | 8.657850243 |  |
| Nap during day | 10 | rs224111 | A | G | 0.388797 | 462400 | -0.00784245 | 0.0012587 | 4.60E-10 | 0.009164599 | 9.845352819 |  |
| Nap during day | 10 | rs11258652 | A | C | 0.235891 | 462400 | -0.0100132 | 0.0014441 | 4.10E-12 | 0.010196089 | 10.96487744 |  |
| Nap during day | 10 | rs10764260 | A | G | 0.363097 | 462400 | -0.00733254 | 0.00128128 | 1.00E-08 | 0.008427261 | 9.046513417 |  |
| Nap during day | 11 | rs4587762 | A | G | 0.604757 | 462400 | -0.00694078 | 0.00125608 | 3.30E-08 | 0.008124349 | 8.718678949 |  |
| Nap during day | 11 | rs10835420 | A | T | 0.248826 | 462400 | -0.00857976 | 0.00141521 | 1.30E-09 | 0.008922308 | 9.582721744 |  |
| Nap during day | 11 | rs174541 | C | T | 0.35911 | 462400 | 0.0101856 | 0.00127505 | 1.40E-15 | 0.01174255 | 12.64770314 |  |
| Nap during day | 11 | rs6483215 | G | A | 0.764113 | 462400 | -0.00841794 | 0.00144207 | 5.30E-09 | 0.008584212 | 9.216455911 |  |
| Nap during day | 11 | rs11224896 | C | T | 0.110091 | 462400 | -0.0111859 | 0.00195596 | 1.10E-08 | 0.008403462 | 9.020748307 |  |
| Nap during day | 11 | rs10840017 | G | A | 0.233223 | 462400 | -0.00866558 | 0.00149279 | 6.40E-09 | 0.008537877 | 9.166279762 |  |
| Nap during day | 12 | rs4402351 | G | A | 0.148069 | 462400 | -0.0117649 | 0.00173327 | 1.10E-11 | 0.009988888 | 10.73980591 |  |
| Nap during day | 12 | rs1479116 | A | G | 0.351118 | 462400 | 0.00796453 | 0.00129506 | 7.80E-10 | 0.009042219 | 9.712682425 |  |
| Nap during day | 12 | rs35011311 | T | G | 0.264996 | 462400 | -0.00946899 | 0.00139578 | 1.20E-11 | 0.009970417 | 10.71974544 |  |
| Nap during day | 12 | rs11615756 | T | C | 0.403516 | 462400 | 0.0182385 | 0.00125078 | 3.70E-48 | 0.021438048 | 23.31933995 |  |
| Nap during day | 13 | rs2769916 | A | G | 0.688798 | 462400 | 0.00877372 | 0.00132557 | 3.60E-11 | 0.009734371 | 10.46346497 |  |
| Nap during day | 14 | rs2370926 | C | T | 0.367305 | 462400 | -0.00828326 | 0.00127397 | 7.90E-11 | 0.009562086 | 10.27648839 |  |
| Nap during day | 14 | rs10150432 | G | A | 0.184721 | 462400 | 0.0104521 | 0.00157996 | 3.70E-11 | 0.009728413 | 10.45699781 |  |
| Nap during day | 15 | rs11071755 | A | G | 0.424644 | 462400 | -0.00696004 | 0.00123874 | 1.90E-08 | 0.008265721 | 8.871657449 |  |
| Nap during day | 15 | rs17158413 | A | G | 0.237392 | 462400 | 0.00855728 | 0.00144202 | 3.00E-09 | 0.008722626 | 9.366372411 |  |
| Nap during day | 16 | rs7191614 | G | A | 0.290789 | 462400 | 0.00769805 | 0.00135296 | 1.30E-08 | 0.008361588 | 8.975419578 |  |
| Nap during day | 16 | rs7198121 | C | T | 0.538397 | 462400 | -0.00681975 | 0.00123097 | 3.00E-08 | 0.00814892 | 8.745263564 |  |
| Nap during day | 16 | rs60920123 | A | G | 0.432894 | 462400 | -0.00744881 | 0.00123803 | 1.80E-09 | 0.008845113 | 9.499072146 |  |
| Nap during day | 16 | rs8050478 | A | G | 0.500601 | 462400 | -0.00763479 | 0.0012248 | 4.60E-10 | 0.009164599 | 9.845352819 |  |
| Nap during day | 17 | rs3935190 | A | G | 0.536792 | 462400 | 0.00826969 | 0.00123459 | 2.10E-11 | 0.00985087 | 10.58993581 |  |
| Nap during day | 17 | rs12451365 | C | T | 0.204133 | 462400 | 0.0108452 | 0.00151874 | 9.30E-13 | 0.010500154 | 11.29533904 |  |
| Nap during day | 18 | rs962247 | A | G | 0.475853 | 462400 | -0.00817957 | 0.00123599 | 3.60E-11 | 0.009734371 | 10.46346497 |  |
| Nap during day | 18 | rs9965170 | A | G | 0.423866 | 462400 | -0.014193 | 0.00123929 | 2.30E-30 | 0.016838713 | 18.2307047 |  |
| Nap during day | 18 | rs2033103 | T | C | 0.450439 | 462400 | 0.00753532 | 0.00123118 | 9.30E-10 | 0.009001101 | 9.668115493 |  |
| Nap during day | 20 | rs3810484 | G | A | 0.443064 | 462400 | -0.00710613 | 0.00123272 | 8.20E-09 | 0.00847662 | 9.099951886 |  |
| Nap during day | 20 | rs17265513 | C | T | 0.198242 | 462400 | 0.00888057 | 0.00153558 | 7.30E-09 | 0.008505408 | 9.13112224 |  |
| Nap during day | 20 | rs910187 | A | G | 0.373013 | 462400 | -0.00724033 | 0.00126743 | 1.10E-08 | 0.008403462 | 9.020748307 |  |
| Nap during day | 21 | rs1883048 | C | T | 0.524834 | 462400 | 0.0078482 | 0.00123337 | 2.00E-10 | 0.009354524 | 10.05131253 |  |
| Nap during day | 22 | rs2284016 | C | T | 0.399515 | 462400 | 0.0070943 | 0.0012508 | 1.40E-08 | 0.008342945 | 8.955239427 |  |
| Sleep duration | 1 | rs915416 | G | C | 0.70906 | 460099 | -0.0127275 | 0.00176031 | 4.80E-13 | 0.010659588 | 11.41157099 | 51.28360354 |
| Sleep duration | 1 | rs2186122 | T | A | 0.559911 | 460099 | -0.0108333 | 0.00162062 | 2.30E-11 | 0.009855857 | 10.54257609 |  |
| Sleep duration | 1 | rs2279681 | G | C | 0.341785 | 460099 | 0.00929853 | 0.00168475 | 3.40E-08 | 0.008136905 | 8.688768978 |  |
| Sleep duration | 1 | rs7517981 | C | T | 0.601498 | 460099 | -0.0099657 | 0.00163399 | 1.10E-09 | 0.00898407 | 9.601592335 |  |
| Sleep duration | 1 | rs12567114 | A | G | 0.276368 | 460099 | 0.0123378 | 0.00179427 | 6.10E-12 | 0.010138442 | 10.84794594 |  |
| Sleep duration | 1 | rs1463053 | A | G | 0.639694 | 460099 | 0.0092696 | 0.00166133 | 2.40E-08 | 0.00822667 | 8.785417669 |  |
| Sleep duration | 1 | rs6681755 | A | G | 0.199783 | 460099 | 0.0115272 | 0.00200498 | 9.00E-09 | 0.008474611 | 9.052462166 |  |
| Sleep duration | 2 | rs374153 | T | C | 0.842567 | 460099 | -0.0130999 | 0.00219716 | 2.50E-09 | 0.008788422 | 9.390641848 |  |
| Sleep duration | 2 | rs2863957 | A | C | 0.220537 | 460099 | 0.0289035 | 0.00192922 | 9.60E-51 | 0.022082314 | 23.9162473 |  |
| Sleep duration | 2 | rs1972712 | C | T | 0.249455 | 460099 | 0.011796 | 0.00184758 | 1.70E-10 | 0.009414606 | 10.06609426 |  |
| Sleep duration | 2 | rs72831782 | A | T | 0.269409 | 460099 | -0.0101806 | 0.00184372 | 3.40E-08 | 0.008136905 | 8.688768978 |  |
| Sleep duration | 2 | rs2683630 | G | C | 0.629076 | 460099 | 0.0149506 | 0.0016555 | 1.70E-19 | 0.013313022 | 14.29051934 |  |
| Sleep duration | 2 | rs75539574 | C | A | 0.085774 | 460099 | 0.0236649 | 0.00287424 | 1.80E-16 | 0.012139226 | 13.01505481 |  |
| Sleep duration | 2 | rs35662245 | A | T | 0.338713 | 460099 | 0.0101569 | 0.00169075 | 1.90E-09 | 0.008854282 | 9.461644325 |  |
| Sleep duration | 3 | rs6783516 | T | G | 0.583817 | 460099 | -0.00983781 | 0.00163116 | 1.60E-09 | 0.008895283 | 9.505851235 |  |
| Sleep duration | 3 | rs76258078 | G | A | 0.049994 | 460099 | -0.0216874 | 0.00368137 | 3.80E-09 | 0.008687029 | 9.281352273 |  |
| Sleep duration | 3 | rs113021516 | C | G | 0.335898 | 460099 | 0.0114805 | 0.00169722 | 1.30E-11 | 0.009978285 | 10.67485443 |  |
| Sleep duration | 3 | rs17732997 | G | C | 0.429679 | 460099 | -0.00883899 | 0.00161761 | 4.60E-08 | 0.008058238 | 8.604084592 |  |
| Sleep duration | 3 | rs9810474 | T | C | 0.232184 | 460099 | -0.0111549 | 0.00189404 | 3.90E-09 | 0.008680703 | 9.274533582 |  |
| Sleep duration | 3 | rs7644809 | C | T | 0.576033 | 460099 | -0.0101517 | 0.00162466 | 4.10E-10 | 0.009214016 | 9.849628948 |  |
| Sleep duration | 4 | rs13107325 | T | C | 0.074905 | 460099 | -0.0242704 | 0.00303933 | 1.40E-15 | 0.011771872 | 12.61650579 |  |
| Sleep duration | 4 | rs2192528 | G | A | 0.522493 | 460099 | -0.00980706 | 0.00160143 | 9.10E-10 | 0.009028683 | 9.64970599 |  |
| Sleep duration | 4 | rs2839753 | C | T | 0.265347 | 460099 | -0.0106369 | 0.00181248 | 4.40E-09 | 0.008651261 | 9.242803469 |  |
| Sleep duration | 5 | rs7711696 | T | G | 0.304987 | 460099 | -0.00986657 | 0.00173522 | 1.30E-08 | 0.008382469 | 8.953204835 |  |
| Sleep duration | 5 | rs12518468 | C | T | 0.328685 | 460099 | -0.0106435 | 0.00170288 | 4.10E-10 | 0.009214016 | 9.849628948 |  |
| Sleep duration | 5 | rs365663 | G | A | 0.45495 | 460099 | -0.00928337 | 0.00161004 | 8.10E-09 | 0.008500834 | 9.080712604 |  |
| Sleep duration | 5 | rs6889592 | A | G | 0.332608 | 460099 | 0.0117662 | 0.00169707 | 4.10E-12 | 0.01022155 | 10.93778877 |  |
| Sleep duration | 5 | rs151014368 | A | G | 0.20734 | 460099 | 0.0113794 | 0.00198822 | 1.00E-08 | 0.008448307 | 9.024124192 |  |
| Sleep duration | 6 | rs9382445 | C | T | 0.375168 | 460099 | -0.00948383 | 0.00164901 | 8.90E-09 | 0.008477395 | 9.055460912 |  |
| Sleep duration | 6 | rs113113059 | C | T | 0.219834 | 460099 | -0.0111278 | 0.00193332 | 8.60E-09 | 0.008485935 | 9.064661509 |  |
| Sleep duration | 6 | rs7740402 | G | T | 0.3061 | 460099 | -0.00951481 | 0.00173455 | 4.10E-08 | 0.008088272 | 8.636415175 |  |
| Sleep duration | 6 | rs9345234 | C | A | 0.57786 | 460099 | 0.0091922 | 0.00162351 | 1.50E-08 | 0.008346349 | 8.91430112 |  |
| Sleep duration | 6 | rs34556183 | G | A | 0.279888 | 460099 | -0.0133534 | 0.00178155 | 6.60E-14 | 0.011049827 | 11.83400702 |  |
| Sleep duration | 7 | rs11982852 | T | C | 0.243858 | 460099 | -0.0117257 | 0.00186248 | 3.10E-10 | 0.009278168 | 9.918848245 |  |
| Sleep duration | 7 | rs62444917 | C | A | 0.222315 | 460099 | 0.0129633 | 0.00192625 | 1.70E-11 | 0.009920908 | 10.61285632 |  |
| Sleep duration | 7 | rs2079070 | G | C | 0.734582 | 460099 | -0.0134358 | 0.00181083 | 1.20E-13 | 0.010933634 | 11.708193 |  |
| Sleep duration | 8 | rs7831557 | A | G | 0.517438 | 460099 | -0.0105656 | 0.00160135 | 4.20E-11 | 0.009725057 | 10.40128788 |  |
| Sleep duration | 8 | rs7016314 | C | T | 0.655927 | 460099 | 0.0100011 | 0.00168824 | 3.10E-09 | 0.008736471 | 9.334642119 |  |
| Sleep duration | 9 | rs17391944 | G | T | 0.04986 | 460099 | 0.0218515 | 0.00372396 | 4.40E-09 | 0.008651261 | 9.242803469 |  |
| Sleep duration | 10 | rs112100783 | A | G | 0.033439 | 460099 | -0.0252948 | 0.00454852 | 2.70E-08 | 0.00819642 | 8.752846476 |  |
| Sleep duration | 10 | rs10510128 | A | G | 0.207951 | 460099 | 0.0114031 | 0.00197431 | 7.70E-09 | 0.008513416 | 9.094268414 |  |
| Sleep duration | 10 | rs2236295 | T | G | 0.403043 | 460099 | -0.00907587 | 0.00163637 | 2.90E-08 | 0.008178013 | 8.733027515 |  |
| Sleep duration | 11 | rs1517572 | C | A | 0.581256 | 460099 | 0.0116589 | 0.00162182 | 6.50E-13 | 0.010598723 | 11.34571381 |  |
| Sleep duration | 11 | rs7115856 | C | A | 0.461273 | 460099 | 0.010819 | 0.00160279 | 1.50E-11 | 0.009947691 | 10.64179526 |  |
| Sleep duration | 11 | rs11039216 | T | C | 0.532944 | 460099 | -0.0102766 | 0.00160373 | 1.50E-10 | 0.009442794 | 10.09652009 |  |
| Sleep duration | 11 | rs2734831 | G | T | 0.606881 | 460099 | -0.00979722 | 0.00163853 | 2.20E-09 | 0.008819158 | 9.423776448 |  |
| Sleep duration | 11 | rs174564 | G | A | 0.348602 | 460099 | 0.00974534 | 0.001678 | 6.30E-09 | 0.008563087 | 9.147786651 |  |
| Sleep duration | 11 | rs1939455 | T | G | 0.120291 | 460099 | -0.0158002 | 0.0025174 | 3.50E-10 | 0.009250371 | 9.888854158 |  |
| Sleep duration | 11 | rs1553132 | G | A | 0.258638 | 460099 | 0.0105257 | 0.0018253 | 8.10E-09 | 0.008500834 | 9.080712604 |  |
| Sleep duration | 12 | rs34354917 | A | C | 0.288625 | 460099 | -0.0100207 | 0.00176809 | 1.40E-08 | 0.008363779 | 8.933074215 |  |
| Sleep duration | 12 | rs4767550 | G | A | 0.413171 | 460099 | 0.0108732 | 0.00163264 | 2.70E-11 | 0.009821157 | 10.50508946 |  |
| Sleep duration | 13 | rs6561715 | A | T | 0.630636 | 460099 | 0.00978234 | 0.001661 | 3.90E-09 | 0.008680703 | 9.274533582 |  |
| Sleep duration | 14 | rs55658675 | T | C | 0.352906 | 460099 | -0.00969462 | 0.00167463 | 7.10E-09 | 0.008533528 | 9.115937748 |  |
| Sleep duration | 14 | rs11621908 | T | C | 0.082807 | 460099 | -0.019985 | 0.00294327 | 1.10E-11 | 0.010013832 | 10.71326773 |  |
| Sleep duration | 14 | rs2748809 | C | T | 0.429258 | 460099 | -0.00925348 | 0.00164549 | 1.90E-08 | 0.008286363 | 8.849697405 |  |
| Sleep duration | 15 | rs8038326 | G | A | 0.273169 | 460099 | -0.0133838 | 0.00179311 | 8.40E-14 | 0.011003081 | 11.78338668 |  |
| Sleep duration | 16 | rs56367859 | G | A | 0.397585 | 460099 | 0.0116218 | 0.00163585 | 1.20E-12 | 0.01047461 | 11.21144738 |  |
| Sleep duration | 16 | rs9302680 | A | G | 0.439272 | 460099 | 0.0120438 | 0.00161069 | 7.60E-14 | 0.011022507 | 11.80442264 |  |
| Sleep duration | 16 | rs11643715 | G | C | 0.292625 | 460099 | 0.0109493 | 0.00176035 | 5.00E-10 | 0.009168217 | 9.800218059 |  |
| Sleep duration | 16 | rs8047587 | T | G | 0.439514 | 460099 | -0.0110198 | 0.00161283 | 8.30E-12 | 0.010073524 | 10.77777873 |  |
| Sleep duration | 16 | rs72771082 | G | A | 0.217832 | 460099 | 0.0109737 | 0.00193583 | 1.40E-08 | 0.008363779 | 8.933074215 |  |
| Sleep duration | 17 | rs8074498 | A | T | 0.580951 | 460099 | -0.00932835 | 0.00163392 | 1.10E-08 | 0.008424447 | 8.998422313 |  |
| Sleep duration | 17 | rs11650677 | A | G | 0.339185 | 460099 | 0.0111701 | 0.00168963 | 3.80E-11 | 0.009746882 | 10.42486016 |  |
| Sleep duration | 17 | rs9903898 | T | C | 0.488883 | 460099 | -0.00944768 | 0.00160094 | 3.60E-09 | 0.008700185 | 9.29553103 |  |
| Sleep duration | 17 | rs8072993 | G | T | 0.635677 | 460099 | 0.0109222 | 0.00199248 | 4.20E-08 | 0.008081989 | 8.629651216 |  |
| Sleep duration | 18 | rs1348047 | T | G | 0.267253 | 460099 | -0.012642 | 0.00182049 | 3.80E-12 | 0.010237375 | 10.95489786 |  |
| Sleep duration | 19 | rs35126035 | C | A | 0.55833 | 460099 | -0.00919552 | 0.00164378 | 2.20E-08 | 0.00824895 | 8.809408981 |  |
| Sleep duration | 19 | rs34786000 | T | G | 0.553361 | 460099 | 0.0109576 | 0.00162787 | 1.70E-11 | 0.009920908 | 10.61285632 |  |
| Sleep duration | 20 | rs2072727 | C | T | 0.56459 | 460099 | -0.00926905 | 0.00161401 | 9.30E-09 | 0.008466434 | 9.043652433 |  |
| Sleep duration | 22 | rs9611007 | T | C | 0.141673 | 460099 | -0.0135938 | 0.00229721 | 3.30E-09 | 0.008721318 | 9.31830921 |  |
| Sleeplessness | 1 | rs2803296 | C | G | 0.543586 | 462341 | -0.00862206 | 0.00149047 | 7.30E-09 | 0.008505951 | 9.130543538 | 59.20613991 |
| Sleeplessness | 1 | rs12049261 | C | G | 0.292534 | 462341 | 0.0111868 | 0.00163048 | 6.80E-12 | 0.010091033 | 10.84936308 |  |
| Sleeplessness | 1 | rs6690017 | G | T | 0.408855 | 462341 | -0.0102669 | 0.00151022 | 1.10E-11 | 0.009989526 | 10.73912627 |  |
| Sleeplessness | 1 | rs2644128 | G | C | 0.548279 | 462341 | 0.0106284 | 0.00149103 | 1.00E-12 | 0.010486155 | 11.27867953 |  |
| Sleeplessness | 2 | rs4572538 | T | C | 0.364055 | 462341 | -0.00960596 | 0.00156161 | 7.70E-10 | 0.009045803 | 9.715326648 |  |
| Sleeplessness | 2 | rs56365214 | A | C | 0.155781 | 462341 | -0.0147948 | 0.00205225 | 5.60E-13 | 0.010602867 | 11.40555813 |  |
| Sleeplessness | 2 | rs4577309 | G | A | 0.533655 | 462341 | -0.00854833 | 0.00149214 | 1.00E-08 | 0.008427799 | 9.045940032 |  |
| Sleeplessness | 2 | rs12470989 | G | A | 0.203934 | 462341 | -0.0102429 | 0.00184485 | 2.80E-08 | 0.008167182 | 8.763904863 |  |
| Sleeplessness | 2 | rs113851554 | T | G | 0.057291 | 462341 | 0.0467802 | 0.00331329 | 2.90E-45 | 0.020760158 | 22.5634468 |  |
| Sleeplessness | 2 | rs56093896 | A | C | 0.214053 | 462341 | -0.0124111 | 0.00181352 | 7.70E-12 | 0.010064888 | 10.82096771 |  |
| Sleeplessness | 3 | rs2014830 | T | C | 0.303519 | 462341 | -0.0116018 | 0.0016233 | 8.90E-13 | 0.010509709 | 11.30428298 |  |
| Sleeplessness | 3 | rs705219 | A | T | 0.887373 | 462341 | 0.013423 | 0.00235299 | 1.20E-08 | 0.008382209 | 8.996592214 |  |
| Sleeplessness | 3 | rs9845387 | A | C | 0.040279 | 462341 | -0.0218562 | 0.00377648 | 7.10E-09 | 0.008512814 | 9.137973658 |  |
| Sleeplessness | 4 | rs1988337 | G | A | 0.552395 | 462341 | 0.00838726 | 0.00149645 | 2.10E-08 | 0.008240785 | 8.843541559 |  |
| Sleeplessness | 4 | rs11097861 | G | A | 0.716256 | 462341 | 0.0100437 | 0.00164946 | 1.10E-09 | 0.008962262 | 9.624791688 |  |
| Sleeplessness | 4 | rs2604551 | G | T | 0.640384 | 462341 | -0.00848084 | 0.00155238 | 4.70E-08 | 0.008033066 | 8.618824495 |  |
| Sleeplessness | 5 | rs1592757 | C | G | 0.355788 | 462341 | 0.0102216 | 0.00154996 | 4.30E-11 | 0.009696277 | 10.42078561 |  |
| Sleeplessness | 5 | rs7711696 | T | G | 0.305042 | 462341 | 0.0111716 | 0.00161138 | 4.10E-12 | 0.010196739 | 10.9641837 |  |
| Sleeplessness | 5 | rs1430205 | T | C | 0.461507 | 462341 | 0.00947493 | 0.00149107 | 2.10E-10 | 0.009344096 | 10.03872033 |  |
| Sleeplessness | 6 | rs314280 | G | A | 0.547047 | 462341 | 0.00971363 | 0.00149134 | 7.30E-11 | 0.009580146 | 10.29477001 |  |
| Sleeplessness | 7 | rs6975972 | G | A | 0.578726 | 462341 | -0.00902066 | 0.00150428 | 2.00E-09 | 0.008820543 | 9.471241485 |  |
| Sleeplessness | 7 | rs8180817 | C | G | 0.431016 | 462341 | -0.0100553 | 0.00150974 | 2.70E-11 | 0.009797317 | 10.53045044 |  |
| Sleeplessness | 8 | rs17151854 | T | G | 0.15241 | 462341 | 0.0129893 | 0.00207434 | 3.80E-10 | 0.009209085 | 9.892324274 |  |
| Sleeplessness | 9 | rs11790060 | C | T | 0.330834 | 462341 | -0.0103391 | 0.00157854 | 5.80E-11 | 0.009630796 | 10.34972742 |  |
| Sleeplessness | 10 | rs224032 | A | G | 0.550358 | 462341 | 0.00839066 | 0.00149108 | 1.80E-08 | 0.008279971 | 8.885944384 |  |
| Sleeplessness | 10 | rs17709610 | G | A | 0.297979 | 462341 | -0.0099161 | 0.00162078 | 9.50E-10 | 0.00899669 | 9.662099873 |  |
| Sleeplessness | 10 | rs2297787 | A | T | 0.080193 | 462341 | -0.0178001 | 0.00274996 | 9.60E-11 | 0.009519475 | 10.22894717 |  |
| Sleeplessness | 11 | rs72924721 | T | C | 0.073137 | 462341 | 0.016478 | 0.00288103 | 1.10E-08 | 0.008403998 | 9.020176541 |  |
| Sleeplessness | 11 | rs10838708 | A | G | 0.45899 | 462341 | -0.00947656 | 0.00150271 | 2.90E-10 | 0.009270848 | 9.959290411 |  |
| Sleeplessness | 12 | rs68094047 | T | C | 0.251267 | 462341 | 0.0103356 | 0.0017167 | 1.70E-09 | 0.008859282 | 9.51320968 |  |
| Sleeplessness | 12 | rs931221 | A | T | 0.236738 | 462341 | 0.0106361 | 0.00175313 | 1.30E-09 | 0.008922878 | 9.582114676 |  |
| Sleeplessness | 12 | rs324017 | C | A | 0.705433 | 462341 | -0.00988248 | 0.00163145 | 1.40E-09 | 0.00890535 | 9.563122345 |  |
| Sleeplessness | 13 | rs9570080 | C | T | 0.344131 | 462341 | -0.0106379 | 0.00157858 | 1.60E-11 | 0.009909775 | 10.65253361 |  |
| Sleeplessness | 13 | rs6561715 | A | T | 0.630662 | 462341 | -0.011623 | 0.00154234 | 4.80E-14 | 0.011084254 | 11.92919266 |  |
| Sleeplessness | 13 | rs1547630 | A | G | 0.651513 | 462341 | 0.00910814 | 0.00156447 | 5.80E-09 | 0.008562639 | 9.191919432 |  |
| Sleeplessness | 15 | rs4886860 | C | G | 0.767408 | 462341 | -0.011796 | 0.00175566 | 1.80E-11 | 0.009884552 | 10.62514899 |  |
| Sleeplessness | 15 | rs11635495 | C | T | 0.512179 | 462341 | 0.00937342 | 0.00148509 | 2.80E-10 | 0.009278838 | 9.967954218 |  |
| Sleeplessness | 16 | rs2062113 | C | T | 0.568257 | 462341 | -0.00961678 | 0.00150275 | 1.60E-10 | 0.009405384 | 10.10518853 |  |
| Sleeplessness | 17 | rs9894577 | A | G | 0.3182 | 462341 | 0.0132051 | 0.00159687 | 1.30E-16 | 0.012166943 | 13.10876525 |  |
| Sleeplessness | 17 | rs9906181 | G | A | 0.687567 | 462341 | -0.00915026 | 0.00163869 | 2.40E-08 | 0.0082067 | 8.806661175 |  |
| Sleeplessness | 18 | rs11152363 | A | G | 0.186319 | 462341 | 0.0156393 | 0.00192518 | 4.50E-16 | 0.011947421 | 12.86939104 |  |
| Sleeplessness | 19 | rs56330606 | G | A | 0.378954 | 462341 | 0.00930932 | 0.00153044 | 1.20E-09 | 0.008941767 | 9.602582824 |  |
