# Supplementary material for: Association between Sleep Traits and Lung Cancer: A Mendelian Randomization Study
Source: J Immunol Res. 2021 Jun 21;2021:1893882. doi: 10.1155/2021/1893882 (PMC8238591; doi:10.1155/2021/1893882)
Supplement: Supplementary Materials — Supplementary Table 1: two-sample Mendelian randomization estimations showing the effect of sleep traits on cancer using the MR Egger, weighted median, and weighted mode method. Supplementary Table 2: sensitivity analysis performed by Egger regression intercept and heterogeneity test. Supplementary Table 3: SNPs of sleep traits extracted from UK Biobank with statistically significant threshold [P < 5 × 10^−8^; linkage disequilibrium (LD) r2 < 0.001, LD distance > 10000 kb]. Supplementary Table 4: SNPs used in two-sample Mendelian randomization analysis. Supplementary Table 5: outliers selected by RadialMR and the reanalysis results after excluding outliers. Supplementary Table 6: multivariable two-sample Mendelian randomization estimation showing the effects of different sleep traits on lung cancer. [file 1893882.f1.zip › Supplementary Table 4 (1).docx]

Supplementary Table 4: SNPs used in Two-sample Mendelian randomization analysis.

| exposure | SNP | effect_allele.exposure | other_allele.exposure | effect_allele.outcome | other_allele.outcome | beta.exposure | beta.outcome | eaf.exposure | eaf.outcome | remove | chr | se.outcome | samplesize.outcome | pval.outcome | chr.exposure | se.exposure | samplesize.exposure | pval.exposure | outcome |
| --- | --- | --- | --- | --- | --- | --- | --- | --- | --- | --- | --- | --- | --- | --- | --- | --- | --- | --- | --- |
| Nap during day | rs1001817 | T | C | T | C | -0.008 | 0.020 | 0.494 | 0.508 | FALSE | 3 | 0.018 | 27209 | 0.271 | 3 | 0.001 | 462400 | 2.30E-10 | Lung cancer |
| Nap during day | rs1011024 | G | A | G | A | -0.010 | -0.030 | 0.155 | 0.159 | FALSE | 7 | 0.023 | 27209 | 0.219 | 7 | 0.002 | 462400 | 1.70E-09 | Lung cancer |
| Nap during day | rs10150432 | G | A | G | A | 0.010 | 0.029 | 0.185 | 0.184 | FALSE | 14 | 0.024 | 27209 | 0.222 | 14 | 0.002 | 462400 | 3.70E-11 | Lung cancer |
| Nap during day | rs10757347 | G | A | G | A | 0.008 | -0.008 | 0.223 | 0.210 | FALSE | 9 | 0.022 | 27209 | 0.714 | 9 | 0.001 | 462400 | 4.10E-08 | Lung cancer |
| Nap during day | rs10764260 | A | G | A | G | -0.007 | 0.007 | 0.363 | 0.368 | FALSE | 10 | 0.018 | 27209 | 0.711 | 10 | 0.001 | 462400 | 1.00E-08 | Lung cancer |
| Nap during day | rs10835420 | A | T | A | T | -0.009 | 0.021 | 0.249 | 0.249 | FALSE | 11 | 0.022 | 27209 | 0.322 | 11 | 0.001 | 462400 | 1.30E-09 | Lung cancer |
| Nap during day | rs10840017 | G | A | G | A | -0.009 | 0.029 | 0.233 | 0.235 | FALSE | 11 | 0.026 | 27209 | 0.257 | 11 | 0.001 | 462400 | 6.40E-09 | Lung cancer |
| Nap during day | rs10868046 | A | G | A | G | 0.007 | 0.012 | 0.604 | 0.586 | FALSE | 9 | 0.018 | 27209 | 0.513 | 9 | 0.001 | 462400 | 7.90E-09 | Lung cancer |
| Nap during day | rs10875622 | A | G | A | G | 0.010 | 0.003 | 0.576 | 0.574 | FALSE | 5 | 0.018 | 27209 | 0.867 | 5 | 0.001 | 462400 | 1.30E-16 | Lung cancer |
| Nap during day | rs11071755 | A | G | A | G | -0.007 | 0.049 | 0.425 | 0.398 | FALSE | 15 | 0.020 | 27209 | 0.010 | 15 | 0.001 | 462400 | 1.90E-08 | Lung cancer |
| Nap during day | rs11121194 | T | C | T | C | 0.007 | 0.005 | 0.634 | 0.613 | FALSE | 1 | 0.018 | 27209 | 0.786 | 1 | 0.001 | 462400 | 2.40E-08 | Lung cancer |
| Nap during day | rs11125776 | G | T | G | T | -0.012 | 0.008 | 0.144 | 0.134 | FALSE | 2 | 0.026 | 27209 | 0.773 | 2 | 0.002 | 462400 | 5.60E-12 | Lung cancer |
| Nap during day | rs11224896 | C | T | C | T | -0.011 | -0.034 | 0.110 | 0.099 | FALSE | 11 | 0.029 | 27209 | 0.263 | 11 | 0.002 | 462400 | 1.10E-08 | Lung cancer |
| Nap during day | rs11252681 | A | G | A | G | 0.016 | 0.007 | 0.045 | 0.054 | FALSE | 10 | 0.044 | 27209 | 0.874 | 10 | 0.003 | 462400 | 4.10E-08 | Lung cancer |
| Nap during day | rs11258652 | A | C | A | C | -0.010 | -0.024 | 0.236 | 0.235 | FALSE | 10 | 0.020 | 27209 | 0.266 | 10 | 0.001 | 462400 | 4.10E-12 | Lung cancer |
| Nap during day | rs113886333 | T | C | T | C | 0.018 | 0.051 | 0.036 | 0.039 | FALSE | 4 | 0.049 | 27209 | 0.288 | 4 | 0.003 | 462400 | 3.00E-08 | Lung cancer |
| Nap during day | rs11615756 | T | C | T | C | 0.018 | 0.035 | 0.404 | 0.394 | FALSE | 12 | 0.019 | 27209 | 0.057 | 12 | 0.001 | 462400 | 3.70E-48 | Lung cancer |
| Nap during day | rs12042846 | C | T | C | T | 0.009 | 0.005 | 0.179 | 0.165 | FALSE | 1 | 0.025 | 27209 | 0.844 | 1 | 0.002 | 462400 | 1.80E-08 | Lung cancer |
| Nap during day | rs12140153 | T | G | T | G | -0.024 | 0.030 | 0.094 | 0.082 | FALSE | 1 | 0.037 | 27209 | 0.424 | 1 | 0.002 | 462400 | 1.20E-29 | Lung cancer |
| Nap during day | rs12346996 | C | T | C | T | -0.008 | 0.001 | 0.729 | 0.730 | FALSE | 9 | 0.021 | 27209 | 0.952 | 9 | 0.001 | 462400 | 1.50E-09 | Lung cancer |
| Nap during day | rs12451365 | C | T | C | T | 0.011 | -0.037 | 0.204 | 0.208 | FALSE | 17 | 0.021 | 27209 | 0.104 | 17 | 0.002 | 462400 | 9.30E-13 | Lung cancer |
| Nap during day | rs12615434 | T | C | T | C | 0.012 | 0.007 | 0.113 | 0.105 | FALSE | 2 | 0.029 | 27209 | 0.805 | 2 | 0.002 | 462400 | 1.80E-09 | Lung cancer |
| Nap during day | rs12657723 | T | C | T | C | 0.008 | 0.029 | 0.321 | 0.320 | FALSE | 5 | 0.020 | 27209 | 0.142 | 5 | 0.001 | 462400 | 4.40E-10 | Lung cancer |
| Nap during day | rs12992648 | G | A | G | A | -0.008 | 0.003 | 0.282 | 0.283 | FALSE | 2 | 0.021 | 27209 | 0.882 | 2 | 0.001 | 462400 | 2.10E-08 | Lung cancer |
| Nap during day | rs13023587 | G | C | G | C | -0.008 | -0.007 | 0.508 | 0.498 | FALSE | 2 | 0.018 | 27209 | 0.688 | 2 | 0.001 | 462400 | 9.30E-11 | Lung cancer |
| Nap during day | rs13033444 | G | A | G | A | 0.009 | 0.039 | 0.282 | 0.278 | FALSE | 2 | 0.021 | 27209 | 0.060 | 2 | 0.001 | 462400 | 4.60E-12 | Lung cancer |
| Nap during day | rs13266972 | G | A | G | A | -0.007 | 0.019 | 0.698 | 0.694 | FALSE | 8 | 0.019 | 27209 | 0.345 | 8 | 0.001 | 462400 | 4.70E-08 | Lung cancer |
| Nap during day | rs13284688 | C | T | C | T | 0.014 | 0.066 | 0.207 | 0.193 | FALSE | 9 | 0.024 | 27209 | 0.004 | 9 | 0.002 | 462400 | 1.40E-21 | Lung cancer |
| Nap during day | rs1479116 | A | G | A | G | 0.008 | -0.002 | 0.351 | 0.349 | FALSE | 12 | 0.019 | 27209 | 0.927 | 12 | 0.001 | 462400 | 7.80E-10 | Lung cancer |
| Nap during day | rs17158413 | A | G | A | G | 0.009 | -0.050 | 0.237 | 0.222 | FALSE | 15 | 0.021 | 27209 | 0.025 | 15 | 0.001 | 462400 | 3.00E-09 | Lung cancer |
| Nap during day | rs17265513 | C | T | C | T | 0.009 | -0.022 | 0.198 | 0.197 | FALSE | 20 | 0.022 | 27209 | 0.343 | 20 | 0.002 | 462400 | 7.30E-09 | Lung cancer |
| Nap during day | rs174541 | C | T | C | T | 0.010 | -0.070 | 0.359 | 0.361 | FALSE | 11 | 0.018 | 27209 | 0.000 | 11 | 0.001 | 462400 | 1.40E-15 | Lung cancer |
| Nap during day | rs1856502 | A | T | A | T | 0.007 | -0.020 | 0.457 | 0.383 | FALSE | 6 | 0.019 | 27209 | 0.318 | 6 | 0.001 | 462400 | 7.20E-09 | Lung cancer |
| Nap during day | rs1883048 | C | T | C | T | 0.008 | 0.035 | 0.525 | 0.541 | FALSE | 21 | 0.018 | 27209 | 0.074 | 21 | 0.001 | 462400 | 2.00E-10 | Lung cancer |
| Nap during day | rs1931175 | G | C | G | C | 0.007 | 0.019 | 0.383 | 0.361 | FALSE | 1 | 0.019 | 27209 | 0.311 | 1 | 0.001 | 462400 | 3.20E-09 | Lung cancer |
| Nap during day | rs2033103 | T | C | T | C | 0.008 | 0.012 | 0.450 | 0.453 | FALSE | 18 | 0.018 | 27209 | 0.508 | 18 | 0.001 | 462400 | 9.30E-10 | Lung cancer |
| Nap during day | rs2099810 | G | A | G | A | -0.008 | -0.001 | 0.496 | 0.496 | FALSE | 5 | 0.018 | 27209 | 0.965 | 5 | 0.001 | 462400 | 1.90E-10 | Lung cancer |
| Nap during day | rs2143792 | A | G | A | G | -0.007 | -0.023 | 0.431 | 0.427 | FALSE | 6 | 0.018 | 27209 | 0.213 | 6 | 0.001 | 462400 | 6.80E-09 | Lung cancer |
| Nap during day | rs224111 | A | G | A | G | -0.008 | 0.006 | 0.389 | 0.406 | FALSE | 10 | 0.018 | 27209 | 0.746 | 10 | 0.001 | 462400 | 4.60E-10 | Lung cancer |
| Nap during day | rs2284016 | C | T | C | T | 0.007 | -0.030 | 0.400 | 0.426 | FALSE | 22 | 0.018 | 27209 | 0.111 | 22 | 0.001 | 462400 | 1.40E-08 | Lung cancer |
| Nap during day | rs2370926 | C | T | C | T | -0.008 | 0.013 | 0.367 | 0.355 | FALSE | 14 | 0.019 | 27209 | 0.492 | 14 | 0.001 | 462400 | 7.90E-11 | Lung cancer |
| Nap during day | rs2390669 | C | A | C | A | -0.011 | 0.014 | 0.129 | 0.135 | FALSE | 2 | 0.027 | 27209 | 0.599 | 2 | 0.002 | 462400 | 8.40E-10 | Lung cancer |
| Nap during day | rs2431108 | C | T | C | T | 0.013 | -0.021 | 0.328 | 0.329 | FALSE | 5 | 0.019 | 27209 | 0.272 | 5 | 0.001 | 462400 | 7.50E-22 | Lung cancer |
| Nap during day | rs2653349 | G | A | G | A | -0.016 | -0.041 | 0.787 | 0.800 | FALSE | 6 | 0.023 | 27209 | 0.072 | 6 | 0.001 | 462400 | 1.00E-27 | Lung cancer |
| Nap during day | rs2769916 | A | G | A | G | 0.009 | -0.013 | 0.689 | 0.693 | FALSE | 13 | 0.020 | 27209 | 0.507 | 13 | 0.001 | 462400 | 3.60E-11 | Lung cancer |
| Nap during day | rs2786547 | T | C | T | C | -0.011 | 0.000 | 0.177 | 0.198 | FALSE | 1 | 0.023 | 27209 | 0.997 | 1 | 0.002 | 462400 | 6.80E-12 | Lung cancer |
| Nap during day | rs285815 | A | T | A | T | -0.007 | 0.015 | 0.546 | 0.556 | FALSE | 8 | 0.018 | 27209 | 0.416 | 8 | 0.001 | 462400 | 2.00E-09 | Lung cancer |
| Nap during day | rs34262487 | A | C | A | C | -0.015 | -0.009 | 0.073 | 0.082 | FALSE | 6 | 0.032 | 27209 | 0.797 | 6 | 0.002 | 462400 | 6.10E-10 | Lung cancer |
| Nap during day | rs351776 | C | A | C | A | 0.008 | -0.002 | 0.548 | 0.552 | FALSE | 8 | 0.018 | 27209 | 0.926 | 8 | 0.001 | 462400 | 5.70E-10 | Lung cancer |
| Nap during day | rs35851551 | G | A | G | A | -0.012 | 0.030 | 0.101 | 0.095 | FALSE | 7 | 0.033 | 27209 | 0.357 | 7 | 0.002 | 462400 | 5.50E-09 | Lung cancer |
| Nap during day | rs3810484 | G | A | G | A | -0.007 | -0.037 | 0.443 | 0.434 | FALSE | 20 | 0.020 | 27209 | 0.083 | 20 | 0.001 | 462400 | 8.20E-09 | Lung cancer |
| Nap during day | rs3935190 | A | G | A | G | 0.008 | -0.021 | 0.537 | 0.539 | FALSE | 17 | 0.019 | 27209 | 0.250 | 17 | 0.001 | 462400 | 2.10E-11 | Lung cancer |
| Nap during day | rs40005 | A | G | A | G | 0.008 | 0.009 | 0.769 | 0.763 | FALSE | 3 | 0.021 | 27209 | 0.677 | 3 | 0.001 | 462400 | 2.80E-08 | Lung cancer |
| Nap during day | rs4402351 | G | A | G | A | -0.012 | 0.069 | 0.148 | 0.171 | FALSE | 12 | 0.025 | 27209 | 0.004 | 12 | 0.002 | 462400 | 1.10E-11 | Lung cancer |
| Nap during day | rs4587762 | A | G | A | G | -0.007 | -0.028 | 0.605 | 0.592 | FALSE | 11 | 0.019 | 27209 | 0.134 | 11 | 0.001 | 462400 | 3.30E-08 | Lung cancer |
| Nap during day | rs467897 | A | G | A | G | -0.009 | -0.018 | 0.680 | 0.683 | FALSE | 5 | 0.020 | 27209 | 0.362 | 5 | 0.001 | 462400 | 1.50E-12 | Lung cancer |
| Nap during day | rs4692709 | T | C | T | C | -0.007 | 0.016 | 0.547 | 0.558 | FALSE | 4 | 0.018 | 27209 | 0.383 | 4 | 0.001 | 462400 | 1.50E-08 | Lung cancer |
| Nap during day | rs4856536 | A | G | A | G | -0.009 | -0.032 | 0.729 | 0.733 | FALSE | 3 | 0.021 | 27209 | 0.115 | 3 | 0.001 | 462400 | 4.80E-10 | Lung cancer |
| Nap during day | rs60920123 | A | G | A | G | -0.007 | -0.009 | 0.433 | 0.418 | FALSE | 16 | 0.018 | 27209 | 0.618 | 16 | 0.001 | 462400 | 1.80E-09 | Lung cancer |
| Nap during day | rs614987 | C | A | C | A | 0.011 | 0.014 | 0.614 | 0.615 | FALSE | 6 | 0.018 | 27209 | 0.465 | 6 | 0.001 | 462400 | 1.30E-17 | Lung cancer |
| Nap during day | rs62425620 | T | C | T | C | 0.008 | 0.019 | 0.370 | 0.353 | FALSE | 6 | 0.020 | 27209 | 0.336 | 6 | 0.001 | 462400 | 1.20E-09 | Lung cancer |
| Nap during day | rs62560863 | T | C | T | C | 0.011 | -0.050 | 0.101 | 0.109 | FALSE | 9 | 0.028 | 27209 | 0.099 | 9 | 0.002 | 462400 | 1.80E-08 | Lung cancer |
| Nap during day | rs6452787 | G | A | G | A | -0.007 | -0.011 | 0.467 | 0.476 | FALSE | 5 | 0.018 | 27209 | 0.547 | 5 | 0.001 | 462400 | 3.50E-09 | Lung cancer |
| Nap during day | rs6483215 | G | A | G | A | -0.008 | -0.035 | 0.764 | 0.768 | FALSE | 11 | 0.022 | 27209 | 0.103 | 11 | 0.001 | 462400 | 5.30E-09 | Lung cancer |
| Nap during day | rs6919087 | G | T | G | T | -0.010 | -0.004 | 0.312 | 0.308 | FALSE | 6 | 0.019 | 27209 | 0.831 | 6 | 0.001 | 462400 | 4.30E-15 | Lung cancer |
| Nap during day | rs6942927 | A | G | A | G | 0.015 | 0.016 | 0.123 | 0.212 | FALSE | 7 | 0.022 | 27209 | 0.479 | 7 | 0.002 | 462400 | 1.50E-11 | Lung cancer |
| Nap during day | rs7038206 | G | A | G | A | 0.007 | -0.013 | 0.608 | 0.611 | FALSE | 9 | 0.018 | 27209 | 0.468 | 9 | 0.001 | 462400 | 4.00E-09 | Lung cancer |
| Nap during day | rs7191614 | G | A | G | A | 0.008 | -0.014 | 0.291 | 0.262 | FALSE | 16 | 0.020 | 27209 | 0.514 | 16 | 0.001 | 462400 | 1.30E-08 | Lung cancer |
| Nap during day | rs7198121 | C | T | C | T | -0.007 | 0.003 | 0.538 | 0.537 | FALSE | 16 | 0.018 | 27209 | 0.860 | 16 | 0.001 | 462400 | 3.00E-08 | Lung cancer |
| Nap during day | rs72781017 | G | A | G | A | -0.008 | 0.016 | 0.403 | 0.438 | FALSE | 5 | 0.018 | 27209 | 0.391 | 5 | 0.001 | 462400 | 1.30E-09 | Lung cancer |
| Nap during day | rs7422655 | T | C | T | C | -0.008 | 0.001 | 0.736 | 0.728 | FALSE | 2 | 0.020 | 27209 | 0.966 | 2 | 0.001 | 462400 | 7.10E-09 | Lung cancer |
| Nap during day | rs75022160 | T | C | T | C | -0.010 | -0.020 | 0.137 | 0.123 | FALSE | 2 | 0.027 | 27209 | 0.477 | 2 | 0.002 | 462400 | 4.90E-08 | Lung cancer |
| Nap during day | rs7555990 | T | C | T | C | -0.010 | -0.048 | 0.136 | 0.129 | FALSE | 1 | 0.025 | 27209 | 0.073 | 1 | 0.002 | 462400 | 6.80E-09 | Lung cancer |
| Nap during day | rs7698842 | G | A | G | A | 0.008 | 0.009 | 0.742 | 0.721 | FALSE | 4 | 0.021 | 27209 | 0.659 | 4 | 0.001 | 462400 | 4.00E-08 | Lung cancer |
| Nap during day | rs77154532 | G | A | G | A | -0.007 | -0.014 | 0.360 | 0.376 | FALSE | 3 | 0.018 | 27209 | 0.460 | 3 | 0.001 | 462400 | 1.60E-08 | Lung cancer |
| Nap during day | rs7752899 | T | C | T | C | 0.008 | 0.015 | 0.443 | 0.440 | FALSE | 6 | 0.018 | 27209 | 0.421 | 6 | 0.001 | 462400 | 5.70E-12 | Lung cancer |
| Nap during day | rs7814873 | T | C | T | C | -0.007 | -0.010 | 0.616 | 0.601 | FALSE | 8 | 0.019 | 27209 | 0.602 | 8 | 0.001 | 462400 | 3.30E-08 | Lung cancer |
| Nap during day | rs785145 | G | T | G | T | 0.007 | 0.022 | 0.432 | 0.438 | FALSE | 6 | 0.018 | 27209 | 0.227 | 6 | 0.001 | 462400 | 1.10E-08 | Lung cancer |
| Nap during day | rs8050478 | A | G | A | G | -0.008 | -0.022 | 0.501 | 0.485 | FALSE | 16 | 0.018 | 27209 | 0.231 | 16 | 0.001 | 462400 | 4.60E-10 | Lung cancer |
| Nap during day | rs903678 | A | G | A | G | 0.014 | 0.011 | 0.337 | 0.324 | FALSE | 1 | 0.019 | 27209 | 0.573 | 1 | 0.001 | 462400 | 4.60E-26 | Lung cancer |
| Nap during day | rs908442 | T | A | T | A | -0.010 | -0.016 | 0.408 | 0.399 | FALSE | 2 | 0.018 | 27209 | 0.387 | 2 | 0.001 | 462400 | 4.70E-15 | Lung cancer |
| Nap during day | rs910187 | A | G | A | G | -0.007 | -0.001 | 0.373 | 0.374 | FALSE | 20 | 0.019 | 27209 | 0.972 | 20 | 0.001 | 462400 | 1.10E-08 | Lung cancer |
| Nap during day | rs9467772 | T | A | T | A | -0.009 | 0.020 | 0.200 | 0.188 | FALSE | 6 | 0.023 | 27209 | 0.382 | 6 | 0.002 | 462400 | 2.40E-09 | Lung cancer |
| Nap during day | rs962247 | A | G | A | G | -0.008 | 0.005 | 0.476 | 0.478 | FALSE | 18 | 0.018 | 27209 | 0.773 | 18 | 0.001 | 462400 | 3.60E-11 | Lung cancer |
| Nap during day | rs971415 | G | A | G | A | -0.011 | 0.029 | 0.123 | 0.144 | FALSE | 9 | 0.026 | 27209 | 0.260 | 9 | 0.002 | 462400 | 6.30E-09 | Lung cancer |
| Nap during day | rs9965170 | A | G | A | G | -0.014 | -0.017 | 0.424 | 0.427 | FALSE | 18 | 0.018 | 27209 | 0.343 | 18 | 0.001 | 462400 | 2.30E-30 | Lung cancer |
| Nap during day | rs9998136 | G | C | G | C | 0.009 | -0.003 | 0.748 | 0.756 | FALSE | 4 | 0.021 | 27209 | 0.872 | 4 | 0.001 | 462400 | 2.20E-11 | Lung cancer |
| Chronotype | rs10058356 | T | C | T | C | 0.013 | 0.020 | 0.698 | 0.692 | FALSE | 5 | 0.019 | 27209 | 0.310 | 5 | 0.002 | 413343 | 3.70E-09 | Lung cancer |
| Chronotype | rs10118767 | T | C | T | C | 0.014 | -0.016 | 0.198 | 0.227 | FALSE | 9 | 0.021 | 27209 | 0.464 | 9 | 0.003 | 413343 | 4.50E-08 | Lung cancer |
| Chronotype | rs10149448 | G | A | G | A | 0.012 | -0.034 | 0.396 | 0.408 | FALSE | 14 | 0.018 | 27209 | 0.078 | 14 | 0.002 | 413343 | 3.00E-08 | Lung cancer |
| Chronotype | rs10175975 | T | C | T | C | -0.018 | -0.041 | 0.182 | 0.189 | FALSE | 2 | 0.022 | 27209 | 0.083 | 2 | 0.003 | 413343 | 4.40E-12 | Lung cancer |
| Chronotype | rs10280205 | C | T | C | T | 0.013 | 0.040 | 0.309 | 0.322 | FALSE | 7 | 0.020 | 27209 | 0.038 | 7 | 0.002 | 413343 | 1.50E-09 | Lung cancer |
| Chronotype | rs10402849 | T | C | T | C | -0.015 | 0.008 | 0.202 | 0.203 | FALSE | 19 | 0.022 | 27209 | 0.732 | 19 | 0.003 | 413343 | 2.30E-09 | Lung cancer |
| Chronotype | rs10461917 | C | T | C | T | 0.012 | 0.013 | 0.690 | 0.705 | FALSE | 5 | 0.020 | 27209 | 0.535 | 5 | 0.002 | 413343 | 2.50E-08 | Lung cancer |
| Chronotype | rs1056322 | G | C | G | C | 0.013 | 0.015 | 0.321 | 0.326 | FALSE | 22 | 0.019 | 27209 | 0.445 | 22 | 0.002 | 413343 | 6.60E-09 | Lung cancer |
| Chronotype | rs10737452 | T | C | T | C | 0.014 | -0.028 | 0.624 | 0.598 | FALSE | 1 | 0.019 | 27209 | 0.131 | 1 | 0.002 | 413343 | 7.90E-12 | Lung cancer |
| Chronotype | rs10742179 | G | A | G | A | 0.013 | 0.047 | 0.739 | 0.730 | FALSE | 11 | 0.019 | 27209 | 0.022 | 11 | 0.002 | 413343 | 2.60E-08 | Lung cancer |
| Chronotype | rs10954933 | G | A | G | A | -0.015 | -0.006 | 0.428 | 0.423 | FALSE | 8 | 0.018 | 27209 | 0.762 | 8 | 0.002 | 413343 | 6.00E-14 | Lung cancer |
| Chronotype | rs10988239 | T | C | T | C | 0.013 | -0.017 | 0.512 | 0.496 | FALSE | 9 | 0.019 | 27209 | 0.372 | 9 | 0.002 | 413343 | 4.70E-10 | Lung cancer |
| Chronotype | rs11032362 | A | G | A | G | -0.026 | 0.007 | 0.091 | 0.095 | FALSE | 11 | 0.030 | 27209 | 0.826 | 11 | 0.004 | 413343 | 1.20E-13 | Lung cancer |
| Chronotype | rs111761918 | A | G | A | G | 0.022 | -0.085 | 0.068 | 0.075 | FALSE | 1 | 0.031 | 27209 | 0.014 | 1 | 0.004 | 413343 | 4.80E-08 | Lung cancer |
| Chronotype | rs11183201 | C | T | C | T | -0.013 | -0.035 | 0.508 | 0.509 | FALSE | 12 | 0.019 | 27209 | 0.056 | 12 | 0.002 | 413343 | 2.40E-10 | Lung cancer |
| Chronotype | rs112555644 | T | C | T | C | -0.029 | 0.017 | 0.068 | 0.072 | FALSE | 3 | 0.042 | 27209 | 0.694 | 3 | 0.004 | 413343 | 1.30E-11 | Lung cancer |
| Chronotype | rs113171806 | C | T | C | T | 0.018 | 0.005 | 0.114 | 0.097 | FALSE | 9 | 0.039 | 27209 | 0.902 | 9 | 0.003 | 413343 | 1.40E-08 | Lung cancer |
| Chronotype | rs1135946 | C | T | C | T | 0.017 | 0.008 | 0.232 | 0.244 | FALSE | 4 | 0.022 | 27209 | 0.708 | 4 | 0.002 | 413343 | 6.90E-13 | Lung cancer |
| Chronotype | rs114870822 | A | G | A | G | -0.049 | 0.118 | 0.013 | 0.022 | FALSE | 2 | 0.070 | 27209 | 0.074 | 2 | 0.009 | 413343 | 4.60E-08 | Lung cancer |
| Chronotype | rs11587758 | A | G | A | G | -0.019 | 0.027 | 0.396 | 0.397 | FALSE | 1 | 0.019 | 27209 | 0.151 | 1 | 0.002 | 413343 | 1.20E-19 | Lung cancer |
| Chronotype | rs116131939 | T | C | T | C | -0.023 | 0.013 | 0.084 | 0.073 | FALSE | 2 | 0.035 | 27209 | 0.721 | 2 | 0.004 | 413343 | 8.00E-10 | Lung cancer |
| Chronotype | rs11714441 | T | C | T | C | 0.012 | 0.003 | 0.401 | 0.405 | FALSE | 3 | 0.019 | 27209 | 0.858 | 3 | 0.002 | 413343 | 1.30E-08 | Lung cancer |
| Chronotype | rs12117333 | A | G | A | G | 0.024 | -0.005 | 0.077 | 0.080 | FALSE | 1 | 0.032 | 27209 | 0.886 | 1 | 0.004 | 413343 | 2.40E-10 | Lung cancer |
| Chronotype | rs12140153 | T | G | T | G | 0.027 | 0.030 | 0.094 | 0.082 | FALSE | 1 | 0.037 | 27209 | 0.424 | 1 | 0.004 | 413343 | 4.20E-14 | Lung cancer |
| Chronotype | rs12249410 | T | G | T | G | 0.019 | -0.076 | 0.110 | 0.104 | FALSE | 10 | 0.027 | 27209 | 0.011 | 10 | 0.003 | 413343 | 1.00E-08 | Lung cancer |
| Chronotype | rs12377175 | C | A | C | A | 0.016 | 0.002 | 0.229 | 0.227 | FALSE | 9 | 0.021 | 27209 | 0.924 | 9 | 0.002 | 413343 | 4.80E-11 | Lung cancer |
| Chronotype | rs12432176 | A | C | A | C | -0.012 | -0.018 | 0.380 | 0.373 | FALSE | 14 | 0.019 | 27209 | 0.365 | 14 | 0.002 | 413343 | 9.70E-09 | Lung cancer |
| Chronotype | rs12462111 | T | C | T | C | 0.013 | 0.007 | 0.465 | 0.432 | FALSE | 19 | 0.019 | 27209 | 0.718 | 19 | 0.002 | 413343 | 1.90E-10 | Lung cancer |
| Chronotype | rs12525312 | C | T | C | T | 0.012 | -0.014 | 0.551 | 0.584 | FALSE | 6 | 0.018 | 27209 | 0.440 | 6 | 0.002 | 413343 | 1.90E-09 | Lung cancer |
| Chronotype | rs12713014 | G | A | G | A | 0.026 | 0.065 | 0.059 | 0.074 | FALSE | 2 | 0.036 | 27209 | 0.062 | 2 | 0.004 | 413343 | 1.90E-09 | Lung cancer |
| Chronotype | rs12811046 | G | A | G | A | 0.013 | -0.021 | 0.445 | 0.447 | FALSE | 12 | 0.018 | 27209 | 0.242 | 12 | 0.002 | 413343 | 2.40E-10 | Lung cancer |
| Chronotype | rs12927162 | G | A | G | A | 0.021 | -0.028 | 0.277 | 0.284 | FALSE | 16 | 0.020 | 27209 | 0.190 | 16 | 0.002 | 413343 | 3.20E-20 | Lung cancer |
| Chronotype | rs12965577 | G | A | G | A | 0.016 | 0.035 | 0.335 | 0.341 | FALSE | 18 | 0.019 | 27209 | 0.063 | 18 | 0.002 | 413343 | 2.00E-13 | Lung cancer |
| Chronotype | rs12969848 | T | C | T | C | -0.016 | 0.008 | 0.529 | 0.534 | FALSE | 18 | 0.018 | 27209 | 0.644 | 18 | 0.002 | 413343 | 9.60E-16 | Lung cancer |
| Chronotype | rs12971913 | A | G | A | G | 0.013 | -0.012 | 0.448 | 0.454 | FALSE | 19 | 0.018 | 27209 | 0.500 | 19 | 0.002 | 413343 | 6.60E-10 | Lung cancer |
| Chronotype | rs13011556 | G | C | G | C | -0.017 | -0.012 | 0.239 | 0.243 | FALSE | 2 | 0.022 | 27209 | 0.594 | 2 | 0.002 | 413343 | 1.80E-12 | Lung cancer |
| Chronotype | rs13059636 | G | A | G | A | -0.013 | 0.026 | 0.470 | 0.453 | FALSE | 3 | 0.018 | 27209 | 0.147 | 3 | 0.002 | 413343 | 6.00E-11 | Lung cancer |
| Chronotype | rs13258797 | A | G | A | G | -0.016 | 0.018 | 0.169 | 0.193 | FALSE | 8 | 0.023 | 27209 | 0.451 | 8 | 0.003 | 413343 | 5.00E-09 | Lung cancer |
| Chronotype | rs13316611 | T | G | T | G | -0.014 | 0.037 | 0.256 | 0.255 | FALSE | 3 | 0.021 | 27209 | 0.076 | 3 | 0.002 | 413343 | 6.80E-09 | Lung cancer |
| Chronotype | rs138964083 | T | C | T | C | -0.026 | 0.005 | 0.059 | 0.055 | FALSE | 2 | 0.040 | 27209 | 0.912 | 2 | 0.004 | 413343 | 3.10E-09 | Lung cancer |
| Chronotype | rs139911 | T | C | T | C | 0.018 | 0.025 | 0.576 | 0.567 | FALSE | 22 | 0.018 | 27209 | 0.184 | 22 | 0.002 | 413343 | 2.00E-17 | Lung cancer |
| Chronotype | rs1421085 | C | T | C | T | -0.021 | -0.008 | 0.404 | 0.418 | FALSE | 16 | 0.018 | 27209 | 0.670 | 16 | 0.002 | 413343 | 8.90E-24 | Lung cancer |
| Chronotype | rs1439319 | C | G | C | G | 0.012 | 0.019 | 0.645 | 0.668 | FALSE | 15 | 0.019 | 27209 | 0.333 | 15 | 0.002 | 413343 | 1.40E-08 | Lung cancer |
| Chronotype | rs147762489 | T | C | T | C | 0.016 | -0.036 | 0.249 | 0.243 | FALSE | 5 | 0.020 | 27209 | 0.091 | 5 | 0.002 | 413343 | 5.20E-12 | Lung cancer |
| Chronotype | rs17161045 | C | T | C | T | 0.015 | 0.018 | 0.370 | 0.363 | FALSE | 7 | 0.019 | 27209 | 0.344 | 7 | 0.002 | 413343 | 4.70E-13 | Lung cancer |
| Chronotype | rs17448682 | T | C | T | C | -0.016 | 0.016 | 0.232 | 0.234 | FALSE | 1 | 0.022 | 27209 | 0.478 | 1 | 0.002 | 413343 | 7.90E-12 | Lung cancer |
| Chronotype | rs17517 | A | G | A | G | 0.012 | 0.020 | 0.512 | 0.505 | FALSE | 13 | 0.018 | 27209 | 0.272 | 13 | 0.002 | 413343 | 1.30E-08 | Lung cancer |
| Chronotype | rs17575798 | A | G | A | G | 0.016 | -0.028 | 0.193 | 0.191 | FALSE | 1 | 0.022 | 27209 | 0.227 | 1 | 0.003 | 413343 | 4.00E-10 | Lung cancer |
| Chronotype | rs17604349 | A | G | A | G | 0.022 | 0.003 | 0.180 | 0.195 | FALSE | 16 | 0.023 | 27209 | 0.880 | 16 | 0.003 | 413343 | 1.40E-16 | Lung cancer |
| Chronotype | rs17716502 | T | C | T | C | -0.019 | 0.064 | 0.204 | 0.185 | FALSE | 8 | 0.025 | 27209 | 0.007 | 8 | 0.003 | 413343 | 1.20E-13 | Lung cancer |
| Chronotype | rs17786957 | C | G | C | G | 0.016 | -0.022 | 0.165 | 0.165 | FALSE | 3 | 0.024 | 27209 | 0.368 | 3 | 0.003 | 413343 | 2.90E-09 | Lung cancer |
| Chronotype | rs1800828 | G | C | G | C | 0.013 | 0.037 | 0.253 | 0.238 | FALSE | 3 | 0.022 | 27209 | 0.082 | 3 | 0.002 | 413343 | 2.00E-08 | Lung cancer |
| Chronotype | rs1874493 | G | A | G | A | 0.012 | -0.018 | 0.680 | 0.668 | FALSE | 19 | 0.019 | 27209 | 0.358 | 19 | 0.002 | 413343 | 1.80E-08 | Lung cancer |
| Chronotype | rs1914772 | A | T | A | T | 0.021 | -0.036 | 0.895 | 0.899 | FALSE | 11 | 0.030 | 27209 | 0.229 | 11 | 0.003 | 413343 | 3.30E-10 | Lung cancer |
| Chronotype | rs1927719 | A | T | A | T | -0.014 | -0.046 | 0.766 | 0.765 | FALSE | 13 | 0.022 | 27209 | 0.030 | 13 | 0.002 | 413343 | 6.70E-09 | Lung cancer |
| Chronotype | rs197273 | G | A | G | A | 0.012 | 0.017 | 0.530 | 0.534 | FALSE | 2 | 0.018 | 27209 | 0.345 | 2 | 0.002 | 413343 | 3.10E-09 | Lung cancer |
| Chronotype | rs1983891 | T | C | T | C | 0.013 | 0.016 | 0.276 | 0.281 | FALSE | 6 | 0.020 | 27209 | 0.425 | 6 | 0.002 | 413343 | 8.50E-09 | Lung cancer |
| Chronotype | rs1996399 | A | G | A | G | -0.012 | -0.007 | 0.301 | 0.294 | FALSE | 7 | 0.019 | 27209 | 0.723 | 7 | 0.002 | 413343 | 4.30E-08 | Lung cancer |
| Chronotype | rs202157 | T | C | T | C | 0.019 | -0.026 | 0.701 | 0.699 | FALSE | 7 | 0.020 | 27209 | 0.184 | 7 | 0.002 | 413343 | 1.70E-17 | Lung cancer |
| Chronotype | rs2072727 | C | T | C | T | 0.012 | -0.007 | 0.564 | 0.571 | FALSE | 20 | 0.018 | 27209 | 0.718 | 20 | 0.002 | 413343 | 1.40E-08 | Lung cancer |
| Chronotype | rs2077432 | T | C | T | C | -0.013 | 0.002 | 0.270 | 0.271 | FALSE | 11 | 0.020 | 27209 | 0.932 | 11 | 0.002 | 413343 | 5.70E-09 | Lung cancer |
| Chronotype | rs2239626 | C | T | C | T | -0.014 | -0.022 | 0.306 | 0.297 | FALSE | 3 | 0.019 | 27209 | 0.270 | 3 | 0.002 | 413343 | 3.30E-10 | Lung cancer |
| Chronotype | rs225298 | G | T | G | T | 0.015 | -0.026 | 0.831 | 0.818 | FALSE | 17 | 0.023 | 27209 | 0.264 | 17 | 0.003 | 413343 | 2.20E-08 | Lung cancer |
| Chronotype | rs2291589 | G | T | G | T | 0.015 | -0.001 | 0.377 | 0.355 | FALSE | 9 | 0.019 | 27209 | 0.939 | 9 | 0.002 | 413343 | 4.50E-13 | Lung cancer |
| Chronotype | rs2364972 | G | A | G | A | -0.013 | -0.017 | 0.463 | 0.443 | FALSE | 17 | 0.018 | 27209 | 0.364 | 17 | 0.002 | 413343 | 3.70E-10 | Lung cancer |
| Chronotype | rs2518022 | C | T | C | T | 0.031 | 0.037 | 0.914 | 0.910 | FALSE | 17 | 0.029 | 27209 | 0.234 | 17 | 0.004 | 413343 | 2.60E-17 | Lung cancer |
| Chronotype | rs2653349 | G | A | G | A | 0.028 | -0.041 | 0.787 | 0.800 | FALSE | 6 | 0.023 | 27209 | 0.072 | 6 | 0.002 | 413343 | 2.90E-30 | Lung cancer |
| Chronotype | rs2701524 | C | T | C | T | 0.011 | 0.002 | 0.414 | 0.399 | FALSE | 15 | 0.018 | 27209 | 0.925 | 15 | 0.002 | 413343 | 3.20E-08 | Lung cancer |
| Chronotype | rs2706762 | T | C | T | C | 0.018 | -0.007 | 0.150 | 0.147 | FALSE | 2 | 0.025 | 27209 | 0.792 | 2 | 0.003 | 413343 | 2.30E-10 | Lung cancer |
| Chronotype | rs2712056 | T | C | T | C | -0.016 | 0.002 | 0.186 | 0.184 | FALSE | 2 | 0.023 | 27209 | 0.933 | 2 | 0.003 | 413343 | 1.80E-09 | Lung cancer |
| Chronotype | rs2762088 | G | T | G | T | -0.014 | -0.024 | 0.761 | 0.770 | FALSE | 13 | 0.021 | 27209 | 0.264 | 13 | 0.002 | 413343 | 1.60E-09 | Lung cancer |
| Chronotype | rs28380327 | T | A | T | A | 0.015 | 0.001 | 0.370 | 0.354 | FALSE | 2 | 0.019 | 27209 | 0.950 | 2 | 0.002 | 413343 | 2.50E-13 | Lung cancer |
| Chronotype | rs2850298 | G | A | G | A | -0.019 | -0.009 | 0.699 | 0.668 | FALSE | 2 | 0.020 | 27209 | 0.644 | 2 | 0.002 | 413343 | 3.10E-17 | Lung cancer |
| Chronotype | rs286808 | C | T | C | T | 0.012 | 0.005 | 0.525 | 0.547 | FALSE | 5 | 0.018 | 27209 | 0.788 | 5 | 0.002 | 413343 | 9.40E-09 | Lung cancer |
| Chronotype | rs2881955 | T | C | T | C | -0.014 | -0.016 | 0.278 | 0.274 | FALSE | 6 | 0.020 | 27209 | 0.442 | 6 | 0.002 | 413343 | 2.00E-09 | Lung cancer |
| Chronotype | rs2893787 | A | G | A | G | 0.015 | -0.031 | 0.744 | 0.737 | FALSE | 10 | 0.021 | 27209 | 0.138 | 10 | 0.002 | 413343 | 1.90E-10 | Lung cancer |
| Chronotype | rs2971970 | G | T | G | T | 0.015 | 0.014 | 0.782 | 0.788 | FALSE | 7 | 0.021 | 27209 | 0.535 | 7 | 0.002 | 413343 | 8.00E-10 | Lung cancer |
| Chronotype | rs3100052 | G | A | G | A | 0.012 | -0.008 | 0.613 | 0.601 | FALSE | 8 | 0.018 | 27209 | 0.683 | 8 | 0.002 | 413343 | 5.20E-09 | Lung cancer |
| Chronotype | rs3168135 | A | G | A | G | 0.015 | 0.003 | 0.240 | 0.242 | FALSE | 11 | 0.020 | 27209 | 0.900 | 11 | 0.002 | 413343 | 3.20E-10 | Lung cancer |
| Chronotype | rs34244172 | T | C | T | C | 0.012 | 0.001 | 0.292 | 0.289 | FALSE | 3 | 0.020 | 27209 | 0.977 | 3 | 0.002 | 413343 | 2.90E-08 | Lung cancer |
| Chronotype | rs35101255 | G | A | G | A | 0.024 | -0.019 | 0.079 | 0.090 | FALSE | 6 | 0.031 | 27209 | 0.556 | 6 | 0.004 | 413343 | 8.50E-11 | Lung cancer |
| Chronotype | rs35524253 | A | G | A | G | -0.012 | -0.007 | 0.356 | 0.355 | FALSE | 8 | 0.019 | 27209 | 0.696 | 8 | 0.002 | 413343 | 1.30E-08 | Lung cancer |
| Chronotype | rs3729986 | T | C | T | C | -0.019 | -0.007 | 0.102 | 0.092 | FALSE | 11 | 0.032 | 27209 | 0.829 | 11 | 0.003 | 413343 | 1.70E-08 | Lung cancer |
| Chronotype | rs3760185 | T | C | T | C | 0.019 | -0.024 | 0.248 | 0.238 | FALSE | 17 | 0.022 | 27209 | 0.311 | 17 | 0.002 | 413343 | 2.20E-15 | Lung cancer |
| Chronotype | rs3808964 | T | G | T | G | -0.012 | 0.016 | 0.633 | 0.634 | FALSE | 10 | 0.018 | 27209 | 0.403 | 10 | 0.002 | 413343 | 1.00E-08 | Lung cancer |
| Chronotype | rs4141920 | A | G | A | G | 0.012 | 0.001 | 0.455 | 0.462 | FALSE | 11 | 0.018 | 27209 | 0.975 | 11 | 0.002 | 413343 | 1.20E-08 | Lung cancer |
| Chronotype | rs4237555 | T | C | T | C | -0.012 | -0.010 | 0.528 | 0.537 | FALSE | 11 | 0.019 | 27209 | 0.592 | 11 | 0.002 | 413343 | 9.40E-09 | Lung cancer |
| Chronotype | rs4241964 | G | T | G | T | -0.015 | -0.009 | 0.476 | 0.474 | FALSE | 4 | 0.018 | 27209 | 0.634 | 4 | 0.002 | 413343 | 1.00E-13 | Lung cancer |
| Chronotype | rs4321976 | C | T | C | T | 0.017 | -0.004 | 0.221 | 0.209 | FALSE | 8 | 0.022 | 27209 | 0.865 | 8 | 0.002 | 413343 | 2.30E-12 | Lung cancer |
| Chronotype | rs4484214 | G | A | G | A | 0.014 | 0.030 | 0.315 | 0.325 | FALSE | 3 | 0.020 | 27209 | 0.126 | 3 | 0.002 | 413343 | 4.80E-10 | Lung cancer |
| Chronotype | rs4518438 | C | T | C | T | -0.015 | -0.019 | 0.510 | 0.521 | FALSE | 5 | 0.018 | 27209 | 0.299 | 5 | 0.002 | 413343 | 6.30E-14 | Lung cancer |
| Chronotype | rs4549082 | C | T | C | T | -0.015 | 0.001 | 0.484 | 0.494 | FALSE | 2 | 0.018 | 27209 | 0.941 | 2 | 0.002 | 413343 | 5.50E-14 | Lung cancer |
| Chronotype | rs4595586 | T | A | T | A | 0.023 | 0.028 | 0.507 | 0.515 | FALSE | 12 | 0.017 | 27209 | 0.129 | 12 | 0.002 | 413343 | 6.40E-29 | Lung cancer |
| Chronotype | rs4671379 | C | T | C | T | -0.012 | 0.018 | 0.586 | 0.587 | FALSE | 2 | 0.018 | 27209 | 0.340 | 2 | 0.002 | 413343 | 2.00E-09 | Lung cancer |
| Chronotype | rs4784655 | C | G | C | G | 0.016 | 0.028 | 0.322 | 0.336 | FALSE | 16 | 0.020 | 27209 | 0.155 | 16 | 0.002 | 413343 | 7.40E-14 | Lung cancer |
| Chronotype | rs4886947 | A | G | A | G | 0.012 | 0.007 | 0.660 | 0.653 | FALSE | 15 | 0.022 | 27209 | 0.755 | 15 | 0.002 | 413343 | 3.50E-08 | Lung cancer |
| Chronotype | rs4936291 | G | A | G | A | -0.014 | -0.012 | 0.389 | 0.374 | FALSE | 11 | 0.020 | 27209 | 0.545 | 11 | 0.002 | 413343 | 2.10E-10 | Lung cancer |
| Chronotype | rs4949980 | G | A | G | A | -0.023 | 0.016 | 0.067 | 0.073 | FALSE | 1 | 0.036 | 27209 | 0.671 | 1 | 0.004 | 413343 | 2.30E-08 | Lung cancer |
| Chronotype | rs509476 | C | T | C | T | 0.088 | -0.103 | 0.970 | 0.975 | FALSE | 1 | 0.068 | 27209 | 0.115 | 1 | 0.006 | 413343 | 8.10E-49 | Lung cancer |
| Chronotype | rs56076457 | T | C | T | C | -0.012 | 0.027 | 0.529 | 0.549 | FALSE | 18 | 0.017 | 27209 | 0.137 | 18 | 0.002 | 413343 | 8.90E-09 | Lung cancer |
| Chronotype | rs56372114 | T | C | T | C | 0.012 | -0.010 | 0.384 | 0.396 | FALSE | 1 | 0.018 | 27209 | 0.595 | 1 | 0.002 | 413343 | 6.30E-09 | Lung cancer |
| Chronotype | rs57435966 | T | C | T | C | 0.041 | -0.056 | 0.086 | 0.083 | FALSE | 2 | 0.030 | 27209 | 0.088 | 2 | 0.004 | 413343 | 8.50E-30 | Lung cancer |
| Chronotype | rs57994353 | C | T | C | T | -0.013 | 0.023 | 0.299 | 0.296 | FALSE | 9 | 0.020 | 27209 | 0.244 | 9 | 0.002 | 413343 | 7.80E-09 | Lung cancer |
| Chronotype | rs610590 | G | C | G | C | -0.015 | 0.026 | 0.212 | 0.205 | FALSE | 6 | 0.023 | 27209 | 0.246 | 6 | 0.002 | 413343 | 4.60E-09 | Lung cancer |
| Chronotype | rs6131942 | G | A | G | A | -0.014 | 0.026 | 0.580 | 0.594 | FALSE | 20 | 0.018 | 27209 | 0.152 | 20 | 0.002 | 413343 | 4.50E-12 | Lung cancer |
| Chronotype | rs61773390 | T | G | T | G | -0.026 | 0.010 | 0.195 | 0.188 | FALSE | 1 | 0.023 | 27209 | 0.660 | 1 | 0.003 | 413343 | 1.30E-23 | Lung cancer |
| Chronotype | rs62082401 | G | C | G | C | -0.020 | 0.022 | 0.191 | 0.207 | FALSE | 18 | 0.023 | 27209 | 0.328 | 18 | 0.003 | 413343 | 9.60E-15 | Lung cancer |
| Chronotype | rs62182115 | T | C | T | C | 0.013 | -0.001 | 0.342 | 0.336 | FALSE | 2 | 0.019 | 27209 | 0.938 | 2 | 0.002 | 413343 | 5.50E-10 | Lung cancer |
| Chronotype | rs62553781 | T | C | T | C | 0.038 | 0.012 | 0.035 | 0.029 | FALSE | 9 | 0.054 | 27209 | 0.836 | 9 | 0.006 | 413343 | 1.10E-11 | Lung cancer |
| Chronotype | rs6441169 | A | G | A | G | -0.016 | -0.045 | 0.856 | 0.869 | FALSE | 3 | 0.027 | 27209 | 0.086 | 3 | 0.003 | 413343 | 2.30E-08 | Lung cancer |
| Chronotype | rs6442446 | G | A | G | A | 0.013 | 0.027 | 0.709 | 0.705 | FALSE | 3 | 0.019 | 27209 | 0.172 | 3 | 0.002 | 413343 | 7.20E-09 | Lung cancer |
| Chronotype | rs6504758 | G | A | G | A | -0.012 | -0.006 | 0.536 | 0.563 | FALSE | 17 | 0.019 | 27209 | 0.745 | 17 | 0.002 | 413343 | 1.20E-09 | Lung cancer |
| Chronotype | rs6601686 | T | A | T | A | 0.014 | -0.007 | 0.410 | 0.405 | FALSE | 8 | 0.018 | 27209 | 0.723 | 8 | 0.002 | 413343 | 4.60E-11 | Lung cancer |
| Chronotype | rs6658041 | A | G | A | G | -0.012 | 0.000 | 0.599 | 0.611 | FALSE | 1 | 0.018 | 27209 | 0.981 | 1 | 0.002 | 413343 | 7.50E-09 | Lung cancer |
| Chronotype | rs66710942 | T | C | T | C | -0.013 | -0.012 | 0.592 | 0.566 | FALSE | 3 | 0.019 | 27209 | 0.523 | 3 | 0.002 | 413343 | 1.60E-10 | Lung cancer |
| Chronotype | rs6718119 | G | A | G | A | -0.012 | 0.005 | 0.377 | 0.372 | FALSE | 2 | 0.019 | 27209 | 0.800 | 2 | 0.002 | 413343 | 1.10E-08 | Lung cancer |
| Chronotype | rs67988891 | G | C | G | C | -0.019 | -0.015 | 0.319 | 0.317 | FALSE | 5 | 0.019 | 27209 | 0.454 | 5 | 0.002 | 413343 | 1.10E-17 | Lung cancer |
| Chronotype | rs6967481 | T | C | T | C | -0.016 | 0.033 | 0.497 | 0.496 | FALSE | 7 | 0.017 | 27209 | 0.072 | 7 | 0.002 | 413343 | 3.00E-14 | Lung cancer |
| Chronotype | rs698015 | T | C | T | C | -0.013 | 0.021 | 0.647 | 0.637 | FALSE | 14 | 0.018 | 27209 | 0.256 | 14 | 0.002 | 413343 | 1.20E-09 | Lung cancer |
| Chronotype | rs7148842 | T | C | T | C | 0.012 | 0.004 | 0.386 | 0.386 | FALSE | 14 | 0.019 | 27209 | 0.834 | 14 | 0.002 | 413343 | 7.40E-09 | Lung cancer |
| Chronotype | rs72632979 | G | A | G | A | 0.017 | 0.018 | 0.172 | 0.158 | FALSE | 11 | 0.026 | 27209 | 0.479 | 11 | 0.003 | 413343 | 9.20E-10 | Lung cancer |
| Chronotype | rs72720396 | G | A | G | A | -0.021 | -0.042 | 0.230 | 0.211 | FALSE | 1 | 0.022 | 27209 | 0.065 | 1 | 0.002 | 413343 | 1.30E-17 | Lung cancer |
| Chronotype | rs7304278 | G | A | G | A | -0.015 | 0.019 | 0.724 | 0.696 | FALSE | 12 | 0.019 | 27209 | 0.336 | 12 | 0.002 | 413343 | 1.10E-10 | Lung cancer |
| Chronotype | rs74097630 | T | G | T | G | 0.018 | 0.047 | 0.141 | 0.145 | FALSE | 12 | 0.026 | 27209 | 0.068 | 12 | 0.003 | 413343 | 4.80E-10 | Lung cancer |
| Chronotype | rs74357745 | G | A | G | A | 0.020 | 0.047 | 0.121 | 0.125 | FALSE | 11 | 0.028 | 27209 | 0.088 | 11 | 0.003 | 413343 | 9.60E-11 | Lung cancer |
| Chronotype | rs7547493 | G | A | G | A | -0.028 | 0.037 | 0.177 | 0.174 | FALSE | 1 | 0.024 | 27209 | 0.124 | 1 | 0.003 | 413343 | 3.70E-26 | Lung cancer |
| Chronotype | rs76223855 | C | T | C | T | -0.080 | -0.086 | 0.011 | 0.013 | FALSE | 6 | 0.069 | 27209 | 0.290 | 6 | 0.010 | 413343 | 2.70E-16 | Lung cancer |
| Chronotype | rs7626349 | C | T | C | T | -0.013 | 0.008 | 0.706 | 0.695 | FALSE | 3 | 0.019 | 27209 | 0.666 | 3 | 0.002 | 413343 | 1.90E-08 | Lung cancer |
| Chronotype | rs7652260 | G | C | G | C | 0.016 | 0.000 | 0.162 | 0.160 | FALSE | 3 | 0.026 | 27209 | 0.991 | 3 | 0.003 | 413343 | 8.10E-09 | Lung cancer |
| Chronotype | rs769066 | C | T | C | T | -0.017 | -0.003 | 0.184 | 0.190 | FALSE | 8 | 0.023 | 27209 | 0.911 | 8 | 0.003 | 413343 | 3.20E-10 | Lung cancer |
| Chronotype | rs7783012 | A | G | A | G | -0.012 | 0.017 | 0.591 | 0.559 | FALSE | 7 | 0.018 | 27209 | 0.355 | 7 | 0.002 | 413343 | 5.20E-09 | Lung cancer |
| Chronotype | rs78095690 | C | T | C | T | -0.011 | -0.009 | 0.437 | 0.441 | FALSE | 20 | 0.018 | 27209 | 0.634 | 20 | 0.002 | 413343 | 2.40E-08 | Lung cancer |
| Chronotype | rs7959983 | C | T | C | T | -0.014 | 0.023 | 0.405 | 0.408 | FALSE | 12 | 0.019 | 27209 | 0.214 | 12 | 0.002 | 413343 | 9.00E-12 | Lung cancer |
| Chronotype | rs80097534 | T | G | T | G | 0.022 | -0.074 | 0.098 | 0.115 | FALSE | 12 | 0.027 | 27209 | 0.016 | 12 | 0.003 | 413343 | 1.00E-10 | Lung cancer |
| Chronotype | rs812925 | G | C | G | C | -0.015 | -0.013 | 0.352 | 0.365 | FALSE | 2 | 0.018 | 27209 | 0.492 | 2 | 0.002 | 413343 | 1.10E-12 | Lung cancer |
| Chronotype | rs848552 | G | C | G | C | -0.012 | -0.011 | 0.528 | 0.540 | FALSE | 2 | 0.018 | 27209 | 0.544 | 2 | 0.002 | 413343 | 1.40E-09 | Lung cancer |
| Chronotype | rs9291813 | C | T | C | T | -0.013 | -0.009 | 0.761 | 0.770 | FALSE | 5 | 0.021 | 27209 | 0.671 | 5 | 0.002 | 413343 | 1.90E-08 | Lung cancer |
| Chronotype | rs9348050 | C | T | C | T | 0.012 | 0.020 | 0.511 | 0.522 | FALSE | 6 | 0.017 | 27209 | 0.269 | 6 | 0.002 | 413343 | 9.50E-10 | Lung cancer |
| Chronotype | rs9395520 | T | C | T | C | -0.017 | -0.001 | 0.304 | 0.320 | FALSE | 6 | 0.019 | 27209 | 0.956 | 6 | 0.002 | 413343 | 4.10E-15 | Lung cancer |
| Chronotype | rs9476310 | T | C | T | C | -0.013 | -0.032 | 0.511 | 0.513 | FALSE | 6 | 0.019 | 27209 | 0.087 | 6 | 0.002 | 413343 | 6.50E-10 | Lung cancer |
| Chronotype | rs9573971 | G | A | G | A | 0.053 | 0.062 | 0.034 | 0.032 | FALSE | 13 | 0.051 | 27209 | 0.219 | 13 | 0.006 | 413343 | 9.70E-21 | Lung cancer |
| Chronotype | rs9597250 | A | C | A | C | 0.016 | 0.038 | 0.189 | 0.191 | FALSE | 13 | 0.023 | 27209 | 0.102 | 13 | 0.003 | 413343 | 6.50E-10 | Lung cancer |
| Chronotype | rs9795439 | G | A | G | A | 0.015 | 0.016 | 0.804 | 0.794 | FALSE | 11 | 0.023 | 27209 | 0.510 | 11 | 0.003 | 413343 | 7.90E-09 | Lung cancer |
| Chronotype | rs9831488 | G | A | G | A | -0.014 | -0.009 | 0.353 | 0.344 | FALSE | 3 | 0.019 | 27209 | 0.660 | 3 | 0.002 | 413343 | 1.10E-10 | Lung cancer |
| Chronotype | rs9932577 | A | C | A | C | 0.013 | 0.004 | 0.506 | 0.516 | FALSE | 16 | 0.019 | 27209 | 0.850 | 16 | 0.002 | 413343 | 7.40E-10 | Lung cancer |
| Chronotype | rs9962650 | G | C | G | C | -0.014 | -0.002 | 0.423 | 0.413 | FALSE | 18 | 0.018 | 27209 | 0.923 | 18 | 0.002 | 413343 | 1.50E-11 | Lung cancer |
| Chronotype | rs9964420 | A | C | A | C | 0.021 | 0.016 | 0.303 | 0.283 | FALSE | 18 | 0.020 | 27209 | 0.418 | 18 | 0.002 | 413343 | 4.40E-22 | Lung cancer |
| Getting up in morning | rs1017168 | C | A | C | A | 0.010 | 0.001 | 0.644 | 0.640 | FALSE | 12 | 0.019 | 27209 | 0.948 | 12 | 0.002 | 461658 | 3.60E-10 | Lung cancer |
| Getting up in morning | rs10175975 | T | C | T | C | 0.012 | -0.041 | 0.182 | 0.189 | FALSE | 2 | 0.022 | 27209 | 0.083 | 2 | 0.002 | 461658 | 3.70E-09 | Lung cancer |
| Getting up in morning | rs10455248 | T | C | T | C | -0.010 | 0.025 | 0.282 | 0.287 | FALSE | 6 | 0.020 | 27209 | 0.200 | 6 | 0.002 | 461658 | 2.60E-08 | Lung cancer |
| Getting up in morning | rs10462020 | G | T | G | T | 0.017 | 0.011 | 0.196 | 0.188 | FALSE | 1 | 0.023 | 27209 | 0.637 | 1 | 0.002 | 461658 | 1.90E-16 | Lung cancer |
| Getting up in morning | rs10518446 | C | G | C | G | 0.020 | 0.057 | 0.163 | 0.161 | FALSE | 1 | 0.025 | 27209 | 0.020 | 1 | 0.002 | 461658 | 9.80E-20 | Lung cancer |
| Getting up in morning | rs10779704 | C | A | C | A | 0.010 | -0.009 | 0.625 | 0.617 | FALSE | 1 | 0.019 | 27209 | 0.652 | 1 | 0.002 | 461658 | 9.90E-09 | Lung cancer |
| Getting up in morning | rs11075924 | A | C | A | C | -0.010 | -0.006 | 0.496 | 0.493 | FALSE | 16 | 0.018 | 27209 | 0.726 | 16 | 0.002 | 461658 | 5.00E-10 | Lung cancer |
| Getting up in morning | rs112613078 | G | A | G | A | 0.012 | -0.021 | 0.191 | 0.190 | FALSE | 7 | 0.025 | 27209 | 0.430 | 7 | 0.002 | 461658 | 7.50E-10 | Lung cancer |
| Getting up in morning | rs113232113 | T | A | T | A | -0.014 | 0.037 | 0.117 | 0.125 | FALSE | 19 | 0.028 | 27209 | 0.189 | 19 | 0.002 | 461658 | 2.30E-08 | Lung cancer |
| Getting up in morning | rs114443104 | A | G | A | G | 0.019 | -0.015 | 0.058 | 0.051 | FALSE | 2 | 0.043 | 27209 | 0.734 | 2 | 0.003 | 461658 | 2.70E-08 | Lung cancer |
| Getting up in morning | rs11629621 | G | C | G | C | -0.012 | 0.037 | 0.577 | 0.577 | FALSE | 15 | 0.017 | 27209 | 0.046 | 15 | 0.002 | 461658 | 2.10E-13 | Lung cancer |
| Getting up in morning | rs11669535 | C | T | C | T | 0.011 | -0.001 | 0.217 | 0.176 | FALSE | 19 | 0.025 | 27209 | 0.977 | 19 | 0.002 | 461658 | 4.60E-08 | Lung cancer |
| Getting up in morning | rs11672103 | T | C | T | C | -0.009 | 0.003 | 0.553 | 0.539 | FALSE | 19 | 0.018 | 27209 | 0.880 | 19 | 0.002 | 461658 | 6.70E-09 | Lung cancer |
| Getting up in morning | rs12044778 | A | G | A | G | -0.012 | -0.027 | 0.180 | 0.179 | FALSE | 1 | 0.023 | 27209 | 0.248 | 1 | 0.002 | 461658 | 2.20E-08 | Lung cancer |
| Getting up in morning | rs12227309 | T | C | T | C | 0.012 | -0.011 | 0.247 | 0.252 | FALSE | 12 | 0.020 | 27209 | 0.614 | 12 | 0.002 | 461658 | 1.50E-10 | Lung cancer |
| Getting up in morning | rs1223149 | C | T | C | T | 0.014 | -0.019 | 0.804 | 0.809 | FALSE | 2 | 0.023 | 27209 | 0.417 | 2 | 0.002 | 461658 | 1.30E-12 | Lung cancer |
| Getting up in morning | rs12326675 | G | C | G | C | 0.012 | 0.020 | 0.193 | 0.204 | FALSE | 18 | 0.025 | 27209 | 0.422 | 18 | 0.002 | 461658 | 4.20E-08 | Lung cancer |
| Getting up in morning | rs12523700 | A | T | A | T | 0.014 | 0.017 | 0.118 | 0.097 | FALSE | 6 | 0.033 | 27209 | 0.613 | 6 | 0.003 | 461658 | 8.40E-09 | Lung cancer |
| Getting up in morning | rs12601968 | T | G | T | G | -0.011 | 0.010 | 0.327 | 0.308 | FALSE | 17 | 0.019 | 27209 | 0.606 | 17 | 0.002 | 461658 | 8.90E-11 | Lung cancer |
| Getting up in morning | rs12736689 | C | T | C | T | 0.053 | 0.098 | 0.030 | 0.026 | FALSE | 1 | 0.065 | 27209 | 0.115 | 1 | 0.005 | 461658 | 3.70E-29 | Lung cancer |
| Getting up in morning | rs12752290 | C | T | C | T | 0.012 | 0.029 | 0.442 | 0.450 | FALSE | 1 | 0.019 | 27209 | 0.129 | 1 | 0.002 | 461658 | 5.70E-13 | Lung cancer |
| Getting up in morning | rs12969848 | T | C | T | C | 0.010 | 0.008 | 0.529 | 0.534 | FALSE | 18 | 0.018 | 27209 | 0.644 | 18 | 0.002 | 461658 | 2.90E-09 | Lung cancer |
| Getting up in morning | rs13116306 | T | C | T | C | -0.010 | 0.022 | 0.421 | 0.421 | FALSE | 4 | 0.018 | 27209 | 0.233 | 4 | 0.002 | 461658 | 4.00E-09 | Lung cancer |
| Getting up in morning | rs13155750 | G | A | G | A | -0.012 | 0.038 | 0.234 | 0.227 | FALSE | 5 | 0.022 | 27209 | 0.079 | 5 | 0.002 | 461658 | 1.30E-10 | Lung cancer |
| Getting up in morning | rs133067 | T | C | T | C | 0.011 | 0.000 | 0.791 | 0.812 | FALSE | 22 | 0.023 | 27209 | 0.998 | 22 | 0.002 | 461658 | 1.50E-08 | Lung cancer |
| Getting up in morning | rs1333536 | T | C | T | C | -0.009 | 0.021 | 0.385 | 0.369 | FALSE | 13 | 0.019 | 27209 | 0.261 | 13 | 0.002 | 461658 | 2.30E-08 | Lung cancer |
| Getting up in morning | rs141391319 | A | G | A | G | 0.035 | 0.028 | 0.032 | 0.032 | FALSE | 1 | 0.052 | 27209 | 0.592 | 1 | 0.005 | 461658 | 1.60E-14 | Lung cancer |
| Getting up in morning | rs1420607 | A | G | A | G | 0.012 | 0.004 | 0.272 | 0.275 | FALSE | 16 | 0.020 | 27209 | 0.860 | 16 | 0.002 | 461658 | 1.30E-11 | Lung cancer |
| Getting up in morning | rs1421085 | C | T | C | T | 0.011 | -0.008 | 0.403 | 0.418 | FALSE | 16 | 0.018 | 27209 | 0.670 | 16 | 0.002 | 461658 | 1.60E-11 | Lung cancer |
| Getting up in morning | rs145831787 | T | C | T | C | -0.017 | -0.040 | 0.113 | 0.109 | FALSE | 3 | 0.027 | 27209 | 0.164 | 3 | 0.003 | 461658 | 1.60E-11 | Lung cancer |
| Getting up in morning | rs1470503 | T | C | T | C | -0.013 | 0.008 | 0.428 | 0.452 | FALSE | 2 | 0.018 | 27209 | 0.661 | 2 | 0.002 | 461658 | 1.90E-15 | Lung cancer |
| Getting up in morning | rs1606803 | T | C | T | C | 0.011 | 0.018 | 0.287 | 0.280 | FALSE | 2 | 0.020 | 27209 | 0.378 | 2 | 0.002 | 461658 | 9.50E-11 | Lung cancer |
| Getting up in morning | rs17112198 | G | A | G | A | 0.010 | 0.001 | 0.299 | 0.266 | FALSE | 14 | 0.020 | 27209 | 0.949 | 14 | 0.002 | 461658 | 5.30E-09 | Lung cancer |
| Getting up in morning | rs17152364 | G | A | G | A | -0.012 | 0.036 | 0.301 | 0.315 | FALSE | 11 | 0.020 | 27209 | 0.067 | 11 | 0.002 | 461658 | 1.40E-11 | Lung cancer |
| Getting up in morning | rs17464772 | A | G | A | G | 0.013 | 0.002 | 0.352 | 0.318 | FALSE | 12 | 0.019 | 27209 | 0.917 | 12 | 0.002 | 461658 | 6.90E-14 | Lung cancer |
| Getting up in morning | rs17716502 | T | C | T | C | 0.011 | 0.064 | 0.204 | 0.185 | FALSE | 8 | 0.025 | 27209 | 0.007 | 8 | 0.002 | 461658 | 4.10E-08 | Lung cancer |
| Getting up in morning | rs17766755 | A | G | A | G | -0.009 | 0.021 | 0.360 | 0.355 | FALSE | 14 | 0.019 | 27209 | 0.274 | 14 | 0.002 | 461658 | 1.30E-08 | Lung cancer |
| Getting up in morning | rs17777135 | A | G | A | G | 0.012 | 0.031 | 0.200 | 0.202 | FALSE | 2 | 0.024 | 27209 | 0.186 | 2 | 0.002 | 461658 | 1.10E-08 | Lung cancer |
| Getting up in morning | rs1854558 | A | G | A | G | -0.010 | -0.008 | 0.262 | 0.252 | FALSE | 9 | 0.020 | 27209 | 0.716 | 9 | 0.002 | 461658 | 7.90E-09 | Lung cancer |
| Getting up in morning | rs1914397 | A | T | A | T | 0.010 | -0.003 | 0.448 | 0.439 | FALSE | 7 | 0.018 | 27209 | 0.882 | 7 | 0.002 | 461658 | 1.10E-10 | Lung cancer |
| Getting up in morning | rs2044742 | A | G | A | G | -0.013 | -0.003 | 0.132 | 0.123 | FALSE | 8 | 0.027 | 27209 | 0.906 | 8 | 0.002 | 461658 | 1.30E-08 | Lung cancer |
| Getting up in morning | rs2360802 | T | A | T | A | 0.013 | 0.003 | 0.225 | 0.229 | FALSE | 8 | 0.021 | 27209 | 0.907 | 8 | 0.002 | 461658 | 2.10E-11 | Lung cancer |
| Getting up in morning | rs2653355 | A | C | A | C | -0.024 | -0.044 | 0.823 | 0.844 | FALSE | 6 | 0.026 | 27209 | 0.077 | 6 | 0.002 | 461658 | 1.60E-29 | Lung cancer |
| Getting up in morning | rs2971970 | G | T | G | T | -0.011 | 0.014 | 0.782 | 0.788 | FALSE | 7 | 0.021 | 27209 | 0.535 | 7 | 0.002 | 461658 | 3.20E-08 | Lung cancer |
| Getting up in morning | rs3125735 | T | C | T | C | -0.012 | 0.052 | 0.199 | 0.198 | FALSE | 13 | 0.023 | 27209 | 0.023 | 13 | 0.002 | 461658 | 1.90E-09 | Lung cancer |
| Getting up in morning | rs34757401 | G | A | G | A | 0.011 | -0.032 | 0.227 | 0.223 | FALSE | 14 | 0.021 | 27209 | 0.153 | 14 | 0.002 | 461658 | 1.80E-08 | Lung cancer |
| Getting up in morning | rs3760185 | T | C | T | C | -0.012 | -0.024 | 0.248 | 0.238 | FALSE | 17 | 0.022 | 27209 | 0.311 | 17 | 0.002 | 461658 | 1.10E-10 | Lung cancer |
| Getting up in morning | rs3766163 | C | T | C | T | 0.010 | 0.004 | 0.273 | 0.289 | FALSE | 1 | 0.020 | 27209 | 0.846 | 1 | 0.002 | 461658 | 6.70E-09 | Lung cancer |
| Getting up in morning | rs406952 | C | T | C | T | 0.011 | 0.006 | 0.376 | 0.372 | FALSE | 2 | 0.019 | 27209 | 0.758 | 2 | 0.002 | 461658 | 1.50E-11 | Lung cancer |
| Getting up in morning | rs4483990 | C | A | C | A | -0.018 | -0.007 | 0.157 | 0.153 | FALSE | 2 | 0.024 | 27209 | 0.771 | 2 | 0.002 | 461658 | 7.10E-17 | Lung cancer |
| Getting up in morning | rs45510091 | G | A | G | A | 0.020 | 0.008 | 0.055 | 0.048 | FALSE | 4 | 0.041 | 27209 | 0.855 | 4 | 0.004 | 461658 | 5.70E-09 | Lung cancer |
| Getting up in morning | rs4790352 | A | G | A | G | 0.018 | -0.017 | 0.918 | 0.920 | FALSE | 17 | 0.033 | 27209 | 0.607 | 17 | 0.003 | 461658 | 8.80E-10 | Lung cancer |
| Getting up in morning | rs4958317 | A | G | A | G | 0.015 | -0.014 | 0.289 | 0.273 | FALSE | 5 | 0.020 | 27209 | 0.483 | 5 | 0.002 | 461658 | 1.20E-16 | Lung cancer |
| Getting up in morning | rs6141724 | G | T | G | T | -0.010 | 0.027 | 0.370 | 0.395 | FALSE | 20 | 0.020 | 27209 | 0.169 | 20 | 0.002 | 461658 | 2.30E-09 | Lung cancer |
| Getting up in morning | rs61926781 | T | C | T | C | -0.019 | -0.052 | 0.059 | 0.051 | FALSE | 12 | 0.045 | 27209 | 0.293 | 12 | 0.003 | 461658 | 8.90E-09 | Lung cancer |
| Getting up in morning | rs620598 | G | A | G | A | 0.011 | 0.014 | 0.226 | 0.219 | FALSE | 6 | 0.022 | 27209 | 0.516 | 6 | 0.002 | 461658 | 7.40E-09 | Lung cancer |
| Getting up in morning | rs627685 | C | T | C | T | 0.010 | 0.025 | 0.304 | 0.303 | FALSE | 18 | 0.020 | 27209 | 0.213 | 18 | 0.002 | 461658 | 2.80E-09 | Lung cancer |
| Getting up in morning | rs6745423 | A | T | A | T | -0.013 | 0.008 | 0.722 | 0.728 | FALSE | 2 | 0.020 | 27209 | 0.695 | 2 | 0.002 | 461658 | 6.10E-13 | Lung cancer |
| Getting up in morning | rs7105482 | G | A | G | A | 0.009 | -0.004 | 0.395 | 0.422 | FALSE | 11 | 0.018 | 27209 | 0.812 | 11 | 0.002 | 461658 | 4.40E-08 | Lung cancer |
| Getting up in morning | rs7144028 | C | A | C | A | -0.009 | -0.038 | 0.499 | 0.504 | FALSE | 14 | 0.019 | 27209 | 0.038 | 14 | 0.002 | 461658 | 1.50E-08 | Lung cancer |
| Getting up in morning | rs7206027 | T | A | T | A | -0.010 | -0.028 | 0.386 | 0.396 | FALSE | 16 | 0.018 | 27209 | 0.134 | 16 | 0.002 | 461658 | 2.80E-09 | Lung cancer |
| Getting up in morning | rs72895663 | G | A | G | A | 0.011 | 0.039 | 0.231 | 0.202 | FALSE | 6 | 0.024 | 27209 | 0.095 | 6 | 0.002 | 461658 | 5.80E-09 | Lung cancer |
| Getting up in morning | rs73179222 | A | G | A | G | 0.010 | 0.002 | 0.372 | 0.359 | FALSE | 8 | 0.019 | 27209 | 0.933 | 8 | 0.002 | 461658 | 1.10E-09 | Lung cancer |
| Getting up in morning | rs73608603 | G | A | G | A | -0.013 | 0.045 | 0.131 | 0.140 | FALSE | 11 | 0.027 | 27209 | 0.090 | 11 | 0.002 | 461658 | 1.30E-08 | Lung cancer |
| Getting up in morning | rs74555583 | A | G | A | G | -0.017 | -0.017 | 0.085 | 0.096 | FALSE | 20 | 0.031 | 27209 | 0.600 | 20 | 0.003 | 461658 | 2.00E-09 | Lung cancer |
| Getting up in morning | rs77556405 | A | G | A | G | 0.016 | -0.034 | 0.172 | 0.171 | FALSE | 17 | 0.023 | 27209 | 0.168 | 17 | 0.002 | 461658 | 1.50E-14 | Lung cancer |
| Getting up in morning | rs77556698 | T | G | T | G | -0.012 | 0.005 | 0.216 | 0.208 | FALSE | 14 | 0.022 | 27209 | 0.826 | 14 | 0.002 | 461658 | 1.40E-09 | Lung cancer |
| Getting up in morning | rs7833021 | T | C | T | C | 0.011 | 0.022 | 0.773 | 0.774 | FALSE | 8 | 0.021 | 27209 | 0.322 | 8 | 0.002 | 461658 | 3.30E-09 | Lung cancer |
| Getting up in morning | rs7899208 | C | T | C | T | 0.014 | 0.001 | 0.874 | 0.860 | FALSE | 10 | 0.025 | 27209 | 0.971 | 10 | 0.002 | 461658 | 1.50E-08 | Lung cancer |
| Getting up in morning | rs9399613 | T | C | T | C | -0.011 | -0.020 | 0.289 | 0.296 | FALSE | 6 | 0.019 | 27209 | 0.307 | 6 | 0.002 | 461658 | 5.20E-10 | Lung cancer |
| Getting up in morning | rs9573982 | C | T | C | T | -0.021 | 0.019 | 0.051 | 0.050 | FALSE | 13 | 0.041 | 27209 | 0.643 | 13 | 0.004 | 461658 | 1.20E-08 | Lung cancer |
| Getting up in morning | rs9644465 | A | G | A | G | 0.012 | -0.049 | 0.212 | 0.203 | FALSE | 8 | 0.021 | 27209 | 0.028 | 8 | 0.002 | 461658 | 3.00E-09 | Lung cancer |
| Sleep duration | rs10510128 | A | G | A | G | 0.011 | -0.023 | 0.208 | 0.221 | FALSE | 10 | 0.021 | 27209 | 0.297 | 10 | 0.002 | 460099 | 7.70E-09 | Lung cancer |
| Sleep duration | rs11039216 | T | C | T | C | -0.010 | -0.007 | 0.533 | 0.484 | FALSE | 11 | 0.019 | 27209 | 0.710 | 11 | 0.002 | 460099 | 1.50E-10 | Lung cancer |
| Sleep duration | rs113021516 | C | G | C | G | 0.011 | -0.025 | 0.336 | 0.335 | FALSE | 3 | 0.018 | 27209 | 0.186 | 3 | 0.002 | 460099 | 1.30E-11 | Lung cancer |
| Sleep duration | rs113113059 | C | T | C | T | -0.011 | 0.014 | 0.220 | 0.215 | FALSE | 6 | 0.022 | 27209 | 0.530 | 6 | 0.002 | 460099 | 8.60E-09 | Lung cancer |
| Sleep duration | rs11621908 | T | C | T | C | -0.020 | -0.067 | 0.083 | 0.087 | FALSE | 14 | 0.031 | 27209 | 0.049 | 14 | 0.003 | 460099 | 1.10E-11 | Lung cancer |
| Sleep duration | rs11643715 | G | C | G | C | 0.011 | -0.014 | 0.293 | 0.289 | FALSE | 16 | 0.019 | 27209 | 0.474 | 16 | 0.002 | 460099 | 5.00E-10 | Lung cancer |
| Sleep duration | rs11650677 | A | G | A | G | 0.011 | 0.008 | 0.339 | 0.354 | FALSE | 17 | 0.019 | 27209 | 0.659 | 17 | 0.002 | 460099 | 3.80E-11 | Lung cancer |
| Sleep duration | rs11982852 | T | C | T | C | -0.012 | -0.016 | 0.244 | 0.242 | FALSE | 7 | 0.020 | 27209 | 0.442 | 7 | 0.002 | 460099 | 3.10E-10 | Lung cancer |
| Sleep duration | rs12518468 | C | T | C | T | -0.011 | 0.056 | 0.329 | 0.322 | FALSE | 5 | 0.020 | 27209 | 0.005 | 5 | 0.002 | 460099 | 4.10E-10 | Lung cancer |
| Sleep duration | rs12567114 | A | G | A | G | 0.012 | -0.011 | 0.276 | 0.273 | FALSE | 1 | 0.020 | 27209 | 0.579 | 1 | 0.002 | 460099 | 6.10E-12 | Lung cancer |
| Sleep duration | rs13107325 | T | C | T | C | -0.024 | 0.046 | 0.075 | 0.079 | FALSE | 4 | 0.036 | 27209 | 0.187 | 4 | 0.003 | 460099 | 1.40E-15 | Lung cancer |
| Sleep duration | rs1348047 | T | G | T | G | -0.013 | 0.050 | 0.267 | 0.256 | FALSE | 18 | 0.021 | 27209 | 0.016 | 18 | 0.002 | 460099 | 3.80E-12 | Lung cancer |
| Sleep duration | rs1463053 | A | G | A | G | 0.009 | -0.008 | 0.640 | 0.620 | FALSE | 1 | 0.019 | 27209 | 0.670 | 1 | 0.002 | 460099 | 2.40E-08 | Lung cancer |
| Sleep duration | rs151014368 | A | G | A | G | 0.011 | -0.030 | 0.207 | 0.222 | FALSE | 5 | 0.022 | 27209 | 0.187 | 5 | 0.002 | 460099 | 1.00E-08 | Lung cancer |
| Sleep duration | rs1517572 | C | A | C | A | 0.012 | -0.036 | 0.581 | 0.578 | FALSE | 11 | 0.019 | 27209 | 0.054 | 11 | 0.002 | 460099 | 6.50E-13 | Lung cancer |
| Sleep duration | rs1553132 | G | A | G | A | 0.011 | -0.038 | 0.259 | 0.248 | FALSE | 11 | 0.020 | 27209 | 0.068 | 11 | 0.002 | 460099 | 8.10E-09 | Lung cancer |
| Sleep duration | rs17391944 | G | T | G | T | 0.022 | 0.021 | 0.050 | 0.058 | FALSE | 9 | 0.039 | 27209 | 0.605 | 9 | 0.004 | 460099 | 4.40E-09 | Lung cancer |
| Sleep duration | rs174564 | G | A | G | A | 0.010 | -0.061 | 0.349 | 0.330 | FALSE | 11 | 0.018 | 27209 | 0.002 | 11 | 0.002 | 460099 | 6.30E-09 | Lung cancer |
| Sleep duration | rs1939455 | T | G | T | G | -0.016 | -0.018 | 0.120 | 0.111 | FALSE | 11 | 0.029 | 27209 | 0.547 | 11 | 0.003 | 460099 | 3.50E-10 | Lung cancer |
| Sleep duration | rs1972712 | C | T | C | T | 0.012 | -0.009 | 0.249 | 0.255 | FALSE | 2 | 0.021 | 27209 | 0.660 | 2 | 0.002 | 460099 | 1.70E-10 | Lung cancer |
| Sleep duration | rs2072727 | C | T | C | T | -0.009 | -0.007 | 0.565 | 0.571 | FALSE | 20 | 0.018 | 27209 | 0.718 | 20 | 0.002 | 460099 | 9.30E-09 | Lung cancer |
| Sleep duration | rs2079070 | G | C | G | C | -0.013 | 0.027 | 0.735 | 0.725 | FALSE | 7 | 0.020 | 27209 | 0.195 | 7 | 0.002 | 460099 | 1.20E-13 | Lung cancer |
| Sleep duration | rs2186122 | T | A | T | A | -0.011 | 0.032 | 0.560 | 0.527 | FALSE | 1 | 0.017 | 27209 | 0.083 | 1 | 0.002 | 460099 | 2.30E-11 | Lung cancer |
| Sleep duration | rs2192528 | G | A | G | A | -0.010 | 0.014 | 0.522 | 0.523 | FALSE | 4 | 0.018 | 27209 | 0.452 | 4 | 0.002 | 460099 | 9.10E-10 | Lung cancer |
| Sleep duration | rs2236295 | T | G | T | G | -0.009 | 0.017 | 0.403 | 0.385 | FALSE | 10 | 0.019 | 27209 | 0.366 | 10 | 0.002 | 460099 | 2.90E-08 | Lung cancer |
| Sleep duration | rs2279681 | G | C | G | C | 0.009 | -0.024 | 0.342 | 0.352 | FALSE | 1 | 0.018 | 27209 | 0.207 | 1 | 0.002 | 460099 | 3.40E-08 | Lung cancer |
| Sleep duration | rs2683630 | G | C | G | C | 0.015 | -0.001 | 0.629 | 0.594 | FALSE | 2 | 0.018 | 27209 | 0.950 | 2 | 0.002 | 460099 | 1.70E-19 | Lung cancer |
| Sleep duration | rs2734831 | G | T | G | T | -0.010 | -0.030 | 0.607 | 0.592 | FALSE | 11 | 0.019 | 27209 | 0.106 | 11 | 0.002 | 460099 | 2.20E-09 | Lung cancer |
| Sleep duration | rs2748809 | C | T | C | T | -0.009 | 0.003 | 0.429 | 0.413 | FALSE | 14 | 0.019 | 27209 | 0.859 | 14 | 0.002 | 460099 | 1.90E-08 | Lung cancer |
| Sleep duration | rs2839753 | C | T | C | T | -0.011 | 0.023 | 0.265 | 0.266 | FALSE | 4 | 0.021 | 27209 | 0.265 | 4 | 0.002 | 460099 | 4.40E-09 | Lung cancer |
| Sleep duration | rs2863957 | A | C | A | C | 0.029 | -0.001 | 0.221 | 0.226 | FALSE | 2 | 0.021 | 27209 | 0.962 | 2 | 0.002 | 460099 | 9.60E-51 | Lung cancer |
| Sleep duration | rs34354917 | A | C | A | C | -0.010 | -0.002 | 0.289 | 0.285 | FALSE | 12 | 0.023 | 27209 | 0.916 | 12 | 0.002 | 460099 | 1.40E-08 | Lung cancer |
| Sleep duration | rs34786000 | T | G | T | G | 0.011 | 0.002 | 0.553 | 0.531 | FALSE | 19 | 0.019 | 27209 | 0.923 | 19 | 0.002 | 460099 | 1.70E-11 | Lung cancer |
| Sleep duration | rs35126035 | C | A | C | A | -0.009 | 0.016 | 0.558 | 0.530 | FALSE | 19 | 0.019 | 27209 | 0.416 | 19 | 0.002 | 460099 | 2.20E-08 | Lung cancer |
| Sleep duration | rs35662245 | A | T | A | T | 0.010 | -0.015 | 0.339 | 0.346 | FALSE | 2 | 0.018 | 27209 | 0.437 | 2 | 0.002 | 460099 | 1.90E-09 | Lung cancer |
| Sleep duration | rs365663 | G | A | G | A | -0.009 | 0.009 | 0.455 | 0.476 | FALSE | 5 | 0.019 | 27209 | 0.638 | 5 | 0.002 | 460099 | 8.10E-09 | Lung cancer |
| Sleep duration | rs374153 | T | C | T | C | -0.013 | 0.056 | 0.843 | 0.844 | FALSE | 2 | 0.023 | 27209 | 0.024 | 2 | 0.002 | 460099 | 2.50E-09 | Lung cancer |
| Sleep duration | rs4767550 | G | A | G | A | 0.011 | 0.033 | 0.413 | 0.405 | FALSE | 12 | 0.019 | 27209 | 0.080 | 12 | 0.002 | 460099 | 2.70E-11 | Lung cancer |
| Sleep duration | rs55658675 | T | C | T | C | -0.010 | -0.035 | 0.353 | 0.338 | FALSE | 14 | 0.018 | 27209 | 0.072 | 14 | 0.002 | 460099 | 7.10E-09 | Lung cancer |
| Sleep duration | rs56367859 | G | A | G | A | 0.012 | 0.008 | 0.398 | 0.413 | FALSE | 16 | 0.018 | 27209 | 0.659 | 16 | 0.002 | 460099 | 1.20E-12 | Lung cancer |
| Sleep duration | rs62444917 | C | A | C | A | 0.013 | -0.052 | 0.222 | 0.244 | FALSE | 7 | 0.020 | 27209 | 0.018 | 7 | 0.002 | 460099 | 1.70E-11 | Lung cancer |
| Sleep duration | rs6561715 | A | T | A | T | 0.010 | -0.018 | 0.631 | 0.635 | FALSE | 13 | 0.019 | 27209 | 0.335 | 13 | 0.002 | 460099 | 3.90E-09 | Lung cancer |
| Sleep duration | rs6681755 | A | G | A | G | 0.012 | -0.049 | 0.200 | 0.194 | FALSE | 1 | 0.022 | 27209 | 0.035 | 1 | 0.002 | 460099 | 9.00E-09 | Lung cancer |
| Sleep duration | rs6783516 | T | G | T | G | -0.010 | -0.016 | 0.584 | 0.589 | FALSE | 3 | 0.019 | 27209 | 0.386 | 3 | 0.002 | 460099 | 1.60E-09 | Lung cancer |
| Sleep duration | rs6889592 | A | G | A | G | 0.012 | 0.019 | 0.333 | 0.331 | FALSE | 5 | 0.019 | 27209 | 0.322 | 5 | 0.002 | 460099 | 4.10E-12 | Lung cancer |
| Sleep duration | rs7016314 | C | T | C | T | 0.010 | -0.005 | 0.656 | 0.655 | FALSE | 8 | 0.019 | 27209 | 0.790 | 8 | 0.002 | 460099 | 3.10E-09 | Lung cancer |
| Sleep duration | rs7115856 | C | A | C | A | 0.011 | -0.034 | 0.461 | 0.460 | FALSE | 11 | 0.017 | 27209 | 0.059 | 11 | 0.002 | 460099 | 1.50E-11 | Lung cancer |
| Sleep duration | rs72771082 | G | A | G | A | 0.011 | 0.009 | 0.218 | 0.229 | FALSE | 16 | 0.021 | 27209 | 0.692 | 16 | 0.002 | 460099 | 1.40E-08 | Lung cancer |
| Sleep duration | rs72831782 | A | T | A | T | -0.010 | 0.032 | 0.269 | 0.255 | FALSE | 2 | 0.024 | 27209 | 0.174 | 2 | 0.002 | 460099 | 3.40E-08 | Lung cancer |
| Sleep duration | rs7517981 | C | T | C | T | -0.010 | 0.035 | 0.601 | 0.581 | FALSE | 1 | 0.018 | 27209 | 0.063 | 1 | 0.002 | 460099 | 1.10E-09 | Lung cancer |
| Sleep duration | rs75539574 | C | A | C | A | 0.024 | -0.002 | 0.086 | 0.087 | FALSE | 2 | 0.033 | 27209 | 0.954 | 2 | 0.003 | 460099 | 1.80E-16 | Lung cancer |
| Sleep duration | rs76258078 | G | A | G | A | -0.022 | 0.035 | 0.050 | 0.039 | FALSE | 3 | 0.051 | 27209 | 0.503 | 3 | 0.004 | 460099 | 3.80E-09 | Lung cancer |
| Sleep duration | rs7644809 | C | T | C | T | -0.010 | 0.009 | 0.576 | 0.575 | FALSE | 3 | 0.018 | 27209 | 0.612 | 3 | 0.002 | 460099 | 4.10E-10 | Lung cancer |
| Sleep duration | rs7711696 | T | G | T | G | -0.010 | 0.054 | 0.305 | 0.294 | FALSE | 5 | 0.021 | 27209 | 0.006 | 5 | 0.002 | 460099 | 1.30E-08 | Lung cancer |
| Sleep duration | rs7740402 | G | T | G | T | -0.010 | -0.004 | 0.306 | 0.320 | FALSE | 6 | 0.019 | 27209 | 0.848 | 6 | 0.002 | 460099 | 4.10E-08 | Lung cancer |
| Sleep duration | rs7831557 | A | G | A | G | -0.011 | -0.034 | 0.517 | 0.521 | FALSE | 8 | 0.019 | 27209 | 0.062 | 8 | 0.002 | 460099 | 4.20E-11 | Lung cancer |
| Sleep duration | rs8038326 | G | A | G | A | -0.013 | 0.004 | 0.273 | 0.290 | FALSE | 15 | 0.020 | 27209 | 0.826 | 15 | 0.002 | 460099 | 8.40E-14 | Lung cancer |
| Sleep duration | rs8047587 | T | G | T | G | -0.011 | 0.006 | 0.440 | 0.445 | FALSE | 16 | 0.019 | 27209 | 0.742 | 16 | 0.002 | 460099 | 8.30E-12 | Lung cancer |
| Sleep duration | rs915416 | G | C | G | C | -0.013 | -0.042 | 0.709 | 0.689 | FALSE | 1 | 0.020 | 27209 | 0.032 | 1 | 0.002 | 460099 | 4.80E-13 | Lung cancer |
| Sleep duration | rs9302680 | A | G | A | G | 0.012 | 0.016 | 0.439 | 0.464 | FALSE | 16 | 0.018 | 27209 | 0.377 | 16 | 0.002 | 460099 | 7.60E-14 | Lung cancer |
| Sleep duration | rs9345234 | C | A | C | A | 0.009 | -0.018 | 0.578 | 0.557 | FALSE | 6 | 0.018 | 27209 | 0.333 | 6 | 0.002 | 460099 | 1.50E-08 | Lung cancer |
| Sleep duration | rs9382445 | C | T | C | T | -0.009 | 0.007 | 0.375 | 0.357 | FALSE | 6 | 0.019 | 27209 | 0.724 | 6 | 0.002 | 460099 | 8.90E-09 | Lung cancer |
| Sleep duration | rs9611007 | T | C | T | C | -0.014 | 0.036 | 0.142 | 0.152 | FALSE | 22 | 0.027 | 27209 | 0.173 | 22 | 0.002 | 460099 | 3.30E-09 | Lung cancer |
| Sleep duration | rs9810474 | T | C | T | C | -0.011 | 0.026 | 0.232 | 0.227 | FALSE | 3 | 0.022 | 27209 | 0.234 | 3 | 0.002 | 460099 | 3.90E-09 | Lung cancer |
| Sleep duration | rs9903898 | T | C | T | C | -0.009 | 0.014 | 0.489 | 0.486 | FALSE | 17 | 0.020 | 27209 | 0.496 | 17 | 0.002 | 460099 | 3.60E-09 | Lung cancer |
| Sleeplessness | rs10838708 | A | G | A | G | -0.009 | 0.003 | 0.459 | 0.478 | FALSE | 11 | 0.018 | 27209 | 0.892 | 11 | 0.002 | 462341 | 2.90E-10 | Lung cancer |
| Sleeplessness | rs11097861 | G | A | G | A | 0.010 | 0.008 | 0.716 | 0.704 | FALSE | 4 | 0.020 | 27209 | 0.678 | 4 | 0.002 | 462341 | 1.10E-09 | Lung cancer |
| Sleeplessness | rs11152363 | A | G | A | G | 0.016 | 0.037 | 0.186 | 0.189 | FALSE | 18 | 0.023 | 27209 | 0.106 | 18 | 0.002 | 462341 | 4.50E-16 | Lung cancer |
| Sleeplessness | rs113851554 | T | G | T | G | 0.047 | 0.023 | 0.057 | 0.064 | FALSE | 2 | 0.040 | 27209 | 0.565 | 2 | 0.003 | 462341 | 2.90E-45 | Lung cancer |
| Sleeplessness | rs11635495 | C | T | C | T | 0.009 | 0.018 | 0.512 | 0.505 | FALSE | 15 | 0.018 | 27209 | 0.328 | 15 | 0.001 | 462341 | 2.80E-10 | Lung cancer |
| Sleeplessness | rs11790060 | C | T | C | T | -0.010 | 0.033 | 0.331 | 0.345 | FALSE | 9 | 0.019 | 27209 | 0.084 | 9 | 0.002 | 462341 | 5.80E-11 | Lung cancer |
| Sleeplessness | rs12049261 | C | G | C | G | 0.011 | 0.005 | 0.293 | 0.288 | FALSE | 1 | 0.020 | 27209 | 0.825 | 1 | 0.002 | 462341 | 6.80E-12 | Lung cancer |
| Sleeplessness | rs12470989 | G | A | G | A | -0.010 | -0.022 | 0.204 | 0.198 | FALSE | 2 | 0.022 | 27209 | 0.330 | 2 | 0.002 | 462341 | 2.80E-08 | Lung cancer |
| Sleeplessness | rs1430205 | T | C | T | C | 0.009 | -0.002 | 0.462 | 0.477 | FALSE | 5 | 0.018 | 27209 | 0.925 | 5 | 0.001 | 462341 | 2.10E-10 | Lung cancer |
| Sleeplessness | rs1547630 | A | G | A | G | 0.009 | -0.021 | 0.652 | 0.630 | FALSE | 13 | 0.020 | 27209 | 0.290 | 13 | 0.002 | 462341 | 5.80E-09 | Lung cancer |
| Sleeplessness | rs1592757 | C | G | C | G | 0.010 | -0.034 | 0.356 | 0.361 | FALSE | 5 | 0.018 | 27209 | 0.077 | 5 | 0.002 | 462341 | 4.30E-11 | Lung cancer |
| Sleeplessness | rs17151854 | T | G | T | G | 0.013 | -0.003 | 0.152 | 0.148 | FALSE | 8 | 0.025 | 27209 | 0.915 | 8 | 0.002 | 462341 | 3.80E-10 | Lung cancer |
| Sleeplessness | rs17709610 | G | A | G | A | -0.010 | -0.029 | 0.298 | 0.269 | FALSE | 10 | 0.019 | 27209 | 0.150 | 10 | 0.002 | 462341 | 9.50E-10 | Lung cancer |
| Sleeplessness | rs1988337 | G | A | G | A | 0.008 | 0.022 | 0.552 | 0.554 | FALSE | 4 | 0.018 | 27209 | 0.242 | 4 | 0.001 | 462341 | 2.10E-08 | Lung cancer |
| Sleeplessness | rs2014830 | T | C | T | C | -0.012 | -0.012 | 0.304 | 0.325 | FALSE | 3 | 0.019 | 27209 | 0.526 | 3 | 0.002 | 462341 | 8.90E-13 | Lung cancer |
| Sleeplessness | rs2062113 | C | T | C | T | -0.010 | -0.031 | 0.568 | 0.563 | FALSE | 16 | 0.019 | 27209 | 0.102 | 16 | 0.002 | 462341 | 1.60E-10 | Lung cancer |
| Sleeplessness | rs224032 | A | G | A | G | 0.008 | -0.001 | 0.550 | 0.526 | FALSE | 10 | 0.018 | 27209 | 0.942 | 10 | 0.001 | 462341 | 1.80E-08 | Lung cancer |
| Sleeplessness | rs2297787 | A | T | A | T | -0.018 | 0.020 | 0.080 | 0.093 | FALSE | 10 | 0.031 | 27209 | 0.528 | 10 | 0.003 | 462341 | 9.60E-11 | Lung cancer |
| Sleeplessness | rs2604551 | G | T | G | T | -0.008 | -0.027 | 0.640 | 0.645 | FALSE | 4 | 0.019 | 27209 | 0.152 | 4 | 0.002 | 462341 | 4.70E-08 | Lung cancer |
| Sleeplessness | rs2644128 | G | C | G | C | 0.011 | 0.009 | 0.548 | 0.527 | FALSE | 1 | 0.018 | 27209 | 0.602 | 1 | 0.001 | 462341 | 1.00E-12 | Lung cancer |
| Sleeplessness | rs2803296 | C | G | C | G | -0.009 | -0.007 | 0.544 | 0.487 | FALSE | 1 | 0.019 | 27209 | 0.716 | 1 | 0.001 | 462341 | 7.30E-09 | Lung cancer |
| Sleeplessness | rs314280 | G | A | G | A | 0.010 | 0.018 | 0.547 | 0.555 | FALSE | 6 | 0.018 | 27209 | 0.331 | 6 | 0.001 | 462341 | 7.30E-11 | Lung cancer |
| Sleeplessness | rs324017 | C | A | C | A | -0.010 | -0.034 | 0.705 | 0.705 | FALSE | 12 | 0.020 | 27209 | 0.091 | 12 | 0.002 | 462341 | 1.40E-09 | Lung cancer |
| Sleeplessness | rs4572538 | T | C | T | C | -0.010 | -0.005 | 0.364 | 0.383 | FALSE | 2 | 0.019 | 27209 | 0.802 | 2 | 0.002 | 462341 | 7.70E-10 | Lung cancer |
| Sleeplessness | rs4577309 | G | A | G | A | -0.009 | -0.058 | 0.534 | 0.522 | FALSE | 2 | 0.019 | 27209 | 0.001 | 2 | 0.001 | 462341 | 1.00E-08 | Lung cancer |
| Sleeplessness | rs4886860 | C | G | C | G | -0.012 | -0.009 | 0.767 | 0.769 | FALSE | 15 | 0.022 | 27209 | 0.664 | 15 | 0.002 | 462341 | 1.80E-11 | Lung cancer |
| Sleeplessness | rs56093896 | A | C | A | C | -0.012 | 0.000 | 0.214 | 0.220 | FALSE | 2 | 0.022 | 27209 | 0.997 | 2 | 0.002 | 462341 | 7.70E-12 | Lung cancer |
| Sleeplessness | rs56330606 | G | A | G | A | 0.009 | 0.004 | 0.379 | 0.348 | FALSE | 19 | 0.019 | 27209 | 0.844 | 19 | 0.002 | 462341 | 1.20E-09 | Lung cancer |
| Sleeplessness | rs56365214 | A | C | A | C | -0.015 | 0.023 | 0.156 | 0.148 | FALSE | 2 | 0.026 | 27209 | 0.372 | 2 | 0.002 | 462341 | 5.60E-13 | Lung cancer |
| Sleeplessness | rs6561715 | A | T | A | T | -0.012 | -0.018 | 0.631 | 0.635 | FALSE | 13 | 0.019 | 27209 | 0.335 | 13 | 0.002 | 462341 | 4.80E-14 | Lung cancer |
| Sleeplessness | rs6690017 | G | T | G | T | -0.010 | -0.029 | 0.409 | 0.411 | FALSE | 1 | 0.018 | 27209 | 0.116 | 1 | 0.002 | 462341 | 1.10E-11 | Lung cancer |
| Sleeplessness | rs68094047 | T | C | T | C | 0.010 | -0.023 | 0.251 | 0.245 | FALSE | 12 | 0.021 | 27209 | 0.279 | 12 | 0.002 | 462341 | 1.70E-09 | Lung cancer |
| Sleeplessness | rs6975972 | G | A | G | A | -0.009 | 0.031 | 0.579 | 0.573 | FALSE | 7 | 0.018 | 27209 | 0.098 | 7 | 0.002 | 462341 | 2.00E-09 | Lung cancer |
| Sleeplessness | rs705219 | A | T | A | T | 0.013 | 0.074 | 0.887 | 0.892 | FALSE | 3 | 0.026 | 27209 | 0.010 | 3 | 0.002 | 462341 | 1.20E-08 | Lung cancer |
| Sleeplessness | rs72924721 | T | C | T | C | 0.016 | -0.010 | 0.073 | 0.071 | FALSE | 11 | 0.035 | 27209 | 0.787 | 11 | 0.003 | 462341 | 1.10E-08 | Lung cancer |
| Sleeplessness | rs7711696 | T | G | T | G | 0.011 | 0.054 | 0.305 | 0.294 | FALSE | 5 | 0.021 | 27209 | 0.006 | 5 | 0.002 | 462341 | 4.10E-12 | Lung cancer |
| Sleeplessness | rs8180817 | C | G | C | G | -0.010 | -0.018 | 0.431 | 0.439 | FALSE | 7 | 0.018 | 27209 | 0.326 | 7 | 0.002 | 462341 | 2.70E-11 | Lung cancer |
| Sleeplessness | rs931221 | A | T | A | T | 0.011 | -0.005 | 0.237 | 0.237 | FALSE | 12 | 0.021 | 27209 | 0.806 | 12 | 0.002 | 462341 | 1.30E-09 | Lung cancer |
| Sleeplessness | rs9570080 | C | T | C | T | -0.011 | -0.016 | 0.344 | 0.345 | FALSE | 13 | 0.019 | 27209 | 0.397 | 13 | 0.002 | 462341 | 1.60E-11 | Lung cancer |
| Sleeplessness | rs9845387 | A | C | A | C | -0.022 | -0.024 | 0.040 | 0.042 | FALSE | 3 | 0.043 | 27209 | 0.594 | 3 | 0.004 | 462341 | 7.10E-09 | Lung cancer |
| Sleeplessness | rs9894577 | A | G | A | G | 0.013 | 0.020 | 0.318 | 0.332 | FALSE | 17 | 0.019 | 27209 | 0.291 | 17 | 0.002 | 462341 | 1.30E-16 | Lung cancer |
| Sleeplessness | rs9906181 | G | A | G | A | -0.009 | 0.010 | 0.688 | 0.674 | FALSE | 17 | 0.024 | 27209 | 0.701 | 17 | 0.002 | 462341 | 2.40E-08 | Lung cancer |
| Getting up in morning | rs1017168 | C | A | C | A | 0.010 | -0.028 | 0.644 | 0.639 | FALSE | 12 | 0.030 | 18313 | 0.347 | 12 | 0.002 | 461658 | 3.60E-10 | Squamous cell cancer |
| Getting up in morning | rs10175975 | T | C | T | C | 0.012 | -0.073 | 0.182 | 0.189 | FALSE | 2 | 0.033 | 18313 | 0.044 | 2 | 0.002 | 461658 | 3.70E-09 | Squamous cell cancer |
| Getting up in morning | rs10455248 | T | C | T | C | -0.010 | 0.048 | 0.282 | 0.288 | FALSE | 6 | 0.031 | 18313 | 0.115 | 6 | 0.002 | 461658 | 2.60E-08 | Squamous cell cancer |
| Getting up in morning | rs10462020 | G | T | G | T | 0.017 | -0.005 | 0.196 | 0.189 | FALSE | 1 | 0.034 | 18313 | 0.890 | 1 | 0.002 | 461658 | 1.90E-16 | Squamous cell cancer |
| Getting up in morning | rs10518446 | C | G | C | G | 0.020 | 0.087 | 0.163 | 0.162 | FALSE | 1 | 0.040 | 18313 | 0.022 | 1 | 0.002 | 461658 | 9.80E-20 | Squamous cell cancer |
| Getting up in morning | rs10779704 | C | A | C | A | 0.010 | -0.030 | 0.625 | 0.617 | FALSE | 1 | 0.030 | 18313 | 0.320 | 1 | 0.002 | 461658 | 9.90E-09 | Squamous cell cancer |
| Getting up in morning | rs11075924 | A | C | A | C | -0.010 | 0.004 | 0.496 | 0.493 | FALSE | 16 | 0.027 | 18313 | 0.876 | 16 | 0.002 | 461658 | 5.00E-10 | Squamous cell cancer |
| Getting up in morning | rs112613078 | G | A | G | A | 0.012 | -0.047 | 0.191 | 0.190 | FALSE | 7 | 0.037 | 18313 | 0.252 | 7 | 0.002 | 461658 | 7.50E-10 | Squamous cell cancer |
| Getting up in morning | rs113232113 | T | A | T | A | -0.014 | 0.084 | 0.117 | 0.125 | FALSE | 19 | 0.045 | 18313 | 0.052 | 19 | 0.002 | 461658 | 2.30E-08 | Squamous cell cancer |
| Getting up in morning | rs114443104 | A | G | A | G | 0.019 | 0.032 | 0.058 | 0.051 | FALSE | 2 | 0.067 | 18313 | 0.649 | 2 | 0.003 | 461658 | 2.70E-08 | Squamous cell cancer |
| Getting up in morning | rs11629621 | G | C | G | C | -0.012 | 0.046 | 0.577 | 0.577 | FALSE | 15 | 0.026 | 18313 | 0.105 | 15 | 0.002 | 461658 | 2.10E-13 | Squamous cell cancer |
| Getting up in morning | rs11669535 | C | T | C | T | 0.011 | 0.029 | 0.217 | 0.177 | FALSE | 19 | 0.041 | 18313 | 0.487 | 19 | 0.002 | 461658 | 4.60E-08 | Squamous cell cancer |
| Getting up in morning | rs11672103 | T | C | T | C | -0.009 | 0.005 | 0.553 | 0.538 | FALSE | 19 | 0.028 | 18313 | 0.859 | 19 | 0.002 | 461658 | 6.70E-09 | Squamous cell cancer |
| Getting up in morning | rs12044778 | A | G | A | G | -0.012 | -0.017 | 0.180 | 0.179 | FALSE | 1 | 0.034 | 18313 | 0.634 | 1 | 0.002 | 461658 | 2.20E-08 | Squamous cell cancer |
| Getting up in morning | rs12227309 | T | C | T | C | 0.012 | 0.017 | 0.247 | 0.254 | FALSE | 12 | 0.032 | 18313 | 0.606 | 12 | 0.002 | 461658 | 1.50E-10 | Squamous cell cancer |
| Getting up in morning | rs1223149 | C | T | C | T | 0.014 | 0.011 | 0.804 | 0.810 | FALSE | 2 | 0.034 | 18313 | 0.767 | 2 | 0.002 | 461658 | 1.30E-12 | Squamous cell cancer |
| Getting up in morning | rs12326675 | G | C | G | C | 0.012 | -0.044 | 0.193 | 0.202 | FALSE | 18 | 0.036 | 18313 | 0.251 | 18 | 0.002 | 461658 | 4.20E-08 | Squamous cell cancer |
| Getting up in morning | rs12523700 | A | T | A | T | 0.014 | -0.008 | 0.118 | 0.096 | FALSE | 6 | 0.050 | 18313 | 0.873 | 6 | 0.003 | 461658 | 8.40E-09 | Squamous cell cancer |
| Getting up in morning | rs12601968 | T | G | T | G | -0.011 | 0.028 | 0.327 | 0.308 | FALSE | 17 | 0.031 | 18313 | 0.363 | 17 | 0.002 | 461658 | 8.90E-11 | Squamous cell cancer |
| Getting up in morning | rs12736689 | C | T | C | T | 0.053 | 0.120 | 0.030 | 0.026 | FALSE | 1 | 0.100 | 18313 | 0.215 | 1 | 0.005 | 461658 | 3.70E-29 | Squamous cell cancer |
| Getting up in morning | rs12752290 | C | T | C | T | 0.012 | 0.041 | 0.442 | 0.450 | FALSE | 1 | 0.030 | 18313 | 0.156 | 1 | 0.002 | 461658 | 5.70E-13 | Squamous cell cancer |
| Getting up in morning | rs12969848 | T | C | T | C | 0.010 | 0.023 | 0.529 | 0.535 | FALSE | 18 | 0.027 | 18313 | 0.426 | 18 | 0.002 | 461658 | 2.90E-09 | Squamous cell cancer |
| Getting up in morning | rs13116306 | T | C | T | C | -0.010 | 0.009 | 0.421 | 0.420 | FALSE | 4 | 0.028 | 18313 | 0.754 | 4 | 0.002 | 461658 | 4.00E-09 | Squamous cell cancer |
| Getting up in morning | rs13155750 | G | A | G | A | -0.012 | 0.006 | 0.234 | 0.226 | FALSE | 5 | 0.033 | 18313 | 0.851 | 5 | 0.002 | 461658 | 1.30E-10 | Squamous cell cancer |
| Getting up in morning | rs133067 | T | C | T | C | 0.011 | 0.008 | 0.791 | 0.813 | FALSE | 22 | 0.034 | 18313 | 0.828 | 22 | 0.002 | 461658 | 1.50E-08 | Squamous cell cancer |
| Getting up in morning | rs1333536 | T | C | T | C | -0.009 | 0.042 | 0.385 | 0.368 | FALSE | 13 | 0.029 | 18313 | 0.151 | 13 | 0.002 | 461658 | 2.30E-08 | Squamous cell cancer |
| Getting up in morning | rs141391319 | A | G | A | G | 0.035 | -0.013 | 0.032 | 0.032 | FALSE | 1 | 0.076 | 18313 | 0.874 | 1 | 0.005 | 461658 | 1.60E-14 | Squamous cell cancer |
| Getting up in morning | rs1420607 | A | G | A | G | 0.012 | 0.063 | 0.272 | 0.277 | FALSE | 16 | 0.033 | 18313 | 0.049 | 16 | 0.002 | 461658 | 1.30E-11 | Squamous cell cancer |
| Getting up in morning | rs1421085 | C | T | C | T | 0.011 | 0.013 | 0.403 | 0.420 | FALSE | 16 | 0.028 | 18313 | 0.646 | 16 | 0.002 | 461658 | 1.60E-11 | Squamous cell cancer |
| Getting up in morning | rs145831787 | T | C | T | C | -0.017 | -0.080 | 0.113 | 0.109 | FALSE | 3 | 0.040 | 18313 | 0.075 | 3 | 0.003 | 461658 | 1.60E-11 | Squamous cell cancer |
| Getting up in morning | rs1470503 | T | C | T | C | -0.013 | -0.010 | 0.428 | 0.451 | FALSE | 2 | 0.027 | 18313 | 0.732 | 2 | 0.002 | 461658 | 1.90E-15 | Squamous cell cancer |
| Getting up in morning | rs1606803 | T | C | T | C | 0.011 | -0.033 | 0.287 | 0.279 | FALSE | 2 | 0.030 | 18313 | 0.304 | 2 | 0.002 | 461658 | 9.50E-11 | Squamous cell cancer |
| Getting up in morning | rs17112198 | G | A | G | A | 0.010 | 0.030 | 0.299 | 0.266 | FALSE | 14 | 0.032 | 18313 | 0.354 | 14 | 0.002 | 461658 | 5.30E-09 | Squamous cell cancer |
| Getting up in morning | rs17152364 | G | A | G | A | -0.012 | 0.013 | 0.301 | 0.314 | FALSE | 11 | 0.030 | 18313 | 0.675 | 11 | 0.002 | 461658 | 1.40E-11 | Squamous cell cancer |
| Getting up in morning | rs17464772 | A | G | A | G | 0.013 | 0.003 | 0.352 | 0.317 | FALSE | 12 | 0.030 | 18313 | 0.921 | 12 | 0.002 | 461658 | 6.90E-14 | Squamous cell cancer |
| Getting up in morning | rs17716502 | T | C | T | C | 0.011 | 0.073 | 0.204 | 0.185 | FALSE | 8 | 0.038 | 18313 | 0.049 | 8 | 0.002 | 461658 | 4.10E-08 | Squamous cell cancer |
| Getting up in morning | rs17766755 | A | G | A | G | -0.009 | 0.031 | 0.360 | 0.354 | FALSE | 14 | 0.029 | 18313 | 0.287 | 14 | 0.002 | 461658 | 1.30E-08 | Squamous cell cancer |
| Getting up in morning | rs17777135 | A | G | A | G | 0.012 | 0.096 | 0.200 | 0.203 | FALSE | 2 | 0.039 | 18313 | 0.008 | 2 | 0.002 | 461658 | 1.10E-08 | Squamous cell cancer |
| Getting up in morning | rs1854558 | A | G | A | G | -0.010 | 0.014 | 0.262 | 0.254 | FALSE | 9 | 0.032 | 18313 | 0.673 | 9 | 0.002 | 461658 | 7.90E-09 | Squamous cell cancer |
| Getting up in morning | rs1914397 | A | T | A | T | 0.010 | -0.010 | 0.448 | 0.438 | FALSE | 7 | 0.027 | 18313 | 0.727 | 7 | 0.002 | 461658 | 1.10E-10 | Squamous cell cancer |
| Getting up in morning | rs2044742 | A | G | A | G | -0.013 | 0.012 | 0.132 | 0.123 | FALSE | 8 | 0.042 | 18313 | 0.775 | 8 | 0.002 | 461658 | 1.30E-08 | Squamous cell cancer |
| Getting up in morning | rs2360802 | T | A | T | A | 0.013 | 0.006 | 0.225 | 0.228 | FALSE | 8 | 0.032 | 18313 | 0.847 | 8 | 0.002 | 461658 | 2.10E-11 | Squamous cell cancer |
| Getting up in morning | rs2653355 | A | C | A | C | -0.024 | -0.038 | 0.823 | 0.844 | FALSE | 6 | 0.039 | 18313 | 0.327 | 6 | 0.002 | 461658 | 1.60E-29 | Squamous cell cancer |
| Getting up in morning | rs2971970 | G | T | G | T | -0.011 | 0.033 | 0.782 | 0.790 | FALSE | 7 | 0.032 | 18313 | 0.339 | 7 | 0.002 | 461658 | 3.20E-08 | Squamous cell cancer |
| Getting up in morning | rs3125735 | T | C | T | C | -0.012 | 0.051 | 0.199 | 0.198 | FALSE | 13 | 0.036 | 18313 | 0.151 | 13 | 0.002 | 461658 | 1.90E-09 | Squamous cell cancer |
| Getting up in morning | rs34757401 | G | A | G | A | 0.011 | -0.014 | 0.227 | 0.222 | FALSE | 14 | 0.033 | 18313 | 0.684 | 14 | 0.002 | 461658 | 1.80E-08 | Squamous cell cancer |
| Getting up in morning | rs3760185 | T | C | T | C | -0.012 | -0.026 | 0.248 | 0.238 | FALSE | 17 | 0.035 | 18313 | 0.486 | 17 | 0.002 | 461658 | 1.10E-10 | Squamous cell cancer |
| Getting up in morning | rs3766163 | C | T | C | T | 0.010 | 0.022 | 0.273 | 0.289 | FALSE | 1 | 0.030 | 18313 | 0.482 | 1 | 0.002 | 461658 | 6.70E-09 | Squamous cell cancer |
| Getting up in morning | rs406952 | C | T | C | T | 0.011 | 0.007 | 0.376 | 0.371 | FALSE | 2 | 0.029 | 18313 | 0.824 | 2 | 0.002 | 461658 | 1.50E-11 | Squamous cell cancer |
| Getting up in morning | rs4483990 | C | A | C | A | -0.018 | 0.021 | 0.157 | 0.155 | FALSE | 2 | 0.038 | 18313 | 0.593 | 2 | 0.002 | 461658 | 7.10E-17 | Squamous cell cancer |
| Getting up in morning | rs45510091 | G | A | G | A | 0.020 | 0.025 | 0.055 | 0.048 | FALSE | 4 | 0.064 | 18313 | 0.714 | 4 | 0.004 | 461658 | 5.70E-09 | Squamous cell cancer |
| Getting up in morning | rs4790352 | A | G | A | G | 0.018 | 0.019 | 0.918 | 0.921 | FALSE | 17 | 0.048 | 18313 | 0.711 | 17 | 0.003 | 461658 | 8.80E-10 | Squamous cell cancer |
| Getting up in morning | rs4958317 | A | G | A | G | 0.015 | -0.002 | 0.289 | 0.273 | FALSE | 5 | 0.031 | 18313 | 0.940 | 5 | 0.002 | 461658 | 1.20E-16 | Squamous cell cancer |
| Getting up in morning | rs6141724 | G | T | G | T | -0.010 | 0.023 | 0.370 | 0.396 | FALSE | 20 | 0.030 | 18313 | 0.456 | 20 | 0.002 | 461658 | 2.30E-09 | Squamous cell cancer |
| Getting up in morning | rs61926781 | T | C | T | C | -0.019 | 0.004 | 0.059 | 0.051 | FALSE | 12 | 0.072 | 18313 | 0.961 | 12 | 0.003 | 461658 | 8.90E-09 | Squamous cell cancer |
| Getting up in morning | rs620598 | G | A | G | A | 0.011 | 0.013 | 0.226 | 0.218 | FALSE | 6 | 0.034 | 18313 | 0.709 | 6 | 0.002 | 461658 | 7.40E-09 | Squamous cell cancer |
| Getting up in morning | rs627685 | C | T | C | T | 0.010 | -0.012 | 0.304 | 0.302 | FALSE | 18 | 0.029 | 18313 | 0.688 | 18 | 0.002 | 461658 | 2.80E-09 | Squamous cell cancer |
| Getting up in morning | rs6745423 | A | T | A | T | -0.013 | 0.014 | 0.722 | 0.728 | FALSE | 2 | 0.030 | 18313 | 0.647 | 2 | 0.002 | 461658 | 6.10E-13 | Squamous cell cancer |
| Getting up in morning | rs7105482 | G | A | G | A | 0.009 | 0.019 | 0.395 | 0.422 | FALSE | 11 | 0.028 | 18313 | 0.510 | 11 | 0.002 | 461658 | 4.40E-08 | Squamous cell cancer |
| Getting up in morning | rs7144028 | C | A | C | A | -0.009 | -0.029 | 0.499 | 0.506 | FALSE | 14 | 0.028 | 18313 | 0.297 | 14 | 0.002 | 461658 | 1.50E-08 | Squamous cell cancer |
| Getting up in morning | rs7206027 | T | A | T | A | -0.010 | -0.057 | 0.386 | 0.393 | FALSE | 16 | 0.026 | 18313 | 0.050 | 16 | 0.002 | 461658 | 2.80E-09 | Squamous cell cancer |
| Getting up in morning | rs72895663 | G | A | G | A | 0.011 | 0.008 | 0.231 | 0.200 | FALSE | 6 | 0.036 | 18313 | 0.830 | 6 | 0.002 | 461658 | 5.80E-09 | Squamous cell cancer |
| Getting up in morning | rs73179222 | A | G | A | G | 0.010 | 0.034 | 0.372 | 0.360 | FALSE | 8 | 0.029 | 18313 | 0.236 | 8 | 0.002 | 461658 | 1.10E-09 | Squamous cell cancer |
| Getting up in morning | rs73608603 | G | A | G | A | -0.013 | 0.043 | 0.131 | 0.140 | FALSE | 11 | 0.041 | 18313 | 0.295 | 11 | 0.002 | 461658 | 1.30E-08 | Squamous cell cancer |
| Getting up in morning | rs74555583 | A | G | A | G | -0.017 | 0.000 | 0.085 | 0.098 | FALSE | 20 | 0.048 | 18313 | 0.996 | 20 | 0.003 | 461658 | 2.00E-09 | Squamous cell cancer |
| Getting up in morning | rs77556405 | A | G | A | G | 0.016 | -0.038 | 0.172 | 0.171 | FALSE | 17 | 0.035 | 18313 | 0.314 | 17 | 0.002 | 461658 | 1.50E-14 | Squamous cell cancer |
| Getting up in morning | rs77556698 | T | G | T | G | -0.012 | 0.004 | 0.216 | 0.208 | FALSE | 14 | 0.034 | 18313 | 0.917 | 14 | 0.002 | 461658 | 1.40E-09 | Squamous cell cancer |
| Getting up in morning | rs7833021 | T | C | T | C | 0.011 | 0.023 | 0.773 | 0.774 | FALSE | 8 | 0.032 | 18313 | 0.500 | 8 | 0.002 | 461658 | 3.30E-09 | Squamous cell cancer |
| Getting up in morning | rs7899208 | C | T | C | T | 0.014 | -0.017 | 0.874 | 0.859 | FALSE | 10 | 0.039 | 18313 | 0.673 | 10 | 0.002 | 461658 | 1.50E-08 | Squamous cell cancer |
| Getting up in morning | rs9399613 | T | C | T | C | -0.011 | -0.017 | 0.289 | 0.296 | FALSE | 6 | 0.029 | 18313 | 0.586 | 6 | 0.002 | 461658 | 5.20E-10 | Squamous cell cancer |
| Getting up in morning | rs9573982 | C | T | C | T | -0.021 | 0.021 | 0.051 | 0.049 | FALSE | 13 | 0.062 | 18313 | 0.746 | 13 | 0.004 | 461658 | 1.20E-08 | Squamous cell cancer |
| Getting up in morning | rs9644465 | A | G | A | G | 0.012 | -0.059 | 0.212 | 0.203 | FALSE | 8 | 0.031 | 18313 | 0.085 | 8 | 0.002 | 461658 | 3.00E-09 | Squamous cell cancer |
| Chronotype | rs10058356 | T | C | T | C | 0.013 | -0.005 | 0.698 | 0.691 | FALSE | 5 | 0.029 | 18313 | 0.865 | 5 | 0.002 | 413343 | 3.70E-09 | Squamous cell cancer |
| Chronotype | rs10118767 | T | C | T | C | 0.014 | -0.029 | 0.198 | 0.226 | FALSE | 9 | 0.032 | 18313 | 0.393 | 9 | 0.003 | 413343 | 4.50E-08 | Squamous cell cancer |
| Chronotype | rs10149448 | G | A | G | A | 0.012 | -0.050 | 0.396 | 0.408 | FALSE | 14 | 0.027 | 18313 | 0.092 | 14 | 0.002 | 413343 | 3.00E-08 | Squamous cell cancer |
| Chronotype | rs10175975 | T | C | T | C | -0.018 | -0.073 | 0.182 | 0.189 | FALSE | 2 | 0.033 | 18313 | 0.044 | 2 | 0.003 | 413343 | 4.40E-12 | Squamous cell cancer |
| Chronotype | rs10280205 | C | T | C | T | 0.013 | 0.022 | 0.309 | 0.321 | FALSE | 7 | 0.030 | 18313 | 0.463 | 7 | 0.002 | 413343 | 1.50E-09 | Squamous cell cancer |
| Chronotype | rs10402849 | T | C | T | C | -0.015 | 0.023 | 0.202 | 0.204 | FALSE | 19 | 0.034 | 18313 | 0.505 | 19 | 0.003 | 413343 | 2.30E-09 | Squamous cell cancer |
| Chronotype | rs10461917 | C | T | C | T | 0.012 | 0.026 | 0.690 | 0.707 | FALSE | 5 | 0.030 | 18313 | 0.410 | 5 | 0.002 | 413343 | 2.50E-08 | Squamous cell cancer |
| Chronotype | rs1056322 | G | C | G | C | 0.013 | -0.017 | 0.321 | 0.325 | FALSE | 22 | 0.029 | 18313 | 0.579 | 22 | 0.002 | 413343 | 6.60E-09 | Squamous cell cancer |
| Chronotype | rs10737452 | T | C | T | C | 0.014 | -0.028 | 0.624 | 0.600 | FALSE | 1 | 0.029 | 18313 | 0.335 | 1 | 0.002 | 413343 | 7.90E-12 | Squamous cell cancer |
| Chronotype | rs10742179 | G | A | G | A | 0.013 | 0.072 | 0.739 | 0.730 | FALSE | 11 | 0.029 | 18313 | 0.024 | 11 | 0.002 | 413343 | 2.60E-08 | Squamous cell cancer |
| Chronotype | rs10954933 | G | A | G | A | -0.015 | -0.036 | 0.428 | 0.422 | FALSE | 8 | 0.026 | 18313 | 0.197 | 8 | 0.002 | 413343 | 6.00E-14 | Squamous cell cancer |
| Chronotype | rs10988239 | T | C | T | C | 0.013 | -0.005 | 0.512 | 0.496 | FALSE | 9 | 0.029 | 18313 | 0.870 | 9 | 0.002 | 413343 | 4.70E-10 | Squamous cell cancer |
| Chronotype | rs11032362 | A | G | A | G | -0.026 | -0.018 | 0.091 | 0.094 | FALSE | 11 | 0.046 | 18313 | 0.713 | 11 | 0.004 | 413343 | 1.20E-13 | Squamous cell cancer |
| Chronotype | rs111761918 | A | G | A | G | 0.022 | -0.081 | 0.068 | 0.075 | FALSE | 1 | 0.047 | 18313 | 0.129 | 1 | 0.004 | 413343 | 4.80E-08 | Squamous cell cancer |
| Chronotype | rs11183201 | C | T | C | T | -0.013 | -0.067 | 0.508 | 0.506 | FALSE | 12 | 0.030 | 18313 | 0.019 | 12 | 0.002 | 413343 | 2.40E-10 | Squamous cell cancer |
| Chronotype | rs112555644 | T | C | T | C | -0.029 | 0.170 | 0.068 | 0.074 | FALSE | 3 | 0.073 | 18313 | 0.010 | 3 | 0.004 | 413343 | 1.30E-11 | Squamous cell cancer |
| Chronotype | rs113171806 | C | T | C | T | 0.018 | 0.076 | 0.114 | 0.098 | FALSE | 9 | 0.064 | 18313 | 0.225 | 9 | 0.003 | 413343 | 1.40E-08 | Squamous cell cancer |
| Chronotype | rs1135946 | C | T | C | T | 0.017 | 0.061 | 0.232 | 0.246 | FALSE | 4 | 0.035 | 18313 | 0.069 | 4 | 0.002 | 413343 | 6.90E-13 | Squamous cell cancer |
| Chronotype | rs114870822 | A | G | A | G | -0.049 | 0.177 | 0.013 | 0.022 | FALSE | 2 | 0.108 | 18313 | 0.075 | 2 | 0.009 | 413343 | 4.60E-08 | Squamous cell cancer |
| Chronotype | rs11587758 | A | G | A | G | -0.019 | 0.024 | 0.396 | 0.396 | FALSE | 1 | 0.029 | 18313 | 0.400 | 1 | 0.002 | 413343 | 1.20E-19 | Squamous cell cancer |
| Chronotype | rs116131939 | T | C | T | C | -0.023 | -0.021 | 0.084 | 0.072 | FALSE | 2 | 0.053 | 18313 | 0.712 | 2 | 0.004 | 413343 | 8.00E-10 | Squamous cell cancer |
| Chronotype | rs11714441 | T | C | T | C | 0.012 | -0.004 | 0.401 | 0.405 | FALSE | 3 | 0.029 | 18313 | 0.897 | 3 | 0.002 | 413343 | 1.30E-08 | Squamous cell cancer |
| Chronotype | rs12117333 | A | G | A | G | 0.024 | -0.009 | 0.077 | 0.080 | FALSE | 1 | 0.049 | 18313 | 0.855 | 1 | 0.004 | 413343 | 2.40E-10 | Squamous cell cancer |
| Chronotype | rs12140153 | T | G | T | G | 0.027 | 0.073 | 0.094 | 0.082 | FALSE | 1 | 0.058 | 18313 | 0.205 | 1 | 0.004 | 413343 | 4.20E-14 | Squamous cell cancer |
| Chronotype | rs12249410 | T | G | T | G | 0.019 | -0.057 | 0.110 | 0.105 | FALSE | 10 | 0.042 | 18313 | 0.220 | 10 | 0.003 | 413343 | 1.00E-08 | Squamous cell cancer |
| Chronotype | rs12377175 | C | A | C | A | 0.016 | 0.035 | 0.229 | 0.229 | FALSE | 9 | 0.033 | 18313 | 0.291 | 9 | 0.002 | 413343 | 4.80E-11 | Squamous cell cancer |
| Chronotype | rs12432176 | A | C | A | C | -0.012 | 0.023 | 0.380 | 0.376 | FALSE | 14 | 0.030 | 18313 | 0.453 | 14 | 0.002 | 413343 | 9.70E-09 | Squamous cell cancer |
| Chronotype | rs12462111 | T | C | T | C | 0.013 | -0.049 | 0.465 | 0.429 | FALSE | 19 | 0.028 | 18313 | 0.105 | 19 | 0.002 | 413343 | 1.90E-10 | Squamous cell cancer |
| Chronotype | rs12525312 | C | T | C | T | 0.012 | -0.021 | 0.551 | 0.585 | FALSE | 6 | 0.028 | 18313 | 0.463 | 6 | 0.002 | 413343 | 1.90E-09 | Squamous cell cancer |
| Chronotype | rs12713014 | G | A | G | A | 0.026 | 0.058 | 0.059 | 0.074 | FALSE | 2 | 0.053 | 18313 | 0.276 | 2 | 0.004 | 413343 | 1.90E-09 | Squamous cell cancer |
| Chronotype | rs12811046 | G | A | G | A | 0.013 | -0.003 | 0.445 | 0.449 | FALSE | 12 | 0.027 | 18313 | 0.905 | 12 | 0.002 | 413343 | 2.40E-10 | Squamous cell cancer |
| Chronotype | rs12927162 | G | A | G | A | 0.021 | -0.001 | 0.277 | 0.287 | FALSE | 16 | 0.032 | 18313 | 0.972 | 16 | 0.002 | 413343 | 3.20E-20 | Squamous cell cancer |
| Chronotype | rs12965577 | G | A | G | A | 0.016 | 0.069 | 0.335 | 0.343 | FALSE | 18 | 0.030 | 18313 | 0.019 | 18 | 0.002 | 413343 | 2.00E-13 | Squamous cell cancer |
| Chronotype | rs12969848 | T | C | T | C | -0.016 | 0.023 | 0.529 | 0.535 | FALSE | 18 | 0.027 | 18313 | 0.426 | 18 | 0.002 | 413343 | 9.60E-16 | Squamous cell cancer |
| Chronotype | rs12971913 | A | G | A | G | 0.013 | -0.015 | 0.448 | 0.457 | FALSE | 19 | 0.027 | 18313 | 0.593 | 19 | 0.002 | 413343 | 6.60E-10 | Squamous cell cancer |
| Chronotype | rs13011556 | G | C | G | C | -0.017 | -0.017 | 0.239 | 0.242 | FALSE | 2 | 0.033 | 18313 | 0.622 | 2 | 0.002 | 413343 | 1.80E-12 | Squamous cell cancer |
| Chronotype | rs13059636 | G | A | G | A | -0.013 | 0.043 | 0.470 | 0.454 | FALSE | 3 | 0.029 | 18313 | 0.128 | 3 | 0.002 | 413343 | 6.00E-11 | Squamous cell cancer |
| Chronotype | rs13258797 | A | G | A | G | -0.016 | 0.031 | 0.169 | 0.193 | FALSE | 8 | 0.036 | 18313 | 0.389 | 8 | 0.003 | 413343 | 5.00E-09 | Squamous cell cancer |
| Chronotype | rs13316611 | T | G | T | G | -0.014 | 0.026 | 0.256 | 0.255 | FALSE | 3 | 0.032 | 18313 | 0.420 | 3 | 0.002 | 413343 | 6.80E-09 | Squamous cell cancer |
| Chronotype | rs138964083 | T | C | T | C | -0.026 | 0.050 | 0.059 | 0.055 | FALSE | 2 | 0.063 | 18313 | 0.432 | 2 | 0.004 | 413343 | 3.10E-09 | Squamous cell cancer |
| Chronotype | rs139911 | T | C | T | C | 0.018 | -0.007 | 0.576 | 0.566 | FALSE | 22 | 0.029 | 18313 | 0.803 | 22 | 0.002 | 413343 | 2.00E-17 | Squamous cell cancer |
| Chronotype | rs1421085 | C | T | C | T | -0.021 | 0.013 | 0.404 | 0.420 | FALSE | 16 | 0.028 | 18313 | 0.646 | 16 | 0.002 | 413343 | 8.90E-24 | Squamous cell cancer |
| Chronotype | rs1439319 | C | G | C | G | 0.012 | 0.009 | 0.645 | 0.666 | FALSE | 15 | 0.029 | 18313 | 0.760 | 15 | 0.002 | 413343 | 1.40E-08 | Squamous cell cancer |
| Chronotype | rs147762489 | T | C | T | C | 0.016 | -0.026 | 0.249 | 0.243 | FALSE | 5 | 0.031 | 18313 | 0.431 | 5 | 0.002 | 413343 | 5.20E-12 | Squamous cell cancer |
| Chronotype | rs17161045 | C | T | C | T | 0.015 | 0.024 | 0.370 | 0.363 | FALSE | 7 | 0.029 | 18313 | 0.406 | 7 | 0.002 | 413343 | 4.70E-13 | Squamous cell cancer |
| Chronotype | rs17448682 | T | C | T | C | -0.016 | 0.029 | 0.232 | 0.234 | FALSE | 1 | 0.034 | 18313 | 0.400 | 1 | 0.002 | 413343 | 7.90E-12 | Squamous cell cancer |
| Chronotype | rs17517 | A | G | A | G | 0.012 | 0.030 | 0.512 | 0.503 | FALSE | 13 | 0.027 | 18313 | 0.293 | 13 | 0.002 | 413343 | 1.30E-08 | Squamous cell cancer |
| Chronotype | rs17575798 | A | G | A | G | 0.016 | -0.010 | 0.193 | 0.192 | FALSE | 1 | 0.034 | 18313 | 0.785 | 1 | 0.003 | 413343 | 4.00E-10 | Squamous cell cancer |
| Chronotype | rs17604349 | A | G | A | G | 0.022 | 0.047 | 0.180 | 0.196 | FALSE | 16 | 0.036 | 18313 | 0.181 | 16 | 0.003 | 413343 | 1.40E-16 | Squamous cell cancer |
| Chronotype | rs17716502 | T | C | T | C | -0.019 | 0.073 | 0.204 | 0.185 | FALSE | 8 | 0.038 | 18313 | 0.049 | 8 | 0.003 | 413343 | 1.20E-13 | Squamous cell cancer |
| Chronotype | rs17786957 | C | G | C | G | 0.016 | -0.006 | 0.165 | 0.164 | FALSE | 3 | 0.037 | 18313 | 0.870 | 3 | 0.003 | 413343 | 2.90E-09 | Squamous cell cancer |
| Chronotype | rs1800828 | G | C | G | C | 0.013 | 0.055 | 0.253 | 0.239 | FALSE | 3 | 0.034 | 18313 | 0.095 | 3 | 0.002 | 413343 | 2.00E-08 | Squamous cell cancer |
| Chronotype | rs1874493 | G | A | G | A | 0.012 | -0.045 | 0.680 | 0.668 | FALSE | 19 | 0.030 | 18313 | 0.127 | 19 | 0.002 | 413343 | 1.80E-08 | Squamous cell cancer |
| Chronotype | rs1914772 | A | T | A | T | 0.021 | -0.076 | 0.895 | 0.898 | FALSE | 11 | 0.048 | 18313 | 0.105 | 11 | 0.003 | 413343 | 3.30E-10 | Squamous cell cancer |
| Chronotype | rs1927719 | A | T | A | T | -0.014 | -0.030 | 0.766 | 0.765 | FALSE | 13 | 0.033 | 18313 | 0.368 | 13 | 0.002 | 413343 | 6.70E-09 | Squamous cell cancer |
| Chronotype | rs197273 | G | A | G | A | 0.012 | 0.024 | 0.530 | 0.532 | FALSE | 2 | 0.027 | 18313 | 0.387 | 2 | 0.002 | 413343 | 3.10E-09 | Squamous cell cancer |
| Chronotype | rs1983891 | T | C | T | C | 0.013 | 0.017 | 0.276 | 0.281 | FALSE | 6 | 0.031 | 18313 | 0.589 | 6 | 0.002 | 413343 | 8.50E-09 | Squamous cell cancer |
| Chronotype | rs1996399 | A | G | A | G | -0.012 | -0.043 | 0.301 | 0.293 | FALSE | 7 | 0.029 | 18313 | 0.162 | 7 | 0.002 | 413343 | 4.30E-08 | Squamous cell cancer |
| Chronotype | rs202157 | T | C | T | C | 0.019 | -0.044 | 0.701 | 0.700 | FALSE | 7 | 0.031 | 18313 | 0.148 | 7 | 0.002 | 413343 | 1.70E-17 | Squamous cell cancer |
| Chronotype | rs2072727 | C | T | C | T | 0.012 | 0.010 | 0.564 | 0.571 | FALSE | 20 | 0.027 | 18313 | 0.729 | 20 | 0.002 | 413343 | 1.40E-08 | Squamous cell cancer |
| Chronotype | rs2077432 | T | C | T | C | -0.013 | 0.040 | 0.270 | 0.272 | FALSE | 11 | 0.032 | 18313 | 0.200 | 11 | 0.002 | 413343 | 5.70E-09 | Squamous cell cancer |
| Chronotype | rs2239626 | C | T | C | T | -0.014 | -0.012 | 0.306 | 0.297 | FALSE | 3 | 0.030 | 18313 | 0.700 | 3 | 0.002 | 413343 | 3.30E-10 | Squamous cell cancer |
| Chronotype | rs225298 | G | T | G | T | 0.015 | -0.043 | 0.831 | 0.818 | FALSE | 17 | 0.036 | 18313 | 0.237 | 17 | 0.003 | 413343 | 2.20E-08 | Squamous cell cancer |
| Chronotype | rs2291589 | G | T | G | T | 0.015 | 0.009 | 0.377 | 0.355 | FALSE | 9 | 0.029 | 18313 | 0.761 | 9 | 0.002 | 413343 | 4.50E-13 | Squamous cell cancer |
| Chronotype | rs2364972 | G | A | G | A | -0.013 | 0.002 | 0.463 | 0.443 | FALSE | 17 | 0.028 | 18313 | 0.954 | 17 | 0.002 | 413343 | 3.70E-10 | Squamous cell cancer |
| Chronotype | rs2518022 | C | T | C | T | 0.031 | 0.054 | 0.914 | 0.911 | FALSE | 17 | 0.044 | 18313 | 0.269 | 17 | 0.004 | 413343 | 2.60E-17 | Squamous cell cancer |
| Chronotype | rs2653349 | G | A | G | A | 0.028 | -0.049 | 0.787 | 0.799 | FALSE | 6 | 0.036 | 18313 | 0.160 | 6 | 0.002 | 413343 | 2.90E-30 | Squamous cell cancer |
| Chronotype | rs2701524 | C | T | C | T | 0.011 | 0.010 | 0.414 | 0.399 | FALSE | 15 | 0.028 | 18313 | 0.733 | 15 | 0.002 | 413343 | 3.20E-08 | Squamous cell cancer |
| Chronotype | rs2706762 | T | C | T | C | 0.018 | -0.021 | 0.150 | 0.146 | FALSE | 2 | 0.038 | 18313 | 0.607 | 2 | 0.003 | 413343 | 2.30E-10 | Squamous cell cancer |
| Chronotype | rs2712056 | T | C | T | C | -0.016 | 0.021 | 0.186 | 0.183 | FALSE | 2 | 0.035 | 18313 | 0.563 | 2 | 0.003 | 413343 | 1.80E-09 | Squamous cell cancer |
| Chronotype | rs2762088 | G | T | G | T | -0.014 | -0.016 | 0.761 | 0.771 | FALSE | 13 | 0.033 | 18313 | 0.619 | 13 | 0.002 | 413343 | 1.60E-09 | Squamous cell cancer |
| Chronotype | rs28380327 | T | A | T | A | 0.015 | -0.036 | 0.370 | 0.352 | FALSE | 2 | 0.028 | 18313 | 0.226 | 2 | 0.002 | 413343 | 2.50E-13 | Squamous cell cancer |
| Chronotype | rs2850298 | G | A | G | A | -0.019 | -0.001 | 0.699 | 0.669 | FALSE | 2 | 0.030 | 18313 | 0.975 | 2 | 0.002 | 413343 | 3.10E-17 | Squamous cell cancer |
| Chronotype | rs286808 | C | T | C | T | 0.012 | -0.014 | 0.525 | 0.548 | FALSE | 5 | 0.028 | 18313 | 0.622 | 5 | 0.002 | 413343 | 9.40E-09 | Squamous cell cancer |
| Chronotype | rs2881955 | T | C | T | C | -0.014 | -0.031 | 0.278 | 0.273 | FALSE | 6 | 0.030 | 18313 | 0.331 | 6 | 0.002 | 413343 | 2.00E-09 | Squamous cell cancer |
| Chronotype | rs2893787 | A | G | A | G | 0.015 | -0.034 | 0.744 | 0.737 | FALSE | 10 | 0.032 | 18313 | 0.294 | 10 | 0.002 | 413343 | 1.90E-10 | Squamous cell cancer |
| Chronotype | rs2971970 | G | T | G | T | 0.015 | 0.033 | 0.782 | 0.790 | FALSE | 7 | 0.032 | 18313 | 0.339 | 7 | 0.002 | 413343 | 8.00E-10 | Squamous cell cancer |
| Chronotype | rs3100052 | G | A | G | A | 0.012 | -0.032 | 0.613 | 0.598 | FALSE | 8 | 0.029 | 18313 | 0.273 | 8 | 0.002 | 413343 | 5.20E-09 | Squamous cell cancer |
| Chronotype | rs3168135 | A | G | A | G | 0.015 | 0.053 | 0.240 | 0.245 | FALSE | 11 | 0.033 | 18313 | 0.096 | 11 | 0.002 | 413343 | 3.20E-10 | Squamous cell cancer |
| Chronotype | rs34244172 | T | C | T | C | 0.012 | -0.020 | 0.292 | 0.288 | FALSE | 3 | 0.030 | 18313 | 0.527 | 3 | 0.002 | 413343 | 2.90E-08 | Squamous cell cancer |
| Chronotype | rs35101255 | G | A | G | A | 0.024 | -0.105 | 0.079 | 0.090 | FALSE | 6 | 0.043 | 18313 | 0.035 | 6 | 0.004 | 413343 | 8.50E-11 | Squamous cell cancer |
| Chronotype | rs35524253 | A | G | A | G | -0.012 | 0.038 | 0.356 | 0.356 | FALSE | 8 | 0.030 | 18313 | 0.196 | 8 | 0.002 | 413343 | 1.30E-08 | Squamous cell cancer |
| Chronotype | rs3729986 | T | C | T | C | -0.019 | 0.025 | 0.102 | 0.091 | FALSE | 11 | 0.051 | 18313 | 0.635 | 11 | 0.003 | 413343 | 1.70E-08 | Squamous cell cancer |
| Chronotype | rs3760185 | T | C | T | C | 0.019 | -0.026 | 0.248 | 0.238 | FALSE | 17 | 0.035 | 18313 | 0.486 | 17 | 0.002 | 413343 | 2.20E-15 | Squamous cell cancer |
| Chronotype | rs3808964 | T | G | T | G | -0.012 | 0.012 | 0.633 | 0.633 | FALSE | 10 | 0.028 | 18313 | 0.668 | 10 | 0.002 | 413343 | 1.00E-08 | Squamous cell cancer |
| Chronotype | rs4141920 | A | G | A | G | 0.012 | -0.014 | 0.455 | 0.462 | FALSE | 11 | 0.027 | 18313 | 0.626 | 11 | 0.002 | 413343 | 1.20E-08 | Squamous cell cancer |
| Chronotype | rs4237555 | T | C | T | C | -0.012 | -0.049 | 0.528 | 0.537 | FALSE | 11 | 0.030 | 18313 | 0.096 | 11 | 0.002 | 413343 | 9.40E-09 | Squamous cell cancer |
| Chronotype | rs4241964 | G | T | G | T | -0.015 | -0.017 | 0.476 | 0.475 | FALSE | 4 | 0.028 | 18313 | 0.559 | 4 | 0.002 | 413343 | 1.00E-13 | Squamous cell cancer |
| Chronotype | rs4321976 | C | T | C | T | 0.017 | -0.040 | 0.221 | 0.207 | FALSE | 8 | 0.032 | 18313 | 0.251 | 8 | 0.002 | 413343 | 2.30E-12 | Squamous cell cancer |
| Chronotype | rs4484214 | G | A | G | A | 0.014 | 0.026 | 0.315 | 0.324 | FALSE | 3 | 0.030 | 18313 | 0.395 | 3 | 0.002 | 413343 | 4.80E-10 | Squamous cell cancer |
| Chronotype | rs4518438 | C | T | C | T | -0.015 | -0.002 | 0.510 | 0.522 | FALSE | 5 | 0.027 | 18313 | 0.954 | 5 | 0.002 | 413343 | 6.30E-14 | Squamous cell cancer |
| Chronotype | rs4549082 | C | T | C | T | -0.015 | 0.023 | 0.484 | 0.494 | FALSE | 2 | 0.028 | 18313 | 0.409 | 2 | 0.002 | 413343 | 5.50E-14 | Squamous cell cancer |
| Chronotype | rs4595586 | T | A | T | A | 0.023 | 0.059 | 0.507 | 0.516 | FALSE | 12 | 0.026 | 18313 | 0.036 | 12 | 0.002 | 413343 | 6.40E-29 | Squamous cell cancer |
| Chronotype | rs4671379 | C | T | C | T | -0.012 | 0.040 | 0.586 | 0.588 | FALSE | 2 | 0.027 | 18313 | 0.165 | 2 | 0.002 | 413343 | 2.00E-09 | Squamous cell cancer |
| Chronotype | rs4784655 | C | G | C | G | 0.016 | 0.047 | 0.322 | 0.338 | FALSE | 16 | 0.031 | 18313 | 0.121 | 16 | 0.002 | 413343 | 7.40E-14 | Squamous cell cancer |
| Chronotype | rs4886947 | A | G | A | G | 0.012 | 0.034 | 0.660 | 0.653 | FALSE | 15 | 0.032 | 18313 | 0.322 | 15 | 0.002 | 413343 | 3.50E-08 | Squamous cell cancer |
| Chronotype | rs4936291 | G | A | G | A | -0.014 | -0.045 | 0.389 | 0.374 | FALSE | 11 | 0.029 | 18313 | 0.152 | 11 | 0.002 | 413343 | 2.10E-10 | Squamous cell cancer |
| Chronotype | rs4949980 | G | A | G | A | -0.023 | 0.061 | 0.067 | 0.075 | FALSE | 1 | 0.056 | 18313 | 0.273 | 1 | 0.004 | 413343 | 2.30E-08 | Squamous cell cancer |
| Chronotype | rs509476 | C | T | C | T | 0.088 | -0.132 | 0.970 | 0.975 | FALSE | 1 | 0.105 | 18313 | 0.193 | 1 | 0.006 | 413343 | 8.10E-49 | Squamous cell cancer |
| Chronotype | rs56076457 | T | C | T | C | -0.012 | 0.019 | 0.529 | 0.550 | FALSE | 18 | 0.027 | 18313 | 0.491 | 18 | 0.002 | 413343 | 8.90E-09 | Squamous cell cancer |
| Chronotype | rs56372114 | T | C | T | C | 0.012 | -0.034 | 0.384 | 0.395 | FALSE | 1 | 0.027 | 18313 | 0.237 | 1 | 0.002 | 413343 | 6.30E-09 | Squamous cell cancer |
| Chronotype | rs57435966 | T | C | T | C | 0.041 | -0.038 | 0.086 | 0.084 | FALSE | 2 | 0.046 | 18313 | 0.456 | 2 | 0.004 | 413343 | 8.50E-30 | Squamous cell cancer |
| Chronotype | rs57994353 | C | T | C | T | -0.013 | 0.036 | 0.299 | 0.295 | FALSE | 9 | 0.031 | 18313 | 0.248 | 9 | 0.002 | 413343 | 7.80E-09 | Squamous cell cancer |
| Chronotype | rs610590 | G | C | G | C | -0.015 | 0.032 | 0.212 | 0.204 | FALSE | 6 | 0.036 | 18313 | 0.362 | 6 | 0.002 | 413343 | 4.60E-09 | Squamous cell cancer |
| Chronotype | rs6131942 | G | A | G | A | -0.014 | 0.038 | 0.580 | 0.596 | FALSE | 20 | 0.027 | 18313 | 0.188 | 20 | 0.002 | 413343 | 4.50E-12 | Squamous cell cancer |
| Chronotype | rs61773390 | T | G | T | G | -0.026 | -0.006 | 0.195 | 0.189 | FALSE | 1 | 0.034 | 18313 | 0.865 | 1 | 0.003 | 413343 | 1.30E-23 | Squamous cell cancer |
| Chronotype | rs62082401 | G | C | G | C | -0.020 | -0.025 | 0.191 | 0.205 | FALSE | 18 | 0.033 | 18313 | 0.483 | 18 | 0.003 | 413343 | 9.60E-15 | Squamous cell cancer |
| Chronotype | rs62182115 | T | C | T | C | 0.013 | 0.016 | 0.342 | 0.336 | FALSE | 2 | 0.029 | 18313 | 0.601 | 2 | 0.002 | 413343 | 5.50E-10 | Squamous cell cancer |
| Chronotype | rs62553781 | T | C | T | C | 0.038 | 0.097 | 0.035 | 0.029 | FALSE | 9 | 0.087 | 18313 | 0.261 | 9 | 0.006 | 413343 | 1.10E-11 | Squamous cell cancer |
| Chronotype | rs6441169 | A | G | A | G | -0.016 | -0.033 | 0.856 | 0.869 | FALSE | 3 | 0.041 | 18313 | 0.427 | 3 | 0.003 | 413343 | 2.30E-08 | Squamous cell cancer |
| Chronotype | rs6442446 | G | A | G | A | 0.013 | -0.007 | 0.709 | 0.704 | FALSE | 3 | 0.030 | 18313 | 0.826 | 3 | 0.002 | 413343 | 7.20E-09 | Squamous cell cancer |
| Chronotype | rs6504758 | G | A | G | A | -0.012 | 0.001 | 0.536 | 0.565 | FALSE | 17 | 0.028 | 18313 | 0.984 | 17 | 0.002 | 413343 | 1.20E-09 | Squamous cell cancer |
| Chronotype | rs6601686 | T | A | T | A | 0.014 | 0.001 | 0.410 | 0.404 | FALSE | 8 | 0.028 | 18313 | 0.960 | 8 | 0.002 | 413343 | 4.60E-11 | Squamous cell cancer |
| Chronotype | rs6658041 | A | G | A | G | -0.012 | 0.004 | 0.599 | 0.611 | FALSE | 1 | 0.028 | 18313 | 0.900 | 1 | 0.002 | 413343 | 7.50E-09 | Squamous cell cancer |
| Chronotype | rs66710942 | T | C | T | C | -0.013 | 0.000 | 0.592 | 0.566 | FALSE | 3 | 0.029 | 18313 | 0.997 | 3 | 0.002 | 413343 | 1.60E-10 | Squamous cell cancer |
| Chronotype | rs6718119 | G | A | G | A | -0.012 | 0.007 | 0.377 | 0.372 | FALSE | 2 | 0.029 | 18313 | 0.823 | 2 | 0.002 | 413343 | 1.10E-08 | Squamous cell cancer |
| Chronotype | rs67988891 | G | C | G | C | -0.019 | 0.011 | 0.319 | 0.318 | FALSE | 5 | 0.029 | 18313 | 0.707 | 5 | 0.002 | 413343 | 1.10E-17 | Squamous cell cancer |
| Chronotype | rs6967481 | T | C | T | C | -0.016 | 0.035 | 0.497 | 0.496 | FALSE | 7 | 0.027 | 18313 | 0.219 | 7 | 0.002 | 413343 | 3.00E-14 | Squamous cell cancer |
| Chronotype | rs698015 | T | C | T | C | -0.013 | -0.014 | 0.647 | 0.637 | FALSE | 14 | 0.028 | 18313 | 0.619 | 14 | 0.002 | 413343 | 1.20E-09 | Squamous cell cancer |
| Chronotype | rs7148842 | T | C | T | C | 0.012 | 0.041 | 0.386 | 0.389 | FALSE | 14 | 0.031 | 18313 | 0.178 | 14 | 0.002 | 413343 | 7.40E-09 | Squamous cell cancer |
| Chronotype | rs72632979 | G | A | G | A | 0.017 | -0.018 | 0.172 | 0.157 | FALSE | 11 | 0.039 | 18313 | 0.652 | 11 | 0.003 | 413343 | 9.20E-10 | Squamous cell cancer |
| Chronotype | rs72720396 | G | A | G | A | -0.021 | -0.086 | 0.230 | 0.209 | FALSE | 1 | 0.032 | 18313 | 0.017 | 1 | 0.002 | 413343 | 1.30E-17 | Squamous cell cancer |
| Chronotype | rs7304278 | G | A | G | A | -0.015 | 0.028 | 0.724 | 0.697 | FALSE | 12 | 0.029 | 18313 | 0.358 | 12 | 0.002 | 413343 | 1.10E-10 | Squamous cell cancer |
| Chronotype | rs74097630 | T | G | T | G | 0.018 | 0.078 | 0.141 | 0.147 | FALSE | 12 | 0.041 | 18313 | 0.050 | 12 | 0.003 | 413343 | 4.80E-10 | Squamous cell cancer |
| Chronotype | rs74357745 | G | A | G | A | 0.020 | 0.046 | 0.121 | 0.125 | FALSE | 11 | 0.043 | 18313 | 0.291 | 11 | 0.003 | 413343 | 9.60E-11 | Squamous cell cancer |
| Chronotype | rs7547493 | G | A | G | A | -0.028 | 0.063 | 0.177 | 0.175 | FALSE | 1 | 0.038 | 18313 | 0.090 | 1 | 0.003 | 413343 | 3.70E-26 | Squamous cell cancer |
| Chronotype | rs76223855 | C | T | C | T | -0.080 | -0.198 | 0.011 | 0.012 | FALSE | 6 | 0.090 | 18313 | 0.110 | 6 | 0.010 | 413343 | 2.70E-16 | Squamous cell cancer |
| Chronotype | rs7626349 | C | T | C | T | -0.013 | 0.015 | 0.706 | 0.696 | FALSE | 3 | 0.029 | 18313 | 0.632 | 3 | 0.002 | 413343 | 1.90E-08 | Squamous cell cancer |
| Chronotype | rs7652260 | G | C | G | C | 0.016 | -0.015 | 0.162 | 0.159 | FALSE | 3 | 0.039 | 18313 | 0.712 | 3 | 0.003 | 413343 | 8.10E-09 | Squamous cell cancer |
| Chronotype | rs769066 | C | T | C | T | -0.017 | 0.016 | 0.184 | 0.189 | FALSE | 8 | 0.035 | 18313 | 0.646 | 8 | 0.003 | 413343 | 3.20E-10 | Squamous cell cancer |
| Chronotype | rs7783012 | A | G | A | G | -0.012 | 0.023 | 0.591 | 0.560 | FALSE | 7 | 0.027 | 18313 | 0.414 | 7 | 0.002 | 413343 | 5.20E-09 | Squamous cell cancer |
| Chronotype | rs78095690 | C | T | C | T | -0.011 | 0.028 | 0.437 | 0.444 | FALSE | 20 | 0.029 | 18313 | 0.342 | 20 | 0.002 | 413343 | 2.40E-08 | Squamous cell cancer |
| Chronotype | rs7959983 | C | T | C | T | -0.014 | 0.041 | 0.405 | 0.407 | FALSE | 12 | 0.029 | 18313 | 0.154 | 12 | 0.002 | 413343 | 9.00E-12 | Squamous cell cancer |
| Chronotype | rs80097534 | T | G | T | G | 0.022 | -0.051 | 0.098 | 0.116 | FALSE | 12 | 0.042 | 18313 | 0.273 | 12 | 0.003 | 413343 | 1.00E-10 | Squamous cell cancer |
| Chronotype | rs812925 | G | C | G | C | -0.015 | -0.008 | 0.352 | 0.366 | FALSE | 2 | 0.028 | 18313 | 0.789 | 2 | 0.002 | 413343 | 1.10E-12 | Squamous cell cancer |
| Chronotype | rs848552 | G | C | G | C | -0.012 | -0.009 | 0.528 | 0.541 | FALSE | 2 | 0.028 | 18313 | 0.744 | 2 | 0.002 | 413343 | 1.40E-09 | Squamous cell cancer |
| Chronotype | rs9291813 | C | T | C | T | -0.013 | 0.005 | 0.761 | 0.771 | FALSE | 5 | 0.032 | 18313 | 0.870 | 5 | 0.002 | 413343 | 1.90E-08 | Squamous cell cancer |
| Chronotype | rs9348050 | C | T | C | T | 0.012 | 0.019 | 0.511 | 0.522 | FALSE | 6 | 0.027 | 18313 | 0.492 | 6 | 0.002 | 413343 | 9.50E-10 | Squamous cell cancer |
| Chronotype | rs9395520 | T | C | T | C | -0.017 | -0.012 | 0.304 | 0.321 | FALSE | 6 | 0.029 | 18313 | 0.686 | 6 | 0.002 | 413343 | 4.10E-15 | Squamous cell cancer |
| Chronotype | rs9476310 | T | C | T | C | -0.013 | 0.004 | 0.511 | 0.514 | FALSE | 6 | 0.028 | 18313 | 0.894 | 6 | 0.002 | 413343 | 6.50E-10 | Squamous cell cancer |
| Chronotype | rs9573971 | G | A | G | A | 0.053 | 0.058 | 0.034 | 0.032 | FALSE | 13 | 0.077 | 18313 | 0.462 | 13 | 0.006 | 413343 | 9.70E-21 | Squamous cell cancer |
| Chronotype | rs9597250 | A | C | A | C | 0.016 | 0.048 | 0.189 | 0.191 | FALSE | 13 | 0.036 | 18313 | 0.179 | 13 | 0.003 | 413343 | 6.50E-10 | Squamous cell cancer |
| Chronotype | rs9795439 | G | A | G | A | 0.015 | 0.042 | 0.804 | 0.795 | FALSE | 11 | 0.035 | 18313 | 0.267 | 11 | 0.003 | 413343 | 7.90E-09 | Squamous cell cancer |
| Chronotype | rs9831488 | G | A | G | A | -0.014 | -0.017 | 0.353 | 0.343 | FALSE | 3 | 0.029 | 18313 | 0.574 | 3 | 0.002 | 413343 | 1.10E-10 | Squamous cell cancer |
| Chronotype | rs9932577 | A | C | A | C | 0.013 | -0.013 | 0.506 | 0.515 | FALSE | 16 | 0.029 | 18313 | 0.661 | 16 | 0.002 | 413343 | 7.40E-10 | Squamous cell cancer |
| Chronotype | rs9962650 | G | C | G | C | -0.014 | -0.018 | 0.423 | 0.412 | FALSE | 18 | 0.027 | 18313 | 0.532 | 18 | 0.002 | 413343 | 1.50E-11 | Squamous cell cancer |
| Chronotype | rs9964420 | A | C | A | C | 0.021 | 0.020 | 0.303 | 0.282 | FALSE | 18 | 0.031 | 18313 | 0.521 | 18 | 0.002 | 413343 | 4.40E-22 | Squamous cell cancer |
| Nap during day | rs1001817 | T | C | T | C | -0.008 | 0.020 | 0.494 | 0.509 | FALSE | 3 | 0.028 | 18313 | 0.472 | 3 | 0.001 | 462400 | 2.30E-10 | Squamous cell cancer |
| Nap during day | rs1011024 | G | A | G | A | -0.010 | -0.035 | 0.155 | 0.160 | FALSE | 7 | 0.035 | 18313 | 0.359 | 7 | 0.002 | 462400 | 1.70E-09 | Squamous cell cancer |
| Nap during day | rs10150432 | G | A | G | A | 0.010 | -0.017 | 0.185 | 0.182 | FALSE | 14 | 0.035 | 18313 | 0.653 | 14 | 0.002 | 462400 | 3.70E-11 | Squamous cell cancer |
| Nap during day | rs10757347 | G | A | G | A | 0.008 | -0.051 | 0.223 | 0.209 | FALSE | 9 | 0.032 | 18313 | 0.143 | 9 | 0.001 | 462400 | 4.10E-08 | Squamous cell cancer |
| Nap during day | rs10764260 | A | G | A | G | -0.007 | 0.017 | 0.363 | 0.369 | FALSE | 10 | 0.029 | 18313 | 0.551 | 10 | 0.001 | 462400 | 1.00E-08 | Squamous cell cancer |
| Nap during day | rs10835420 | A | T | A | T | -0.009 | 0.018 | 0.249 | 0.249 | FALSE | 11 | 0.033 | 18313 | 0.584 | 11 | 0.001 | 462400 | 1.30E-09 | Squamous cell cancer |
| Nap during day | rs10840017 | G | A | G | A | -0.009 | -0.007 | 0.233 | 0.233 | FALSE | 11 | 0.039 | 18313 | 0.874 | 11 | 0.001 | 462400 | 6.40E-09 | Squamous cell cancer |
| Nap during day | rs10868046 | A | G | A | G | 0.007 | -0.008 | 0.604 | 0.587 | FALSE | 9 | 0.029 | 18313 | 0.778 | 9 | 0.001 | 462400 | 7.90E-09 | Squamous cell cancer |
| Nap during day | rs10875622 | A | G | A | G | 0.010 | 0.031 | 0.576 | 0.575 | FALSE | 5 | 0.027 | 18313 | 0.279 | 5 | 0.001 | 462400 | 1.30E-16 | Squamous cell cancer |
| Nap during day | rs11071755 | A | G | A | G | -0.007 | 0.040 | 0.425 | 0.397 | FALSE | 15 | 0.030 | 18313 | 0.178 | 15 | 0.001 | 462400 | 1.90E-08 | Squamous cell cancer |
| Nap during day | rs11121194 | T | C | T | C | 0.007 | -0.013 | 0.634 | 0.612 | FALSE | 1 | 0.029 | 18313 | 0.647 | 1 | 0.001 | 462400 | 2.40E-08 | Squamous cell cancer |
| Nap during day | rs11125776 | G | T | G | T | -0.012 | 0.017 | 0.144 | 0.133 | FALSE | 2 | 0.040 | 18313 | 0.683 | 2 | 0.002 | 462400 | 5.60E-12 | Squamous cell cancer |
| Nap during day | rs11224896 | C | T | C | T | -0.011 | -0.056 | 0.110 | 0.100 | FALSE | 11 | 0.043 | 18313 | 0.237 | 11 | 0.002 | 462400 | 1.10E-08 | Squamous cell cancer |
| Nap during day | rs11252681 | A | G | A | G | 0.016 | -0.050 | 0.045 | 0.053 | FALSE | 10 | 0.063 | 18313 | 0.476 | 10 | 0.003 | 462400 | 4.10E-08 | Squamous cell cancer |
| Nap during day | rs11258652 | A | C | A | C | -0.010 | 0.026 | 0.236 | 0.236 | FALSE | 10 | 0.033 | 18313 | 0.423 | 10 | 0.001 | 462400 | 4.10E-12 | Squamous cell cancer |
| Nap during day | rs113886333 | T | C | T | C | 0.018 | -0.025 | 0.036 | 0.038 | FALSE | 4 | 0.069 | 18313 | 0.745 | 4 | 0.003 | 462400 | 3.00E-08 | Squamous cell cancer |
| Nap during day | rs11615756 | T | C | T | C | 0.018 | 0.046 | 0.404 | 0.395 | FALSE | 12 | 0.029 | 18313 | 0.109 | 12 | 0.001 | 462400 | 3.70E-48 | Squamous cell cancer |
| Nap during day | rs12042846 | C | T | C | T | 0.009 | 0.000 | 0.179 | 0.165 | FALSE | 1 | 0.039 | 18313 | 0.996 | 1 | 0.002 | 462400 | 1.80E-08 | Squamous cell cancer |
| Nap during day | rs12140153 | T | G | T | G | -0.024 | 0.073 | 0.094 | 0.082 | FALSE | 1 | 0.058 | 18313 | 0.205 | 1 | 0.002 | 462400 | 1.20E-29 | Squamous cell cancer |
| Nap during day | rs12346996 | C | T | C | T | -0.008 | -0.017 | 0.729 | 0.729 | FALSE | 9 | 0.032 | 18313 | 0.600 | 9 | 0.001 | 462400 | 1.50E-09 | Squamous cell cancer |
| Nap during day | rs12451365 | C | T | C | T | 0.011 | -0.047 | 0.204 | 0.208 | FALSE | 17 | 0.032 | 18313 | 0.177 | 17 | 0.002 | 462400 | 9.30E-13 | Squamous cell cancer |
| Nap during day | rs12615434 | T | C | T | C | 0.012 | 0.058 | 0.113 | 0.107 | FALSE | 2 | 0.046 | 18313 | 0.199 | 2 | 0.002 | 462400 | 1.80E-09 | Squamous cell cancer |
| Nap during day | rs12657723 | T | C | T | C | 0.008 | 0.046 | 0.321 | 0.320 | FALSE | 5 | 0.031 | 18313 | 0.126 | 5 | 0.001 | 462400 | 4.40E-10 | Squamous cell cancer |
| Nap during day | rs12992648 | G | A | G | A | -0.008 | 0.047 | 0.282 | 0.285 | FALSE | 2 | 0.033 | 18313 | 0.144 | 2 | 0.001 | 462400 | 2.10E-08 | Squamous cell cancer |
| Nap during day | rs13023587 | G | C | G | C | -0.008 | -0.035 | 0.508 | 0.497 | FALSE | 2 | 0.028 | 18313 | 0.216 | 2 | 0.001 | 462400 | 9.30E-11 | Squamous cell cancer |
| Nap during day | rs13033444 | G | A | G | A | 0.009 | 0.065 | 0.282 | 0.279 | FALSE | 2 | 0.033 | 18313 | 0.043 | 2 | 0.001 | 462400 | 4.60E-12 | Squamous cell cancer |
| Nap during day | rs13266972 | G | A | G | A | -0.007 | 0.016 | 0.698 | 0.694 | FALSE | 8 | 0.030 | 18313 | 0.612 | 8 | 0.001 | 462400 | 4.70E-08 | Squamous cell cancer |
| Nap during day | rs13284688 | C | T | C | T | 0.014 | 0.038 | 0.207 | 0.191 | FALSE | 9 | 0.036 | 18313 | 0.294 | 9 | 0.002 | 462400 | 1.40E-21 | Squamous cell cancer |
| Nap during day | rs1479116 | A | G | A | G | 0.008 | 0.019 | 0.351 | 0.349 | FALSE | 12 | 0.030 | 18313 | 0.524 | 12 | 0.001 | 462400 | 7.80E-10 | Squamous cell cancer |
| Nap during day | rs17158413 | A | G | A | G | 0.009 | -0.071 | 0.237 | 0.221 | FALSE | 15 | 0.031 | 18313 | 0.038 | 15 | 0.001 | 462400 | 3.00E-09 | Squamous cell cancer |
| Nap during day | rs17265513 | C | T | C | T | 0.009 | 0.041 | 0.198 | 0.198 | FALSE | 20 | 0.036 | 18313 | 0.240 | 20 | 0.002 | 462400 | 7.30E-09 | Squamous cell cancer |
| Nap during day | rs174541 | C | T | C | T | 0.010 | -0.065 | 0.359 | 0.363 | FALSE | 11 | 0.027 | 18313 | 0.029 | 11 | 0.001 | 462400 | 1.40E-15 | Squamous cell cancer |
| Nap during day | rs1856502 | A | T | A | T | 0.007 | -0.007 | 0.457 | 0.384 | FALSE | 6 | 0.030 | 18313 | 0.818 | 6 | 0.001 | 462400 | 7.20E-09 | Squamous cell cancer |
| Nap during day | rs1883048 | C | T | C | T | 0.008 | 0.032 | 0.525 | 0.541 | FALSE | 21 | 0.028 | 18313 | 0.292 | 21 | 0.001 | 462400 | 2.00E-10 | Squamous cell cancer |
| Nap during day | rs1931175 | G | C | G | C | 0.007 | 0.005 | 0.383 | 0.362 | FALSE | 1 | 0.028 | 18313 | 0.858 | 1 | 0.001 | 462400 | 3.20E-09 | Squamous cell cancer |
| Nap during day | rs2033103 | T | C | T | C | 0.008 | 0.060 | 0.450 | 0.455 | FALSE | 18 | 0.029 | 18313 | 0.032 | 18 | 0.001 | 462400 | 9.30E-10 | Squamous cell cancer |
| Nap during day | rs2099810 | G | A | G | A | -0.008 | -0.032 | 0.496 | 0.494 | FALSE | 5 | 0.029 | 18313 | 0.257 | 5 | 0.001 | 462400 | 1.90E-10 | Squamous cell cancer |
| Nap during day | rs2143792 | A | G | A | G | -0.007 | -0.027 | 0.431 | 0.428 | FALSE | 6 | 0.027 | 18313 | 0.349 | 6 | 0.001 | 462400 | 6.80E-09 | Squamous cell cancer |
| Nap during day | rs224111 | A | G | A | G | -0.008 | 0.017 | 0.389 | 0.406 | FALSE | 10 | 0.028 | 18313 | 0.548 | 10 | 0.001 | 462400 | 4.60E-10 | Squamous cell cancer |
| Nap during day | rs2284016 | C | T | C | T | 0.007 | -0.031 | 0.400 | 0.429 | FALSE | 22 | 0.027 | 18313 | 0.290 | 22 | 0.001 | 462400 | 1.40E-08 | Squamous cell cancer |
| Nap during day | rs2370926 | C | T | C | T | -0.008 | 0.012 | 0.367 | 0.352 | FALSE | 14 | 0.029 | 18313 | 0.683 | 14 | 0.001 | 462400 | 7.90E-11 | Squamous cell cancer |
| Nap during day | rs2390669 | C | A | C | A | -0.011 | 0.067 | 0.129 | 0.136 | FALSE | 2 | 0.043 | 18313 | 0.112 | 2 | 0.002 | 462400 | 8.40E-10 | Squamous cell cancer |
| Nap during day | rs2431108 | C | T | C | T | 0.013 | 0.018 | 0.328 | 0.330 | FALSE | 5 | 0.030 | 18313 | 0.560 | 5 | 0.001 | 462400 | 7.50E-22 | Squamous cell cancer |
| Nap during day | rs2653349 | G | A | G | A | -0.016 | -0.049 | 0.787 | 0.799 | FALSE | 6 | 0.036 | 18313 | 0.160 | 6 | 0.001 | 462400 | 1.00E-27 | Squamous cell cancer |
| Nap during day | rs2769916 | A | G | A | G | 0.009 | -0.044 | 0.689 | 0.691 | FALSE | 13 | 0.031 | 18313 | 0.154 | 13 | 0.001 | 462400 | 3.60E-11 | Squamous cell cancer |
| Nap during day | rs2786547 | T | C | T | C | -0.011 | 0.023 | 0.177 | 0.200 | FALSE | 1 | 0.036 | 18313 | 0.524 | 1 | 0.002 | 462400 | 6.80E-12 | Squamous cell cancer |
| Nap during day | rs285815 | A | T | A | T | -0.007 | 0.056 | 0.546 | 0.559 | FALSE | 8 | 0.026 | 18313 | 0.045 | 8 | 0.001 | 462400 | 2.00E-09 | Squamous cell cancer |
| Nap during day | rs34262487 | A | C | A | C | -0.015 | 0.011 | 0.073 | 0.082 | FALSE | 6 | 0.051 | 18313 | 0.829 | 6 | 0.002 | 462400 | 6.10E-10 | Squamous cell cancer |
| Nap during day | rs351776 | C | A | C | A | 0.008 | -0.023 | 0.548 | 0.550 | FALSE | 8 | 0.028 | 18313 | 0.407 | 8 | 0.001 | 462400 | 5.70E-10 | Squamous cell cancer |
| Nap during day | rs35851551 | G | A | G | A | -0.012 | 0.056 | 0.101 | 0.094 | FALSE | 7 | 0.051 | 18313 | 0.267 | 7 | 0.002 | 462400 | 5.50E-09 | Squamous cell cancer |
| Nap during day | rs3810484 | G | A | G | A | -0.007 | 0.009 | 0.443 | 0.435 | FALSE | 20 | 0.033 | 18313 | 0.800 | 20 | 0.001 | 462400 | 8.20E-09 | Squamous cell cancer |
| Nap during day | rs3935190 | A | G | A | G | 0.008 | -0.064 | 0.537 | 0.539 | FALSE | 17 | 0.030 | 18313 | 0.026 | 17 | 0.001 | 462400 | 2.10E-11 | Squamous cell cancer |
| Nap during day | rs40005 | A | G | A | G | 0.008 | -0.022 | 0.769 | 0.762 | FALSE | 3 | 0.033 | 18313 | 0.508 | 3 | 0.001 | 462400 | 2.80E-08 | Squamous cell cancer |
| Nap during day | rs4402351 | G | A | G | A | -0.012 | 0.056 | 0.148 | 0.171 | FALSE | 12 | 0.038 | 18313 | 0.130 | 12 | 0.002 | 462400 | 1.10E-11 | Squamous cell cancer |
| Nap during day | rs4587762 | A | G | A | G | -0.007 | -0.043 | 0.605 | 0.590 | FALSE | 11 | 0.029 | 18313 | 0.133 | 11 | 0.001 | 462400 | 3.30E-08 | Squamous cell cancer |
| Nap during day | rs467897 | A | G | A | G | -0.009 | -0.062 | 0.680 | 0.681 | FALSE | 5 | 0.032 | 18313 | 0.044 | 5 | 0.001 | 462400 | 1.50E-12 | Squamous cell cancer |
| Nap during day | rs4692709 | T | C | T | C | -0.007 | 0.002 | 0.547 | 0.557 | FALSE | 4 | 0.028 | 18313 | 0.947 | 4 | 0.001 | 462400 | 1.50E-08 | Squamous cell cancer |
| Nap during day | rs4856536 | A | G | A | G | -0.009 | -0.044 | 0.729 | 0.733 | FALSE | 3 | 0.032 | 18313 | 0.162 | 3 | 0.001 | 462400 | 4.80E-10 | Squamous cell cancer |
| Nap during day | rs60920123 | A | G | A | G | -0.007 | -0.014 | 0.433 | 0.417 | FALSE | 16 | 0.027 | 18313 | 0.619 | 16 | 0.001 | 462400 | 1.80E-09 | Squamous cell cancer |
| Nap during day | rs614987 | C | A | C | A | 0.011 | -0.006 | 0.614 | 0.615 | FALSE | 6 | 0.028 | 18313 | 0.844 | 6 | 0.001 | 462400 | 1.30E-17 | Squamous cell cancer |
| Nap during day | rs62425620 | T | C | T | C | 0.008 | -0.010 | 0.370 | 0.352 | FALSE | 6 | 0.030 | 18313 | 0.742 | 6 | 0.001 | 462400 | 1.20E-09 | Squamous cell cancer |
| Nap during day | rs62560863 | T | C | T | C | 0.011 | -0.060 | 0.101 | 0.109 | FALSE | 9 | 0.042 | 18313 | 0.198 | 9 | 0.002 | 462400 | 1.80E-08 | Squamous cell cancer |
| Nap during day | rs6452787 | G | A | G | A | -0.007 | -0.029 | 0.467 | 0.474 | FALSE | 5 | 0.026 | 18313 | 0.295 | 5 | 0.001 | 462400 | 3.50E-09 | Squamous cell cancer |
| Nap during day | rs6483215 | G | A | G | A | -0.008 | 0.029 | 0.764 | 0.770 | FALSE | 11 | 0.032 | 18313 | 0.384 | 11 | 0.001 | 462400 | 5.30E-09 | Squamous cell cancer |
| Nap during day | rs6919087 | G | T | G | T | -0.010 | -0.035 | 0.312 | 0.306 | FALSE | 6 | 0.029 | 18313 | 0.253 | 6 | 0.001 | 462400 | 4.30E-15 | Squamous cell cancer |
| Nap during day | rs6942927 | A | G | A | G | 0.015 | 0.008 | 0.123 | 0.211 | FALSE | 7 | 0.034 | 18313 | 0.812 | 7 | 0.002 | 462400 | 1.50E-11 | Squamous cell cancer |
| Nap during day | rs7038206 | G | A | G | A | 0.007 | -0.017 | 0.608 | 0.612 | FALSE | 9 | 0.028 | 18313 | 0.543 | 9 | 0.001 | 462400 | 4.00E-09 | Squamous cell cancer |
| Nap during day | rs7191614 | G | A | G | A | 0.008 | -0.021 | 0.291 | 0.260 | FALSE | 16 | 0.031 | 18313 | 0.505 | 16 | 0.001 | 462400 | 1.30E-08 | Squamous cell cancer |
| Nap during day | rs7198121 | C | T | C | T | -0.007 | 0.017 | 0.538 | 0.538 | FALSE | 16 | 0.027 | 18313 | 0.543 | 16 | 0.001 | 462400 | 3.00E-08 | Squamous cell cancer |
| Nap during day | rs72781017 | G | A | G | A | -0.008 | -0.009 | 0.403 | 0.437 | FALSE | 5 | 0.027 | 18313 | 0.758 | 5 | 0.001 | 462400 | 1.30E-09 | Squamous cell cancer |
| Nap during day | rs7422655 | T | C | T | C | -0.008 | -0.014 | 0.736 | 0.728 | FALSE | 2 | 0.031 | 18313 | 0.650 | 2 | 0.001 | 462400 | 7.10E-09 | Squamous cell cancer |
| Nap during day | rs75022160 | T | C | T | C | -0.010 | 0.004 | 0.137 | 0.123 | FALSE | 2 | 0.042 | 18313 | 0.925 | 2 | 0.002 | 462400 | 4.90E-08 | Squamous cell cancer |
| Nap during day | rs7555990 | T | C | T | C | -0.010 | -0.059 | 0.136 | 0.130 | FALSE | 1 | 0.038 | 18313 | 0.156 | 1 | 0.002 | 462400 | 6.80E-09 | Squamous cell cancer |
| Nap during day | rs7698842 | G | A | G | A | 0.008 | 0.025 | 0.742 | 0.722 | FALSE | 4 | 0.031 | 18313 | 0.443 | 4 | 0.001 | 462400 | 4.00E-08 | Squamous cell cancer |
| Nap during day | rs77154532 | G | A | G | A | -0.007 | -0.009 | 0.360 | 0.377 | FALSE | 3 | 0.028 | 18313 | 0.765 | 3 | 0.001 | 462400 | 1.60E-08 | Squamous cell cancer |
| Nap during day | rs7752899 | T | C | T | C | 0.008 | 0.024 | 0.443 | 0.441 | FALSE | 6 | 0.028 | 18313 | 0.391 | 6 | 0.001 | 462400 | 5.70E-12 | Squamous cell cancer |
| Nap during day | rs7814873 | T | C | T | C | -0.007 | -0.006 | 0.616 | 0.601 | FALSE | 8 | 0.030 | 18313 | 0.831 | 8 | 0.001 | 462400 | 3.30E-08 | Squamous cell cancer |
| Nap during day | rs785145 | G | T | G | T | 0.007 | 0.017 | 0.432 | 0.436 | FALSE | 6 | 0.028 | 18313 | 0.545 | 6 | 0.001 | 462400 | 1.10E-08 | Squamous cell cancer |
| Nap during day | rs8050478 | A | G | A | G | -0.008 | 0.006 | 0.501 | 0.486 | FALSE | 16 | 0.027 | 18313 | 0.836 | 16 | 0.001 | 462400 | 4.60E-10 | Squamous cell cancer |
| Nap during day | rs903678 | A | G | A | G | 0.014 | 0.008 | 0.337 | 0.324 | FALSE | 1 | 0.030 | 18313 | 0.791 | 1 | 0.001 | 462400 | 4.60E-26 | Squamous cell cancer |
| Nap during day | rs908442 | T | A | T | A | -0.010 | -0.008 | 0.408 | 0.399 | FALSE | 2 | 0.028 | 18313 | 0.785 | 2 | 0.001 | 462400 | 4.70E-15 | Squamous cell cancer |
| Nap during day | rs910187 | A | G | A | G | -0.007 | -0.011 | 0.373 | 0.374 | FALSE | 20 | 0.028 | 18313 | 0.713 | 20 | 0.001 | 462400 | 1.10E-08 | Squamous cell cancer |
| Nap during day | rs9467772 | T | A | T | A | -0.009 | 0.053 | 0.200 | 0.188 | FALSE | 6 | 0.037 | 18313 | 0.140 | 6 | 0.002 | 462400 | 2.40E-09 | Squamous cell cancer |
| Nap during day | rs962247 | A | G | A | G | -0.008 | -0.004 | 0.476 | 0.478 | FALSE | 18 | 0.028 | 18313 | 0.887 | 18 | 0.001 | 462400 | 3.60E-11 | Squamous cell cancer |
| Nap during day | rs971415 | G | A | G | A | -0.011 | 0.060 | 0.123 | 0.145 | FALSE | 9 | 0.040 | 18313 | 0.130 | 9 | 0.002 | 462400 | 6.30E-09 | Squamous cell cancer |
| Nap during day | rs9965170 | A | G | A | G | -0.014 | -0.007 | 0.424 | 0.428 | FALSE | 18 | 0.027 | 18313 | 0.795 | 18 | 0.001 | 462400 | 2.30E-30 | Squamous cell cancer |
| Nap during day | rs9998136 | G | C | G | C | 0.009 | -0.035 | 0.748 | 0.755 | FALSE | 4 | 0.033 | 18313 | 0.279 | 4 | 0.001 | 462400 | 2.20E-11 | Squamous cell cancer |
| Sleep duration | rs10510128 | A | G | A | G | 0.011 | -0.039 | 0.208 | 0.220 | FALSE | 10 | 0.032 | 18313 | 0.252 | 10 | 0.002 | 460099 | 7.70E-09 | Squamous cell cancer |
| Sleep duration | rs11039216 | T | C | T | C | -0.010 | -0.023 | 0.533 | 0.483 | FALSE | 11 | 0.029 | 18313 | 0.436 | 11 | 0.002 | 460099 | 1.50E-10 | Squamous cell cancer |
| Sleep duration | rs113021516 | C | G | C | G | 0.011 | -0.013 | 0.336 | 0.336 | FALSE | 3 | 0.029 | 18313 | 0.672 | 3 | 0.002 | 460099 | 1.30E-11 | Squamous cell cancer |
| Sleep duration | rs113113059 | C | T | C | T | -0.011 | 0.017 | 0.220 | 0.214 | FALSE | 6 | 0.034 | 18313 | 0.614 | 6 | 0.002 | 460099 | 8.60E-09 | Squamous cell cancer |
| Sleep duration | rs11621908 | T | C | T | C | -0.020 | -0.158 | 0.083 | 0.086 | FALSE | 14 | 0.043 | 18313 | 0.003 | 14 | 0.003 | 460099 | 1.10E-11 | Squamous cell cancer |
| Sleep duration | rs11643715 | G | C | G | C | 0.011 | -0.012 | 0.293 | 0.289 | FALSE | 16 | 0.030 | 18313 | 0.707 | 16 | 0.002 | 460099 | 5.00E-10 | Squamous cell cancer |
| Sleep duration | rs11650677 | A | G | A | G | 0.011 | -0.027 | 0.339 | 0.354 | FALSE | 17 | 0.028 | 18313 | 0.355 | 17 | 0.002 | 460099 | 3.80E-11 | Squamous cell cancer |
| Sleep duration | rs11982852 | T | C | T | C | -0.012 | -0.018 | 0.244 | 0.243 | FALSE | 7 | 0.031 | 18313 | 0.595 | 7 | 0.002 | 460099 | 3.10E-10 | Squamous cell cancer |
| Sleep duration | rs12518468 | C | T | C | T | -0.011 | 0.089 | 0.329 | 0.324 | FALSE | 5 | 0.032 | 18313 | 0.003 | 5 | 0.002 | 460099 | 4.10E-10 | Squamous cell cancer |
| Sleep duration | rs12567114 | A | G | A | G | 0.012 | -0.018 | 0.276 | 0.272 | FALSE | 1 | 0.030 | 18313 | 0.582 | 1 | 0.002 | 460099 | 6.10E-12 | Squamous cell cancer |
| Sleep duration | rs13107325 | T | C | T | C | -0.024 | 0.014 | 0.075 | 0.078 | FALSE | 4 | 0.053 | 18313 | 0.799 | 4 | 0.003 | 460099 | 1.40E-15 | Squamous cell cancer |
| Sleep duration | rs1348047 | T | G | T | G | -0.013 | 0.057 | 0.267 | 0.255 | FALSE | 18 | 0.033 | 18313 | 0.078 | 18 | 0.002 | 460099 | 3.80E-12 | Squamous cell cancer |
| Sleep duration | rs1463053 | A | G | A | G | 0.009 | -0.033 | 0.640 | 0.619 | FALSE | 1 | 0.030 | 18313 | 0.254 | 1 | 0.002 | 460099 | 2.40E-08 | Squamous cell cancer |
| Sleep duration | rs151014368 | A | G | A | G | 0.011 | -0.047 | 0.207 | 0.223 | FALSE | 5 | 0.032 | 18313 | 0.179 | 5 | 0.002 | 460099 | 1.00E-08 | Squamous cell cancer |
| Sleep duration | rs1517572 | C | A | C | A | 0.012 | -0.060 | 0.581 | 0.577 | FALSE | 11 | 0.030 | 18313 | 0.037 | 11 | 0.002 | 460099 | 6.50E-13 | Squamous cell cancer |
| Sleep duration | rs1553132 | G | A | G | A | 0.011 | -0.003 | 0.259 | 0.249 | FALSE | 11 | 0.031 | 18313 | 0.934 | 11 | 0.002 | 460099 | 8.10E-09 | Squamous cell cancer |
| Sleep duration | rs17391944 | G | T | G | T | 0.022 | 0.065 | 0.050 | 0.059 | FALSE | 9 | 0.061 | 18313 | 0.281 | 9 | 0.004 | 460099 | 4.40E-09 | Squamous cell cancer |
| Sleep duration | rs174564 | G | A | G | A | 0.010 | -0.065 | 0.349 | 0.332 | FALSE | 11 | 0.027 | 18313 | 0.031 | 11 | 0.002 | 460099 | 6.30E-09 | Squamous cell cancer |
| Sleep duration | rs1939455 | T | G | T | G | -0.016 | -0.042 | 0.120 | 0.111 | FALSE | 11 | 0.044 | 18313 | 0.385 | 11 | 0.003 | 460099 | 3.50E-10 | Squamous cell cancer |
| Sleep duration | rs1972712 | C | T | C | T | 0.012 | 0.005 | 0.249 | 0.255 | FALSE | 2 | 0.032 | 18313 | 0.876 | 2 | 0.002 | 460099 | 1.70E-10 | Squamous cell cancer |
| Sleep duration | rs2072727 | C | T | C | T | -0.009 | 0.010 | 0.565 | 0.571 | FALSE | 20 | 0.027 | 18313 | 0.729 | 20 | 0.002 | 460099 | 9.30E-09 | Squamous cell cancer |
| Sleep duration | rs2079070 | G | C | G | C | -0.013 | 0.036 | 0.735 | 0.726 | FALSE | 7 | 0.030 | 18313 | 0.267 | 7 | 0.002 | 460099 | 1.20E-13 | Squamous cell cancer |
| Sleep duration | rs2186122 | T | A | T | A | -0.011 | 0.057 | 0.560 | 0.527 | FALSE | 1 | 0.026 | 18313 | 0.044 | 1 | 0.002 | 460099 | 2.30E-11 | Squamous cell cancer |
| Sleep duration | rs2192528 | G | A | G | A | -0.010 | -0.017 | 0.522 | 0.520 | FALSE | 4 | 0.028 | 18313 | 0.561 | 4 | 0.002 | 460099 | 9.10E-10 | Squamous cell cancer |
| Sleep duration | rs2236295 | T | G | T | G | -0.009 | -0.006 | 0.403 | 0.385 | FALSE | 10 | 0.029 | 18313 | 0.846 | 10 | 0.002 | 460099 | 2.90E-08 | Squamous cell cancer |
| Sleep duration | rs2279681 | G | C | G | C | 0.009 | -0.025 | 0.342 | 0.352 | FALSE | 1 | 0.028 | 18313 | 0.402 | 1 | 0.002 | 460099 | 3.40E-08 | Squamous cell cancer |
| Sleep duration | rs2683630 | G | C | G | C | 0.015 | -0.009 | 0.629 | 0.594 | FALSE | 2 | 0.028 | 18313 | 0.758 | 2 | 0.002 | 460099 | 1.70E-19 | Squamous cell cancer |
| Sleep duration | rs2734831 | G | T | G | T | -0.010 | -0.047 | 0.607 | 0.590 | FALSE | 11 | 0.029 | 18313 | 0.101 | 11 | 0.002 | 460099 | 2.20E-09 | Squamous cell cancer |
| Sleep duration | rs2748809 | C | T | C | T | -0.009 | 0.008 | 0.429 | 0.414 | FALSE | 14 | 0.030 | 18313 | 0.805 | 14 | 0.002 | 460099 | 1.90E-08 | Squamous cell cancer |
| Sleep duration | rs2839753 | C | T | C | T | -0.011 | -0.011 | 0.265 | 0.264 | FALSE | 4 | 0.031 | 18313 | 0.738 | 4 | 0.002 | 460099 | 4.40E-09 | Squamous cell cancer |
| Sleep duration | rs2863957 | A | C | A | C | 0.029 | 0.018 | 0.221 | 0.226 | FALSE | 2 | 0.033 | 18313 | 0.595 | 2 | 0.002 | 460099 | 9.60E-51 | Squamous cell cancer |
| Sleep duration | rs34354917 | A | C | A | C | -0.010 | -0.011 | 0.289 | 0.285 | FALSE | 12 | 0.035 | 18313 | 0.759 | 12 | 0.002 | 460099 | 1.40E-08 | Squamous cell cancer |
| Sleep duration | rs34786000 | T | G | T | G | 0.011 | 0.004 | 0.553 | 0.531 | FALSE | 19 | 0.029 | 18313 | 0.889 | 19 | 0.002 | 460099 | 1.70E-11 | Squamous cell cancer |
| Sleep duration | rs35126035 | C | A | C | A | -0.009 | 0.033 | 0.558 | 0.529 | FALSE | 19 | 0.028 | 18313 | 0.270 | 19 | 0.002 | 460099 | 2.20E-08 | Squamous cell cancer |
| Sleep duration | rs35662245 | A | T | A | T | 0.010 | -0.014 | 0.339 | 0.346 | FALSE | 2 | 0.028 | 18313 | 0.644 | 2 | 0.002 | 460099 | 1.90E-09 | Squamous cell cancer |
| Sleep duration | rs365663 | G | A | G | A | -0.009 | 0.014 | 0.455 | 0.479 | FALSE | 5 | 0.029 | 18313 | 0.630 | 5 | 0.002 | 460099 | 8.10E-09 | Squamous cell cancer |
| Sleep duration | rs374153 | T | C | T | C | -0.013 | 0.110 | 0.843 | 0.845 | FALSE | 2 | 0.033 | 18313 | 0.004 | 2 | 0.002 | 460099 | 2.50E-09 | Squamous cell cancer |
| Sleep duration | rs4767550 | G | A | G | A | 0.011 | 0.041 | 0.413 | 0.405 | FALSE | 12 | 0.029 | 18313 | 0.158 | 12 | 0.002 | 460099 | 2.70E-11 | Squamous cell cancer |
| Sleep duration | rs55658675 | T | C | T | C | -0.010 | -0.040 | 0.353 | 0.339 | FALSE | 14 | 0.028 | 18313 | 0.180 | 14 | 0.002 | 460099 | 7.10E-09 | Squamous cell cancer |
| Sleep duration | rs56367859 | G | A | G | A | 0.012 | -0.011 | 0.398 | 0.413 | FALSE | 16 | 0.027 | 18313 | 0.695 | 16 | 0.002 | 460099 | 1.20E-12 | Squamous cell cancer |
| Sleep duration | rs62444917 | C | A | C | A | 0.013 | -0.033 | 0.222 | 0.245 | FALSE | 7 | 0.031 | 18313 | 0.330 | 7 | 0.002 | 460099 | 1.70E-11 | Squamous cell cancer |
| Sleep duration | rs6561715 | A | T | A | T | 0.010 | -0.006 | 0.631 | 0.634 | FALSE | 13 | 0.028 | 18313 | 0.840 | 13 | 0.002 | 460099 | 3.90E-09 | Squamous cell cancer |
| Sleep duration | rs6681755 | A | G | A | G | 0.012 | -0.072 | 0.200 | 0.193 | FALSE | 1 | 0.032 | 18313 | 0.044 | 1 | 0.002 | 460099 | 9.00E-09 | Squamous cell cancer |
| Sleep duration | rs6783516 | T | G | T | G | -0.010 | -0.041 | 0.584 | 0.589 | FALSE | 3 | 0.029 | 18313 | 0.155 | 3 | 0.002 | 460099 | 1.60E-09 | Squamous cell cancer |
| Sleep duration | rs6889592 | A | G | A | G | 0.012 | 0.059 | 0.333 | 0.332 | FALSE | 5 | 0.031 | 18313 | 0.046 | 5 | 0.002 | 460099 | 4.10E-12 | Squamous cell cancer |
| Sleep duration | rs7016314 | C | T | C | T | 0.010 | 0.003 | 0.656 | 0.656 | FALSE | 8 | 0.030 | 18313 | 0.925 | 8 | 0.002 | 460099 | 3.10E-09 | Squamous cell cancer |
| Sleep duration | rs7115856 | C | A | C | A | 0.011 | -0.045 | 0.461 | 0.460 | FALSE | 11 | 0.026 | 18313 | 0.108 | 11 | 0.002 | 460099 | 1.50E-11 | Squamous cell cancer |
| Sleep duration | rs72771082 | G | A | G | A | 0.011 | 0.009 | 0.218 | 0.230 | FALSE | 16 | 0.033 | 18313 | 0.780 | 16 | 0.002 | 460099 | 1.40E-08 | Squamous cell cancer |
| Sleep duration | rs72831782 | A | T | A | T | -0.010 | 0.011 | 0.269 | 0.254 | FALSE | 2 | 0.036 | 18313 | 0.765 | 2 | 0.002 | 460099 | 3.40E-08 | Squamous cell cancer |
| Sleep duration | rs7517981 | C | T | C | T | -0.010 | 0.068 | 0.601 | 0.583 | FALSE | 1 | 0.027 | 18313 | 0.021 | 1 | 0.002 | 460099 | 1.10E-09 | Squamous cell cancer |
| Sleep duration | rs75539574 | C | A | C | A | 0.024 | -0.026 | 0.086 | 0.087 | FALSE | 2 | 0.049 | 18313 | 0.632 | 2 | 0.003 | 460099 | 1.80E-16 | Squamous cell cancer |
| Sleep duration | rs76258078 | G | A | G | A | -0.022 | 0.162 | 0.050 | 0.039 | FALSE | 3 | 0.089 | 18313 | 0.047 | 3 | 0.004 | 460099 | 3.80E-09 | Squamous cell cancer |
| Sleep duration | rs7644809 | C | T | C | T | -0.010 | 0.037 | 0.576 | 0.577 | FALSE | 3 | 0.027 | 18313 | 0.191 | 3 | 0.002 | 460099 | 4.10E-10 | Squamous cell cancer |
| Sleep duration | rs7711696 | T | G | T | G | -0.010 | 0.028 | 0.305 | 0.291 | FALSE | 5 | 0.031 | 18313 | 0.358 | 5 | 0.002 | 460099 | 1.30E-08 | Squamous cell cancer |
| Sleep duration | rs7740402 | G | T | G | T | -0.010 | -0.011 | 0.306 | 0.320 | FALSE | 6 | 0.029 | 18313 | 0.725 | 6 | 0.002 | 460099 | 4.10E-08 | Squamous cell cancer |
| Sleep duration | rs7831557 | A | G | A | G | -0.011 | -0.035 | 0.517 | 0.522 | FALSE | 8 | 0.028 | 18313 | 0.218 | 8 | 0.002 | 460099 | 4.20E-11 | Squamous cell cancer |
| Sleep duration | rs8038326 | G | A | G | A | -0.013 | -0.005 | 0.273 | 0.290 | FALSE | 15 | 0.030 | 18313 | 0.871 | 15 | 0.002 | 460099 | 8.40E-14 | Squamous cell cancer |
| Sleep duration | rs8047587 | T | G | T | G | -0.011 | 0.034 | 0.440 | 0.447 | FALSE | 16 | 0.029 | 18313 | 0.239 | 16 | 0.002 | 460099 | 8.30E-12 | Squamous cell cancer |
| Sleep duration | rs915416 | G | C | G | C | -0.013 | -0.063 | 0.709 | 0.689 | FALSE | 1 | 0.032 | 18313 | 0.039 | 1 | 0.002 | 460099 | 4.80E-13 | Squamous cell cancer |
| Sleep duration | rs9302680 | A | G | A | G | 0.012 | 0.007 | 0.439 | 0.465 | FALSE | 16 | 0.027 | 18313 | 0.816 | 16 | 0.002 | 460099 | 7.60E-14 | Squamous cell cancer |
| Sleep duration | rs9345234 | C | A | C | A | 0.009 | -0.021 | 0.578 | 0.555 | FALSE | 6 | 0.028 | 18313 | 0.448 | 6 | 0.002 | 460099 | 1.50E-08 | Squamous cell cancer |
| Sleep duration | rs9382445 | C | T | C | T | -0.009 | -0.023 | 0.375 | 0.357 | FALSE | 6 | 0.028 | 18313 | 0.436 | 6 | 0.002 | 460099 | 8.90E-09 | Squamous cell cancer |
| Sleep duration | rs9611007 | T | C | T | C | -0.014 | 0.060 | 0.142 | 0.154 | FALSE | 22 | 0.041 | 18313 | 0.141 | 22 | 0.002 | 460099 | 3.30E-09 | Squamous cell cancer |
| Sleep duration | rs9810474 | T | C | T | C | -0.011 | 0.032 | 0.232 | 0.226 | FALSE | 3 | 0.034 | 18313 | 0.338 | 3 | 0.002 | 460099 | 3.90E-09 | Squamous cell cancer |
| Sleep duration | rs9903898 | T | C | T | C | -0.009 | 0.047 | 0.489 | 0.488 | FALSE | 17 | 0.032 | 18313 | 0.143 | 17 | 0.002 | 460099 | 3.60E-09 | Squamous cell cancer |
| Sleeplessness | rs10838708 | A | G | A | G | -0.009 | 0.000 | 0.459 | 0.479 | FALSE | 11 | 0.028 | 18313 | 0.996 | 11 | 0.002 | 462341 | 2.90E-10 | Squamous cell cancer |
| Sleeplessness | rs11097861 | G | A | G | A | 0.010 | -0.036 | 0.716 | 0.702 | FALSE | 4 | 0.031 | 18313 | 0.253 | 4 | 0.002 | 462341 | 1.10E-09 | Squamous cell cancer |
| Sleeplessness | rs11152363 | A | G | A | G | 0.016 | 0.039 | 0.186 | 0.188 | FALSE | 18 | 0.036 | 18313 | 0.267 | 18 | 0.002 | 462341 | 4.50E-16 | Squamous cell cancer |
| Sleeplessness | rs113851554 | T | G | T | G | 0.047 | -0.018 | 0.057 | 0.064 | FALSE | 2 | 0.058 | 18313 | 0.779 | 2 | 0.003 | 462341 | 2.90E-45 | Squamous cell cancer |
| Sleeplessness | rs11635495 | C | T | C | T | 0.009 | 0.064 | 0.512 | 0.506 | FALSE | 15 | 0.026 | 18313 | 0.023 | 15 | 0.001 | 462341 | 2.80E-10 | Squamous cell cancer |
| Sleeplessness | rs11790060 | C | T | C | T | -0.010 | 0.039 | 0.331 | 0.345 | FALSE | 9 | 0.030 | 18313 | 0.188 | 9 | 0.002 | 462341 | 5.80E-11 | Squamous cell cancer |
| Sleeplessness | rs12049261 | C | G | C | G | 0.011 | -0.025 | 0.293 | 0.287 | FALSE | 1 | 0.030 | 18313 | 0.433 | 1 | 0.002 | 462341 | 6.80E-12 | Squamous cell cancer |
| Sleeplessness | rs12470989 | G | A | G | A | -0.010 | -0.022 | 0.204 | 0.198 | FALSE | 2 | 0.033 | 18313 | 0.539 | 2 | 0.002 | 462341 | 2.80E-08 | Squamous cell cancer |
| Sleeplessness | rs1430205 | T | C | T | C | 0.009 | -0.022 | 0.462 | 0.475 | FALSE | 5 | 0.027 | 18313 | 0.436 | 5 | 0.001 | 462341 | 2.10E-10 | Squamous cell cancer |
| Sleeplessness | rs1547630 | A | G | A | G | 0.009 | -0.010 | 0.652 | 0.630 | FALSE | 13 | 0.031 | 18313 | 0.747 | 13 | 0.002 | 462341 | 5.80E-09 | Squamous cell cancer |
| Sleeplessness | rs1592757 | C | G | C | G | 0.010 | -0.001 | 0.356 | 0.362 | FALSE | 5 | 0.029 | 18313 | 0.968 | 5 | 0.002 | 462341 | 4.30E-11 | Squamous cell cancer |
| Sleeplessness | rs17151854 | T | G | T | G | 0.013 | 0.003 | 0.152 | 0.147 | FALSE | 8 | 0.039 | 18313 | 0.940 | 8 | 0.002 | 462341 | 3.80E-10 | Squamous cell cancer |
| Sleeplessness | rs17709610 | G | A | G | A | -0.010 | -0.009 | 0.298 | 0.270 | FALSE | 10 | 0.030 | 18313 | 0.783 | 10 | 0.002 | 462341 | 9.50E-10 | Squamous cell cancer |
| Sleeplessness | rs1988337 | G | A | G | A | 0.008 | -0.017 | 0.552 | 0.552 | FALSE | 4 | 0.029 | 18313 | 0.558 | 4 | 0.001 | 462341 | 2.10E-08 | Squamous cell cancer |
| Sleeplessness | rs2014830 | T | C | T | C | -0.012 | -0.001 | 0.304 | 0.326 | FALSE | 3 | 0.029 | 18313 | 0.972 | 3 | 0.002 | 462341 | 8.90E-13 | Squamous cell cancer |
| Sleeplessness | rs2062113 | C | T | C | T | -0.010 | 0.017 | 0.568 | 0.566 | FALSE | 16 | 0.028 | 18313 | 0.574 | 16 | 0.002 | 462341 | 1.60E-10 | Squamous cell cancer |
| Sleeplessness | rs224032 | A | G | A | G | 0.008 | 0.012 | 0.550 | 0.527 | FALSE | 10 | 0.027 | 18313 | 0.662 | 10 | 0.001 | 462341 | 1.80E-08 | Squamous cell cancer |
| Sleeplessness | rs2297787 | A | T | A | T | -0.018 | 0.052 | 0.080 | 0.094 | FALSE | 10 | 0.048 | 18313 | 0.276 | 10 | 0.003 | 462341 | 9.60E-11 | Squamous cell cancer |
| Sleeplessness | rs2604551 | G | T | G | T | -0.008 | 0.005 | 0.640 | 0.648 | FALSE | 4 | 0.029 | 18313 | 0.862 | 4 | 0.002 | 462341 | 4.70E-08 | Squamous cell cancer |
| Sleeplessness | rs2644128 | G | C | G | C | 0.011 | 0.015 | 0.548 | 0.528 | FALSE | 1 | 0.027 | 18313 | 0.582 | 1 | 0.001 | 462341 | 1.00E-12 | Squamous cell cancer |
| Sleeplessness | rs2803296 | C | G | C | G | -0.009 | 0.001 | 0.544 | 0.488 | FALSE | 1 | 0.030 | 18313 | 0.976 | 1 | 0.001 | 462341 | 7.30E-09 | Squamous cell cancer |
| Sleeplessness | rs314280 | G | A | G | A | 0.010 | 0.012 | 0.547 | 0.555 | FALSE | 6 | 0.027 | 18313 | 0.664 | 6 | 0.001 | 462341 | 7.30E-11 | Squamous cell cancer |
| Sleeplessness | rs324017 | C | A | C | A | -0.010 | -0.068 | 0.705 | 0.705 | FALSE | 12 | 0.032 | 18313 | 0.029 | 12 | 0.002 | 462341 | 1.40E-09 | Squamous cell cancer |
| Sleeplessness | rs4572538 | T | C | T | C | -0.010 | -0.027 | 0.364 | 0.382 | FALSE | 2 | 0.028 | 18313 | 0.377 | 2 | 0.002 | 462341 | 7.70E-10 | Squamous cell cancer |
| Sleeplessness | rs4577309 | G | A | G | A | -0.009 | -0.084 | 0.534 | 0.522 | FALSE | 2 | 0.030 | 18313 | 0.003 | 2 | 0.001 | 462341 | 1.00E-08 | Squamous cell cancer |
| Sleeplessness | rs4886860 | C | G | C | G | -0.012 | -0.029 | 0.767 | 0.767 | FALSE | 15 | 0.034 | 18313 | 0.392 | 15 | 0.002 | 462341 | 1.80E-11 | Squamous cell cancer |
| Sleeplessness | rs56093896 | A | C | A | C | -0.012 | 0.020 | 0.214 | 0.220 | FALSE | 2 | 0.034 | 18313 | 0.552 | 2 | 0.002 | 462341 | 7.70E-12 | Squamous cell cancer |
| Sleeplessness | rs56330606 | G | A | G | A | 0.009 | 0.000 | 0.379 | 0.349 | FALSE | 19 | 0.029 | 18313 | 0.994 | 19 | 0.002 | 462341 | 1.20E-09 | Squamous cell cancer |
| Sleeplessness | rs56365214 | A | C | A | C | -0.015 | -0.029 | 0.156 | 0.147 | FALSE | 2 | 0.038 | 18313 | 0.478 | 2 | 0.002 | 462341 | 5.60E-13 | Squamous cell cancer |
| Sleeplessness | rs6561715 | A | T | A | T | -0.012 | -0.006 | 0.631 | 0.634 | FALSE | 13 | 0.028 | 18313 | 0.840 | 13 | 0.002 | 462341 | 4.80E-14 | Squamous cell cancer |
| Sleeplessness | rs6690017 | G | T | G | T | -0.010 | -0.031 | 0.409 | 0.410 | FALSE | 1 | 0.027 | 18313 | 0.277 | 1 | 0.002 | 462341 | 1.10E-11 | Squamous cell cancer |
| Sleeplessness | rs68094047 | T | C | T | C | 0.010 | -0.012 | 0.251 | 0.245 | FALSE | 12 | 0.032 | 18313 | 0.728 | 12 | 0.002 | 462341 | 1.70E-09 | Squamous cell cancer |
| Sleeplessness | rs6975972 | G | A | G | A | -0.009 | -0.002 | 0.579 | 0.572 | FALSE | 7 | 0.028 | 18313 | 0.939 | 7 | 0.002 | 462341 | 2.00E-09 | Squamous cell cancer |
| Sleeplessness | rs705219 | A | T | A | T | 0.013 | 0.040 | 0.887 | 0.891 | FALSE | 3 | 0.041 | 18313 | 0.364 | 3 | 0.002 | 462341 | 1.20E-08 | Squamous cell cancer |
| Sleeplessness | rs72924721 | T | C | T | C | 0.016 | -0.067 | 0.073 | 0.071 | FALSE | 11 | 0.051 | 18313 | 0.246 | 11 | 0.003 | 462341 | 1.10E-08 | Squamous cell cancer |
| Sleeplessness | rs7711696 | T | G | T | G | 0.011 | 0.028 | 0.305 | 0.291 | FALSE | 5 | 0.031 | 18313 | 0.358 | 5 | 0.002 | 462341 | 4.10E-12 | Squamous cell cancer |
| Sleeplessness | rs8180817 | C | G | C | G | -0.010 | -0.024 | 0.431 | 0.439 | FALSE | 7 | 0.027 | 18313 | 0.393 | 7 | 0.002 | 462341 | 2.70E-11 | Squamous cell cancer |
| Sleeplessness | rs931221 | A | T | A | T | 0.011 | -0.002 | 0.237 | 0.236 | FALSE | 12 | 0.032 | 18313 | 0.946 | 12 | 0.002 | 462341 | 1.30E-09 | Squamous cell cancer |
| Sleeplessness | rs9570080 | C | T | C | T | -0.011 | -0.097 | 0.344 | 0.344 | FALSE | 13 | 0.026 | 18313 | 0.001 | 13 | 0.002 | 462341 | 1.60E-11 | Squamous cell cancer |
| Sleeplessness | rs9845387 | A | C | A | C | -0.022 | -0.017 | 0.040 | 0.043 | FALSE | 3 | 0.064 | 18313 | 0.812 | 3 | 0.004 | 462341 | 7.10E-09 | Squamous cell cancer |
| Sleeplessness | rs9894577 | A | G | A | G | 0.013 | 0.012 | 0.318 | 0.333 | FALSE | 17 | 0.029 | 18313 | 0.701 | 17 | 0.002 | 462341 | 1.30E-16 | Squamous cell cancer |
| Sleeplessness | rs9906181 | G | A | G | A | -0.009 | 0.015 | 0.688 | 0.674 | FALSE | 17 | 0.037 | 18313 | 0.692 | 17 | 0.002 | 462341 | 2.40E-08 | Squamous cell cancer |
| Nap during day | rs1001817 | T | C | T | C | -0.008 | 0.017 | 0.494 | 0.508 | FALSE | 3 | 0.028 | 18336 | 0.538 | 3 | 0.001 | 462400 | 2.30E-10 | Adenocarcinoma |
| Nap during day | rs1011024 | G | A | G | A | -0.010 | -0.010 | 0.155 | 0.159 | FALSE | 7 | 0.037 | 18336 | 0.786 | 7 | 0.002 | 462400 | 1.70E-09 | Adenocarcinoma |
| Nap during day | rs10150432 | G | A | G | A | 0.010 | 0.046 | 0.185 | 0.186 | FALSE | 14 | 0.037 | 18336 | 0.207 | 14 | 0.002 | 462400 | 3.70E-11 | Adenocarcinoma |
| Nap during day | rs10757347 | G | A | G | A | 0.008 | -0.011 | 0.223 | 0.208 | FALSE | 9 | 0.033 | 18336 | 0.763 | 9 | 0.001 | 462400 | 4.10E-08 | Adenocarcinoma |
| Nap during day | rs10764260 | A | G | A | G | -0.007 | 0.025 | 0.363 | 0.370 | FALSE | 10 | 0.029 | 18336 | 0.380 | 10 | 0.001 | 462400 | 1.00E-08 | Adenocarcinoma |
| Nap during day | rs10835420 | A | T | A | T | -0.009 | 0.042 | 0.249 | 0.252 | FALSE | 11 | 0.033 | 18336 | 0.198 | 11 | 0.001 | 462400 | 1.30E-09 | Adenocarcinoma |
| Nap during day | rs10840017 | G | A | G | A | -0.009 | 0.078 | 0.233 | 0.238 | FALSE | 11 | 0.041 | 18336 | 0.048 | 11 | 0.001 | 462400 | 6.40E-09 | Adenocarcinoma |
| Nap during day | rs10868046 | A | G | A | G | 0.007 | 0.048 | 0.604 | 0.588 | FALSE | 9 | 0.027 | 18336 | 0.100 | 9 | 0.001 | 462400 | 7.90E-09 | Adenocarcinoma |
| Nap during day | rs10875622 | A | G | A | G | 0.010 | 0.011 | 0.576 | 0.573 | FALSE | 5 | 0.027 | 18336 | 0.710 | 5 | 0.001 | 462400 | 1.30E-16 | Adenocarcinoma |
| Nap during day | rs11071755 | A | G | A | G | -0.007 | 0.066 | 0.425 | 0.400 | FALSE | 15 | 0.030 | 18336 | 0.024 | 15 | 0.001 | 462400 | 1.90E-08 | Adenocarcinoma |
| Nap during day | rs11121194 | T | C | T | C | 0.007 | 0.008 | 0.634 | 0.609 | FALSE | 1 | 0.028 | 18336 | 0.770 | 1 | 0.001 | 462400 | 2.40E-08 | Adenocarcinoma |
| Nap during day | rs11125776 | G | T | G | T | -0.012 | -0.034 | 0.144 | 0.132 | FALSE | 2 | 0.039 | 18336 | 0.417 | 2 | 0.002 | 462400 | 5.60E-12 | Adenocarcinoma |
| Nap during day | rs11224896 | C | T | C | T | -0.011 | -0.028 | 0.110 | 0.101 | FALSE | 11 | 0.043 | 18336 | 0.553 | 11 | 0.002 | 462400 | 1.10E-08 | Adenocarcinoma |
| Nap during day | rs11252681 | A | G | A | G | 0.016 | -0.004 | 0.045 | 0.053 | FALSE | 10 | 0.068 | 18336 | 0.961 | 10 | 0.003 | 462400 | 4.10E-08 | Adenocarcinoma |
| Nap during day | rs11258652 | A | C | A | C | -0.010 | -0.081 | 0.236 | 0.232 | FALSE | 10 | 0.029 | 18336 | 0.014 | 10 | 0.001 | 462400 | 4.10E-12 | Adenocarcinoma |
| Nap during day | rs113886333 | T | C | T | C | 0.018 | 0.083 | 0.036 | 0.039 | FALSE | 4 | 0.074 | 18336 | 0.259 | 4 | 0.003 | 462400 | 3.00E-08 | Adenocarcinoma |
| Nap during day | rs11615756 | T | C | T | C | 0.018 | 0.036 | 0.404 | 0.393 | FALSE | 12 | 0.029 | 18336 | 0.214 | 12 | 0.001 | 462400 | 3.70E-48 | Adenocarcinoma |
| Nap during day | rs12042846 | C | T | C | T | 0.009 | -0.019 | 0.179 | 0.165 | FALSE | 1 | 0.038 | 18336 | 0.644 | 1 | 0.002 | 462400 | 1.80E-08 | Adenocarcinoma |
| Nap during day | rs12140153 | T | G | T | G | -0.024 | -0.007 | 0.094 | 0.082 | FALSE | 1 | 0.056 | 18336 | 0.905 | 1 | 0.002 | 462400 | 1.20E-29 | Adenocarcinoma |
| Nap during day | rs12346996 | C | T | C | T | -0.008 | 0.038 | 0.729 | 0.731 | FALSE | 9 | 0.030 | 18336 | 0.248 | 9 | 0.001 | 462400 | 1.50E-09 | Adenocarcinoma |
| Nap during day | rs12451365 | C | T | C | T | 0.011 | -0.011 | 0.204 | 0.208 | FALSE | 17 | 0.034 | 18336 | 0.760 | 17 | 0.002 | 462400 | 9.30E-13 | Adenocarcinoma |
| Nap during day | rs12615434 | T | C | T | C | 0.012 | -0.025 | 0.113 | 0.104 | FALSE | 2 | 0.043 | 18336 | 0.583 | 2 | 0.002 | 462400 | 1.80E-09 | Adenocarcinoma |
| Nap during day | rs12657723 | T | C | T | C | 0.008 | 0.044 | 0.321 | 0.318 | FALSE | 5 | 0.031 | 18336 | 0.150 | 5 | 0.001 | 462400 | 4.40E-10 | Adenocarcinoma |
| Nap during day | rs12992648 | G | A | G | A | -0.008 | -0.015 | 0.282 | 0.283 | FALSE | 2 | 0.031 | 18336 | 0.640 | 2 | 0.001 | 462400 | 2.10E-08 | Adenocarcinoma |
| Nap during day | rs13023587 | G | C | G | C | -0.008 | -0.004 | 0.508 | 0.499 | FALSE | 2 | 0.027 | 18336 | 0.898 | 2 | 0.001 | 462400 | 9.30E-11 | Adenocarcinoma |
| Nap during day | rs13033444 | G | A | G | A | 0.009 | 0.019 | 0.282 | 0.277 | FALSE | 2 | 0.032 | 18336 | 0.559 | 2 | 0.001 | 462400 | 4.60E-12 | Adenocarcinoma |
| Nap during day | rs13266972 | G | A | G | A | -0.007 | 0.070 | 0.698 | 0.696 | FALSE | 8 | 0.028 | 18336 | 0.024 | 8 | 0.001 | 462400 | 4.70E-08 | Adenocarcinoma |
| Nap during day | rs13284688 | C | T | C | T | 0.014 | 0.064 | 0.207 | 0.192 | FALSE | 9 | 0.037 | 18336 | 0.072 | 9 | 0.002 | 462400 | 1.40E-21 | Adenocarcinoma |
| Nap during day | rs1479116 | A | G | A | G | 0.008 | 0.008 | 0.351 | 0.351 | FALSE | 12 | 0.029 | 18336 | 0.791 | 12 | 0.001 | 462400 | 7.80E-10 | Adenocarcinoma |
| Nap during day | rs17158413 | A | G | A | G | 0.009 | 0.000 | 0.237 | 0.225 | FALSE | 15 | 0.033 | 18336 | 0.997 | 15 | 0.001 | 462400 | 3.00E-09 | Adenocarcinoma |
| Nap during day | rs17265513 | C | T | C | T | 0.009 | -0.078 | 0.198 | 0.195 | FALSE | 20 | 0.033 | 18336 | 0.032 | 20 | 0.002 | 462400 | 7.30E-09 | Adenocarcinoma |
| Nap during day | rs174541 | C | T | C | T | 0.010 | -0.087 | 0.359 | 0.359 | FALSE | 11 | 0.027 | 18336 | 0.004 | 11 | 0.001 | 462400 | 1.40E-15 | Adenocarcinoma |
| Nap during day | rs1856502 | A | T | A | T | 0.007 | -0.035 | 0.457 | 0.384 | FALSE | 6 | 0.029 | 18336 | 0.249 | 6 | 0.001 | 462400 | 7.20E-09 | Adenocarcinoma |
| Nap during day | rs1883048 | C | T | C | T | 0.008 | 0.035 | 0.525 | 0.540 | FALSE | 21 | 0.028 | 18336 | 0.239 | 21 | 0.001 | 462400 | 2.00E-10 | Adenocarcinoma |
| Nap during day | rs1931175 | G | C | G | C | 0.007 | 0.004 | 0.383 | 0.359 | FALSE | 1 | 0.028 | 18336 | 0.902 | 1 | 0.001 | 462400 | 3.20E-09 | Adenocarcinoma |
| Nap during day | rs2033103 | T | C | T | C | 0.008 | -0.009 | 0.450 | 0.452 | FALSE | 18 | 0.027 | 18336 | 0.745 | 18 | 0.001 | 462400 | 9.30E-10 | Adenocarcinoma |
| Nap during day | rs2099810 | G | A | G | A | -0.008 | 0.042 | 0.496 | 0.498 | FALSE | 5 | 0.027 | 18336 | 0.139 | 5 | 0.001 | 462400 | 1.90E-10 | Adenocarcinoma |
| Nap during day | rs2143792 | A | G | A | G | -0.007 | -0.029 | 0.431 | 0.425 | FALSE | 6 | 0.027 | 18336 | 0.316 | 6 | 0.001 | 462400 | 6.80E-09 | Adenocarcinoma |
| Nap during day | rs224111 | A | G | A | G | -0.008 | 0.005 | 0.389 | 0.408 | FALSE | 10 | 0.028 | 18336 | 0.866 | 10 | 0.001 | 462400 | 4.60E-10 | Adenocarcinoma |
| Nap during day | rs2284016 | C | T | C | T | 0.007 | -0.027 | 0.400 | 0.422 | FALSE | 22 | 0.027 | 18336 | 0.350 | 22 | 0.001 | 462400 | 1.40E-08 | Adenocarcinoma |
| Nap during day | rs2370926 | C | T | C | T | -0.008 | -0.006 | 0.367 | 0.359 | FALSE | 14 | 0.029 | 18336 | 0.846 | 14 | 0.001 | 462400 | 7.90E-11 | Adenocarcinoma |
| Nap during day | rs2390669 | C | A | C | A | -0.011 | -0.005 | 0.129 | 0.134 | FALSE | 2 | 0.040 | 18336 | 0.896 | 2 | 0.002 | 462400 | 8.40E-10 | Adenocarcinoma |
| Nap during day | rs2431108 | C | T | C | T | 0.013 | -0.015 | 0.328 | 0.331 | FALSE | 5 | 0.029 | 18336 | 0.623 | 5 | 0.001 | 462400 | 7.50E-22 | Adenocarcinoma |
| Nap during day | rs2653349 | G | A | G | A | -0.016 | -0.045 | 0.787 | 0.800 | FALSE | 6 | 0.036 | 18336 | 0.206 | 6 | 0.001 | 462400 | 1.00E-27 | Adenocarcinoma |
| Nap during day | rs2769916 | A | G | A | G | 0.009 | 0.016 | 0.689 | 0.695 | FALSE | 13 | 0.029 | 18336 | 0.597 | 13 | 0.001 | 462400 | 3.60E-11 | Adenocarcinoma |
| Nap during day | rs2786547 | T | C | T | C | -0.011 | -0.008 | 0.177 | 0.198 | FALSE | 1 | 0.035 | 18336 | 0.828 | 1 | 0.002 | 462400 | 6.80E-12 | Adenocarcinoma |
| Nap during day | rs285815 | A | T | A | T | -0.007 | 0.018 | 0.546 | 0.555 | FALSE | 8 | 0.027 | 18336 | 0.533 | 8 | 0.001 | 462400 | 2.00E-09 | Adenocarcinoma |
| Nap during day | rs34262487 | A | C | A | C | -0.015 | 0.029 | 0.073 | 0.084 | FALSE | 6 | 0.049 | 18336 | 0.564 | 6 | 0.002 | 462400 | 6.10E-10 | Adenocarcinoma |
| Nap during day | rs351776 | C | A | C | A | 0.008 | 0.003 | 0.548 | 0.552 | FALSE | 8 | 0.027 | 18336 | 0.909 | 8 | 0.001 | 462400 | 5.70E-10 | Adenocarcinoma |
| Nap during day | rs35851551 | G | A | G | A | -0.012 | 0.026 | 0.101 | 0.095 | FALSE | 7 | 0.050 | 18336 | 0.616 | 7 | 0.002 | 462400 | 5.50E-09 | Adenocarcinoma |
| Nap during day | rs3810484 | G | A | G | A | -0.007 | -0.067 | 0.443 | 0.434 | FALSE | 20 | 0.030 | 18336 | 0.042 | 20 | 0.001 | 462400 | 8.20E-09 | Adenocarcinoma |
| Nap during day | rs3935190 | A | G | A | G | 0.008 | 0.028 | 0.537 | 0.543 | FALSE | 17 | 0.027 | 18336 | 0.337 | 17 | 0.001 | 462400 | 2.10E-11 | Adenocarcinoma |
| Nap during day | rs40005 | A | G | A | G | 0.008 | 0.052 | 0.769 | 0.765 | FALSE | 3 | 0.030 | 18336 | 0.115 | 3 | 0.001 | 462400 | 2.80E-08 | Adenocarcinoma |
| Nap during day | rs4402351 | G | A | G | A | -0.012 | 0.088 | 0.148 | 0.170 | FALSE | 12 | 0.039 | 18336 | 0.018 | 12 | 0.002 | 462400 | 1.10E-11 | Adenocarcinoma |
| Nap during day | rs4587762 | A | G | A | G | -0.007 | -0.053 | 0.605 | 0.595 | FALSE | 11 | 0.030 | 18336 | 0.067 | 11 | 0.001 | 462400 | 3.30E-08 | Adenocarcinoma |
| Nap during day | rs467897 | A | G | A | G | -0.009 | 0.035 | 0.680 | 0.686 | FALSE | 5 | 0.029 | 18336 | 0.261 | 5 | 0.001 | 462400 | 1.50E-12 | Adenocarcinoma |
| Nap during day | rs4692709 | T | C | T | C | -0.007 | 0.026 | 0.547 | 0.556 | FALSE | 4 | 0.027 | 18336 | 0.364 | 4 | 0.001 | 462400 | 1.50E-08 | Adenocarcinoma |
| Nap during day | rs4856536 | A | G | A | G | -0.009 | -0.047 | 0.729 | 0.733 | FALSE | 3 | 0.032 | 18336 | 0.138 | 3 | 0.001 | 462400 | 4.80E-10 | Adenocarcinoma |
| Nap during day | rs60920123 | A | G | A | G | -0.007 | -0.024 | 0.433 | 0.420 | FALSE | 16 | 0.027 | 18336 | 0.405 | 16 | 0.001 | 462400 | 1.80E-09 | Adenocarcinoma |
| Nap during day | rs614987 | C | A | C | A | 0.011 | 0.007 | 0.614 | 0.614 | FALSE | 6 | 0.028 | 18336 | 0.809 | 6 | 0.001 | 462400 | 1.30E-17 | Adenocarcinoma |
| Nap during day | rs62425620 | T | C | T | C | 0.008 | 0.076 | 0.370 | 0.357 | FALSE | 6 | 0.033 | 18336 | 0.015 | 6 | 0.001 | 462400 | 1.20E-09 | Adenocarcinoma |
| Nap during day | rs62560863 | T | C | T | C | 0.011 | -0.053 | 0.101 | 0.109 | FALSE | 9 | 0.042 | 18336 | 0.248 | 9 | 0.002 | 462400 | 1.80E-08 | Adenocarcinoma |
| Nap during day | rs6452787 | G | A | G | A | -0.007 | -0.005 | 0.467 | 0.478 | FALSE | 5 | 0.027 | 18336 | 0.853 | 5 | 0.001 | 462400 | 3.50E-09 | Adenocarcinoma |
| Nap during day | rs6483215 | G | A | G | A | -0.008 | -0.044 | 0.764 | 0.769 | FALSE | 11 | 0.034 | 18336 | 0.189 | 11 | 0.001 | 462400 | 5.30E-09 | Adenocarcinoma |
| Nap during day | rs6919087 | G | T | G | T | -0.010 | 0.017 | 0.312 | 0.309 | FALSE | 6 | 0.030 | 18336 | 0.573 | 6 | 0.001 | 462400 | 4.30E-15 | Adenocarcinoma |
| Nap during day | rs6942927 | A | G | A | G | 0.015 | 0.012 | 0.123 | 0.212 | FALSE | 7 | 0.033 | 18336 | 0.734 | 7 | 0.002 | 462400 | 1.50E-11 | Adenocarcinoma |
| Nap during day | rs7038206 | G | A | G | A | 0.007 | 0.000 | 0.608 | 0.612 | FALSE | 9 | 0.028 | 18336 | 0.999 | 9 | 0.001 | 462400 | 4.00E-09 | Adenocarcinoma |
| Nap during day | rs7191614 | G | A | G | A | 0.008 | -0.018 | 0.291 | 0.263 | FALSE | 16 | 0.030 | 18336 | 0.582 | 16 | 0.001 | 462400 | 1.30E-08 | Adenocarcinoma |
| Nap during day | rs7198121 | C | T | C | T | -0.007 | -0.032 | 0.538 | 0.532 | FALSE | 16 | 0.029 | 18336 | 0.266 | 16 | 0.001 | 462400 | 3.00E-08 | Adenocarcinoma |
| Nap during day | rs72781017 | G | A | G | A | -0.008 | 0.037 | 0.403 | 0.437 | FALSE | 5 | 0.028 | 18336 | 0.190 | 5 | 0.001 | 462400 | 1.30E-09 | Adenocarcinoma |
| Nap during day | rs7422655 | T | C | T | C | -0.008 | -0.019 | 0.736 | 0.727 | FALSE | 2 | 0.031 | 18336 | 0.558 | 2 | 0.001 | 462400 | 7.10E-09 | Adenocarcinoma |
| Nap during day | rs75022160 | T | C | T | C | -0.010 | -0.002 | 0.137 | 0.124 | FALSE | 2 | 0.042 | 18336 | 0.960 | 2 | 0.002 | 462400 | 4.90E-08 | Adenocarcinoma |
| Nap during day | rs7555990 | T | C | T | C | -0.010 | -0.020 | 0.136 | 0.129 | FALSE | 1 | 0.039 | 18336 | 0.622 | 1 | 0.002 | 462400 | 6.80E-09 | Adenocarcinoma |
| Nap during day | rs7698842 | G | A | G | A | 0.008 | 0.018 | 0.742 | 0.720 | FALSE | 4 | 0.031 | 18336 | 0.581 | 4 | 0.001 | 462400 | 4.00E-08 | Adenocarcinoma |
| Nap during day | rs77154532 | G | A | G | A | -0.007 | 0.010 | 0.360 | 0.379 | FALSE | 3 | 0.029 | 18336 | 0.734 | 3 | 0.001 | 462400 | 1.60E-08 | Adenocarcinoma |
| Nap during day | rs7752899 | T | C | T | C | 0.008 | 0.004 | 0.443 | 0.438 | FALSE | 6 | 0.028 | 18336 | 0.885 | 6 | 0.001 | 462400 | 5.70E-12 | Adenocarcinoma |
| Nap during day | rs7814873 | T | C | T | C | -0.007 | -0.003 | 0.616 | 0.600 | FALSE | 8 | 0.029 | 18336 | 0.923 | 8 | 0.001 | 462400 | 3.30E-08 | Adenocarcinoma |
| Nap during day | rs785145 | G | T | G | T | 0.007 | 0.026 | 0.432 | 0.439 | FALSE | 6 | 0.028 | 18336 | 0.348 | 6 | 0.001 | 462400 | 1.10E-08 | Adenocarcinoma |
| Nap during day | rs8050478 | A | G | A | G | -0.008 | -0.007 | 0.501 | 0.487 | FALSE | 16 | 0.027 | 18336 | 0.808 | 16 | 0.001 | 462400 | 4.60E-10 | Adenocarcinoma |
| Nap during day | rs903678 | A | G | A | G | 0.014 | -0.001 | 0.337 | 0.324 | FALSE | 1 | 0.029 | 18336 | 0.964 | 1 | 0.001 | 462400 | 4.60E-26 | Adenocarcinoma |
| Nap during day | rs908442 | T | A | T | A | -0.010 | -0.005 | 0.408 | 0.402 | FALSE | 2 | 0.028 | 18336 | 0.875 | 2 | 0.001 | 462400 | 4.70E-15 | Adenocarcinoma |
| Nap during day | rs910187 | A | G | A | G | -0.007 | -0.003 | 0.373 | 0.374 | FALSE | 20 | 0.029 | 18336 | 0.921 | 20 | 0.001 | 462400 | 1.10E-08 | Adenocarcinoma |
| Nap during day | rs9467772 | T | A | T | A | -0.009 | -0.031 | 0.200 | 0.187 | FALSE | 6 | 0.034 | 18336 | 0.386 | 6 | 0.002 | 462400 | 2.40E-09 | Adenocarcinoma |
| Nap during day | rs962247 | A | G | A | G | -0.008 | 0.016 | 0.476 | 0.479 | FALSE | 18 | 0.028 | 18336 | 0.577 | 18 | 0.001 | 462400 | 3.60E-11 | Adenocarcinoma |
| Nap during day | rs971415 | G | A | G | A | -0.011 | -0.030 | 0.123 | 0.142 | FALSE | 9 | 0.038 | 18336 | 0.461 | 9 | 0.002 | 462400 | 6.30E-09 | Adenocarcinoma |
| Nap during day | rs9965170 | A | G | A | G | -0.014 | -0.044 | 0.424 | 0.426 | FALSE | 18 | 0.026 | 18336 | 0.118 | 18 | 0.001 | 462400 | 2.30E-30 | Adenocarcinoma |
| Nap during day | rs9998136 | G | C | G | C | 0.009 | 0.016 | 0.748 | 0.758 | FALSE | 4 | 0.031 | 18336 | 0.633 | 4 | 0.001 | 462400 | 2.20E-11 | Adenocarcinoma |
| Chronotype | rs10058356 | T | C | T | C | 0.013 | 0.034 | 0.698 | 0.693 | FALSE | 5 | 0.028 | 18336 | 0.262 | 5 | 0.002 | 413343 | 3.70E-09 | Adenocarcinoma |
| Chronotype | rs10118767 | T | C | T | C | 0.014 | 0.028 | 0.198 | 0.228 | FALSE | 9 | 0.033 | 18336 | 0.403 | 9 | 0.003 | 413343 | 4.50E-08 | Adenocarcinoma |
| Chronotype | rs10149448 | G | A | G | A | 0.012 | -0.045 | 0.396 | 0.407 | FALSE | 14 | 0.028 | 18336 | 0.131 | 14 | 0.002 | 413343 | 3.00E-08 | Adenocarcinoma |
| Chronotype | rs10175975 | T | C | T | C | -0.018 | 0.017 | 0.182 | 0.191 | FALSE | 2 | 0.035 | 18336 | 0.628 | 2 | 0.003 | 413343 | 4.40E-12 | Adenocarcinoma |
| Chronotype | rs10280205 | C | T | C | T | 0.013 | 0.067 | 0.309 | 0.322 | FALSE | 7 | 0.031 | 18336 | 0.025 | 7 | 0.002 | 413343 | 1.50E-09 | Adenocarcinoma |
| Chronotype | rs10402849 | T | C | T | C | -0.015 | -0.002 | 0.202 | 0.200 | FALSE | 19 | 0.034 | 18336 | 0.964 | 19 | 0.003 | 413343 | 2.30E-09 | Adenocarcinoma |
| Chronotype | rs10461917 | C | T | C | T | 0.012 | -0.001 | 0.690 | 0.704 | FALSE | 5 | 0.030 | 18336 | 0.974 | 5 | 0.002 | 413343 | 2.50E-08 | Adenocarcinoma |
| Chronotype | rs1056322 | G | C | G | C | 0.013 | 0.041 | 0.321 | 0.327 | FALSE | 22 | 0.031 | 18336 | 0.180 | 22 | 0.002 | 413343 | 6.60E-09 | Adenocarcinoma |
| Chronotype | rs10737452 | T | C | T | C | 0.014 | -0.033 | 0.624 | 0.598 | FALSE | 1 | 0.029 | 18336 | 0.251 | 1 | 0.002 | 413343 | 7.90E-12 | Adenocarcinoma |
| Chronotype | rs10742179 | G | A | G | A | 0.013 | 0.008 | 0.739 | 0.726 | FALSE | 11 | 0.030 | 18336 | 0.807 | 11 | 0.002 | 413343 | 2.60E-08 | Adenocarcinoma |
| Chronotype | rs10954933 | G | A | G | A | -0.015 | -0.014 | 0.428 | 0.423 | FALSE | 8 | 0.027 | 18336 | 0.630 | 8 | 0.002 | 413343 | 6.00E-14 | Adenocarcinoma |
| Chronotype | rs10988239 | T | C | T | C | 0.013 | 0.008 | 0.512 | 0.498 | FALSE | 9 | 0.030 | 18336 | 0.794 | 9 | 0.002 | 413343 | 4.70E-10 | Adenocarcinoma |
| Chronotype | rs11032362 | A | G | A | G | -0.026 | 0.044 | 0.091 | 0.096 | FALSE | 11 | 0.047 | 18336 | 0.344 | 11 | 0.004 | 413343 | 1.20E-13 | Adenocarcinoma |
| Chronotype | rs111761918 | A | G | A | G | 0.022 | -0.056 | 0.068 | 0.074 | FALSE | 1 | 0.050 | 18336 | 0.312 | 1 | 0.004 | 413343 | 4.80E-08 | Adenocarcinoma |
| Chronotype | rs11183201 | C | T | C | T | -0.013 | -0.023 | 0.508 | 0.510 | FALSE | 12 | 0.029 | 18336 | 0.420 | 12 | 0.002 | 413343 | 2.40E-10 | Adenocarcinoma |
| Chronotype | rs112555644 | T | C | T | C | -0.029 | 0.019 | 0.068 | 0.071 | FALSE | 3 | 0.067 | 18336 | 0.785 | 3 | 0.004 | 413343 | 1.30E-11 | Adenocarcinoma |
| Chronotype | rs113171806 | C | T | C | T | 0.018 | 0.028 | 0.114 | 0.097 | FALSE | 9 | 0.061 | 18336 | 0.656 | 9 | 0.003 | 413343 | 1.40E-08 | Adenocarcinoma |
| Chronotype | rs1135946 | C | T | C | T | 0.017 | -0.036 | 0.232 | 0.242 | FALSE | 4 | 0.032 | 18336 | 0.288 | 4 | 0.002 | 413343 | 6.90E-13 | Adenocarcinoma |
| Chronotype | rs114870822 | A | G | A | G | -0.049 | -0.062 | 0.013 | 0.021 | FALSE | 2 | 0.088 | 18336 | 0.553 | 2 | 0.009 | 413343 | 4.60E-08 | Adenocarcinoma |
| Chronotype | rs11587758 | A | G | A | G | -0.019 | 0.017 | 0.396 | 0.395 | FALSE | 1 | 0.029 | 18336 | 0.553 | 1 | 0.002 | 413343 | 1.20E-19 | Adenocarcinoma |
| Chronotype | rs116131939 | T | C | T | C | -0.023 | 0.087 | 0.084 | 0.075 | FALSE | 2 | 0.056 | 18336 | 0.107 | 2 | 0.004 | 413343 | 8.00E-10 | Adenocarcinoma |
| Chronotype | rs11714441 | T | C | T | C | 0.012 | 0.042 | 0.401 | 0.404 | FALSE | 3 | 0.030 | 18336 | 0.157 | 3 | 0.002 | 413343 | 1.30E-08 | Adenocarcinoma |
| Chronotype | rs12117333 | A | G | A | G | 0.024 | -0.044 | 0.077 | 0.078 | FALSE | 1 | 0.049 | 18336 | 0.414 | 1 | 0.004 | 413343 | 2.40E-10 | Adenocarcinoma |
| Chronotype | rs12140153 | T | G | T | G | 0.027 | -0.007 | 0.094 | 0.082 | FALSE | 1 | 0.056 | 18336 | 0.905 | 1 | 0.004 | 413343 | 4.20E-14 | Adenocarcinoma |
| Chronotype | rs12249410 | T | G | T | G | 0.019 | -0.090 | 0.110 | 0.103 | FALSE | 10 | 0.040 | 18336 | 0.052 | 10 | 0.003 | 413343 | 1.00E-08 | Adenocarcinoma |
| Chronotype | rs12377175 | C | A | C | A | 0.016 | 0.002 | 0.229 | 0.226 | FALSE | 9 | 0.032 | 18336 | 0.947 | 9 | 0.002 | 413343 | 4.80E-11 | Adenocarcinoma |
| Chronotype | rs12432176 | A | C | A | C | -0.012 | -0.001 | 0.380 | 0.372 | FALSE | 14 | 0.029 | 18336 | 0.971 | 14 | 0.002 | 413343 | 9.70E-09 | Adenocarcinoma |
| Chronotype | rs12462111 | T | C | T | C | 0.013 | 0.051 | 0.465 | 0.435 | FALSE | 19 | 0.031 | 18336 | 0.093 | 19 | 0.002 | 413343 | 1.90E-10 | Adenocarcinoma |
| Chronotype | rs12525312 | C | T | C | T | 0.012 | -0.017 | 0.551 | 0.582 | FALSE | 6 | 0.028 | 18336 | 0.546 | 6 | 0.002 | 413343 | 1.90E-09 | Adenocarcinoma |
| Chronotype | rs12713014 | G | A | G | A | 0.026 | 0.014 | 0.059 | 0.072 | FALSE | 2 | 0.053 | 18336 | 0.795 | 2 | 0.004 | 413343 | 1.90E-09 | Adenocarcinoma |
| Chronotype | rs12811046 | G | A | G | A | 0.013 | -0.011 | 0.445 | 0.445 | FALSE | 12 | 0.027 | 18336 | 0.689 | 12 | 0.002 | 413343 | 2.40E-10 | Adenocarcinoma |
| Chronotype | rs12927162 | G | A | G | A | 0.021 | -0.015 | 0.277 | 0.283 | FALSE | 16 | 0.032 | 18336 | 0.662 | 16 | 0.002 | 413343 | 3.20E-20 | Adenocarcinoma |
| Chronotype | rs12965577 | G | A | G | A | 0.016 | 0.000 | 0.335 | 0.338 | FALSE | 18 | 0.029 | 18336 | 0.993 | 18 | 0.002 | 413343 | 2.00E-13 | Adenocarcinoma |
| Chronotype | rs12969848 | T | C | T | C | -0.016 | 0.021 | 0.529 | 0.534 | FALSE | 18 | 0.027 | 18336 | 0.458 | 18 | 0.002 | 413343 | 9.60E-16 | Adenocarcinoma |
| Chronotype | rs12971913 | A | G | A | G | 0.013 | 0.007 | 0.448 | 0.453 | FALSE | 19 | 0.028 | 18336 | 0.802 | 19 | 0.002 | 413343 | 6.60E-10 | Adenocarcinoma |
| Chronotype | rs13011556 | G | C | G | C | -0.017 | -0.011 | 0.239 | 0.244 | FALSE | 2 | 0.034 | 18336 | 0.763 | 2 | 0.002 | 413343 | 1.80E-12 | Adenocarcinoma |
| Chronotype | rs13059636 | G | A | G | A | -0.013 | 0.018 | 0.470 | 0.451 | FALSE | 3 | 0.028 | 18336 | 0.535 | 3 | 0.002 | 413343 | 6.00E-11 | Adenocarcinoma |
| Chronotype | rs13258797 | A | G | A | G | -0.016 | -0.028 | 0.169 | 0.194 | FALSE | 8 | 0.034 | 18336 | 0.444 | 8 | 0.003 | 413343 | 5.00E-09 | Adenocarcinoma |
| Chronotype | rs13316611 | T | G | T | G | -0.014 | 0.034 | 0.256 | 0.255 | FALSE | 3 | 0.032 | 18336 | 0.287 | 3 | 0.002 | 413343 | 6.80E-09 | Adenocarcinoma |
| Chronotype | rs138964083 | T | C | T | C | -0.026 | 0.001 | 0.059 | 0.055 | FALSE | 2 | 0.059 | 18336 | 0.982 | 2 | 0.004 | 413343 | 3.10E-09 | Adenocarcinoma |
| Chronotype | rs139911 | T | C | T | C | 0.018 | 0.013 | 0.576 | 0.565 | FALSE | 22 | 0.028 | 18336 | 0.649 | 22 | 0.002 | 413343 | 2.00E-17 | Adenocarcinoma |
| Chronotype | rs1421085 | C | T | C | T | -0.021 | -0.015 | 0.404 | 0.417 | FALSE | 16 | 0.028 | 18336 | 0.597 | 16 | 0.002 | 413343 | 8.90E-24 | Adenocarcinoma |
| Chronotype | rs1439319 | C | G | C | G | 0.012 | 0.019 | 0.645 | 0.669 | FALSE | 15 | 0.029 | 18336 | 0.546 | 15 | 0.002 | 413343 | 1.40E-08 | Adenocarcinoma |
| Chronotype | rs147762489 | T | C | T | C | 0.016 | -0.029 | 0.249 | 0.245 | FALSE | 5 | 0.031 | 18336 | 0.379 | 5 | 0.002 | 413343 | 5.20E-12 | Adenocarcinoma |
| Chronotype | rs17161045 | C | T | C | T | 0.015 | 0.023 | 0.370 | 0.364 | FALSE | 7 | 0.029 | 18336 | 0.422 | 7 | 0.002 | 413343 | 4.70E-13 | Adenocarcinoma |
| Chronotype | rs17448682 | T | C | T | C | -0.016 | -0.008 | 0.232 | 0.234 | FALSE | 1 | 0.033 | 18336 | 0.822 | 1 | 0.002 | 413343 | 7.90E-12 | Adenocarcinoma |
| Chronotype | rs17517 | A | G | A | G | 0.012 | 0.022 | 0.512 | 0.506 | FALSE | 13 | 0.027 | 18336 | 0.426 | 13 | 0.002 | 413343 | 1.30E-08 | Adenocarcinoma |
| Chronotype | rs17575798 | A | G | A | G | 0.016 | -0.078 | 0.193 | 0.191 | FALSE | 1 | 0.032 | 18336 | 0.029 | 1 | 0.003 | 413343 | 4.00E-10 | Adenocarcinoma |
| Chronotype | rs17604349 | A | G | A | G | 0.022 | -0.013 | 0.180 | 0.193 | FALSE | 16 | 0.034 | 18336 | 0.709 | 16 | 0.003 | 413343 | 1.40E-16 | Adenocarcinoma |
| Chronotype | rs17716502 | T | C | T | C | -0.019 | 0.049 | 0.204 | 0.184 | FALSE | 8 | 0.037 | 18336 | 0.181 | 8 | 0.003 | 413343 | 1.20E-13 | Adenocarcinoma |
| Chronotype | rs17786957 | C | G | C | G | 0.016 | -0.068 | 0.165 | 0.165 | FALSE | 3 | 0.034 | 18336 | 0.073 | 3 | 0.003 | 413343 | 2.90E-09 | Adenocarcinoma |
| Chronotype | rs1800828 | G | C | G | C | 0.013 | -0.006 | 0.253 | 0.237 | FALSE | 3 | 0.032 | 18336 | 0.859 | 3 | 0.002 | 413343 | 2.00E-08 | Adenocarcinoma |
| Chronotype | rs1874493 | G | A | G | A | 0.012 | 0.014 | 0.680 | 0.671 | FALSE | 19 | 0.029 | 18336 | 0.630 | 19 | 0.002 | 413343 | 1.80E-08 | Adenocarcinoma |
| Chronotype | rs1914772 | A | T | A | T | 0.021 | 0.030 | 0.895 | 0.900 | FALSE | 11 | 0.043 | 18336 | 0.516 | 11 | 0.003 | 413343 | 3.30E-10 | Adenocarcinoma |
| Chronotype | rs1927719 | A | T | A | T | -0.014 | -0.059 | 0.766 | 0.767 | FALSE | 13 | 0.034 | 18336 | 0.075 | 13 | 0.002 | 413343 | 6.70E-09 | Adenocarcinoma |
| Chronotype | rs197273 | G | A | G | A | 0.012 | -0.004 | 0.530 | 0.535 | FALSE | 2 | 0.028 | 18336 | 0.893 | 2 | 0.002 | 413343 | 3.10E-09 | Adenocarcinoma |
| Chronotype | rs1983891 | T | C | T | C | 0.013 | 0.026 | 0.276 | 0.280 | FALSE | 6 | 0.031 | 18336 | 0.410 | 6 | 0.002 | 413343 | 8.50E-09 | Adenocarcinoma |
| Chronotype | rs1996399 | A | G | A | G | -0.012 | 0.019 | 0.301 | 0.297 | FALSE | 7 | 0.031 | 18336 | 0.529 | 7 | 0.002 | 413343 | 4.30E-08 | Adenocarcinoma |
| Chronotype | rs202157 | T | C | T | C | 0.019 | -0.045 | 0.701 | 0.696 | FALSE | 7 | 0.031 | 18336 | 0.137 | 7 | 0.002 | 413343 | 1.70E-17 | Adenocarcinoma |
| Chronotype | rs2072727 | C | T | C | T | 0.012 | -0.039 | 0.564 | 0.570 | FALSE | 20 | 0.029 | 18336 | 0.176 | 20 | 0.002 | 413343 | 1.40E-08 | Adenocarcinoma |
| Chronotype | rs2077432 | T | C | T | C | -0.013 | -0.041 | 0.270 | 0.272 | FALSE | 11 | 0.030 | 18336 | 0.197 | 11 | 0.002 | 413343 | 5.70E-09 | Adenocarcinoma |
| Chronotype | rs2239626 | C | T | C | T | -0.014 | 0.000 | 0.306 | 0.300 | FALSE | 3 | 0.030 | 18336 | 0.998 | 3 | 0.002 | 413343 | 3.30E-10 | Adenocarcinoma |
| Chronotype | rs225298 | G | T | G | T | 0.015 | -0.017 | 0.831 | 0.818 | FALSE | 17 | 0.035 | 18336 | 0.627 | 17 | 0.003 | 413343 | 2.20E-08 | Adenocarcinoma |
| Chronotype | rs2291589 | G | T | G | T | 0.015 | -0.016 | 0.377 | 0.358 | FALSE | 9 | 0.028 | 18336 | 0.582 | 9 | 0.002 | 413343 | 4.50E-13 | Adenocarcinoma |
| Chronotype | rs2364972 | G | A | G | A | -0.013 | -0.023 | 0.463 | 0.445 | FALSE | 17 | 0.027 | 18336 | 0.413 | 17 | 0.002 | 413343 | 3.70E-10 | Adenocarcinoma |
| Chronotype | rs2518022 | C | T | C | T | 0.031 | -0.017 | 0.914 | 0.908 | FALSE | 17 | 0.046 | 18336 | 0.722 | 17 | 0.004 | 413343 | 2.60E-17 | Adenocarcinoma |
| Chronotype | rs2653349 | G | A | G | A | 0.028 | -0.045 | 0.787 | 0.800 | FALSE | 6 | 0.036 | 18336 | 0.206 | 6 | 0.002 | 413343 | 2.90E-30 | Adenocarcinoma |
| Chronotype | rs2701524 | C | T | C | T | 0.011 | 0.027 | 0.414 | 0.403 | FALSE | 15 | 0.028 | 18336 | 0.338 | 15 | 0.002 | 413343 | 3.20E-08 | Adenocarcinoma |
| Chronotype | rs2706762 | T | C | T | C | 0.018 | 0.073 | 0.150 | 0.150 | FALSE | 2 | 0.041 | 18336 | 0.064 | 2 | 0.003 | 413343 | 2.30E-10 | Adenocarcinoma |
| Chronotype | rs2712056 | T | C | T | C | -0.016 | -0.008 | 0.186 | 0.186 | FALSE | 2 | 0.034 | 18336 | 0.813 | 2 | 0.003 | 413343 | 1.80E-09 | Adenocarcinoma |
| Chronotype | rs2762088 | G | T | G | T | -0.014 | -0.062 | 0.761 | 0.770 | FALSE | 13 | 0.034 | 18336 | 0.058 | 13 | 0.002 | 413343 | 1.60E-09 | Adenocarcinoma |
| Chronotype | rs28380327 | T | A | T | A | 0.015 | 0.050 | 0.370 | 0.356 | FALSE | 2 | 0.030 | 18336 | 0.087 | 2 | 0.002 | 413343 | 2.50E-13 | Adenocarcinoma |
| Chronotype | rs2850298 | G | A | G | A | -0.019 | -0.014 | 0.699 | 0.669 | FALSE | 2 | 0.031 | 18336 | 0.647 | 2 | 0.002 | 413343 | 3.10E-17 | Adenocarcinoma |
| Chronotype | rs286808 | C | T | C | T | 0.012 | -0.018 | 0.525 | 0.546 | FALSE | 5 | 0.028 | 18336 | 0.524 | 5 | 0.002 | 413343 | 9.40E-09 | Adenocarcinoma |
| Chronotype | rs2881955 | T | C | T | C | -0.014 | -0.017 | 0.278 | 0.274 | FALSE | 6 | 0.030 | 18336 | 0.587 | 6 | 0.002 | 413343 | 2.00E-09 | Adenocarcinoma |
| Chronotype | rs2893787 | A | G | A | G | 0.015 | 0.032 | 0.744 | 0.739 | FALSE | 10 | 0.030 | 18336 | 0.309 | 10 | 0.002 | 413343 | 1.90E-10 | Adenocarcinoma |
| Chronotype | rs2971970 | G | T | G | T | 0.015 | 0.002 | 0.782 | 0.786 | FALSE | 7 | 0.033 | 18336 | 0.957 | 7 | 0.002 | 413343 | 8.00E-10 | Adenocarcinoma |
| Chronotype | rs3100052 | G | A | G | A | 0.012 | -0.006 | 0.613 | 0.601 | FALSE | 8 | 0.028 | 18336 | 0.839 | 8 | 0.002 | 413343 | 5.20E-09 | Adenocarcinoma |
| Chronotype | rs3168135 | A | G | A | G | 0.015 | -0.021 | 0.240 | 0.241 | FALSE | 11 | 0.031 | 18336 | 0.510 | 11 | 0.002 | 413343 | 3.20E-10 | Adenocarcinoma |
| Chronotype | rs34244172 | T | C | T | C | 0.012 | 0.039 | 0.292 | 0.292 | FALSE | 3 | 0.032 | 18336 | 0.217 | 3 | 0.002 | 413343 | 2.90E-08 | Adenocarcinoma |
| Chronotype | rs35101255 | G | A | G | A | 0.024 | 0.012 | 0.079 | 0.089 | FALSE | 6 | 0.049 | 18336 | 0.813 | 6 | 0.004 | 413343 | 8.50E-11 | Adenocarcinoma |
| Chronotype | rs35524253 | A | G | A | G | -0.012 | -0.025 | 0.356 | 0.353 | FALSE | 8 | 0.028 | 18336 | 0.403 | 8 | 0.002 | 413343 | 1.30E-08 | Adenocarcinoma |
| Chronotype | rs3729986 | T | C | T | C | -0.019 | 0.011 | 0.102 | 0.092 | FALSE | 11 | 0.050 | 18336 | 0.836 | 11 | 0.003 | 413343 | 1.70E-08 | Adenocarcinoma |
| Chronotype | rs3760185 | T | C | T | C | 0.019 | -0.019 | 0.248 | 0.237 | FALSE | 17 | 0.035 | 18336 | 0.609 | 17 | 0.002 | 413343 | 2.20E-15 | Adenocarcinoma |
| Chronotype | rs3808964 | T | G | T | G | -0.012 | 0.053 | 0.633 | 0.636 | FALSE | 10 | 0.027 | 18336 | 0.066 | 10 | 0.002 | 413343 | 1.00E-08 | Adenocarcinoma |
| Chronotype | rs4141920 | A | G | A | G | 0.012 | 0.010 | 0.455 | 0.461 | FALSE | 11 | 0.028 | 18336 | 0.736 | 11 | 0.002 | 413343 | 1.20E-08 | Adenocarcinoma |
| Chronotype | rs4237555 | T | C | T | C | -0.012 | -0.021 | 0.528 | 0.535 | FALSE | 11 | 0.029 | 18336 | 0.476 | 11 | 0.002 | 413343 | 9.40E-09 | Adenocarcinoma |
| Chronotype | rs4241964 | G | T | G | T | -0.015 | -0.006 | 0.476 | 0.473 | FALSE | 4 | 0.028 | 18336 | 0.831 | 4 | 0.002 | 413343 | 1.00E-13 | Adenocarcinoma |
| Chronotype | rs4321976 | C | T | C | T | 0.017 | 0.018 | 0.221 | 0.211 | FALSE | 8 | 0.034 | 18336 | 0.605 | 8 | 0.002 | 413343 | 2.30E-12 | Adenocarcinoma |
| Chronotype | rs4484214 | G | A | G | A | 0.014 | 0.017 | 0.315 | 0.323 | FALSE | 3 | 0.030 | 18336 | 0.576 | 3 | 0.002 | 413343 | 4.80E-10 | Adenocarcinoma |
| Chronotype | rs4518438 | C | T | C | T | -0.015 | -0.009 | 0.510 | 0.521 | FALSE | 5 | 0.028 | 18336 | 0.750 | 5 | 0.002 | 413343 | 6.30E-14 | Adenocarcinoma |
| Chronotype | rs4549082 | C | T | C | T | -0.015 | -0.011 | 0.484 | 0.496 | FALSE | 2 | 0.027 | 18336 | 0.705 | 2 | 0.002 | 413343 | 5.50E-14 | Adenocarcinoma |
| Chronotype | rs4595586 | T | A | T | A | 0.023 | 0.020 | 0.507 | 0.514 | FALSE | 12 | 0.027 | 18336 | 0.486 | 12 | 0.002 | 413343 | 6.40E-29 | Adenocarcinoma |
| Chronotype | rs4671379 | C | T | C | T | -0.012 | 0.018 | 0.586 | 0.586 | FALSE | 2 | 0.027 | 18336 | 0.535 | 2 | 0.002 | 413343 | 2.00E-09 | Adenocarcinoma |
| Chronotype | rs4784655 | C | G | C | G | 0.016 | -0.007 | 0.322 | 0.332 | FALSE | 16 | 0.029 | 18336 | 0.823 | 16 | 0.002 | 413343 | 7.40E-14 | Adenocarcinoma |
| Chronotype | rs4886947 | A | G | A | G | 0.012 | 0.019 | 0.660 | 0.652 | FALSE | 15 | 0.032 | 18336 | 0.580 | 15 | 0.002 | 413343 | 3.50E-08 | Adenocarcinoma |
| Chronotype | rs4936291 | G | A | G | A | -0.014 | -0.015 | 0.389 | 0.373 | FALSE | 11 | 0.030 | 18336 | 0.633 | 11 | 0.002 | 413343 | 2.10E-10 | Adenocarcinoma |
| Chronotype | rs4949980 | G | A | G | A | -0.023 | -0.060 | 0.067 | 0.070 | FALSE | 1 | 0.054 | 18336 | 0.322 | 1 | 0.004 | 413343 | 2.30E-08 | Adenocarcinoma |
| Chronotype | rs509476 | C | T | C | T | 0.088 | -0.151 | 0.970 | 0.975 | FALSE | 1 | 0.108 | 18336 | 0.140 | 1 | 0.006 | 413343 | 8.10E-49 | Adenocarcinoma |
| Chronotype | rs56076457 | T | C | T | C | -0.012 | 0.066 | 0.529 | 0.549 | FALSE | 18 | 0.026 | 18336 | 0.018 | 18 | 0.002 | 413343 | 8.90E-09 | Adenocarcinoma |
| Chronotype | rs56372114 | T | C | T | C | 0.012 | 0.029 | 0.384 | 0.399 | FALSE | 1 | 0.028 | 18336 | 0.309 | 1 | 0.002 | 413343 | 6.30E-09 | Adenocarcinoma |
| Chronotype | rs57435966 | T | C | T | C | 0.041 | -0.063 | 0.086 | 0.081 | FALSE | 2 | 0.047 | 18336 | 0.229 | 2 | 0.004 | 413343 | 8.50E-30 | Adenocarcinoma |
| Chronotype | rs57994353 | C | T | C | T | -0.013 | 0.057 | 0.299 | 0.297 | FALSE | 9 | 0.032 | 18336 | 0.067 | 9 | 0.002 | 413343 | 7.80E-09 | Adenocarcinoma |
| Chronotype | rs610590 | G | C | G | C | -0.015 | 0.075 | 0.212 | 0.208 | FALSE | 6 | 0.036 | 18336 | 0.032 | 6 | 0.002 | 413343 | 4.60E-09 | Adenocarcinoma |
| Chronotype | rs6131942 | G | A | G | A | -0.014 | 0.004 | 0.580 | 0.593 | FALSE | 20 | 0.028 | 18336 | 0.883 | 20 | 0.002 | 413343 | 4.50E-12 | Adenocarcinoma |
| Chronotype | rs61773390 | T | G | T | G | -0.026 | 0.038 | 0.195 | 0.187 | FALSE | 1 | 0.036 | 18336 | 0.284 | 1 | 0.003 | 413343 | 1.30E-23 | Adenocarcinoma |
| Chronotype | rs62082401 | G | C | G | C | -0.020 | 0.056 | 0.191 | 0.207 | FALSE | 18 | 0.036 | 18336 | 0.111 | 18 | 0.003 | 413343 | 9.60E-15 | Adenocarcinoma |
| Chronotype | rs62182115 | T | C | T | C | 0.013 | 0.002 | 0.342 | 0.336 | FALSE | 2 | 0.029 | 18336 | 0.942 | 2 | 0.002 | 413343 | 5.50E-10 | Adenocarcinoma |
| Chronotype | rs62553781 | T | C | T | C | 0.038 | -0.065 | 0.035 | 0.029 | FALSE | 9 | 0.075 | 18336 | 0.458 | 9 | 0.006 | 413343 | 1.10E-11 | Adenocarcinoma |
| Chronotype | rs6441169 | A | G | A | G | -0.016 | -0.019 | 0.856 | 0.869 | FALSE | 3 | 0.040 | 18336 | 0.635 | 3 | 0.003 | 413343 | 2.30E-08 | Adenocarcinoma |
| Chronotype | rs6442446 | G | A | G | A | 0.013 | 0.042 | 0.709 | 0.705 | FALSE | 3 | 0.028 | 18336 | 0.170 | 3 | 0.002 | 413343 | 7.20E-09 | Adenocarcinoma |
| Chronotype | rs6504758 | G | A | G | A | -0.012 | -0.007 | 0.536 | 0.563 | FALSE | 17 | 0.028 | 18336 | 0.803 | 17 | 0.002 | 413343 | 1.20E-09 | Adenocarcinoma |
| Chronotype | rs6601686 | T | A | T | A | 0.014 | 0.043 | 0.410 | 0.409 | FALSE | 8 | 0.029 | 18336 | 0.130 | 8 | 0.002 | 413343 | 4.60E-11 | Adenocarcinoma |
| Chronotype | rs6658041 | A | G | A | G | -0.012 | 0.019 | 0.599 | 0.611 | FALSE | 1 | 0.027 | 18336 | 0.504 | 1 | 0.002 | 413343 | 7.50E-09 | Adenocarcinoma |
| Chronotype | rs66710942 | T | C | T | C | -0.013 | -0.035 | 0.592 | 0.569 | FALSE | 3 | 0.030 | 18336 | 0.237 | 3 | 0.002 | 413343 | 1.60E-10 | Adenocarcinoma |
| Chronotype | rs6718119 | G | A | G | A | -0.012 | 0.011 | 0.377 | 0.373 | FALSE | 2 | 0.029 | 18336 | 0.708 | 2 | 0.002 | 413343 | 1.10E-08 | Adenocarcinoma |
| Chronotype | rs67988891 | G | C | G | C | -0.019 | 0.024 | 0.319 | 0.317 | FALSE | 5 | 0.030 | 18336 | 0.418 | 5 | 0.002 | 413343 | 1.10E-17 | Adenocarcinoma |
| Chronotype | rs6967481 | T | C | T | C | -0.016 | 0.012 | 0.497 | 0.497 | FALSE | 7 | 0.027 | 18336 | 0.661 | 7 | 0.002 | 413343 | 3.00E-14 | Adenocarcinoma |
| Chronotype | rs698015 | T | C | T | C | -0.013 | 0.026 | 0.647 | 0.635 | FALSE | 14 | 0.027 | 18336 | 0.369 | 14 | 0.002 | 413343 | 1.20E-09 | Adenocarcinoma |
| Chronotype | rs7148842 | T | C | T | C | 0.012 | -0.008 | 0.386 | 0.383 | FALSE | 14 | 0.029 | 18336 | 0.799 | 14 | 0.002 | 413343 | 7.40E-09 | Adenocarcinoma |
| Chronotype | rs72632979 | G | A | G | A | 0.017 | 0.055 | 0.172 | 0.161 | FALSE | 11 | 0.040 | 18336 | 0.157 | 11 | 0.003 | 413343 | 9.20E-10 | Adenocarcinoma |
| Chronotype | rs72720396 | G | A | G | A | -0.021 | -0.027 | 0.230 | 0.213 | FALSE | 1 | 0.033 | 18336 | 0.448 | 1 | 0.002 | 413343 | 1.30E-17 | Adenocarcinoma |
| Chronotype | rs7304278 | G | A | G | A | -0.015 | 0.027 | 0.724 | 0.696 | FALSE | 12 | 0.029 | 18336 | 0.390 | 12 | 0.002 | 413343 | 1.10E-10 | Adenocarcinoma |
| Chronotype | rs74097630 | T | G | T | G | 0.018 | 0.020 | 0.141 | 0.143 | FALSE | 12 | 0.040 | 18336 | 0.621 | 12 | 0.003 | 413343 | 4.80E-10 | Adenocarcinoma |
| Chronotype | rs74357745 | G | A | G | A | 0.020 | 0.061 | 0.121 | 0.125 | FALSE | 11 | 0.044 | 18336 | 0.153 | 11 | 0.003 | 413343 | 9.60E-11 | Adenocarcinoma |
| Chronotype | rs7547493 | G | A | G | A | -0.028 | 0.020 | 0.177 | 0.170 | FALSE | 1 | 0.037 | 18336 | 0.592 | 1 | 0.003 | 413343 | 3.70E-26 | Adenocarcinoma |
| Chronotype | rs76223855 | C | T | C | T | -0.080 | -0.089 | 0.011 | 0.012 | FALSE | 6 | 0.104 | 18336 | 0.489 | 6 | 0.010 | 413343 | 2.70E-16 | Adenocarcinoma |
| Chronotype | rs7626349 | C | T | C | T | -0.013 | 0.042 | 0.706 | 0.696 | FALSE | 3 | 0.028 | 18336 | 0.170 | 3 | 0.002 | 413343 | 1.90E-08 | Adenocarcinoma |
| Chronotype | rs7652260 | G | C | G | C | 0.016 | 0.010 | 0.162 | 0.160 | FALSE | 3 | 0.040 | 18336 | 0.816 | 3 | 0.003 | 413343 | 8.10E-09 | Adenocarcinoma |
| Chronotype | rs769066 | C | T | C | T | -0.017 | -0.012 | 0.184 | 0.192 | FALSE | 8 | 0.034 | 18336 | 0.738 | 8 | 0.003 | 413343 | 3.20E-10 | Adenocarcinoma |
| Chronotype | rs7783012 | A | G | A | G | -0.012 | 0.017 | 0.591 | 0.557 | FALSE | 7 | 0.027 | 18336 | 0.542 | 7 | 0.002 | 413343 | 5.20E-09 | Adenocarcinoma |
| Chronotype | rs78095690 | C | T | C | T | -0.011 | 0.002 | 0.437 | 0.442 | FALSE | 20 | 0.028 | 18336 | 0.952 | 20 | 0.002 | 413343 | 2.40E-08 | Adenocarcinoma |
| Chronotype | rs7959983 | C | T | C | T | -0.014 | 0.035 | 0.405 | 0.410 | FALSE | 12 | 0.029 | 18336 | 0.222 | 12 | 0.002 | 413343 | 9.00E-12 | Adenocarcinoma |
| Chronotype | rs80097534 | T | G | T | G | 0.022 | -0.075 | 0.098 | 0.114 | FALSE | 12 | 0.042 | 18336 | 0.115 | 12 | 0.003 | 413343 | 1.00E-10 | Adenocarcinoma |
| Chronotype | rs812925 | G | C | G | C | -0.015 | -0.010 | 0.352 | 0.364 | FALSE | 2 | 0.028 | 18336 | 0.741 | 2 | 0.002 | 413343 | 1.10E-12 | Adenocarcinoma |
| Chronotype | rs848552 | G | C | G | C | -0.012 | -0.038 | 0.528 | 0.538 | FALSE | 2 | 0.029 | 18336 | 0.178 | 2 | 0.002 | 413343 | 1.40E-09 | Adenocarcinoma |
| Chronotype | rs9291813 | C | T | C | T | -0.013 | 0.000 | 0.761 | 0.769 | FALSE | 5 | 0.032 | 18336 | 0.994 | 5 | 0.002 | 413343 | 1.90E-08 | Adenocarcinoma |
| Chronotype | rs9348050 | C | T | C | T | 0.012 | 0.015 | 0.511 | 0.520 | FALSE | 6 | 0.027 | 18336 | 0.586 | 6 | 0.002 | 413343 | 9.50E-10 | Adenocarcinoma |
| Chronotype | rs9395520 | T | C | T | C | -0.017 | 0.017 | 0.304 | 0.317 | FALSE | 6 | 0.030 | 18336 | 0.586 | 6 | 0.002 | 413343 | 4.10E-15 | Adenocarcinoma |
| Chronotype | rs9476310 | T | C | T | C | -0.013 | -0.047 | 0.511 | 0.512 | FALSE | 6 | 0.030 | 18336 | 0.113 | 6 | 0.002 | 413343 | 6.50E-10 | Adenocarcinoma |
| Chronotype | rs9573971 | G | A | G | A | 0.053 | 0.016 | 0.034 | 0.032 | FALSE | 13 | 0.074 | 18336 | 0.837 | 13 | 0.006 | 413343 | 9.70E-21 | Adenocarcinoma |
| Chronotype | rs9597250 | A | C | A | C | 0.016 | 0.052 | 0.189 | 0.192 | FALSE | 13 | 0.036 | 18336 | 0.145 | 13 | 0.003 | 413343 | 6.50E-10 | Adenocarcinoma |
| Chronotype | rs9795439 | G | A | G | A | 0.015 | 0.037 | 0.804 | 0.794 | FALSE | 11 | 0.035 | 18336 | 0.337 | 11 | 0.003 | 413343 | 7.90E-09 | Adenocarcinoma |
| Chronotype | rs9831488 | G | A | G | A | -0.014 | 0.077 | 0.353 | 0.349 | FALSE | 3 | 0.031 | 18336 | 0.010 | 3 | 0.002 | 413343 | 1.10E-10 | Adenocarcinoma |
| Chronotype | rs9932577 | A | C | A | C | 0.013 | 0.028 | 0.506 | 0.516 | FALSE | 16 | 0.028 | 18336 | 0.347 | 16 | 0.002 | 413343 | 7.40E-10 | Adenocarcinoma |
| Chronotype | rs9962650 | G | C | G | C | -0.014 | 0.019 | 0.423 | 0.415 | FALSE | 18 | 0.028 | 18336 | 0.505 | 18 | 0.002 | 413343 | 1.50E-11 | Adenocarcinoma |
| Chronotype | rs9964420 | A | C | A | C | 0.021 | 0.017 | 0.303 | 0.283 | FALSE | 18 | 0.031 | 18336 | 0.589 | 18 | 0.002 | 413343 | 4.40E-22 | Adenocarcinoma |
| Getting up in morning | rs1017168 | C | A | C | A | 0.010 | 0.000 | 0.644 | 0.640 | FALSE | 12 | 0.029 | 18336 | 0.994 | 12 | 0.002 | 461658 | 3.60E-10 | Adenocarcinoma |
| Getting up in morning | rs10175975 | T | C | T | C | 0.012 | 0.017 | 0.182 | 0.191 | FALSE | 2 | 0.035 | 18336 | 0.628 | 2 | 0.002 | 461658 | 3.70E-09 | Adenocarcinoma |
| Getting up in morning | rs10455248 | T | C | T | C | -0.010 | -0.007 | 0.282 | 0.285 | FALSE | 6 | 0.030 | 18336 | 0.814 | 6 | 0.002 | 461658 | 2.60E-08 | Adenocarcinoma |
| Getting up in morning | rs10462020 | G | T | G | T | 0.017 | 0.039 | 0.196 | 0.187 | FALSE | 1 | 0.036 | 18336 | 0.278 | 1 | 0.002 | 461658 | 1.90E-16 | Adenocarcinoma |
| Getting up in morning | rs10518446 | C | G | C | G | 0.020 | 0.027 | 0.163 | 0.158 | FALSE | 1 | 0.038 | 18336 | 0.474 | 1 | 0.002 | 461658 | 9.80E-20 | Adenocarcinoma |
| Getting up in morning | rs10779704 | C | A | C | A | 0.010 | -0.015 | 0.625 | 0.613 | FALSE | 1 | 0.029 | 18336 | 0.613 | 1 | 0.002 | 461658 | 9.90E-09 | Adenocarcinoma |
| Getting up in morning | rs11075924 | A | C | A | C | -0.010 | -0.028 | 0.496 | 0.493 | FALSE | 16 | 0.027 | 18336 | 0.318 | 16 | 0.002 | 461658 | 5.00E-10 | Adenocarcinoma |
| Getting up in morning | rs112613078 | G | A | G | A | 0.012 | -0.031 | 0.191 | 0.191 | FALSE | 7 | 0.038 | 18336 | 0.440 | 7 | 0.002 | 461658 | 7.50E-10 | Adenocarcinoma |
| Getting up in morning | rs113232113 | T | A | T | A | -0.014 | -0.017 | 0.117 | 0.125 | FALSE | 19 | 0.040 | 18336 | 0.688 | 19 | 0.002 | 461658 | 2.30E-08 | Adenocarcinoma |
| Getting up in morning | rs114443104 | A | G | A | G | 0.019 | -0.049 | 0.058 | 0.051 | FALSE | 2 | 0.062 | 18336 | 0.481 | 2 | 0.003 | 461658 | 2.70E-08 | Adenocarcinoma |
| Getting up in morning | rs11629621 | G | C | G | C | -0.012 | 0.026 | 0.577 | 0.575 | FALSE | 15 | 0.027 | 18336 | 0.368 | 15 | 0.002 | 461658 | 2.10E-13 | Adenocarcinoma |
| Getting up in morning | rs11669535 | C | T | C | T | 0.011 | -0.045 | 0.217 | 0.177 | FALSE | 19 | 0.037 | 18336 | 0.267 | 19 | 0.002 | 461658 | 4.60E-08 | Adenocarcinoma |
| Getting up in morning | rs11672103 | T | C | T | C | -0.009 | 0.005 | 0.553 | 0.538 | FALSE | 19 | 0.028 | 18336 | 0.869 | 19 | 0.002 | 461658 | 6.70E-09 | Adenocarcinoma |
| Getting up in morning | rs12044778 | A | G | A | G | -0.012 | -0.065 | 0.180 | 0.179 | FALSE | 1 | 0.033 | 18336 | 0.076 | 1 | 0.002 | 461658 | 2.20E-08 | Adenocarcinoma |
| Getting up in morning | rs12227309 | T | C | T | C | 0.012 | -0.022 | 0.247 | 0.250 | FALSE | 12 | 0.031 | 18336 | 0.510 | 12 | 0.002 | 461658 | 1.50E-10 | Adenocarcinoma |
| Getting up in morning | rs1223149 | C | T | C | T | 0.014 | -0.030 | 0.804 | 0.809 | FALSE | 2 | 0.035 | 18336 | 0.398 | 2 | 0.002 | 461658 | 1.30E-12 | Adenocarcinoma |
| Getting up in morning | rs12326675 | G | C | G | C | 0.012 | 0.059 | 0.193 | 0.205 | FALSE | 18 | 0.039 | 18336 | 0.123 | 18 | 0.002 | 461658 | 4.20E-08 | Adenocarcinoma |
| Getting up in morning | rs12523700 | A | T | A | T | 0.014 | 0.044 | 0.118 | 0.097 | FALSE | 6 | 0.051 | 18336 | 0.398 | 6 | 0.003 | 461658 | 8.40E-09 | Adenocarcinoma |
| Getting up in morning | rs12601968 | T | G | T | G | -0.011 | 0.045 | 0.327 | 0.311 | FALSE | 17 | 0.031 | 18336 | 0.135 | 17 | 0.002 | 461658 | 8.90E-11 | Adenocarcinoma |
| Getting up in morning | rs12736689 | C | T | C | T | 0.053 | 0.128 | 0.030 | 0.026 | FALSE | 1 | 0.100 | 18336 | 0.185 | 1 | 0.005 | 461658 | 3.70E-29 | Adenocarcinoma |
| Getting up in morning | rs12752290 | C | T | C | T | 0.012 | 0.016 | 0.442 | 0.449 | FALSE | 1 | 0.029 | 18336 | 0.589 | 1 | 0.002 | 461658 | 5.70E-13 | Adenocarcinoma |
| Getting up in morning | rs12969848 | T | C | T | C | 0.010 | 0.021 | 0.529 | 0.534 | FALSE | 18 | 0.027 | 18336 | 0.458 | 18 | 0.002 | 461658 | 2.90E-09 | Adenocarcinoma |
| Getting up in morning | rs13116306 | T | C | T | C | -0.010 | 0.027 | 0.421 | 0.421 | FALSE | 4 | 0.029 | 18336 | 0.342 | 4 | 0.002 | 461658 | 4.00E-09 | Adenocarcinoma |
| Getting up in morning | rs13155750 | G | A | G | A | -0.012 | 0.039 | 0.234 | 0.228 | FALSE | 5 | 0.034 | 18336 | 0.241 | 5 | 0.002 | 461658 | 1.30E-10 | Adenocarcinoma |
| Getting up in morning | rs133067 | T | C | T | C | 0.011 | 0.028 | 0.791 | 0.813 | FALSE | 22 | 0.034 | 18336 | 0.431 | 22 | 0.002 | 461658 | 1.50E-08 | Adenocarcinoma |
| Getting up in morning | rs1333536 | T | C | T | C | -0.009 | 0.019 | 0.385 | 0.368 | FALSE | 13 | 0.029 | 18336 | 0.511 | 13 | 0.002 | 461658 | 2.30E-08 | Adenocarcinoma |
| Getting up in morning | rs141391319 | A | G | A | G | 0.035 | 0.069 | 0.032 | 0.033 | FALSE | 1 | 0.079 | 18336 | 0.387 | 1 | 0.005 | 461658 | 1.60E-14 | Adenocarcinoma |
| Getting up in morning | rs1420607 | A | G | A | G | 0.012 | -0.020 | 0.272 | 0.275 | FALSE | 16 | 0.030 | 18336 | 0.534 | 16 | 0.002 | 461658 | 1.30E-11 | Adenocarcinoma |
| Getting up in morning | rs1421085 | C | T | C | T | 0.011 | -0.015 | 0.403 | 0.417 | FALSE | 16 | 0.028 | 18336 | 0.597 | 16 | 0.002 | 461658 | 1.60E-11 | Adenocarcinoma |
| Getting up in morning | rs145831787 | T | C | T | C | -0.017 | -0.001 | 0.113 | 0.109 | FALSE | 3 | 0.043 | 18336 | 0.988 | 3 | 0.003 | 461658 | 1.60E-11 | Adenocarcinoma |
| Getting up in morning | rs1470503 | T | C | T | C | -0.013 | 0.034 | 0.428 | 0.451 | FALSE | 2 | 0.029 | 18336 | 0.233 | 2 | 0.002 | 461658 | 1.90E-15 | Adenocarcinoma |
| Getting up in morning | rs1606803 | T | C | T | C | 0.011 | 0.035 | 0.287 | 0.279 | FALSE | 2 | 0.031 | 18336 | 0.266 | 2 | 0.002 | 461658 | 9.50E-11 | Adenocarcinoma |
| Getting up in morning | rs17112198 | G | A | G | A | 0.010 | 0.020 | 0.299 | 0.267 | FALSE | 14 | 0.032 | 18336 | 0.524 | 14 | 0.002 | 461658 | 5.30E-09 | Adenocarcinoma |
| Getting up in morning | rs17152364 | G | A | G | A | -0.012 | 0.075 | 0.301 | 0.314 | FALSE | 11 | 0.032 | 18336 | 0.013 | 11 | 0.002 | 461658 | 1.40E-11 | Adenocarcinoma |
| Getting up in morning | rs17464772 | A | G | A | G | 0.013 | -0.011 | 0.352 | 0.316 | FALSE | 12 | 0.030 | 18336 | 0.713 | 12 | 0.002 | 461658 | 6.90E-14 | Adenocarcinoma |
| Getting up in morning | rs17716502 | T | C | T | C | 0.011 | 0.049 | 0.204 | 0.184 | FALSE | 8 | 0.037 | 18336 | 0.181 | 8 | 0.002 | 461658 | 4.10E-08 | Adenocarcinoma |
| Getting up in morning | rs17766755 | A | G | A | G | -0.009 | 0.009 | 0.360 | 0.355 | FALSE | 14 | 0.029 | 18336 | 0.765 | 14 | 0.002 | 461658 | 1.30E-08 | Adenocarcinoma |
| Getting up in morning | rs17777135 | A | G | A | G | 0.012 | -0.006 | 0.200 | 0.199 | FALSE | 2 | 0.036 | 18336 | 0.870 | 2 | 0.002 | 461658 | 1.10E-08 | Adenocarcinoma |
| Getting up in morning | rs1854558 | A | G | A | G | -0.010 | -0.037 | 0.262 | 0.250 | FALSE | 9 | 0.031 | 18336 | 0.265 | 9 | 0.002 | 461658 | 7.90E-09 | Adenocarcinoma |
| Getting up in morning | rs1914397 | A | T | A | T | 0.010 | 0.017 | 0.448 | 0.443 | FALSE | 7 | 0.028 | 18336 | 0.539 | 7 | 0.002 | 461658 | 1.10E-10 | Adenocarcinoma |
| Getting up in morning | rs2044742 | A | G | A | G | -0.013 | -0.003 | 0.132 | 0.125 | FALSE | 8 | 0.040 | 18336 | 0.949 | 8 | 0.002 | 461658 | 1.30E-08 | Adenocarcinoma |
| Getting up in morning | rs2360802 | T | A | T | A | 0.013 | 0.001 | 0.225 | 0.230 | FALSE | 8 | 0.032 | 18336 | 0.965 | 8 | 0.002 | 461658 | 2.10E-11 | Adenocarcinoma |
| Getting up in morning | rs2653355 | A | C | A | C | -0.024 | -0.047 | 0.823 | 0.843 | FALSE | 6 | 0.039 | 18336 | 0.228 | 6 | 0.002 | 461658 | 1.60E-29 | Adenocarcinoma |
| Getting up in morning | rs2971970 | G | T | G | T | -0.011 | 0.002 | 0.782 | 0.786 | FALSE | 7 | 0.033 | 18336 | 0.957 | 7 | 0.002 | 461658 | 3.20E-08 | Adenocarcinoma |
| Getting up in morning | rs3125735 | T | C | T | C | -0.012 | 0.083 | 0.199 | 0.200 | FALSE | 13 | 0.037 | 18336 | 0.018 | 13 | 0.002 | 461658 | 1.90E-09 | Adenocarcinoma |
| Getting up in morning | rs34757401 | G | A | G | A | 0.011 | -0.007 | 0.227 | 0.225 | FALSE | 14 | 0.033 | 18336 | 0.844 | 14 | 0.002 | 461658 | 1.80E-08 | Adenocarcinoma |
| Getting up in morning | rs3760185 | T | C | T | C | -0.012 | -0.019 | 0.248 | 0.237 | FALSE | 17 | 0.035 | 18336 | 0.609 | 17 | 0.002 | 461658 | 1.10E-10 | Adenocarcinoma |
| Getting up in morning | rs3766163 | C | T | C | T | 0.010 | -0.017 | 0.273 | 0.288 | FALSE | 1 | 0.030 | 18336 | 0.587 | 1 | 0.002 | 461658 | 6.70E-09 | Adenocarcinoma |
| Getting up in morning | rs406952 | C | T | C | T | 0.011 | 0.012 | 0.376 | 0.372 | FALSE | 2 | 0.029 | 18336 | 0.691 | 2 | 0.002 | 461658 | 1.50E-11 | Adenocarcinoma |
| Getting up in morning | rs4483990 | C | A | C | A | -0.018 | -0.067 | 0.157 | 0.149 | FALSE | 2 | 0.035 | 18336 | 0.087 | 2 | 0.002 | 461658 | 7.10E-17 | Adenocarcinoma |
| Getting up in morning | rs45510091 | G | A | G | A | 0.020 | -0.064 | 0.055 | 0.047 | FALSE | 4 | 0.060 | 18336 | 0.347 | 4 | 0.004 | 461658 | 5.70E-09 | Adenocarcinoma |
| Getting up in morning | rs4790352 | A | G | A | G | 0.018 | -0.029 | 0.918 | 0.920 | FALSE | 17 | 0.051 | 18336 | 0.574 | 17 | 0.003 | 461658 | 8.80E-10 | Adenocarcinoma |
| Getting up in morning | rs4958317 | A | G | A | G | 0.015 | 0.018 | 0.289 | 0.274 | FALSE | 5 | 0.031 | 18336 | 0.563 | 5 | 0.002 | 461658 | 1.20E-16 | Adenocarcinoma |
| Getting up in morning | rs6141724 | G | T | G | T | -0.010 | 0.061 | 0.370 | 0.393 | FALSE | 20 | 0.031 | 18336 | 0.044 | 20 | 0.002 | 461658 | 2.30E-09 | Adenocarcinoma |
| Getting up in morning | rs61926781 | T | C | T | C | -0.019 | -0.093 | 0.059 | 0.051 | FALSE | 12 | 0.063 | 18336 | 0.209 | 12 | 0.003 | 461658 | 8.90E-09 | Adenocarcinoma |
| Getting up in morning | rs620598 | G | A | G | A | 0.011 | 0.057 | 0.226 | 0.221 | FALSE | 6 | 0.035 | 18336 | 0.096 | 6 | 0.002 | 461658 | 7.40E-09 | Adenocarcinoma |
| Getting up in morning | rs627685 | C | T | C | T | 0.010 | 0.047 | 0.304 | 0.304 | FALSE | 18 | 0.031 | 18336 | 0.120 | 18 | 0.002 | 461658 | 2.80E-09 | Adenocarcinoma |
| Getting up in morning | rs6745423 | A | T | A | T | -0.013 | -0.004 | 0.722 | 0.727 | FALSE | 2 | 0.030 | 18336 | 0.886 | 2 | 0.002 | 461658 | 6.10E-13 | Adenocarcinoma |
| Getting up in morning | rs7105482 | G | A | G | A | 0.009 | 0.009 | 0.395 | 0.422 | FALSE | 11 | 0.028 | 18336 | 0.760 | 11 | 0.002 | 461658 | 4.40E-08 | Adenocarcinoma |
| Getting up in morning | rs7144028 | C | A | C | A | -0.009 | -0.025 | 0.499 | 0.505 | FALSE | 14 | 0.028 | 18336 | 0.384 | 14 | 0.002 | 461658 | 1.50E-08 | Adenocarcinoma |
| Getting up in morning | rs7206027 | T | A | T | A | -0.010 | -0.050 | 0.386 | 0.395 | FALSE | 16 | 0.026 | 18336 | 0.078 | 16 | 0.002 | 461658 | 2.80E-09 | Adenocarcinoma |
| Getting up in morning | rs72895663 | G | A | G | A | 0.011 | 0.081 | 0.231 | 0.204 | FALSE | 6 | 0.037 | 18336 | 0.023 | 6 | 0.002 | 461658 | 5.80E-09 | Adenocarcinoma |
| Getting up in morning | rs73179222 | A | G | A | G | 0.010 | -0.021 | 0.372 | 0.357 | FALSE | 8 | 0.028 | 18336 | 0.472 | 8 | 0.002 | 461658 | 1.10E-09 | Adenocarcinoma |
| Getting up in morning | rs73608603 | G | A | G | A | -0.013 | 0.055 | 0.131 | 0.140 | FALSE | 11 | 0.041 | 18336 | 0.176 | 11 | 0.002 | 461658 | 1.30E-08 | Adenocarcinoma |
| Getting up in morning | rs74555583 | A | G | A | G | -0.017 | -0.056 | 0.085 | 0.095 | FALSE | 20 | 0.047 | 18336 | 0.285 | 20 | 0.003 | 461658 | 2.00E-09 | Adenocarcinoma |
| Getting up in morning | rs77556405 | A | G | A | G | 0.016 | 0.000 | 0.172 | 0.171 | FALSE | 17 | 0.037 | 18336 | 0.994 | 17 | 0.002 | 461658 | 1.50E-14 | Adenocarcinoma |
| Getting up in morning | rs77556698 | T | G | T | G | -0.012 | 0.001 | 0.216 | 0.209 | FALSE | 14 | 0.033 | 18336 | 0.981 | 14 | 0.002 | 461658 | 1.40E-09 | Adenocarcinoma |
| Getting up in morning | rs7833021 | T | C | T | C | 0.011 | 0.069 | 0.773 | 0.776 | FALSE | 8 | 0.031 | 18336 | 0.043 | 8 | 0.002 | 461658 | 3.30E-09 | Adenocarcinoma |
| Getting up in morning | rs7899208 | C | T | C | T | 0.014 | 0.101 | 0.874 | 0.863 | FALSE | 10 | 0.036 | 18336 | 0.014 | 10 | 0.002 | 461658 | 1.50E-08 | Adenocarcinoma |
| Getting up in morning | rs9399613 | T | C | T | C | -0.011 | -0.047 | 0.289 | 0.295 | FALSE | 6 | 0.029 | 18336 | 0.129 | 6 | 0.002 | 461658 | 5.20E-10 | Adenocarcinoma |
| Getting up in morning | rs9573982 | C | T | C | T | -0.021 | -0.048 | 0.051 | 0.050 | FALSE | 13 | 0.058 | 18336 | 0.460 | 13 | 0.004 | 461658 | 1.20E-08 | Adenocarcinoma |
| Getting up in morning | rs9644465 | A | G | A | G | 0.012 | -0.069 | 0.212 | 0.200 | FALSE | 8 | 0.032 | 18336 | 0.050 | 8 | 0.002 | 461658 | 3.00E-09 | Adenocarcinoma |
| Sleep duration | rs10510128 | A | G | A | G | 0.011 | -0.025 | 0.208 | 0.224 | FALSE | 10 | 0.032 | 18336 | 0.456 | 10 | 0.002 | 460099 | 7.70E-09 | Adenocarcinoma |
| Sleep duration | rs11039216 | T | C | T | C | -0.010 | 0.032 | 0.533 | 0.488 | FALSE | 11 | 0.028 | 18336 | 0.273 | 11 | 0.002 | 460099 | 1.50E-10 | Adenocarcinoma |
| Sleep duration | rs113021516 | C | G | C | G | 0.011 | -0.007 | 0.336 | 0.336 | FALSE | 3 | 0.029 | 18336 | 0.816 | 3 | 0.002 | 460099 | 1.30E-11 | Adenocarcinoma |
| Sleep duration | rs113113059 | C | T | C | T | -0.011 | 0.014 | 0.220 | 0.214 | FALSE | 6 | 0.034 | 18336 | 0.689 | 6 | 0.002 | 460099 | 8.60E-09 | Adenocarcinoma |
| Sleep duration | rs11621908 | T | C | T | C | -0.020 | -0.009 | 0.083 | 0.087 | FALSE | 14 | 0.048 | 18336 | 0.865 | 14 | 0.003 | 460099 | 1.10E-11 | Adenocarcinoma |
| Sleep duration | rs11643715 | G | C | G | C | 0.011 | 0.018 | 0.293 | 0.293 | FALSE | 16 | 0.030 | 18336 | 0.554 | 16 | 0.002 | 460099 | 5.00E-10 | Adenocarcinoma |
| Sleep duration | rs11650677 | A | G | A | G | 0.011 | 0.018 | 0.339 | 0.355 | FALSE | 17 | 0.029 | 18336 | 0.544 | 17 | 0.002 | 460099 | 3.80E-11 | Adenocarcinoma |
| Sleep duration | rs11982852 | T | C | T | C | -0.012 | -0.019 | 0.244 | 0.240 | FALSE | 7 | 0.032 | 18336 | 0.572 | 7 | 0.002 | 460099 | 3.10E-10 | Adenocarcinoma |
| Sleep duration | rs12518468 | C | T | C | T | -0.011 | 0.058 | 0.329 | 0.322 | FALSE | 5 | 0.031 | 18336 | 0.057 | 5 | 0.002 | 460099 | 4.10E-10 | Adenocarcinoma |
| Sleep duration | rs12567114 | A | G | A | G | 0.012 | -0.004 | 0.276 | 0.275 | FALSE | 1 | 0.031 | 18336 | 0.900 | 1 | 0.002 | 460099 | 6.10E-12 | Adenocarcinoma |
| Sleep duration | rs13107325 | T | C | T | C | -0.024 | 0.024 | 0.075 | 0.080 | FALSE | 4 | 0.051 | 18336 | 0.649 | 4 | 0.003 | 460099 | 1.40E-15 | Adenocarcinoma |
| Sleep duration | rs1348047 | T | G | T | G | -0.013 | 0.051 | 0.267 | 0.256 | FALSE | 18 | 0.033 | 18336 | 0.116 | 18 | 0.002 | 460099 | 3.80E-12 | Adenocarcinoma |
| Sleep duration | rs1463053 | A | G | A | G | 0.009 | -0.010 | 0.640 | 0.616 | FALSE | 1 | 0.029 | 18336 | 0.726 | 1 | 0.002 | 460099 | 2.40E-08 | Adenocarcinoma |
| Sleep duration | rs151014368 | A | G | A | G | 0.011 | -0.008 | 0.207 | 0.224 | FALSE | 5 | 0.034 | 18336 | 0.812 | 5 | 0.002 | 460099 | 1.00E-08 | Adenocarcinoma |
| Sleep duration | rs1517572 | C | A | C | A | 0.012 | -0.065 | 0.581 | 0.575 | FALSE | 11 | 0.030 | 18336 | 0.025 | 11 | 0.002 | 460099 | 6.50E-13 | Adenocarcinoma |
| Sleep duration | rs1553132 | G | A | G | A | 0.011 | -0.013 | 0.259 | 0.250 | FALSE | 11 | 0.031 | 18336 | 0.691 | 11 | 0.002 | 460099 | 8.10E-09 | Adenocarcinoma |
| Sleep duration | rs17391944 | G | T | G | T | 0.022 | -0.022 | 0.050 | 0.057 | FALSE | 9 | 0.060 | 18336 | 0.732 | 9 | 0.004 | 460099 | 4.40E-09 | Adenocarcinoma |
| Sleep duration | rs174564 | G | A | G | A | 0.010 | -0.088 | 0.349 | 0.328 | FALSE | 11 | 0.027 | 18336 | 0.004 | 11 | 0.002 | 460099 | 6.30E-09 | Adenocarcinoma |
| Sleep duration | rs1939455 | T | G | T | G | -0.016 | -0.035 | 0.120 | 0.112 | FALSE | 11 | 0.044 | 18336 | 0.458 | 11 | 0.003 | 460099 | 3.50E-10 | Adenocarcinoma |
| Sleep duration | rs1972712 | C | T | C | T | 0.012 | 0.018 | 0.249 | 0.258 | FALSE | 2 | 0.032 | 18336 | 0.577 | 2 | 0.002 | 460099 | 1.70E-10 | Adenocarcinoma |
| Sleep duration | rs2072727 | C | T | C | T | -0.009 | -0.039 | 0.565 | 0.570 | FALSE | 20 | 0.029 | 18336 | 0.176 | 20 | 0.002 | 460099 | 9.30E-09 | Adenocarcinoma |
| Sleep duration | rs2079070 | G | C | G | C | -0.013 | 0.048 | 0.735 | 0.721 | FALSE | 7 | 0.029 | 18336 | 0.131 | 7 | 0.002 | 460099 | 1.20E-13 | Adenocarcinoma |
| Sleep duration | rs2186122 | T | A | T | A | -0.011 | 0.015 | 0.560 | 0.524 | FALSE | 1 | 0.027 | 18336 | 0.592 | 1 | 0.002 | 460099 | 2.30E-11 | Adenocarcinoma |
| Sleep duration | rs2192528 | G | A | G | A | -0.010 | 0.024 | 0.522 | 0.526 | FALSE | 4 | 0.027 | 18336 | 0.398 | 4 | 0.002 | 460099 | 9.10E-10 | Adenocarcinoma |
| Sleep duration | rs2236295 | T | G | T | G | -0.009 | 0.047 | 0.403 | 0.385 | FALSE | 10 | 0.030 | 18336 | 0.106 | 10 | 0.002 | 460099 | 2.90E-08 | Adenocarcinoma |
| Sleep duration | rs2279681 | G | C | G | C | 0.009 | -0.007 | 0.342 | 0.354 | FALSE | 1 | 0.028 | 18336 | 0.811 | 1 | 0.002 | 460099 | 3.40E-08 | Adenocarcinoma |
| Sleep duration | rs2683630 | G | C | G | C | 0.015 | 0.005 | 0.629 | 0.594 | FALSE | 2 | 0.028 | 18336 | 0.849 | 2 | 0.002 | 460099 | 1.70E-19 | Adenocarcinoma |
| Sleep duration | rs2734831 | G | T | G | T | -0.010 | -0.057 | 0.607 | 0.595 | FALSE | 11 | 0.030 | 18336 | 0.047 | 11 | 0.002 | 460099 | 2.20E-09 | Adenocarcinoma |
| Sleep duration | rs2748809 | C | T | C | T | -0.009 | -0.001 | 0.429 | 0.412 | FALSE | 14 | 0.030 | 18336 | 0.966 | 14 | 0.002 | 460099 | 1.90E-08 | Adenocarcinoma |
| Sleep duration | rs2839753 | C | T | C | T | -0.011 | 0.021 | 0.265 | 0.265 | FALSE | 4 | 0.032 | 18336 | 0.525 | 4 | 0.002 | 460099 | 4.40E-09 | Adenocarcinoma |
| Sleep duration | rs2863957 | A | C | A | C | 0.029 | -0.006 | 0.221 | 0.227 | FALSE | 2 | 0.032 | 18336 | 0.851 | 2 | 0.002 | 460099 | 9.60E-51 | Adenocarcinoma |
| Sleep duration | rs34354917 | A | C | A | C | -0.010 | -0.020 | 0.289 | 0.286 | FALSE | 12 | 0.035 | 18336 | 0.590 | 12 | 0.002 | 460099 | 1.40E-08 | Adenocarcinoma |
| Sleep duration | rs34786000 | T | G | T | G | 0.011 | 0.000 | 0.553 | 0.530 | FALSE | 19 | 0.029 | 18336 | 0.995 | 19 | 0.002 | 460099 | 1.70E-11 | Adenocarcinoma |
| Sleep duration | rs35126035 | C | A | C | A | -0.009 | 0.018 | 0.558 | 0.531 | FALSE | 19 | 0.029 | 18336 | 0.564 | 19 | 0.002 | 460099 | 2.20E-08 | Adenocarcinoma |
| Sleep duration | rs35662245 | A | T | A | T | 0.010 | 0.004 | 0.339 | 0.347 | FALSE | 2 | 0.029 | 18336 | 0.890 | 2 | 0.002 | 460099 | 1.90E-09 | Adenocarcinoma |
| Sleep duration | rs365663 | G | A | G | A | -0.009 | 0.009 | 0.455 | 0.473 | FALSE | 5 | 0.029 | 18336 | 0.762 | 5 | 0.002 | 460099 | 8.10E-09 | Adenocarcinoma |
| Sleep duration | rs374153 | T | C | T | C | -0.013 | 0.047 | 0.843 | 0.845 | FALSE | 2 | 0.036 | 18336 | 0.226 | 2 | 0.002 | 460099 | 2.50E-09 | Adenocarcinoma |
| Sleep duration | rs4767550 | G | A | G | A | 0.011 | 0.035 | 0.413 | 0.403 | FALSE | 12 | 0.029 | 18336 | 0.228 | 12 | 0.002 | 460099 | 2.70E-11 | Adenocarcinoma |
| Sleep duration | rs55658675 | T | C | T | C | -0.010 | -0.070 | 0.353 | 0.336 | FALSE | 14 | 0.027 | 18336 | 0.020 | 14 | 0.002 | 460099 | 7.10E-09 | Adenocarcinoma |
| Sleep duration | rs56367859 | G | A | G | A | 0.012 | 0.020 | 0.398 | 0.413 | FALSE | 16 | 0.028 | 18336 | 0.482 | 16 | 0.002 | 460099 | 1.20E-12 | Adenocarcinoma |
| Sleep duration | rs62444917 | C | A | C | A | 0.013 | -0.026 | 0.222 | 0.244 | FALSE | 7 | 0.032 | 18336 | 0.444 | 7 | 0.002 | 460099 | 1.70E-11 | Adenocarcinoma |
| Sleep duration | rs6561715 | A | T | A | T | 0.010 | -0.054 | 0.631 | 0.634 | FALSE | 13 | 0.030 | 18336 | 0.064 | 13 | 0.002 | 460099 | 3.90E-09 | Adenocarcinoma |
| Sleep duration | rs6681755 | A | G | A | G | 0.012 | -0.044 | 0.200 | 0.195 | FALSE | 1 | 0.033 | 18336 | 0.222 | 1 | 0.002 | 460099 | 9.00E-09 | Adenocarcinoma |
| Sleep duration | rs6783516 | T | G | T | G | -0.010 | 0.001 | 0.584 | 0.591 | FALSE | 3 | 0.028 | 18336 | 0.966 | 3 | 0.002 | 460099 | 1.60E-09 | Adenocarcinoma |
| Sleep duration | rs6889592 | A | G | A | G | 0.012 | -0.031 | 0.333 | 0.329 | FALSE | 5 | 0.028 | 18336 | 0.296 | 5 | 0.002 | 460099 | 4.10E-12 | Adenocarcinoma |
| Sleep duration | rs7016314 | C | T | C | T | 0.010 | 0.038 | 0.656 | 0.655 | FALSE | 8 | 0.029 | 18336 | 0.219 | 8 | 0.002 | 460099 | 3.10E-09 | Adenocarcinoma |
| Sleep duration | rs7115856 | C | A | C | A | 0.011 | 0.004 | 0.461 | 0.461 | FALSE | 11 | 0.028 | 18336 | 0.876 | 11 | 0.002 | 460099 | 1.50E-11 | Adenocarcinoma |
| Sleep duration | rs72771082 | G | A | G | A | 0.011 | -0.013 | 0.218 | 0.227 | FALSE | 16 | 0.032 | 18336 | 0.703 | 16 | 0.002 | 460099 | 1.40E-08 | Adenocarcinoma |
| Sleep duration | rs72831782 | A | T | A | T | -0.010 | 0.090 | 0.269 | 0.257 | FALSE | 2 | 0.039 | 18336 | 0.014 | 2 | 0.002 | 460099 | 3.40E-08 | Adenocarcinoma |
| Sleep duration | rs7517981 | C | T | C | T | -0.010 | 0.008 | 0.601 | 0.577 | FALSE | 1 | 0.028 | 18336 | 0.791 | 1 | 0.002 | 460099 | 1.10E-09 | Adenocarcinoma |
| Sleep duration | rs75539574 | C | A | C | A | 0.024 | 0.064 | 0.086 | 0.088 | FALSE | 2 | 0.053 | 18336 | 0.217 | 2 | 0.003 | 460099 | 1.80E-16 | Adenocarcinoma |
| Sleep duration | rs76258078 | G | A | G | A | -0.022 | -0.114 | 0.050 | 0.038 | FALSE | 3 | 0.069 | 18336 | 0.175 | 3 | 0.004 | 460099 | 3.80E-09 | Adenocarcinoma |
| Sleep duration | rs7644809 | C | T | C | T | -0.010 | -0.008 | 0.576 | 0.572 | FALSE | 3 | 0.028 | 18336 | 0.766 | 3 | 0.002 | 460099 | 4.10E-10 | Adenocarcinoma |
| Sleep duration | rs7711696 | T | G | T | G | -0.010 | 0.075 | 0.305 | 0.294 | FALSE | 5 | 0.032 | 18336 | 0.013 | 5 | 0.002 | 460099 | 1.30E-08 | Adenocarcinoma |
| Sleep duration | rs7740402 | G | T | G | T | -0.010 | -0.004 | 0.306 | 0.319 | FALSE | 6 | 0.029 | 18336 | 0.884 | 6 | 0.002 | 460099 | 4.10E-08 | Adenocarcinoma |
| Sleep duration | rs7831557 | A | G | A | G | -0.011 | 0.011 | 0.517 | 0.525 | FALSE | 8 | 0.027 | 18336 | 0.694 | 8 | 0.002 | 460099 | 4.20E-11 | Adenocarcinoma |
| Sleep duration | rs8038326 | G | A | G | A | -0.013 | 0.002 | 0.273 | 0.290 | FALSE | 15 | 0.030 | 18336 | 0.947 | 15 | 0.002 | 460099 | 8.40E-14 | Adenocarcinoma |
| Sleep duration | rs8047587 | T | G | T | G | -0.011 | -0.011 | 0.440 | 0.443 | FALSE | 16 | 0.028 | 18336 | 0.698 | 16 | 0.002 | 460099 | 8.30E-12 | Adenocarcinoma |
| Sleep duration | rs915416 | G | C | G | C | -0.013 | -0.064 | 0.709 | 0.689 | FALSE | 1 | 0.031 | 18336 | 0.034 | 1 | 0.002 | 460099 | 4.80E-13 | Adenocarcinoma |
| Sleep duration | rs9302680 | A | G | A | G | 0.012 | 0.006 | 0.439 | 0.463 | FALSE | 16 | 0.027 | 18336 | 0.827 | 16 | 0.002 | 460099 | 7.60E-14 | Adenocarcinoma |
| Sleep duration | rs9345234 | C | A | C | A | 0.009 | -0.032 | 0.578 | 0.558 | FALSE | 6 | 0.028 | 18336 | 0.254 | 6 | 0.002 | 460099 | 1.50E-08 | Adenocarcinoma |
| Sleep duration | rs9382445 | C | T | C | T | -0.009 | 0.006 | 0.375 | 0.357 | FALSE | 6 | 0.029 | 18336 | 0.832 | 6 | 0.002 | 460099 | 8.90E-09 | Adenocarcinoma |
| Sleep duration | rs9611007 | T | C | T | C | -0.014 | -0.023 | 0.142 | 0.149 | FALSE | 22 | 0.039 | 18336 | 0.581 | 22 | 0.002 | 460099 | 3.30E-09 | Adenocarcinoma |
| Sleep duration | rs9810474 | T | C | T | C | -0.011 | 0.039 | 0.232 | 0.228 | FALSE | 3 | 0.034 | 18336 | 0.249 | 3 | 0.002 | 460099 | 3.90E-09 | Adenocarcinoma |
| Sleep duration | rs9903898 | T | C | T | C | -0.009 | -0.011 | 0.489 | 0.485 | FALSE | 17 | 0.030 | 18336 | 0.726 | 17 | 0.002 | 460099 | 3.60E-09 | Adenocarcinoma |
| Sleeplessness | rs10838708 | A | G | A | G | -0.009 | -0.041 | 0.459 | 0.477 | FALSE | 11 | 0.027 | 18336 | 0.156 | 11 | 0.002 | 462341 | 2.90E-10 | Adenocarcinoma |
| Sleeplessness | rs11097861 | G | A | G | A | 0.010 | 0.047 | 0.716 | 0.706 | FALSE | 4 | 0.029 | 18336 | 0.133 | 4 | 0.002 | 462341 | 1.10E-09 | Adenocarcinoma |
| Sleeplessness | rs11152363 | A | G | A | G | 0.016 | 0.040 | 0.186 | 0.188 | FALSE | 18 | 0.036 | 18336 | 0.259 | 18 | 0.002 | 462341 | 4.50E-16 | Adenocarcinoma |
| Sleeplessness | rs113851554 | T | G | T | G | 0.047 | 0.046 | 0.057 | 0.064 | FALSE | 2 | 0.062 | 18336 | 0.464 | 2 | 0.003 | 462341 | 2.90E-45 | Adenocarcinoma |
| Sleeplessness | rs11635495 | C | T | C | T | 0.009 | 0.030 | 0.512 | 0.505 | FALSE | 15 | 0.027 | 18336 | 0.283 | 15 | 0.001 | 462341 | 2.80E-10 | Adenocarcinoma |
| Sleeplessness | rs11790060 | C | T | C | T | -0.010 | 0.018 | 0.331 | 0.343 | FALSE | 9 | 0.029 | 18336 | 0.539 | 9 | 0.002 | 462341 | 5.80E-11 | Adenocarcinoma |
| Sleeplessness | rs12049261 | C | G | C | G | 0.011 | -0.009 | 0.293 | 0.286 | FALSE | 1 | 0.030 | 18336 | 0.770 | 1 | 0.002 | 462341 | 6.80E-12 | Adenocarcinoma |
| Sleeplessness | rs12470989 | G | A | G | A | -0.010 | -0.029 | 0.204 | 0.200 | FALSE | 2 | 0.032 | 18336 | 0.390 | 2 | 0.002 | 462341 | 2.80E-08 | Adenocarcinoma |
| Sleeplessness | rs1430205 | T | C | T | C | 0.009 | 0.009 | 0.462 | 0.479 | FALSE | 5 | 0.027 | 18336 | 0.734 | 5 | 0.001 | 462341 | 2.10E-10 | Adenocarcinoma |
| Sleeplessness | rs1547630 | A | G | A | G | 0.009 | -0.041 | 0.652 | 0.631 | FALSE | 13 | 0.032 | 18336 | 0.191 | 13 | 0.002 | 462341 | 5.80E-09 | Adenocarcinoma |
| Sleeplessness | rs1592757 | C | G | C | G | 0.010 | -0.038 | 0.356 | 0.361 | FALSE | 5 | 0.028 | 18336 | 0.208 | 5 | 0.002 | 462341 | 4.30E-11 | Adenocarcinoma |
| Sleeplessness | rs17151854 | T | G | T | G | 0.013 | 0.019 | 0.152 | 0.149 | FALSE | 8 | 0.039 | 18336 | 0.639 | 8 | 0.002 | 462341 | 3.80E-10 | Adenocarcinoma |
| Sleeplessness | rs17709610 | G | A | G | A | -0.010 | -0.045 | 0.298 | 0.269 | FALSE | 10 | 0.029 | 18336 | 0.159 | 10 | 0.002 | 462341 | 9.50E-10 | Adenocarcinoma |
| Sleeplessness | rs1988337 | G | A | G | A | 0.008 | 0.009 | 0.552 | 0.554 | FALSE | 4 | 0.028 | 18336 | 0.763 | 4 | 0.001 | 462341 | 2.10E-08 | Adenocarcinoma |
| Sleeplessness | rs2014830 | T | C | T | C | -0.012 | -0.046 | 0.304 | 0.324 | FALSE | 3 | 0.028 | 18336 | 0.127 | 3 | 0.002 | 462341 | 8.90E-13 | Adenocarcinoma |
| Sleeplessness | rs2062113 | C | T | C | T | -0.010 | -0.064 | 0.568 | 0.560 | FALSE | 16 | 0.030 | 18336 | 0.027 | 16 | 0.002 | 462341 | 1.60E-10 | Adenocarcinoma |
| Sleeplessness | rs224032 | A | G | A | G | 0.008 | -0.007 | 0.550 | 0.524 | FALSE | 10 | 0.027 | 18336 | 0.805 | 10 | 0.001 | 462341 | 1.80E-08 | Adenocarcinoma |
| Sleeplessness | rs2297787 | A | T | A | T | -0.018 | 0.002 | 0.080 | 0.093 | FALSE | 10 | 0.046 | 18336 | 0.961 | 10 | 0.003 | 462341 | 9.60E-11 | Adenocarcinoma |
| Sleeplessness | rs2604551 | G | T | G | T | -0.008 | -0.053 | 0.640 | 0.642 | FALSE | 4 | 0.030 | 18336 | 0.068 | 4 | 0.002 | 462341 | 4.70E-08 | Adenocarcinoma |
| Sleeplessness | rs2644128 | G | C | G | C | 0.011 | 0.015 | 0.548 | 0.528 | FALSE | 1 | 0.027 | 18336 | 0.594 | 1 | 0.001 | 462341 | 1.00E-12 | Adenocarcinoma |
| Sleeplessness | rs2803296 | C | G | C | G | -0.009 | 0.003 | 0.544 | 0.487 | FALSE | 1 | 0.030 | 18336 | 0.913 | 1 | 0.001 | 462341 | 7.30E-09 | Adenocarcinoma |
| Sleeplessness | rs314280 | G | A | G | A | 0.010 | 0.035 | 0.547 | 0.555 | FALSE | 6 | 0.026 | 18336 | 0.208 | 6 | 0.001 | 462341 | 7.30E-11 | Adenocarcinoma |
| Sleeplessness | rs324017 | C | A | C | A | -0.010 | -0.054 | 0.705 | 0.708 | FALSE | 12 | 0.032 | 18336 | 0.085 | 12 | 0.002 | 462341 | 1.40E-09 | Adenocarcinoma |
| Sleeplessness | rs4572538 | T | C | T | C | -0.010 | 0.016 | 0.364 | 0.385 | FALSE | 2 | 0.030 | 18336 | 0.595 | 2 | 0.002 | 462341 | 7.70E-10 | Adenocarcinoma |
| Sleeplessness | rs4577309 | G | A | G | A | -0.009 | -0.045 | 0.534 | 0.527 | FALSE | 2 | 0.029 | 18336 | 0.112 | 2 | 0.001 | 462341 | 1.00E-08 | Adenocarcinoma |
| Sleeplessness | rs4886860 | C | G | C | G | -0.012 | -0.015 | 0.767 | 0.772 | FALSE | 15 | 0.034 | 18336 | 0.655 | 15 | 0.002 | 462341 | 1.80E-11 | Adenocarcinoma |
| Sleeplessness | rs56093896 | A | C | A | C | -0.012 | -0.010 | 0.214 | 0.220 | FALSE | 2 | 0.033 | 18336 | 0.766 | 2 | 0.002 | 462341 | 7.70E-12 | Adenocarcinoma |
| Sleeplessness | rs56330606 | G | A | G | A | 0.009 | 0.044 | 0.379 | 0.349 | FALSE | 19 | 0.030 | 18336 | 0.132 | 19 | 0.002 | 462341 | 1.20E-09 | Adenocarcinoma |
| Sleeplessness | rs56365214 | A | C | A | C | -0.015 | 0.071 | 0.156 | 0.149 | FALSE | 2 | 0.041 | 18336 | 0.074 | 2 | 0.002 | 462341 | 5.60E-13 | Adenocarcinoma |
| Sleeplessness | rs6561715 | A | T | A | T | -0.012 | -0.054 | 0.631 | 0.634 | FALSE | 13 | 0.030 | 18336 | 0.064 | 13 | 0.002 | 462341 | 4.80E-14 | Adenocarcinoma |
| Sleeplessness | rs6690017 | G | T | G | T | -0.010 | -0.031 | 0.409 | 0.413 | FALSE | 1 | 0.027 | 18336 | 0.276 | 1 | 0.002 | 462341 | 1.10E-11 | Adenocarcinoma |
| Sleeplessness | rs68094047 | T | C | T | C | 0.010 | -0.021 | 0.251 | 0.247 | FALSE | 12 | 0.031 | 18336 | 0.528 | 12 | 0.002 | 462341 | 1.70E-09 | Adenocarcinoma |
| Sleeplessness | rs6975972 | G | A | G | A | -0.009 | 0.011 | 0.579 | 0.572 | FALSE | 7 | 0.028 | 18336 | 0.709 | 7 | 0.002 | 462341 | 2.00E-09 | Adenocarcinoma |
| Sleeplessness | rs705219 | A | T | A | T | 0.013 | 0.154 | 0.887 | 0.894 | FALSE | 3 | 0.038 | 18336 | 0.001 | 3 | 0.002 | 462341 | 1.20E-08 | Adenocarcinoma |
| Sleeplessness | rs72924721 | T | C | T | C | 0.016 | -0.045 | 0.073 | 0.070 | FALSE | 11 | 0.052 | 18336 | 0.429 | 11 | 0.003 | 462341 | 1.10E-08 | Adenocarcinoma |
| Sleeplessness | rs7711696 | T | G | T | G | 0.011 | 0.075 | 0.305 | 0.294 | FALSE | 5 | 0.032 | 18336 | 0.013 | 5 | 0.002 | 462341 | 4.10E-12 | Adenocarcinoma |
| Sleeplessness | rs8180817 | C | G | C | G | -0.010 | -0.020 | 0.431 | 0.442 | FALSE | 7 | 0.027 | 18336 | 0.473 | 7 | 0.002 | 462341 | 2.70E-11 | Adenocarcinoma |
| Sleeplessness | rs931221 | A | T | A | T | 0.011 | 0.026 | 0.237 | 0.241 | FALSE | 12 | 0.032 | 18336 | 0.428 | 12 | 0.002 | 462341 | 1.30E-09 | Adenocarcinoma |
| Sleeplessness | rs9570080 | C | T | C | T | -0.011 | 0.004 | 0.344 | 0.343 | FALSE | 13 | 0.029 | 18336 | 0.897 | 13 | 0.002 | 462341 | 1.60E-11 | Adenocarcinoma |
| Sleeplessness | rs9845387 | A | C | A | C | -0.022 | -0.074 | 0.040 | 0.042 | FALSE | 3 | 0.064 | 18336 | 0.319 | 3 | 0.004 | 462341 | 7.10E-09 | Adenocarcinoma |
| Sleeplessness | rs9894577 | A | G | A | G | 0.013 | 0.018 | 0.318 | 0.332 | FALSE | 17 | 0.030 | 18336 | 0.555 | 17 | 0.002 | 462341 | 1.30E-16 | Adenocarcinoma |
| Sleeplessness | rs9906181 | G | A | G | A | -0.009 | 0.010 | 0.688 | 0.673 | FALSE | 17 | 0.036 | 18336 | 0.785 | 17 | 0.002 | 462341 | 2.40E-08 | Adenocarcinoma |
