# Supplementary material for: Association between Sleep Traits and Lung Cancer: A Mendelian Randomization Study
Source: J Immunol Res. 2021 Jun 21;2021:1893882. doi: 10.1155/2021/1893882 (PMC8238591; doi:10.1155/2021/1893882)
Supplement: Supplementary Materials — Supplementary Table 1: two-sample Mendelian randomization estimations showing the effect of sleep traits on cancer using the MR Egger, weighted median, and weighted mode method. Supplementary Table 2: sensitivity analysis performed by Egger regression intercept and heterogeneity test. Supplementary Table 3: SNPs of sleep traits extracted from UK Biobank with statistically significant threshold [P < 5 × 10^−8^; linkage disequilibrium (LD) r2 < 0.001, LD distance > 10000 kb]. Supplementary Table 4: SNPs used in two-sample Mendelian randomization analysis. Supplementary Table 5: outliers selected by RadialMR and the reanalysis results after excluding outliers. Supplementary Table 6: multivariable two-sample Mendelian randomization estimation showing the effects of different sleep traits on lung cancer. [file 1893882.f1.zip › Supplementary Table 5 (1).docx]

Supplementary Table 5: Outliers selected by RadialMR and the reanalysis results after excluding outliers.

| Outcome | Exposure | No | SNP (outliers) | Q_statistic | p.value | exclude outliers | method | nsnp | | OR (95% CI) | se | | | pval | |
| --- | --- | --- | --- | --- | --- | --- | --- | --- | --- | --- | --- | --- | --- | --- | --- |
| **Lung cancer** | chronotype | 1 | rs10280205 | 4.20 | 0.04 |  | MR Egger | 129 | 0.54 (0.24-1.21) | | | | 0.41 | | 0.14 |
|  |  | 2 | rs10742179 | 6.07 | 0.01 |  | Weighted median | 129 | | 0.98 (0.68-1.40) | | | 0.18 | 0.90 | |
|  |  | 3 | rs111761918 | 7.51 | 0.01 |  | Inverse variance weighted | 129 | | 0.90 (0.70-1.16) | | | 0.13 | 0.42 | |
|  |  | 4 | rs12249410 | 7.94 | 0.00 |  | Weighted mode | 129 | | 1.42 (0.47-4.36) | | | 0.57 | 0.54 | |
|  |  | 5 | rs17716502 | 6.65 | 0.01 |  |  |  | |  | | |  |  | |
|  |  | 6 | rs1927719 | 4.54 | 0.03 |  |  |  | |  | | |  |  | |
|  |  | 7 | rs72720396 | 3.97 | 0.05 |  |  |  | |  | | |  |  | |
|  |  | 8 | rs80097534 | 7.09 | 0.01 |  |  |  | |  | | |  |  | |
|  |  |  |  |  |  |  |  |  | |  | | |  |  | |
|  | Sleep duration | 1 | rs11621908 | 7.38 | 0.01 |  | MR Egger | 45 | | 1.12 (0.2-6.37) | | | 0.89 | 0.90 | |
|  |  | 2 | rs12518468 | 5.25 | 0.02 |  | Weighted median | 45 | | 0.61 (0.28-1.32) | | | 0.40 | 0.21 | |
|  |  | 3 | rs174564 | 8.70 | 0.00 |  | Inverse variance weighted | 45 | | 0.46 (0.28-0.75) | | | 0.26 | 0.00 | |
|  |  | 4 | rs2734831 | 4.13 | 0.04 |  | Weighted mode | 45 | | 0.88 (0.26-3.01) | | | 0.63 | 0.84 | |
|  |  | 5 | rs374153 | 3.89 | 0.05 |  |  |  | |  | | |  |  | |
|  |  | 6 | rs4767550 | 4.83 | 0.03 |  |  |  | |  | |  | |  | |
|  |  | 7 | rs55658675 | 5.47 | 0.02 |  |  |  | |  | |  | |  | |
|  |  | 8 | rs62444917 | 4.04 | 0.04 |  |  |  | |  | |  | |  | |
|  |  | 9 | rs7711696 | 4.94 | 0.03 |  |  |  | |  | |  | |  | |
|  |  | 10 | rs7831557 | 5.30 | 0.02 |  |  |  | |  | |  | |  | |
|  |  | 11 | rs915416 | 6.86 | 0.01 |  |  |  | |  | |  | |  | |
|  |  |  |  |  |  |  |  |  | |  | |  | |  | |
|  | Nap during day | 1 | rs11071755 | 6.72 | 0.01 |  | MR Egger | 74 | | 2.74 (0.46-16.37) | | 0.91 | | 0.27 | |
|  |  | 2 | rs13284688 | 6.62 | 0.01 |  | Weighted median | 74 | | 2.20 (1.03-4.67) | | 0.38 | | 0.04 | |
|  |  | 3 | rs17158413 | 6.35 | 0.01 |  | Inverse variance weighted | 74 | | 1.79 (1.09-2.94) | | | 0.25 | 0.02 | |
|  |  | 4 | rs174541 | 17.05 | 0.00 |  | Weighted mode | 74 | | 3.33 (0.74-15.04) | | | 0.77 | 0.12 | |
|  |  | 5 | rs4402351 | 8.40 | 0.00 |  |  |  | |  | | |  |  | |
|  |  |  |  |  |  |  |  |  | |  | | |  |  | |
| **Squamous cell lung cancer** | Chronotype | 1 | rs10175975 | 5.16 | 0.02 |  | MR Egger | 121 | | 0.65 (0.20-2.13) | | | 0.60 | 0.48 | |
|  |  | 2 | rs10742179 | 6.37 | 0.01 |  | Weighted median | 121 | | 0.56 (0.34-0.93) | | | 0.26 | 0.02 | |
|  |  | 3 | rs11183201 | 5.17 | 0.02 |  | Inverse variance weighted | 121 | | 0.70 (0.49-1.00) | | | 0.18 | 0.05 | |
|  |  | 4 | rs112555644 | 5.25 | 0.02 |  | Weighted mode | 121 | | 0.25 (0.07-0.91) | | | 0.66 | 0.04 | |
|  |  | 5 | rs12965577 | 5.20 | 0.02 |  |  |  | |  | | |  |  | |
|  |  | 6 | rs35101255 | 5.92 | 0.01 |  |  |  | |  | | |  |  | |
|  |  | 7 | rs4595586 | 5.44 | 0.02 |  |  |  | |  | | |  |  | |
|  |  | 8 | rs72720396 | 7.47 | 0.01 |  |  |  | |  | | |  |  | |
|  |  | 9 | rs76223855 | 5.01 | 0.03 |  |  |  | |  | | |  |  | |
|  |  |  |  |  |  |  |  |  | |  | | |  |  | |
|  | Sleep duration | 1 | rs11621908 | 16.32 | 0.00 |  | MR Egger | 49 | | 0.97 (0.06-15.12) | | | 1.40 | 0.98 | |
|  |  | 2 | rs12518468 | 6.18 | 0.01 |  | Weighted median | 49 | | 0.90 (0.30-2.68) | | | 0.56 | 0.84 | |
|  |  | 3 | rs174564 | 4.33 | 0.04 |  | Inverse variance weighted | 49 | | 0.52 (0.25-1.12) | | | 0.39 | 0.10 | |
|  |  | 4 | rs374153 | 8.79 | 0.00 |  | Weighted mode | 49 | | 1.36 (0.21-8.75) | | | 0.95 | 0.75 | |
|  |  | 5 | rs6889592 | 5.09 | 0.02 |  |  |  | |  | | |  |  | |
|  |  | 6 | rs7517981 | 5.00 | 0.03 |  |  |  | |  | | |  |  | |
|  |  | 7 | rs915416 | 5.42 | 0.02 |  |  |  | |  | | |  |  | |
|  |  |  |  |  |  |  |  |  | |  | | |  |  | |
| **Lung Adenocarcinoma** | Getting up in morning | 1 | rs10280205 | 4.86 | 0.03 |  | MR Egger | 124 | | 0.36 (0.12-1.07) | | | 0.55 | 0.07 | |
|  |  | 2 | rs12249410 | 4.72 | 0.03 |  | Weighted median | 124 | | 1 .00 (0.61-1.63) | | | 0.25 | 1.00 | |
|  |  | 3 | rs17575798 | 5.72 | 0.02 |  | Inverse variance weighted | 124 | | 0.95 (0.67-1.35) | | | 0.18 | 0.78 | |
|  |  | 4 | rs56076457 | 6.47 | 0.01 |  | Weighted mode | 124 | | 1.71 (0.52-5.67) | | | 0.61 | 0.38 | |
|  |  | 5 | rs610590 | 4.05 | 0.04 |  |  |  | |  | | |  |  | |
|  |  | 6 | rs9831488 | 5.82 | 0.02 |  |  |  | |  | | |  |  | |
|  |  |  |  |  |  |  |  |  | |  | | |  |  | |
|  | Nap during day | 1 | rs10840017 | 3.96 | 0.05 |  | MR Egger | 70 | | 5.68 (0.38-84.73) | | | 1.38 | 0.21 | |
|  |  | 2 | rs11071755 | 5.14 | 0.02 |  | Weighted median | 70 | | 2.14 (0.69-6.65) | | | 0.58 | 0.19 | |
|  |  | 3 | rs11258652 | 6.72 | 0.01 |  | Inverse variance weighted | 70 | | 2.35 (1.09-5.06) | | | 0.39 | 0.03 | |
|  |  | 4 | rs13266972 | 6.76 | 0.01 |  | Weighted mode | 70 | | 2.60 (0.34-19.87) | | | 1.04 | 0.36 | |
|  |  | 5 | rs17265513 | 6.36 | 0.01 |  |  |  | |  | | |  |  | |
|  |  | 6 | rs174541 | 11.64 | 0.00 |  |  |  | |  | | |  |  | |
|  |  | 7 | rs3810484 | 4.57 | 0.03 |  |  |  | |  | | |  |  | |
|  |  | 8 | rs4402351 | 5.64 | 0.02 |  |  |  | |  | | |  |  | |
|  |  | 9 | rs62425620 | 4.98 | 0.03 |  |  |  | |  | | |  |  | |
