# Supplementary material for: Association between Sleep Traits and Lung Cancer: A Mendelian Randomization Study
Source: J Immunol Res. 2021 Jun 21;2021:1893882. doi: 10.1155/2021/1893882 (PMC8238591; doi:10.1155/2021/1893882)
Supplement: Supplementary Materials — Supplementary Table 1: two-sample Mendelian randomization estimations showing the effect of sleep traits on cancer using the MR Egger, weighted median, and weighted mode method. Supplementary Table 2: sensitivity analysis performed by Egger regression intercept and heterogeneity test. Supplementary Table 3: SNPs of sleep traits extracted from UK Biobank with statistically significant threshold [P < 5 × 10^−8^; linkage disequilibrium (LD) r2 < 0.001, LD distance > 10000 kb]. Supplementary Table 4: SNPs used in two-sample Mendelian randomization analysis. Supplementary Table 5: outliers selected by RadialMR and the reanalysis results after excluding outliers. Supplementary Table 6: multivariable two-sample Mendelian randomization estimation showing the effects of different sleep traits on lung cancer. [file 1893882.f1.zip › Supplementary Table 6 (1).docx]

Supplementary Table 6: Multivariable Two-sample Mendelian randomization estimation showing the effects of different sleep traits on lung cancer.

| Outcome | Exposure | nSNP | OR (95% CI) | P-value |
| --- | --- | --- | --- | --- |
| Lung cancer | Sleeplessness | 28 | 1.12 (0.47-2.64) | 0.80 |
|  | Sleep duration | 52 | 0.42 (0.21-0.82) | 0.01 |
|  | Nap during day | 64 | 1.60 (0.84-3.04) | 0.15 |
| Squamous cell lung cancer | Sleeplessness | - | - | - |
|  | Sleep duration | 52 | 0.57 (0.23-1.41) | 0.22 |
|  | Nap during day | - | - | - |
| Lung Adenocarcinoma | Sleeplessness | 28 | 7.85 (2.60-23.75) | <0.01 |
|  | Sleep duration | - | - | - |
|  | Nap during day | - | - | - |
